# K-Domain Technology: Constitutive Expression of a Blueberry Keratin-Like Domain Mimics Expression of Multiple MADS-Box Genes in Enhancing Maize Grain Yield

**DOI:** 10.3389/fpls.2021.664983

**Published:** 2021-05-07

**Authors:** Guo-qing Song, Xue Han

**Affiliations:** Plant Biotechnology Resource and Outreach Center, Department of Horticulture, Michigan State University, East Lansing, MI, United States

**Keywords:** flowering mechanism, MADS-box genes, MIKC-type MADS-box protein, *SOC1*, yield increase, *Zea mays*

## Abstract

MADS-box genes are considered as the foundation of all agronomic traits because they play essential roles in almost every aspect of plant reproductive development. Keratin-like (K) domain is a conserved protein domain of tens of MIKC-type MADS-box genes in plants. K-domain technology constitutively expresses a K-domain to mimic expression of the K-domains of other MADS-box genes simultaneously and thus to generate new opportunities for yield enhancement, because the increased K-domains can likely prevent MADS-domain proteins from binding to target DNA. In this study, we evaluated utilizing the K-domain technology to increase maize yield. The K-domain of a blueberry’s *SUPPRESSOR of CONSTITUTIVE EXPRESSION OF CONSTANS 1* (*VcSOC1K*) has similarities to five MADS-box genes in maize. Transgenic maize plants expressing the *VcSOC1K* showed 13–100% of more grain per plant than the nontransgenic plants in all five experiments conducted under different experimental conditions. Transcriptome comparisons revealed 982 differentially expressed genes (DEGs) in the leaves from 83-day old plants, supporting that the K-domain technology were powerful and multiple functional. The results demonstrated that constitutive expression of the *VcSOC1K* was very effective to enhance maize grain production. With the potential of mimicking the K-domains of multiple MADS-box genes, the K-domain technology opens a new approach to increase crop yield.

## Introduction

Plant-specific MIKC proteins have conserved MADS (M-), intervening (I-), keratin-like (K-), and *C*-terminal (*C*-) domains (Theissen et al., 1996; Gramzow and Theissen, 2010). These MIKC proteins consist of MIKC^∗^ and MIKC^*c*^ (classical MIKC) subgroups and are key regulators in plant reproductive processes (Verelst et al., 2007; Adamczyk and Fernandez, 2009; Smaczniak et al., 2012; Liu et al., 2013; Dreni and Kater, 2014; Gramzow and Theissen, 2015; Dreni and Zhang, 2016). For example, six MIKC^∗^-type genes (*AGAMOUS-LIKE 30* (*AGL30*), *AGL65*, *AGL66*, *AGL67*, *AGL94*, and *AGL104*) play a significant role in regulating pollen development in *Arabidopsis thaliana* (Verelst et al., 2007; Kwantes et al., 2012; Liu et al., 2013). The MIKC^*c*^ genes play specific roles in the ABC model of floral development and in timing plant flowering (Amasino, 2010; Lee and Lee, 2010; Wellmer and Riechmann, 2010; Heijmans et al., 2012; Smaczniak et al., 2012).

MADS-box genes were frequent targets of selection during maize domestication and improvement (Zhao et al., 2011; Schilling et al., 2018). They play essential roles in every aspect of plant reproductive development and were considered as the jack of all traits (Heuer et al., 2001; Schilling et al., 2018). For MIKC proteins, the I-domain defines specificity in the formation of DNA binding dimers (Masiero et al., 2011); the K-domain contributes to specificity in protein-protein interactions (Kaufmann et al., 2005; Rumpler et al., 2015). Both the K and C domains function in the formation of higher-order protein complexes, and the C domain also determines the specificity of interactions of MADS-box proteins (van Dijk et al., 2010; Liu et al., 2013). Of the MIKC^*c*^ gene clades, *SUPPRESSOR of* CONSTITUTIVE EXPRESSION OF CONSTANS 1 (*SOC1*) is a positive regulator of the downstream MADS-box genes such as *APETALA1* (*AP1*) and *FRUITFUL* (*FUL*)/*AGAMOUS*-like 8 (*AGL8*) (Lee and Lee, 2010; Alter et al., 2016). Due to their regulatory roles, many of the MIKC^*c*^ genes have a potential to change agronomic traits (Cacharron et al., 2000; Takatsuji and Kapoor, 2002; Lee et al., 2004; Podila et al., 2005; Ryu et al., 2009; Bae et al., 2011; Giovannoni et al., 2013; Alter et al., 2016). For example, a maize *ZMM28* gene (patent application # WO2008148872A1), which is a homolog of the *AGL8*, has been successfully applied to enhance grain yield by its constitutive expression (Munster et al., 2002; Anderson et al., 2019a,b; Catron, 2019).

Being part of the K domain of the blueberry’s (*Vaccinium corymbosum* L.) SOC1 gene (VcSOC1K), overexpression of the VcSOC1K was found effective in promoting flowering, reducing plant height, enhancing abiotic tolerance, and increasing blueberry yield potential through its broad impact the expression of numerous genes (Song et al., 2013; Song and Chen, 2018). This laid a foundation of the K-domain technology, which utilizes a constitutively expressing K-domain to mimic or affect the expression of multiple MADS-box genes simultaneously. In this study, we evaluated the K-domain technology for maize yield increase. We provide the phenotypic data of transgenic maize plants containing a constitutively expressed VcSOC1K (VcSOC1K-CX) from five experiments conducted under different conditions. We show the data of transcriptome comparison to reveal a broad effect of VcSOC1K-CX on plant development at transcript levels. The K-domain technology opens a new approach to enhance crop yield potential by mimicking expression of the K-domains of multiple MADS-box genes.

## Materials and Methods

### Constructs and Plant Transformation

Maize SOC1 gene (*ZmSOC1* or *ZmMADS1*) was cloned from the cDNA of maize inbred line B104. The protein sequence of the cloned 696-bp *ZmSOC1* is identical to that derived from the HQ858775.1 in the GenBank. The ZmSOC1 and VcSOC1K protein sequences were aligned using Clustal Omega at EBI with default parameters^1^.

The *VcSOC1K* was previously cloned into the T-DNA region of the binary vector pBI121 between the CaMV 35S promoter and the *Nos* terminator for constitutive expression (Song and Chen, 2018). The CaMV 35S-*VcSOC1K-Ocs* expression cassette in the PBI121 vector was released by a digestion using *Hin*d III and *Eco*R I, purified from gel, and then ligated to the T-DNA region of the *Hin*d III- and *Eco*R I-digested binary vector pTF101.1. The pTF101.1 contains the *bialaphos resistance* (*bar*) gene under the CaMV 35S promoter for selection of transformed plant cells using glufosinate (GS) herbicide. The resulting pTF101.1-*VcSOC1K* was verified by sequencing the *VcSOC1K* and was then transformed into *Agrobacterium tumefaciens* strain EHA101.

Transformation of the pTF101.1-*VcSOC1K* into maize cultivar Hi-II (A188 × B73) calluses was conducted at the Plant Transformation Facility of Iowa State University. The first generation (T_0_) of transgenic (TR) Hi-II plants were backcrossed with nontransgenic inbred line B73 to produce first generation of backcross (BC_1_) seeds, which have about 75% of the B73 genetic background. T_0_ plants from separate callus clusters were defined as independent transgenic lines. BC_1_ seeds from 18 transgenic lines were obtained. Transgenic BC_1_ plants that showed PCR-positive for both the *bar* gene and the *VcSOC1K* were crossed with inbred line B73 to produce BC_2_ seeds, which have about 87.5% of the B73 genetic background.

### Sequence Analysis of Maize MADS-Box Genes

The amino acid sequence of the *VcSOC1K* was used to search the sequence database “all gene model protein sequences” at Maize Genetics and Genomics Database (MaizeGDB)^2^ using BLAST program blastp. The BLAST hits with *E*-value cutoff of 1e-4 were retained and annotated by BLAST against the database at GenBank. Protein sequence alignment was conducted using CLC Sequence Viewer 8.0. Phylogenetic tree analysis was performed using the Maximum Likelihood method conducted in MEGA X (Jones et al., 1992; Kumar et al., 2018; Stecher et al., 2020).

### Plant Phenotyping

To collect phenotypic data of the BC_1_ plants, three experiments started on May 17th, June 11th, and June 25th were conducted in 2018, and one experiment started on may 11th was performed in 2019. Plants of four transgenic lines were evaluated in all four experiments, and five additional transgenic lines were also evaluated in one or two experiments. For all of the four experiments, BC_1_ seeds were germinated in water-soaked Suremix Perlite planting medium (Michigan Grower Products Inc., Galesburg, MI) in 4-inch plastic pots (8.9 cm width × 12.7 cm height). Individual BC_1_ plant was transplanted to a 4-gallon pot (top diameter 30 cm, bottom diameter 24 cm, depth 27 cm) and the plants were grown in a secured courtyard under natural environmental conditions at Michigan State University, East Lansing, Michigan. All of the plants were irrigated and fertilized as needed. During the summer time, plants were watered every other day and fertilized weekly using 20-20-20 fertilizer. Young leaves of 30 to 40-day old plants, 0.5 g per plant, were collected for each plant, frozen in liquid nitrogen, and stored in a freezer at −80°C for DNA isolation. To avoid biases in phenotypic data collection, verification of the transgenic plants through polymerase chain reaction (PCR) was conducted after phenotypic data collection.

To collect phenotypic data of the BC_2_ plants, one field test started on May 26th was conducted in 2020. One of the four transgenic lines evaluated in the BC_1_ generation was tested in six field plots. A total of 30 plants in three lanes were randomly grown in each of the six plots, including three plots for a high planting density of 40,000 plants/acre and another three for a low planting density of 32,000 plants/acre. Two extra lanes of B73 plants for each plot were used as protection lanes (Supplementary Figure 1). A drip irrigation system was installed in the field for plant irrigation as needed.

Phenotypic data collections included plant height, seed germination date, date of tassel and silk appearance, the total number of stem nodes and leaves, the number of cobs, dry weight of aerial parts without ears, dry weight of ear(s) excluding husk(s), and dry wright of grains. Plant heights measured during plant growth refer to stalk heights from the soil surface to the node of the highest leaf. The final heights of the maize plants refer to stalk heights from the soil surface to the base of the first branch of tassels at the harvest time. All of the plants for each experiment were harvested at a same time after they reached full physiological maturity in late Octobers. The ear(s) of each plant were collected in a paper bag and dried at 25°C for over two months in the lab prior to weighing the dry weights of cob(s) and grains. Grain quality from BC_2_ transgenic and nontransgenic null segregate (NT) and B73 plants was measured using a Grain Analyser (Infratec 1241, FOSS Analytical AB, Denmark).

### Transgene Detection

DNA was isolated from about 200 mg of leaf tissues for each sample using the cetyltrimethylammonium bromide (CTAB) method (Doyle and Doyle, 1987). Two pairs of primers, bar-F and bar-R for the *bar* gene, 35S-F (3′ portion of the *CaMV 35S* promoter) and SOK for the *VcSOC1K* gene (Supplementary Table 1), were used to detect the presence of transgenes in each sample. PCR reaction conditions for all primer pairs started with an initial denaturation for two min at 94°C, 30 cycles of 45 s at 94°C, 60 s at 58°C and 90 s at 72°C, and a final extension for 10 min at 72°C. All amplified PCR and RT-PCR products were separated on 1.0% agarose gel containing ethidium bromide and visualized and photographed under UV light.

### RNA Sequencing and Transcriptome Analysis

The third leaf from the top of 83-day old plants at the Blister (R2) stage, which is a reproductive growth stage occurs 10–14 days after silking, were harvested, frozen immediately in liquid nitrogen, and stored at −80°C in a freezer for RNA isoation. Three transgenic and three NT plants from one field plot were used as individual biological replicates. Total RNA of each sample was isolated from about 500 mg young leaf tissues using a separate CTAB method (Zamboni et al., 2008) and was purified using RNeasy Mini Kit (Qiagen, Valencia, CA, United States). On-Column DNase digestion with the RNase-free DNase Set was used to remove DNA in the RNA samples (Qiagen, Valencia, CA, United States). RNA quality was determined using the High Sensitivity RNA ScreenTape system (Agilent technologies, Santa Clara, CA, United States). All of the RNA samples used for RNA sequencing had an RNA integrity number (RIN) equivalent score greater than 5.0.

The RNA samples were sequenced (150 bp-paired end reads) using the Illumina HiSeq4000. All sequencing was performed at the Research Technology Support Facility at Michigan State University (East Lansing, Michigan, United States). The FastQC program^3^ was used to assess the quality of sequencing reads for the per base quality scores. A total of 7.3–11.0 million pair-reads (MR) for each of the six biological samples with average scores ranging from 38.4 to 39.4 were obtained for transcriptome analysis. A transcriptome reference of BC_1_ plants (ZmTrinity) assembled from about 100 MR of multiple NT and transgenic lines using Trinity/2.8.5 was used to conduct differential expression analysis (Haas et al., 2013). The differentially expressed transcripts (DETs) with the false discovery rate (FDR) value below 0.05 were used for further analyses of different pathway genes. The transcriptome reference ZmTrinity was annotated using Trinotate (Bryant et al., 2017).

Pathway genes of nine phytohormones in *Arabidopsis*, including auxin, cytokinin, ABA, ethylene, gibberellin, brassinosteroid, jasmonic acid, salicylic acid, and strigolactones, were retrieved from RIKEN Plant Hormone Research Network^4^. Similarly, pathway genes of sugar in *Arabidopsis* were identified. These *Arabidopsis* hormone, MADS-box, and sugar genes were used as queries to blast against the transcriptome reference ZmTrinity and the isoforms showing *e*-values less than −20 were identified and used for transcriptome comparisons. Flowering pathway genes in *Arabidopsis* and cereals (Walworth et al., 2016) were used to analyze flowering-related DETs identified in this study. Cytoscape 3.8.2 was used to construct gene networks of overrepresented gene ontology (GO) terms for the selected DETs under BiNGO’s default parameters with selected ontology file “GOSlim_Plants” and selected organism “*A. thaliana*” (Shannon et al., 2003; Maere et al., 2005).

Quantitative reverse transcript PCR (RT-qPCR) using SYBR Green system (LifeTechnologies, Carlsbad, CA, United States) was conducted to check the selected transcripts. The primers were designed according to the RNA-seq data, ZmActin1 was used to normalize the RT-qPCR results (Supplementary Table 1). RT-qPCR was performed on a Roche LightCycler 480 Instrument II (Roche). The reaction conditions for RT-qPCR were 95°C for 5 min, 45 cycles of 30 s at 95°C, 45 s at 62°C and 30 s at 72°C. Transcript levels within samples were normalized to Actin. Fold changes were calculated using 2^–ΔΔCt^, where ΔΔCt = (Ct_TARGET_ − Ct_NOM_)_transgenic_ − (Ct_TARGET_ − Ct_NOM_)_nontransgenic_. Three biological samples and three technical replicates were used for the analysis of each transgenic and nontransgenic line.

### Statistical Analysis

Statistical analysis of the phenotypic data was conducted using ANOVA and TukeyHSD in RStudio (Version 1.3.1093).

## Results and Discussion

### VcSOC1K Sequence Has Similarities to the Proteins of Multiple Maize MADS-Box Genes

**FIGURE 1:**
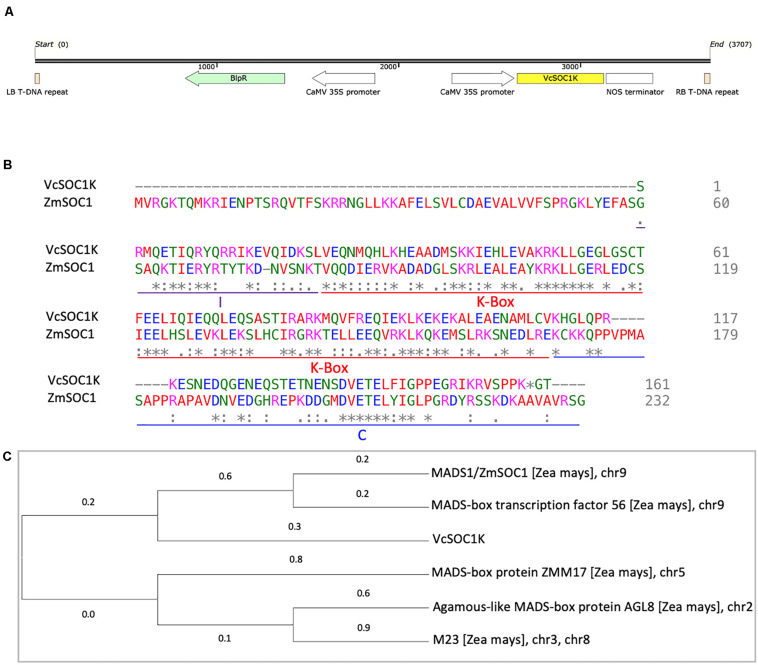
Comparison of VcSOC1K and the selected maize MADS-box genes. **(A)** Diagram of the T-DNA region of the pTF101.1-ZmSOC1 vector. LB: left border. RB: right border. **(B)** Protein sequence alignment of the VcSOC1K and ZmSOC1 (GenBank #: HQ858775.1). **(C)** Phylogenetic analysis of maize MADS-box proteins that showed similarities to the VcSOC1K using the Maximum Likelihood method conducted in MEGA X. There were a total of 172 positions in the final dataset. The tree with the highest log likelihood (–1672.77) is shown. Chromosome numbers refer to the positions in the sequence database Zm-B73-REFERENCE-NAM-5.0 (the maize representative genome, B73, version 5) at Maize Genetics and Genomics Database (MaizeGDB).

The *VcSOC1K*, lacking the MADS box domain, had 36.9% of identity to the maize *SOC1* gene (*ZmSOC1/ZmMADS1*) (Figures 1A,B). It contained both the K-, I-, and C-domains (Figure 1B). A total of five MADS-box genes on five chromosomes in the maize B73 representative genome showed similarities to the VcSOC1K (Figure 1C). This was the foundation of our hypothesis of utilizing constitutive expression of the *VcSOC1K* to mimic expression of the K-domains of multiple MADS-box genes and thus to regulate plant development for grain yield increase in this study.

### VcSOC1-OX Enhanced Grain Production

**FIGURE 2:**
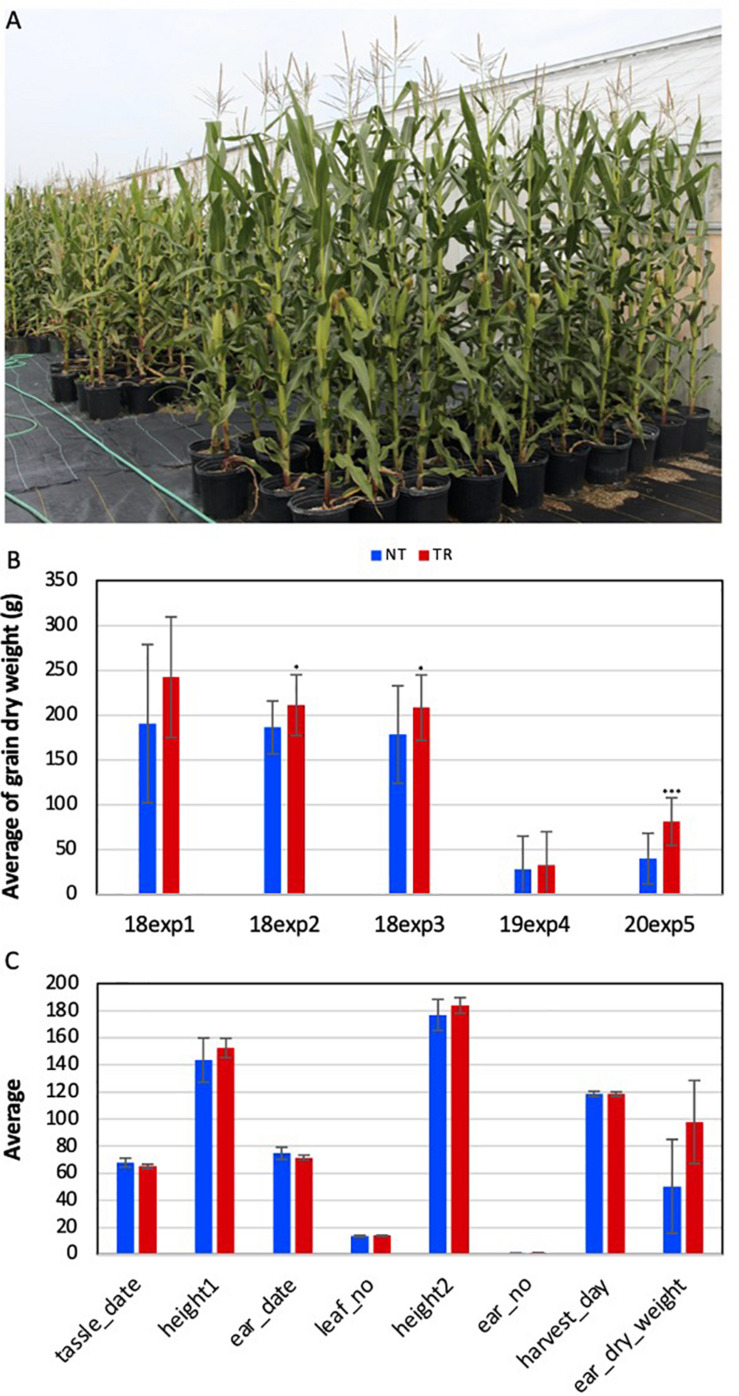
Phenotype assessment of transgenic *VcSOC1K*-CX (TR) and nontransgenic null segregant (NT) maize plants of BC_1_ and BC_2_ lines. The BC_1_ plants were evaluated in three experiments in 2018 [i.e., 18exp1 (No. of NT = 7, No. of TR = 10), 18exp2 (No. of NT = 25, No. of TR = 16), and 18exp3 (No. of NT = 38, No. of TR = 15)] and one experiment (19exp4, No. of NT = 44, No. of TR = 40) in 2019. The BC_2_ plants were evaluated in one field test (20exp5, No. of NT = 87, No. of TR = 86) in 2020. **(A)** BC_1_ plants randomly grown for phenotyping. **(B)** Averages of grain dry weight per plant. **(C)** Phenotypic data of all BC_2_ plants. Tassle date: Days from sowing to the appearance of tassle. Height1: Plant height at the time of tassel appearance. Ear date: Days from sowing to the appearance of silk. Leaf no: Total number of leaves of a mature plant. Height2: Plant height at the time of harvest. Ear no: Total number of ears of a mature plant. Harvest day: Days from sowing to harvest. Ear dry weight: Dry weight of the ears from each plant. Bars indicate standard deviation. Significance codes: “***” < 0.001, “**” < 0.01, and “*” < 0.05.

Five experiments were conducted to evaluate the phenotypic changes in VcSOC1K-CX plants under five environmental conditions in three years. Pot-growing VcSOC1K-CX BC_1_ plants from nine transgenic lines in four experiments and field-growing VcSOC1K-CX BC_2_ plants at two planting densities from one transgenic line were compared with the NT plants (Figure 2A and Supplementary Figure 1). Of the nine agronomic traits investigated, the VcSOC1K-CX plants had a higher grain production per plant than the NT plants in all five experiments (Figure 2B and Supplementary Table 2). The increases for VcSOC1K-CX BC_1_ plants ranged from 13 to 27%. Incredibly, for the 180 BC_2_ plants tested in the field in 2020, the average dry grain weight for the VcSOC1K-CX BC_2_ (81 g/plant) was two-fold as many as that for the NT plants (40 g/plant) (Figure 2B). This difference for the BC_2_ plants, compared to field-growing BC_1_ plants, was due likely to the increased abiotic and biotic stresses caused by the field conditions. All of the other traits (e.g., flowering time, leaf number, and plant height) showed no significant changes (Figure 2C and Supplementary Table 2). Quality of the grains from the BC_2_ plants was measured. The grains from the VcSOC1K-CX plants showed no significant differences from those of the NT plants (Supplementary Table 3).

With the MADS box of MIKC-type SOC1 protein removal, constitutive expression of the truncated *SOC1* genes resulted in *SOC1*-deficient phenotypes in model plants, petunia, Arabidopsis, and *Brachypodium* (Ferrario et al., 2004; Seo et al., 2012). Overexpression of a truncated petunia SOC1 gene containing the K-domain and *C*-region delayed flowering in transgenic petunia plants (Ferrario et al., 2004). Similarly, in both Arabidopsis and *Brachypodium*, expression of the truncated SOC1 genes containing either the K-domain only or the K-domain and I-region inactivated the SOC1 and caused delay of flowering (Seo et al., 2012). In contrast, unlike the other truncated SOC1 genes reported (Ferrario et al., 2004; Seo et al., 2012), when the truncated VcSOC1K lacking only the MADS box were constitutively expressed, both VcSOC1K-CX tobacco or VcSOC1K-OX blueberry plants had *SOC1*-OX phenotypes (Song et al., 2013; Song and Chen, 2018). Surprisingly, the VcSOC1K-CX maize plants in this study showed neither *SOC1*-deficient phenotype in delayed flowering nor obvious SOC1-OX phenotypes of promoted flowering and plant dwarfing (Alter et al., 2016).

### VcSOC1K-CX Affected the Expression of Numerous Genes

Kernel Blister Stage (growth stage R2) is a reproductive growth stage occurs 10–14 days after silking. The R2 stage is of importance for the determination of grain yield. At this stage, we found the leaves of the VcSOC1K-CX BC_1_ plants were often greener leaves than the NT plants. Thus, we conducted transcriptome comparisons between the VcSOC1K-CX and the NT BC_2_ plants. The comparison revealed 2,247 DETs, which were annotated to 982 differentially expressed genes (DEGs) with *E*-value cutoff of 1e-19 (Supplementary Table 4). RT-qPCR analysis of seven selected DEGs were consistent with those from RNA-sequencing data, suggesting that the RNA-seq data were reliable (Supplementary Figure 2).

Of the 982 DEGs, we further identified 21 DEGs in the flowering pathway and 41 DEGs related to phytohormones, including abscisic acid (9 DEGs), auxin (9), brassinosteroid (8), cytokinin (5), ethylene (1), gibberellin (8), and Jasmonate (1). Additionally, there were 3 DEGs of MADS-box genes, 18 DEGs related to sucrose synthesis, and 28 DEGS in the family of mitogen-activated protein kinase (MAPK) related to plant resistance to abiotic and biotic stresses (Bigeard and Hirt, 2018; Krysan and Colcombet, 2018; He et al., 2020). Remarkably, of these essential DEGs, greater than 100-fold changes occurred for 10 repressed and 40 up-regulated DEGs (Table 1). The examples of these DEGs indicated that VcSOC1K-CX could affect grain production, at least, through flowering, phytohormones, MAPK-mediated signaling (Bigeard and Hirt, 2018), or photosynthetic sucrose synthesis (Stein and Granot, 2019), although these DEGs only represented the changes in a specific tissue at a specific developmental stage. For instance, the upregulated ZEAXANTHIN EPOXIDASE Gene (ZEP_ORYSJ) could increase plant resistance to osmotic and drought stresses, seed development and dormancy (Agrawal et al., 2001; Cao et al., 2018). The increased expression of the floral homeotic protein APETALA 2 (AP2_ARATH) could play a broad role in flower and seed development by controlling the expression of other floral organ identity genes (Jofuku et al., 1994; Krogan et al., 2012). The upregulated Mitogen-activated protein kinase kinase kinase YODA (YODA_ARATH) could enhance the regulation of florescence architecture due to its role in promoting extra-embryogenic fate (Lukowitz et al., 2004; Meng et al., 2012). Alpha, alpha-trehalose-phosphate synthase [UDP-forming] 1 (TPS1_ARATH) plays a critical role in vegetative growth and transition to flowering, embryo development and growth, and starch and sucrose degradation (Blazquez et al., 1998; van Dijken et al., 2004; Avonce et al., 2005; Gomez et al., 2006). It was likely that the increased expression of TPS10_ARATH enhanced grain yield (Table 1). More studies at protein levels are still needed to find out how and why the expression of the truncated VcSOC1K with the MADS box removal had such a broad impact on gene expressions.

**TABLE 1:** Differentially expressed transcripts (DETs) of flowering pathway, hormone genes, MADS-box genes, and mitogen-activated protein kinase (MAPK) genes in maize new leaves from 83-day old plants.

Subject id	Log_2_FC	Log_2_CPM	P_Value	FDR	Annotation	Annotation_ e_value	Pathway_gene	Pathway	BLAST_e_value using pathway gene
DN16090_c0_g3_i5	−1.14	4.54	1E-03	4E-02	ALDO2_MAIZE	0E+00	AT5G67030.1	ABA	0E+00
DN14992_c0_g1_i6	−8.43	−0.59	4E-07	3E-05	BGL08_ORYSJ	2E-136	AT5G67030.1	ABA	7E-60
DN17828_c0_g1_i2	1.71	4.94	2E-05	9E-04	BGL14_ORYSJ	0E+00	AT5G67030.1	ABA	2E-103
DN16687_c0_g2_i10	0.81	7.46	3E-04	1E-02	BGL31_ORYSJ	0E+00	AT3G14440.1	ABA	3E-82
DN14155_c0_g1_i5	−8.68	−0.36	7E-08	7E-06	C16B1_PICSI	3E-106	AT3G14440.1	ABA	1E-73
DN14155_c0_g1_i4	1.51	4.41	1E-05	8E-04	C16B1_PICSI	1E-105	AT1G52400.1	ABA	1E-74
DN21426_c2_g1_i8	−1.30	8.20	2E-03	5E-02	CCD4_ARATH	1E-163	AT5G67030.1	ABA	1E-84
DN21426_c2_g1_i3	−2.68	5.02	4E-06	3E-04	CCD4_ARATH	1E-150	AT5G67030.1	ABA	1E-78
DN21164_c0_g1_i17	9.41	1.27	3E-05	1E-03	SFR2_ORYSJ	0E+00	AT5G67030.1	ABA	2E-21
DN16534_c1_g1_i1	−1.45	8.17	4E-04	1E-02	ZCD_CROSA	3E-109	AT1G52400.1	ABA	1E-51
DN21684_c0_g1_i6	−1.25	8.56	1E-06	1E-04	ZEP_ORYSJ	2E-137	AT1G52400.1	ABA	1E-86
DN21684_c0_g1_i3	11.47	3.29	2E-26	2E-23	ZEP_ORYSJ	3E-138	AT1G30100.1	ABA	2E-87
DN21684_c0_g1_i14	3.81	4.94	1E-20	8E-18	ZEP_ORYSJ	3E-77	AT2G27150.1	ABA	3E-46
DN21008_c0_g1_i9	−1.43	7.08	2E-08	2E-06	ZEP_ORYSJ	0E+00	AT1G52400.1	ABA	0E+00
DN21008_c0_g1_i12	8.47	4.96	5E-38	1E-34	ZEP_ORYSJ	0E+00	AT5G45340.1	ABA	2E-156
DN21008_c0_g1_i1	2.05	3.96	3E-04	1E-02	ZEP_ORYSJ	0E+00	AT5G45340.1	ABA	0E+00
DN21096_c0_g5_i3	9.98	1.82	9E-09	1E-06	ADO1_ORYSJ	0E+00	AT1G68050.1	Flowering	0E+00
DN21096_c0_g5_i1	3.94	2.46	6E-04	2E-02	ADO1_ORYSJ	0E+00	AT1G68050.1	Flowering	0E+00
DN20112_c0_g3_i4	9.70	1.55	7E-04	2E-02	ADO3_ORYSJ	0E+00	AT1G68050.1	Flowering	0E+00
DN17539_c1_g1_i9	9.27	1.14	1E-05	6E-04	AP2_ARATH	3E-85	LOC_Os05g03040.1	Flowering	1E-94
DN13126_c0_g1_i5	7.88	−0.17	1E-05	8E-04	ARP6_ORYSJ	2E-131	LOC_Os01g16414.1	Flowering	3E-125
DN18311_c0_g2_i10	8.52	0.43	8E-08	8E-06	ART1_ORYSJ	1E-127	LOC_Os01g09850.1	Flowering	4E-21
DN19270_c1_g1_i5	8.23	0.16	1E-07	1E-05	BH074_ARATH	2E-48	AT4G34530.1	Flowering	4E-41
DN19270_c1_g1_i2	−2.53	−0.18	1E-03	4E-02	BH074_ARATH	1E-48	AT4G34530.1	Flowering	2E-41
DN16755_c0_g1_i13	−0.87	5.64	1E-04	5E-03	CDF2_ARATH	9E-49	LOC_Os03g07360.1	Flowering	1E-163
DN18683_c1_g2_i3	−0.70	5.90	8E-04	3E-02	COL5_ARATH	7E-28	LOC_Os06g44450.1	Flowering	4E-51
DN19294_c1_g1_i15	3.76	1.45	3E-05	2E-03	CSK2A_MAIZE	3E-113	LOC_Os07g02350.1	Flowering	2E-114
DN20067_c1_g1_i18	−1.95	1.10	7E-04	3E-02	GIGAN_ORYSJ	0E+00	AT1G22770.1	Flowering	0E+00
DN21100_c0_g3_i2	−1.79	7.02	5E-05	2E-03	HD3A_ORYSJ	5E-103	LOC_Os06g06300.1	Flowering	8E-109
DN17448_c1_g2_i7	−1.71	2.89	2E-03	5E-02	MAD14_ORYSI	5E-31	LOC_Os03g54160.1	Flowering	2E-34
DN19322_c0_g3_i3	12.08	3.90	5E-10	8E-08	MAD14_ORYSJ	7E-80	LOC_Os03g54160.1	Flowering	1E-76
DN22596_c1_g1_i5	2.27	2.40	2E-04	7E-03	MSI1_ORYSJ	7E-154	LOC_Os03g43890.1	Flowering	5E-169
DN18513_c1_g1_i10	−2.43	0.04	4E-04	2E-02	NFYB3_ARATH	3E-61	LOC_Os07g41580.1	Flowering	1E-63
DN15466_c0_g5_i4	−2.55	3.89	7E-04	2E-02	NFYC4_ARATH	2E-57	LOC_Os03g14669.1	Flowering	5E-78
DN20769_c0_g2_i14	−0.84	4.23	2E-03	5E-02	ORR21_ORYSJ	3E-137	LOC_Os10g32600.1	Flowering	2E-29
DN21356_c1_g1_i7	−1.77	4.41	6E-04	2E-02	PHYA1_MAIZE	0E+00	AT1G09570.1	Flowering	0E+00
DN21356_c1_g1_i5	−0.80	5.28	1E-03	4E-02	PHYA1_MAIZE	0E+00	AT1G09570.1	Flowering	0E+00
DN21425_c0_g3_i3	2.34	2.62	1E-06	8E-05	PRL1_ARATH	0E+00	LOC_Os01g72220.1	Flowering	2E-22
DN16363_c0_g6_i2	−1.48	4.02	9E-05	4E-03	RVE2_ARATH	1E-36	LOC_Os08g06110.2	Flowering	2E-28
DN20573_c2_g1_i15	−9.09	0.01	7E-10	1E-07	ZRAB3_MOUSE	2E-105	AT3G12810.1	Flowering	2E-20
DN18931_c0_g1_i2	9.27	1.14	6E-12	1E-09	#N/A	#N/A	LOC_Os03g39129.1	Flowering	6E-79
DN18130_c0_g1_i17	−0.71	7.64	5E-04	2E-02	ALLN_ALLCE	6E-101	AT1G34040.1	Auxin	3E-111
DN19479_c1_g1_i4	−1.17	5.17	1E-03	4E-02	C7D55_HYOMU	5E-148	AT2G30770.1	Auxin	4E-95
DN18249_c2_g1_i5	9.42	1.29	2E-13	5E-11	C81E1_GLYEC	7E-74	AT2G30770.1	Auxin	1E-36
DN14609_c0_g1_i3	1.14	3.71	2E-03	5E-02	C81E1_GLYEC	8E-51	AT4G31500.1	Auxin	2E-36
DN18341_c0_g3_i7	1.86	3.52	2E-04	7E-03	C93A1_SOYBN	1E-141	AT4G31500.1	Auxin	6E-70
DN17059_c0_g1_i12	1.68	6.92	3E-04	1E-02	F3PH_ARATH	0E+00	AT2G30770.1	Auxin	2E-87
DN19892_c0_g4_i2	7.51	-0.50	7E-04	2E-02	OE64C_ARATH	3E-144	AT1G08980.1	Auxin	5E-97
DN19892_c0_g4_i1	12.41	4.23	2E-48	5E-45	OE64C_ARATH	0E+00	AT1G08980.1	Auxin	7E-96
DN16119_c1_g2_i5	1.21	5.46	2E-04	9E-03	TCMO_CATRO	0E+00	AT4G31500.1	Auxin	3E-47
DN16119_c1_g2_i10	1.35	5.33	2E-04	9E-03	TCMO_POPKI	4E-122	AT4G31500.1	Auxin	5E-36
DN20194_c0_g2_i1	−11.73	2.56	7E-25	5E-22	TRPA1_ARATH	3E-128	AT4G02610.1	Auxin	1E-140
DN12409_c0_g2_i1	2.37	2.89	8E-04	3E-02	**708A6_MAIZE**	3E-144	AT2G36800.1	Br	3E-39
DN16051_c0_g7_i2	−1.18	5.36	4E-05	2E-03	C70B2_ARATH	3E-161	AT2G26710.1	Br	1E-110
DN20659_c0_g1_i1	1.90	5.10	2E-07	2E-05	HMNGT_SORBI	3E-153	AT2G36800.1	Br	1E-34
DN18837_c0_g2_i8	2.39	4.16	4E-04	1E-02	U73C5_ARATH	8E-131	AT2G36800.1	Br	7E-136
DN16873_c0_g2_i12	13.25	5.07	8E-50	2E-46	U83A1_ARATH	4E-113	AT2G36800.1	Br	1E-49
DN15213_c1_g5_i1	−1.29	3.99	2E-05	1E-03	U83A1_ARATH	4E-113	AT2G36800.1	Br	2E-48
DN16873_c0_g2_i15	8.93	0.82	7E-08	7E-06	U83A1_ARATH	1E-64	AT2G36800.1	Br	7E-37
DN16873_c0_g2_i14	−2.73	3.02	9E-12	2E-09	U83A1_ARATH	4E-112	AT2G36800.1	Br	5E-49
DN16873_c0_g2_i11	10.97	2.80	2E-17	7E-15	U83A1_ARATH	2E-112	AT2G36800.1	Br	3E-49
DN19680_c0_g6_i1	2.01	3.89	7E-04	2E-02	UGT1_GARJA	4E-81	AT2G36800.1	Br	1E-21
DN15090_c2_g7_i1	1.25	5.29	5E-04	2E-02	UGT1_GARJA	5E-88	AT2G36800.1	Br	5E-26
DN16302_c0_g5_i1	3.94	0.77	2E-05	1E-03	UGT2_GARJA	5E-167	AT2G36800.1	Br	3E-37
DN9072_c0_g1_i3	2.48	5.04	5E-06	3E-04	**URT1_FRAAN**	5E-72	AT2G36800.1	Br	4E-32
DN9072_c0_g1_i2	9.00	0.88	2E-08	2E-06	**URT1_FRAAN**	1E-45	AT2G36800.1	Br	1E-22
DN21803_c0_g1_i4	−2.62	4.08	2E-06	2E-04	C14B3_MAIZE	0E+00	AT5G38450.1	Cytokinin	4E-60
DN20210_c2_g2_i11	−2.04	2.05	3E-05	2E-03	CKX11_ORYSJ	0E+00	AT5G21482.1	Cytokinin	2E-172
DN18565_c0_g1_i18	10.51	2.34	2E-05	1E-03	CKX4_ORYSJ	0E+00	AT2G41510.1	Cytokinin	0E+00
DN18596_c1_g2_i4	−0.94	5.47	9E-04	3E-02	LOG_ORYSJ	5E-130	AT2G28305.1	Cytokinin	7E-85
DN18596_c1_g2_i12	−7.92	−1.02	5E-06	3E-04	LOG_ORYSJ	3E-30	AT2G28305.1	Cytokinin	6E-25
DN15199_c2_g1_i3	1.33	3.83	1E-04	4E-03	LOGL9_ORYSJ	2E-86	AT2G28305.1	Cytokinin	2E-77
DN20315_c0_g6_i1	−0.88	5.06	9E-05	4E-03	ACCO1_ORYSJ	3E-97	AT1G62380.1	Ethylene	8E-78
DN17486_c1_g5_i9	1.96	3.11	1E-03	3E-02	SRG1_ARATH	8E-51	AT1G62380.1	Ethylene	6E-29
DN17486_c1_g5_i1	−1.25	6.09	1E-04	5E-03	SRG1_ARATH	8E-69	AT1G62380.1	Ethylene	4E-39
DN22035_c0_g2_i3	−1.72	3.73	9E-05	4E-03	AAMT2_MAIZE	0E+00	AT5G56300.1	Gibberellin	2E-36
DN18070_c1_g1_i23	1.16	6.56	1E-05	7E-04	ACSS_MAIZE	0E+00	AT1G79460.1	Gibberellin	9E-101
DN10655_c0_g1_i4	1.56	3.70	2E-05	1E-03	DLO2_ARATH	1E-51	AT4G25420.1	Gibberellin	7E-53
DN12195_c0_g1_i1	7.67	−0.36	2E-05	9E-04	FLS_PETHY	7E-128	AT1G80340.1	Gibberellin	1E-45
DN15982_c0_g4_i5	7.63	-0.40	1E-03	4E-02	G2OX1_ARATH	2E-61	AT1G78440.1	Gibberellin	2E-69
DN17015_c0_g3_i6	1.78	3.27	5E-04	2E-02	G2OX8_ARATH	1E-81	AT4G21200.1	Gibberellin	5E-82
DN16942_c0_g3_i6	13.22	5.04	1E-13	4E-11	IDS3_HORVU	4E-179	AT4G21200.1	Gibberellin	5E-41
DN14718_c0_g1_i7	−1.53	1.87	6E-04	2E-02	NCS1_COPJA	1E-97	AT1G15550.1	Gibberellin	2E-43
DN18924_c0_g1_i2	1.80	4.04	1E-04	5E-03	C86B1_ARATH	2E-133	AT5G63450.1	Jasmonate	1E-93
DN18607_c1_g2_i1	−0.94	5.25	4E-04	1E-02	BLH4_ARATH	2E-123	AT5G41410.1	MADS-box	2E-69
DN18420_c0_g5_i1	2.99	1.05	1E-03	3E-02	BLH9_ARATH	3E-69	AT5G41410.1	MADS-box	4E-46
DN18522_c2_g2_i2	9.19	1.05	1E-03	4E-02	GSO1_ARATH	9E-44	AT3G12145.1	MADS-box	7E-21
DN21745_c1_g1_i6	−10.38	1.24	1E-17	5E-15	CDPK2_ORYSJ	0E+00	AT3G13530.1	MAPK	3E-29
DN22003_c1_g7_i3	12.88	4.69	4E-68	5E-64	CIPK7_ORYSJ	0E+00	AT4G08500.1	MAPK	7E-31
DN22003_c1_g7_i1	−1.25	5.57	7E-06	4E-04	CIPK7_ORYSJ	0E+00	AT4G08500.1	MAPK	7E-31
DN22145_c3_g1_i11	−0.95	4.99	4E-04	2E-02	CIPKL_ORYSJ	0E+00	AT5G66850.1	MAPK	2E-32
DN21072_c2_g1_i9	−0.95	6.49	4E-04	2E-02	DUS1_ARATH	2E-32	AT3G06110.2	MAPK	5E-30
DN21072_c2_g1_i4	−1.68	4.53	4E-08	5E-06	DUS1_ARATH	7E-33	AT3G06110.2	MAPK	1E-30
DN21072_c2_g1_i13	8.58	4.53	4E-55	2E-51	DUS1_ARATH	4E-56	AT3G06110.2	MAPK	6E-54
DN21072_c2_g1_i11	9.02	0.89	3E-09	4E-07	DUS1_ARATH	7E-30	AT3G06110.2	MAPK	4E-28
DN19964_c3_g1_i11	7.97	−0.09	8E-04	3E-02	EDR1_ARATH	2E-73	AT1G73660.1	MAPK	2E-80
DN19964_c3_g1_i10	7.96	−0.09	7E-04	3E-02	EDR1_ARATH	2E-73	AT1G73660.1	MAPK	2E-80
DN17893_c0_g1_i2	−5.63	−0.17	1E-03	4E-02	LRK41_ARATH	0E+00	AT2G17090.1	MAPK	4E-23
DN21224_c0_g1_i14	10.48	2.32	2E-20	1E-17	M3K1_ARATH	6E-110	AT4G08500.1	MAPK	5E-112
DN18222_c0_g1_i2	9.96	1.81	8E-08	8E-06	MPK12_ORYSJ	0E+00	AT3G18040.1	MAPK	0E+00
DN20397_c2_g6_i3	−1.23	2.93	1E-03	4E-02	MPK17_ORYSJ	0E+00	AT3G18040.1	MAPK	0E+00
DN16562_c0_g4_i9	9.88	1.73	5E-07	4E-05	P2C34_ORYSJ	0E+00	AT2G40180.1	MAPK	2E-22
DN22553_c3_g2_i7	8.91	0.80	1E-09	1E-07	P2C58_ORYSJ	0E+00	AT2G30020.1	MAPK	3E-28
DN21118_c0_g3_i2	−6.64	1.57	9E-04	3E-02	P2C62_ORYSJ	1E-117	AT2G30020.1	MAPK	1E-32
DN21118_c0_g3_i1	1.18	3.20	1E-03	3E-02	P2C62_ORYSJ	1E-157	AT2G40180.1	MAPK	1E-30
DN19714_c0_g1_i8	9.68	1.53	3E-07	3E-05	PBS1_ARATH	0E+00	AT2G26330.1	MAPK	1E-53
DN20830_c0_g1_i7	7.08	2.13	1E-12	3E-10	PERK4_ARATH	7E-113	AT2G26330.1	MAPK	6E-49
DN20830_c0_g1_i5	11.65	3.47	1E-25	1E-22	PERK4_ARATH	1E-120	AT2G26330.1	MAPK	1E-55
DN20830_c0_g1_i4	−2.20	4.43	1E-14	4E-12	PERK4_ARATH	4E-120	AT2G26330.1	MAPK	5E-55
DN20830_c0_g1_i20	8.06	3.10	5E-21	3E-18	PERK4_ARATH	4E-120	AT2G26330.1	MAPK	5E-55
DN20830_c0_g1_i15	8.70	0.59	3E-07	2E-05	PERK4_ARATH	2E-112	AT2G26330.1	MAPK	6E-47
DN17348_c0_g3_i3	−1.01	8.02	2E-03	5E-02	PHT1A_ORYSJ	0E+00	AT3G06030.1	MAPK	2E-23
DN19916_c0_g3_i1	1.83	3.90	1E-04	6E-03	PPCK2_ARATH	6E-63	AT3G06030.1	MAPK	3E-28
DN19658_c0_g1_i8	11.27	3.10	5E-10	8E-08	RPK2_ARATH	0E+00	AT2G26330.1	MAPK	2E-56
DN19622_c0_g4_i1	8.84	0.72	1E-03	3E-02	SAPK8_ORYSJ	0E+00	AT1G54960.1	MAPK	2E-20
DN19675_c1_g1_i8	−1.95	0.97	2E-04	9E-03	TMK1_ARATH	0E+00	AT2G17090.1	MAPK	5E-25
DN21785_c0_g2_i2	10.25	2.09	3E-11	6E-09	WAK5_ARATH	2E-132	AT2G26330.1	MAPK	2E-47
DN21785_c0_g2_i10	10.20	2.04	4E-06	3E-04	WAK5_ARATH	3E-129	AT2G26330.1	MAPK	6E-48
DN21388_c0_g1_i1	−10.54	1.40	2E-19	1E-16	WNK1_ORYSJ	0E+00	AT4G08500.1	MAPK	6E-27
DN15449_c0_g2_i3	−8.69	−0.34	1E-07	1E-05	WNK4_ARATH	9E-119	AT4G08500.1	MAPK	1E-31
DN22069_c2_g1_i1	1.59	3.66	2E-04	7E-03	WRK19_ARATH	9E-58	AT4G12020.2	MAPK	8E-58
DN20705_c0_g2_i2	4.79	1.30	7E-07	5E-05	WRK19_ARATH	7E-59	AT4G12020.2	MAPK	6E-59
DN18165_c3_g1_i8	−9.46	0.36	3E-10	5E-08	WRK46_HORVU	0E+00	AT4G12020.2	MAPK	2E-33
DN17290_c0_g5_i3	2.11	3.38	1E-06	1E-04	Y1154_ARATH	0E+00	AT2G26330.1	MAPK	5E-57
DN17290_c0_g5_i1	1.29	4.10	1E-03	4E-02	Y1154_ARATH	0E+00	AT2G26330.1	MAPK	1E-58
DN20698_c0_g1_i1	−10.87	1.71	2E-22	2E-19	Y1461_ARATH	1E-177	AT2G43790.1	MAPK	2E-46
DN15473_c1_g2_i6	9.37	1.23	9E-04	3E-02	Y3475_ARATH	9E-168	AT2G26330.1	MAPK	2E-84
DN15473_c1_g2_i5	8.39	4.22	2E-04	1E-02	Y3475_ARATH	9E-151	AT2G26330.1	MAPK	5E-96
DN19915_c0_g4_i2	7.56	−0.45	1E-04	6E-03	YODA_ARATH	2E-43	AT5G66850.1	MAPK	1E-69
DN15465_c2_g1_i7	9.62	1.47	3E-14	1E-11	YODA_ARATH	1E-61	AT1G53570.1	MAPK	1E-68
DN15465_c2_g1_i4	8.89	0.78	2E-05	1E-03	YODA_ARATH	1E-61	AT1G53570.1	MAPK	1E-68
DN15205_c0_g7_i2	8.26	0.18	4E-08	4E-06	CSCLC_ARATH	3E-171	AT1G69450.1	Sucrose	3E-149
DN16957_c0_g2_i1	6.59	5.11	8E-04	3E-02	CSCLD_ARATH	0E+00	AT1G30360.1	Sucrose	0E+00
DN21735_c0_g1_i4	14.72	6.54	2E-65	2E-61	GSTU1_ORYSI	3E-82	AT1G10370.1	Sucrose	2E-44
DN21089_c0_g6_i2	9.83	1.68	1E-03	4E-02	GSTU1_ORYSJ	7E-54	AT1G10370.1	Sucrose	3E-28
DN19270_c1_g2_i3	1.07	4.24	8E-04	3E-02	GSTUH_ARATH	3E-56	AT1G10370.1	Sucrose	1E-64
DN19617_c2_g5_i1	7.36	−0.64	6E-04	2E-02	GSTX2_MAIZE	4E-146	AT1G10370.1	Sucrose	2E-49
DN21326_c0_g1_i9	1.04	5.67	3E-04	1E-02	HSP70_MAIZE	2E-142	AT1G56410.1	Sucrose	5E-106
DN20957_c0_g5_i1	9.12	0.99	2E-04	7E-03	HSP72_SOLLC	3E-110	AT1G56410.1	Sucrose	2E-80
DN20957_c0_g1_i3	1.73	4.64	7E-06	4E-04	HSP7C_PETHY	0E+00	AT1G56410.1	Sucrose	0E+00
DN22058_c1_g1_i7	10.10	7.86	1E-18	5E-16	HSP7S_PEA	0E+00	AT1G56410.1	Sucrose	9E-157
DN22058_c1_g1_i1	−0.91	8.98	4E-04	2E-02	HSP7S_PEA	0E+00	AT1G56410.1	Sucrose	4E-157
DN22524_c0_g1_i7	3.17	−0.09	5E-04	2E-02	HSP83_ORYSJ	2E-121	AT5G56030.2	Sucrose	4E-103
DN16629_c1_g1_i4	−1.01	5.97	1E-05	6E-04	PFPA_RICCO	2E-118	AT1G76550.1	Sucrose	2E-111
DN16629_c1_g1_i11	-0.82	4.90	1E-03	4E-02	PFPA_RICCO	0E+00	AT1G76550.1	Sucrose	0E+00
DN15779_c0_g1_i5	−1.19	2.94	2E-03	5E-02	PMTH_ARATH	0E+00	AT4G19120.1	Sucrose	5E-122
DN22018_c0_g1_i9	-1.12	3.46	5E-04	2E-02	SPSA3_ORYSJ	0E+00	AT1G04920.1	Sucrose	1E-180
DN15021_c0_g2_i8	−3.20	1.38	2E-06	1E-04	SWET4_ORYSJ	2E-136	AT2G39060.1	Sucrose	8E-39
DN21265_c0_g1_i7	5.50	1.06	4E-04	1E-02	SWT13_ORYSJ	2E-56	AT2G39060.1	Sucrose	5E-26
DN20994_c0_g4_i1	8.04	-0.02	2E-05	1E-03	TPS10_ARATH	2E-148	AT1G60140.1	Sucrose	3E-148
DN22618_c1_g3_i1	−1.08	5.50	1E-04	5E-03	TPS7_ARATH	0E+00	AT1G06410.1	Sucrose	0E+00

*Log_2_FC: Log_2_(Fold change) = Log_2_(TR/NT). CPM: count per million read. FDR: false discovery rate. The bold DETs were verified by RT-qPCR. DN19322_c0_g3_i3 is the best hit of the *VcSOC1K*. Br, Brassinosteroid; ABA, Abscisic acid.*

### Gene Networks of the DETs

**FIGURE 3:**
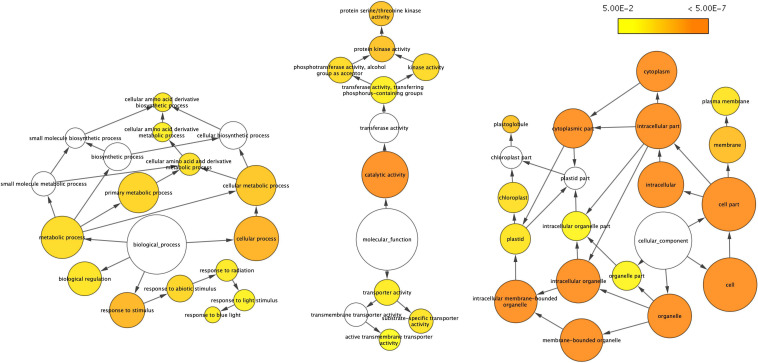
Gene networks of differentially expressed transcripts (DETs) identified from the leaves of the 83-day old plants. The ontology file of GOSlim_Plants in BiNGO was used to identify overrepresented GO terms (*p* < 0.05). Bubble size and color indicate the frequency of the GO term and the *P*-value, respectively.

As a quantitative trait, since there are no convincing molecular criteria available to define different crop yield potentials, we visualized the overall impact of VcSOC1K-CX by further analyses of the DETs using the ontology file of GOSlim_Plants in BiNGO to identify overrepresented GO terms. Thirty-nine overrepresented GO terms were revealed in the gene networks, including 13 in “biological process,” nine in “molecular function,” and 17 in “cellular component” (Figure 3). These overrepresented GO terms indicated a broad impact of the VcSOC1K-CX at different levels, which provided alternative evidence to support that the VcSOC1K-CX worked effectively in regulating plant development.

Of the 13 overrepresented GO terms “biological process,” five were related to abiotic factors (Figure 3). In this study, the field-grown BC_2_ plants were likely exposed to more abiotic stresses than the pot-grown BC_1_ plants tested under more controlled conditions (i.e., water and fertilizing). The higher grain yield increase by 100% for the BC_2_ TR (vs BC_2_ NT) plants (100%), compared to the grain yield increase by 13 to 27% observed in four experiments for BC_1_ TR (vs BC_1_ NT) plants, could be attributed to enhanced abiotic tolerance in transgenic plants that could be more obvious under stress conditions. In the previous report, VcSOC1K-OX resulted in high pH tolerance in blueberry plants (Song and Chen, 2018).

## Conclusion

K-domain technology utilizes expression of the VcSOC1K to regulate plant growth. In maize, the VcSOC1K showed similarities to five MADS-box genes. VcSOC1K-CX resulted in grain yield increase by 13 to 100% in all five experiments conducted under different experimental conditions. Transcriptome comparisons revealed 982 DEGs in the leaves from the growth stage R2 plants, supporting that the K-domain technology were multiple functional. The K-domain technology opens a new approach to increase crop yield by its potential of mimicking the K-domains of multiple MADS-box genes.

## Data Availability Statement

The original contributions presented in the study are publicly available. This data can be found here: NCBI repository, accession number PRJNA701291.

## Author Contributions

G-qS conceived, supervised the study, analyzed data, and wrote the manuscript. XH and G-qS conducted the experiments. Both authors read and approved the final manuscript.

## Conflict of Interest

The authors declare that the research was conducted in the absence of any commercial or financial relationships that could be construed as a potential conflict of interest.

## Footnotes

1 https://www.ebi.ac.uk/Tools/msa/clustalo/

2 https://www.maizegdb.org/assembly

3 www.bioinformatics.babraham.ac.uk/projects/fastqc/

4 http://hormones.psc.riken.jp/

## Supplementary Material

Supplementary Figure 1 80-day old BC_2_ plants growing for phenotyping in the field test in 2020 (20exp5).

Supplementary Figure 2 Comparison of the RT-qPCR analysis result and the RNA-seq data of the selected DETs (Table 1 and Supplementary Table 4). −ΔΔCt is an average of three biological and three technical replicates for each DET. ZmActin1 (SAC1_ARATH) was used to normalize the RT-qPCR results. Bars indicate standard deviation.

Supplementary Table 1 PCR primers.

Supplementary Table 2 Summary of phenotype assessment of BC_1_ plants.

Supplementary Table 3 Effect of VcSOC1K-CX on seed quality of field grown plants. The mean values (±STDEV) were for 3 transgenic, 5 non-transgenic, and 15 wild type B73 plants.

Supplementary Table 4 Differentially expressed transcripts (DETs) in maize new leaves from 83-day old plants. Log_2_FC: Log_2_(Fold change) = Log_2_(TR/NT). CPM, count per million read; FDR, false discovery rate; #N/A, no annotation.
