# Supplementary material for: K-Domain Technology: Constitutive Expression of a Blueberry Keratin-Like Domain Mimics Expression of Multiple MADS-Box Genes in Enhancing Maize Grain Yield
Source: Front Plant Sci. 2021 May 7;12:664983. doi: 10.3389/fpls.2021.664983 (PMC8137907; doi:10.3389/fpls.2021.664983)
Supplement: Supplementary Figure 1 — 80-day old BC2 plants growing for phenotyping in the field test in 2020 (20exp5). [file Data_Sheet_1.docx]

**K-domain technology: Constitutive Expression of a Blueberry Keratin-like Domain Mimics Expression of Multiple MADS-box Genes in Enhancing Maize Grain Yield**

Guo-qing Song* and Xue Han


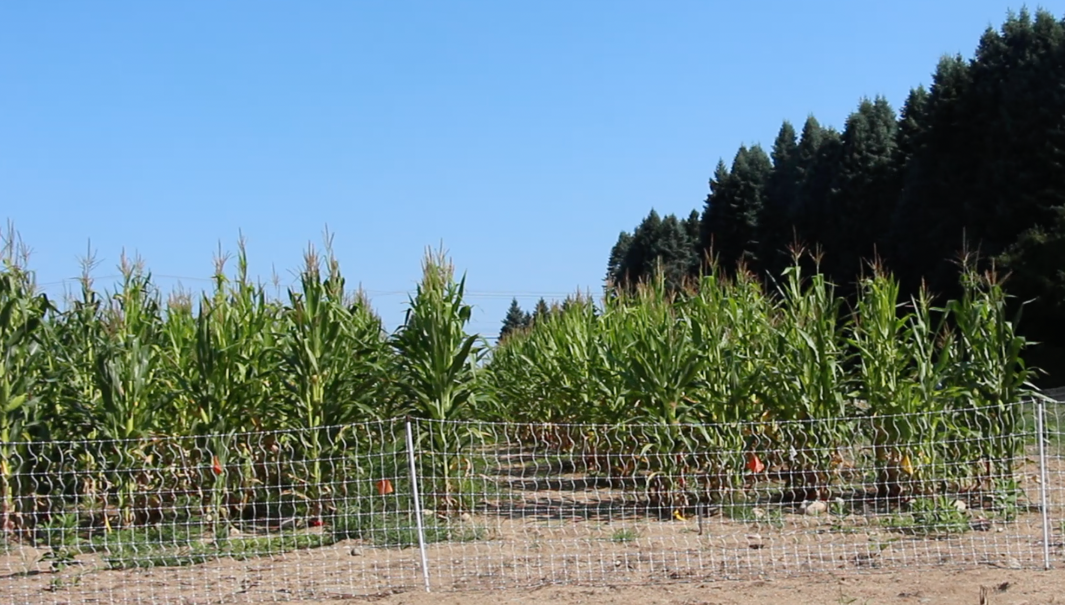


**FIGURE S1 |** 80-day old BC_2_ plants growing for phenotyping in the field test in 2020 (20exp5)

**FIGURE S2 |** Comparison of the RT-qPCR analysis result and the RNA-seq data of the selected DETs (Table 1, Table S4). -∆∆Ct is an average of three biological and three technical replicates for each DET. ZmActin1 (SAC1_ARATH) was used to normalize the RT-qPCR results. Bars indicate standard deviation.

**TABLE S1 |** PCR primers

| Gene | Primer name | Primer sequence (5’ to 3’) | Transcript ID |
| --- | --- | --- | --- |
| **PCR or RT-PCR** |  |  |  |
| ZmSOC1 | MK_F | ATGGTGCGGGGCAAGACGCAG |  |
|  | MK_R | GCCTGACCTGACCGCCACTGC |  |
| ZmSOC1 | MK_F1 | GGGGTACCATGGTGCGGGGCAAGACGCAG |  |
|  | MK_R1 | GCTCTAGAGCCTGACCTGACCGCCACTGC |  |
|  | 35S_F | TGA CGC ACA ATC CCA CTA TC |  |
| bar | BAR_F | ATG AGC CCA GAA CGA CGC C |  |
|  | BAR_R | TCA GAT CTC GGT GAC GG |  |
| ZmAct | ZmAct_F | AATGCTGGGGAAGACAGCTC |  |
|  | ZmAct_R | ACCAGGCTGCATAACTGCAT |  |
| **RT-qPCR** |  |  |  |
| 708A6_MAIZE | 708A6_F | CGA CAA GGT GAA GGA GGT TAT G | DN12409_c0_g2_i1 |
|  | 708A6_R | CCC TTA AGC TTG GCG ATG AA |  |
| AB11G_ARATH | AB11G_F | TCC GAT CTC AGA GCT ACC C | DN21605_c0_g1_i4 |
|  | AB11G_R | AAG GAC TGC GTC AGG AAA C |  |
| CHS2_MAIZE | CHS2_F | GGA GAA CCC GAG CAT GTG | DN18418_c0_g1_i17 |
|  | CHS2_R | GCG TGA TCC GCG ACT TT |  |
| CYPB_VICFA | CYPB_F | CCG ATC TCG CTA GTT CTC AAA | DN16412_c0_g5_i1 |
|  | CYPB_R | TTA GCA CAC CAG GGC TAA C |  |
| PMA2_SOLLC | PMA2_F | GTC GTC TCT TCA TCT CAG CAC | DN22665_c1_g1_i5 |
|  | PMA2_R | ATT GTA ACG CGA GCT TGT AGA |  |
| RHM1_ARATH | RHM1_F | CTA CAA CAT CGG CAC CAA GA | DN36404_c0_g1_i1 |
|  | RHM1_R | GAA GTA CCT CTG GTC GTT GAA G |  |
| SAC1_ARATH | zmActin1_F | TCA AGC AGA AGA CGG CAT AC | DN18124_c2_g1_i7 |
|  | zmActin1_R | GAG AGG AAA GGC GGA GAA AG |  |
| URT1_FRAAN | URT1_F | CCG CTC CAC ATC GTC ATT T | DN9072_c0_g1_i3 |
|  | URT1_R | TGG GTG GAG ACG AAA GAC A |  |

**TABLE S2 |** Summary of phenotype assessment of BC_1_ plants

|  | 18exp1 | |  | | | 18exp2 | | | | | |  | | | | 18exp3 | | | | | | | |  | | | 19exp4 | | | | | | | | |  |  |
| --- | --- | --- | --- | --- | --- | --- | --- | --- | --- | --- | --- | --- | --- | --- | --- | --- | --- | --- | --- | --- | --- | --- | --- | --- | --- | --- | --- | --- | --- | --- | --- | --- | --- | --- | --- | --- | --- |
| Planting time | 2018/5/17 | |  | | | 2018/6/11 | | | | | |  | | | | 2018/6/25 | | | | | | | |  | | | 2019/5/11 | | | | | | | | |  |  |
| Genotype | NT | TR | |  | | | NT | | TR | | | |  | | | | NT | | | | TR | | | |  | | | | NT | | TR | | | |  |  |  |
| Transgenic lines |  | 1,2,3,18 | |  | | |  | | 1,2,3,15,18 | | | |  | | | |  | | | | 1,2,3,7,9,18 | | | |  | | | |  | | 1,2,3,4,9,15,16,18 | | | |  |  |  |
| Germination time (days) | 6.7 ± 4.2 | 5.2 ± 3.1 | |  | | | 4.2 ± 0.5 | | 4 ± 0.4 | | | |  | | | | 4.4 ± 1.1 | | | | 4.3 ± 0.8 | | | |  | | | | NA | | NA | | | |  |  |  |
| Time of tassel appearance | 54.6 ± 4.5 | 53.9 ± 1.6 | |  | | | 52.2 ± 1.4 | | 52.1 ± 1.6 | | | |  | | | | 49.1 ± 3 | | | | 48.5 ± 1.9 | | | |  | | | | NA | | NA | | | |  |  |  |
| Time of silk appearance (days) | 62.4 ± 4 | 61.5 ± 2.4 | |  | | | 58.6 ± 2.2 | | 58.2 ± 2.3 | | | |  | | | | 55.5 ± 3.4 | | | | 56.1 ± 2.1 | | | |  | | | | NA | | NA | | | |  |  |  |
| Flower at Branch # | 5.9 ± 0.4 | 6.1 ± 1 | |  | | | 6.6 ± 0.7 | | 6.9 ± 0.7 | | | |  | | | | 6.7 ± 0.7 | | | | 6.9 ± 0.6 | | | |  | | | | NA | | NA | | | |  |  |  |
| Height soil to 1^st^ corn | 53.9 ± 6.6 | 60 ± 10.4 | |  | | | 66.3 ± 10.5 | | 65.3 ± 11.9 | | | |  | | | | 72.7 ± 9.5 | | | | 75.6 ± 12.7 | | | |  | | | | NA | | NA | | | |  |  |  |
| Leaf number | 11 ± 0.8 | 11.7 ± 1.1 | |  | | | 12.2 ± 0.9 | | 12.1 ± 1 | | | |  | | | | 12 ± 1.3 | | | | 12.5 ± 0.7 | | | |  | | | | NA | | NA | | | |  |  |  |
| Height of plant (cm) | 164.7 ± 12.6 | 166.6 ± 14.1 | | | | 184.5 ± 9.2 | | | | | 188.4 ± 12.6 | | | | | | | | 194 ± 23.2 | | | 202.9 ± 18.9 | | | | | | NA | | | | | NA | | | |  |
| No. of viable Corn | 2 ± 0.8 | 2 ± 0.7 | | |  | | | 1.1 ± 0.3 | | 1.2 ± 0.4 | | | | |  | | | | | 1.7 ± 0.6 | | | 1.9 ± 0.5 | | |  | | | | NA | | | | NA | | | |
| Dry weight of plant aerial (excluding ear) | 168.1 ± 43.7 | 152.5 ± 37.1 | | | | NA | | | | | NA | | |  | | | | 139.4 ± 14 | | | | 149.4 ± 18.6 | | | | | | 131.9 ± 43.8 | | | | 138.6 ± 41.7 | | | | |  |
| Ear dry weight (g) | 227.9 ± 54.3 | 299.8 ± 69.4 | | | | 207.2 ± 32.1 | | | | | 234.2 ± 35.1 | | | | | | | | 198.3 ± 58.4 | | | 230.9 ± 39.9 | | | | | | 40.4 ± 41.1 | | | | | 44.7 ± 40.2 | | | |  |
| Grain dry weight (g) | 190.3 ± 88.3 | 242.2 ± 67.2 | | | | 186.1 ± 29.5 | | | | | 211 ± 33.9 | | |  | | | | 178.1 ± 54.5 | | | | 208.3 ± 36.4 | | | | | | 28.2 ± 36.6 | | | | 32.5 ± 37.3 | | | | |  |

**TABLE S3 |** Effect of VcSOC1K-CX on seed quaility of field grown plants. The mean values (± STDEV) were for 3 transgenic, 5 nontransgenic, and 15 wild type B73 plants.

| Genotype | NT | TR | WT |
| --- | --- | --- | --- |
| DM | 69.9±1.64 | 72.33±3.38 | 70.26±3.88 |
| ADF | 40.28±3.33 | 38.52±4.44 | 40.18±6.78 |
| Ca | 0.74±0.14 | 0.74±0.14 | 0.79±0.1 |
| P | 0.37±0.05 | 0.39±0.06 | 0.34±0.05 |
| K | 5.91±0.33 | 5.73±0.69 | 5.86±0.7 |
| Mg | 0.05±0.06 | 0.07±0.05 | 0.03±0.03 |
| IVTDMD24 | 100.78±0.9 | 100.71±2.57 | 102.25±3.91 |
| NDFD24 | 57.38±2.22 | 57.47±2.22 | 60.13±4.67 |
| IVTDMD30 | 94.38±0.84 | 94.47±1.83 | 95.44±2.93 |
| NDFD30 | 44.78±1.91 | 44.48±1.06 | 44.42±1.74 |
| IVTDMD48 | 82±4.6 | 84.64±5.68 | 80.12±6.56 |
| NDFD48 | 50.62±1.91 | 48.37±2.06 | 47.45±4.23 |
| ASH | 0±0 | 0.02±0.04 | 0.08±0.23 |
| FAT | 3.46±0.82 | 3.6±0.82 | 3.27±0.76 |
| Lignin | 0±0 | 0.02±0.05 | 0.16±0.26 |
| Starch | 73.36±0.35 | 72.57±3.15 | 75.04±3.98 |
| ADP | 3.57±0.24 | 3.32±0.66 | 3.86±0.76 |
| NDF | 53.38±3.54 | 50.14±4.98 | 52.75±10.76 |
| PROTEIN | 7.79±0.39 | 7.91±1.46 | 7.86±2.34 |
| ACETIC | 2.93±0.22 | 2.75±0.39 | 3.04±0.3 |
| CL | -0.1±0.14 | -0.02±0.11 | -0.02±0.33 |
| CU | 2.83±0.11 | 2.95±0.25 | 2.78±0.22 |
| FE | 31.69±73.76 | 26.02±74.65 | -62.12±86.25 |
| LACTIC | 1.25±0.9 | 1.14±0.3 | 0.64±0.3 |
| MN | -4.18±1.62 | -2.12±3.15 | -4.26±3.01 |
| MOISTURE | 30.1±1.64 | 27.67±3.38 | 29.74±3.88 |
| NA | -0.06±0.01 | -0.05±0.01 | -0.05±0.01 |
| NDFCP | -0.59±0.12 | -0.51±0.24 | -0.72±0.25 |
| NFC. | 49.61±1.55 | 49.5±1.03 | 48.88±2.95 |
| NH4 | -0.44±0.02 | -0.42±0.03 | -0.41±0.08 |
| S | 0.1±0.01 | 0.1±0.01 | 0.1±0.01 |
| SOL_CARBOS | -141.09±18.11 | -125.2±29.51 | -143.06±36.06 |
| SOL_PROTEIN | 38.85±0.59 | 38.17±0.57 | 38.56±0.84 |
| SIMPLE_SUGARS | 2.52±0.57 | 2.17±0.58 | 2.11±0.72 |
| UIP | 34.71±0.53 | 34.72±0.44 | 35.22±0.59 |
| ZN | 20.64±0.04 | 20.69±0.1 | 20.66±0.14 |

| **TABLE S4 \|** Differentially expressed transcripts (DETs) in maize new leaves from 83-day old plants. Log_2_FC: Log_2_(Fold change) = Log_2_(TR/NT). CPM: count per million read. FDR: false discovery rate. The bold DETs were verified by RT-qPCR. #N/A: No annotation | | | | | | |
| --- | --- | --- | --- | --- | --- | --- |
|  | Log_2_FC | Log_2_CPM | P_Value | FDR | Annotation | Annotation_e_value |
| DN15963_c2_g1_i3 | -15.13 | 5.94 | 2.4E-138 | 1.5E-133 | SYTM2_ARATH | 0 |
| DN16870_c0_g2_i7 | -15.23 | 6.04 | 2.4E-136 | 7.5E-132 | BAN_ARATH | 7.97E-35 |
| DN15967_c0_g2_i1 | -13.61 | 4.43 | 3.7E-82 | 7.9E-78 | #N/A | #N/A |
| DN17931_c0_g3_i1 | 13.00 | 4.81 | 1.7E-70 | 2.7E-66 | COX6A_ARATH | 1.43E-31 |
| DN22003_c1_g7_i3 | 12.88 | 4.69 | 3.7E-68 | 4.6E-64 | CIPK7_ORYSJ | 0 |
| DN15381_c1_g3_i10 | 13.25 | 5.06 | 3.7E-67 | 3.9E-63 | CBSX3_ARATH | 4.67E-97 |
| DN21106_c0_g5_i5 | 13.10 | 4.92 | 2.2E-66 | 2.0E-62 | IF1A_WHEAT | 4.81E-65 |
| DN21735_c0_g1_i4 | 14.72 | 6.54 | 2.4E-65 | 1.9E-61 | GSTU1_ORYSI | 3.44E-82 |
| DN14175_c0_g1_i3 | 10.56 | 6.13 | 3.5E-64 | 2.5E-60 | #N/A | #N/A |
| DN16210_c0_g4_i12 | -10.79 | 3.92 | 4.1E-59 | 2.6E-55 | #N/A | #N/A |
| DN16783_c3_g2_i9 | -12.89 | 3.71 | 7.9E-59 | 4.5E-55 | #N/A | #N/A |
| DN17530_c0_g2_i1 | -12.86 | 3.68 | 6.0E-58 | 3.2E-54 | RS33_ARATH | 1.31E-139 |
| DN21072_c2_g1_i13 | 8.58 | 4.53 | 3.9E-55 | 1.9E-51 | DUS1_ARATH | 3.56E-56 |
| DN15825_c1_g2_i8 | 9.73 | 4.77 | 5.0E-55 | 2.3E-51 | TRPC_ARATH | 2.46E-161 |
| DN19095_c0_g1_i4 | -8.00 | 3.95 | 6.6E-54 | 2.8E-50 | T184C_XENLA | 2.27E-34 |
| DN19676_c0_g2_i1 | 12.31 | 4.13 | 2.3E-52 | 9.0E-49 | 2A5N_ARATH | 0 |
| DN15825_c1_g2_i7 | 12.90 | 4.72 | 1.3E-51 | 4.9E-48 | TRPC_ARATH | 8.43E-163 |
| DN17710_c0_g1_i10 | -8.91 | 3.61 | 2.8E-51 | 1.0E-47 | YZR3_ARATH | 2.6E-20 |
| DN22194_c1_g3_i1 | 12.39 | 4.21 | 4.8E-51 | 1.6E-47 | CNGC2_ARATH | 0 |
| DN16431_c0_g1_i26 | 12.87 | 4.68 | 5.8E-51 | 1.8E-47 | PGP1B_ARATH | 5.11E-163 |
| DN16873_c0_g2_i12 | 13.25 | 5.07 | 8.1E-50 | 2.4E-46 | U83A1_ARATH | 3.96E-113 |
| DN21210_c0_g1_i12 | -12.56 | 3.38 | 1.2E-49 | 3.4E-46 | IFRD1_MOUSE | 4.53E-28 |
| DN17392_c0_g2_i4 | 12.64 | 4.45 | 1.8E-49 | 5.0E-46 | AGUA_ARATH | 3.39E-176 |
| DN19892_c0_g4_i1 | 12.41 | 4.23 | 2.0E-48 | 5.3E-45 | OE64C_ARATH | 0 |
| DN15342_c0_g1_i3 | 12.44 | 4.26 | 1.2E-46 | 3.0E-43 | QCR7_SOLTU | 2.93E-49 |
| DN17752_c0_g1_i3 | -12.35 | 3.17 | 1.3E-45 | 3.1E-42 | LTD_ORYSJ | 2.19E-82 |
| DN19979_c0_g1_i10 | 12.10 | 3.92 | 3.3E-45 | 7.8E-42 | UBC2_WHEAT | 6.2E-107 |
| DN20721_c1_g1_i28 | 10.08 | 6.02 | 1.3E-43 | 2.9E-40 | #N/A | #N/A |
| DN15137_c0_g2_i5 | 12.76 | 4.58 | 2.2E-43 | 4.7E-40 | BADH1_ORYSJ | 0 |
| DN19502_c1_g1_i4 | -12.18 | 3.00 | 8.9E-42 | 1.9E-38 | #N/A | #N/A |
| DN20069_c0_g1_i2 | 12.07 | 3.89 | 2.0E-41 | 4.1E-38 | VPS39_MOUSE | 4.9E-72 |
| DN18889_c0_g2_i3 | 11.80 | 3.63 | 4.4E-41 | 8.7E-38 | #N/A | #N/A |
| DN21011_c2_g1_i3 | -10.09 | 3.21 | 1.3E-39 | 2.5E-36 | #N/A | #N/A |
| DN18413_c0_g1_i2 | 12.31 | 4.13 | 9.9E-39 | 1.8E-35 | Y3544_ARATH | 3.4E-40 |
| DN21008_c0_g1_i12 | 8.47 | 4.96 | 5.4E-38 | 9.8E-35 | ZEP_ORYSJ | 0 |
| DN17090_c0_g1_i3 | -11.94 | 2.77 | 3.8E-37 | 6.7E-34 | #N/A | #N/A |
| DN15210_c0_g2_i2 | 12.22 | 4.04 | 1.2E-36 | 2.0E-33 | CDKAL_XENTR | 8.22E-180 |
| DN17408_c0_g2_i6 | 11.62 | 3.44 | 4.0E-36 | 6.7E-33 | TM147_XENLA | 1.28E-27 |
| DN20475_c0_g2_i4 | 11.93 | 3.75 | 1.6E-35 | 2.6E-32 | RRP3_HORVU | 3.01E-56 |
| DN19128_c0_g2_i15 | 12.36 | 4.18 | 6.0E-35 | 9.5E-32 | DRB2_ORYSJ | 2.24E-36 |
| DN15013_c0_g1_i2 | 11.64 | 3.46 | 9.6E-35 | 1.5E-31 | #N/A | #N/A |
| DN19873_c0_g5_i2 | -11.76 | 2.59 | 2.0E-34 | 3.0E-31 | TF26_SCHPO | 9.5E-138 |
| DN14303_c0_g1_i6 | 12.08 | 3.90 | 2.9E-34 | 4.2E-31 | AT18F_ARATH | 8.84E-61 |
| DN22114_c0_g1_i18 | 11.45 | 3.28 | 5.0E-34 | 7.2E-31 | TRH22_ORYSJ | 1.14E-57 |
| DN18612_c0_g1_i6 | -11.68 | 2.51 | 2.3E-33 | 3.2E-30 | PNSB2_ARATH | 2.37E-107 |
| DN16085_c2_g1_i13 | 11.67 | 3.49 | 1.5E-32 | 2.1E-29 | #N/A | #N/A |
| DN22454_c0_g1_i20 | 11.65 | 3.48 | 2.6E-32 | 3.4E-29 | OHK4_ORYSJ | 0 |
| DN18612_c0_g1_i12 | -12.39 | 3.22 | 6.4E-32 | 8.5E-29 | PNSB2_ARATH | 1.81E-108 |
| DN13135_c0_g1_i1 | 11.80 | 3.62 | 1.0E-31 | 1.3E-28 | #N/A | #N/A |
| DN18772_c2_g1_i17 | -6.75 | 3.20 | 1.5E-31 | 1.9E-28 | THF1_ORYSJ | 6.5E-116 |
| DN29579_c0_g1_i1 | 14.43 | 6.25 | 3.0E-31 | 3.7E-28 | PAT_STRHY | 3.27E-124 |
| DN21891_c0_g1_i6 | 11.25 | 3.07 | 3.8E-31 | 4.6E-28 | #N/A | #N/A |
| DN20475_c0_g2_i15 | 7.69 | 4.57 | 4.1E-31 | 4.9E-28 | RRP3_HORVU | 1.13E-68 |
| DN15555_c1_g3_i4 | -11.67 | 2.50 | 4.2E-31 | 4.9E-28 | MSR21_ORYSJ | 6.12E-109 |
| DN20042_c0_g2_i16 | -10.06 | 2.45 | 8.4E-31 | 9.6E-28 | UGAL2_ARATH | 2.84E-77 |
| DN22700_c4_g3_i12 | 11.67 | 3.49 | 2.2E-30 | 2.5E-27 | HFA2E_ORYSJ | 3.79E-116 |
| DN18236_c0_g1_i3 | 11.29 | 3.12 | 3.0E-30 | 3.4E-27 | Y3078_ARATH | 0 |
| DN17092_c0_g2_i9 | 11.14 | 2.97 | 3.1E-30 | 3.4E-27 | RECAC_ARATH | 0 |
| DN19342_c2_g2_i6 | 11.37 | 3.20 | 6.0E-30 | 6.5E-27 | #N/A | #N/A |
| DN15346_c0_g1_i24 | -7.93 | 2.80 | 6.5E-30 | 6.8E-27 | #N/A | #N/A |
| DN18060_c0_g4_i11 | -11.55 | 2.38 | 1.6E-29 | 1.7E-26 | #N/A | #N/A |
| DN17900_c0_g1_i9 | -11.64 | 2.47 | 1.3E-28 | 1.3E-25 | ZDH14_ARATH | 6.73E-114 |
| DN20475_c0_g2_i16 | 7.99 | 3.55 | 1.5E-28 | 1.5E-25 | #N/A | #N/A |
| DN22129_c0_g1_i8 | 7.71 | 4.31 | 1.6E-28 | 1.5E-25 | TSJT1_TOBAC | 3.8E-21 |
| DN20826_c0_g3_i1 | 11.15 | 2.98 | 1.9E-28 | 1.8E-25 | #N/A | #N/A |
| DN22372_c0_g1_i19 | -9.43 | 2.56 | 8.6E-28 | 8.3E-25 | RRP4_ARATH | 2.7E-38 |
| DN18159_c0_g4_i1 | -11.79 | 2.62 | 1.1E-27 | 1.0E-24 | IPO5_MOUSE | 0 |
| DN16683_c0_g2_i9 | -12.43 | 3.25 | 1.2E-27 | 1.1E-24 | CCDA2_ORYSJ | 1.08E-173 |
| DN18231_c0_g1_i1 | -11.51 | 2.35 | 1.6E-27 | 1.5E-24 | PB27A_ARATH | 2.24E-53 |
| DN17537_c0_g1_i17 | 12.40 | 4.22 | 1.8E-27 | 1.7E-24 | #N/A | #N/A |
| DN17139_c0_g1_i6 | -11.47 | 2.30 | 1.9E-27 | 1.7E-24 | RKP_ARATH | 0 |
| DN17502_c0_g2_i4 | -11.30 | 2.13 | 1.9E-27 | 1.7E-24 | TI172_ARATH | 1.42E-68 |
| DN16431_c0_g1_i5 | 11.09 | 2.92 | 1.6E-26 | 1.4E-23 | PGP1B_ARATH | 2.92E-163 |
| DN21684_c0_g1_i3 | 11.47 | 3.29 | 2.0E-26 | 1.7E-23 | ZEP_ORYSJ | 2.87E-138 |
| DN21210_c0_g1_i4 | -11.28 | 2.12 | 3.2E-26 | 2.7E-23 | IFRD1_PIG | 1.07E-25 |
| DN19372_c1_g2_i4 | 11.62 | 3.45 | 6.3E-26 | 5.3E-23 | ZPR1_MOUSE | 1.2E-56 |
| DN20830_c0_g1_i5 | 11.65 | 3.47 | 1.2E-25 | 9.5E-23 | PERK4_ARATH | 1.36E-120 |
| DN19873_c0_g5_i4 | -11.13 | 1.97 | 2.5E-25 | 2.1E-22 | TF26_SCHPO | 1.01E-142 |
| DN18821_c0_g6_i1 | 7.06 | 3.31 | 3.3E-25 | 2.7E-22 | HFA2E_ORYSJ | 1.76E-42 |
| DN20194_c0_g2_i1 | -11.73 | 2.56 | 6.5E-25 | 5.2E-22 | TRPA1_ARATH | 3.41E-128 |
| DN22700_c4_g3_i8 | 10.94 | 2.77 | 7.6E-25 | 5.9E-22 | HFA2E_ORYSJ | 1.33E-115 |
| DN20058_c0_g2_i4 | 10.73 | 2.56 | 3.7E-24 | 2.8E-21 | #N/A | #N/A |
| DN19588_c0_g1_i12 | 13.76 | 7.93 | 1.2E-23 | 8.9E-21 | FER1_MAIZE | 1.85E-57 |
| DN17408_c0_g2_i4 | 10.82 | 2.65 | 1.2E-23 | 9.1E-21 | #N/A | #N/A |
| DN22561_c1_g1_i15 | -11.14 | 1.98 | 1.5E-23 | 1.1E-20 | #N/A | #N/A |
| DN19062_c0_g3_i2 | -11.02 | 1.87 | 1.8E-22 | 1.3E-19 | #N/A | #N/A |
| DN22392_c0_g1_i18 | 10.91 | 2.75 | 1.9E-22 | 1.4E-19 | NLP2_ORYSJ | 0 |
| DN21210_c0_g1_i3 | -10.90 | 1.75 | 2.1E-22 | 1.5E-19 | IFRD1_MOUSE | 4.89E-28 |
| DN20698_c0_g1_i1 | -10.87 | 1.71 | 2.2E-22 | 1.6E-19 | Y1461_ARATH | 1.33E-177 |
| DN16431_c0_g1_i39 | 7.52 | 2.57 | 3.4E-22 | 2.4E-19 | PGP1B_ARATH | 7.22E-72 |
| DN20916_c0_g1_i5 | 11.97 | 3.80 | 3.7E-22 | 2.6E-19 | SKI32_ARATH | 4.79E-144 |
| DN20363_c0_g4_i5 | 10.89 | 2.72 | 4.5E-22 | 3.1E-19 | TIC40_PEA | 6.06E-85 |
| DN17483_c0_g1_i12 | 10.46 | 2.29 | 8.7E-22 | 5.9E-19 | #N/A | #N/A |
| DN16431_c0_g1_i12 | 8.91 | 3.14 | 1.2E-21 | 7.9E-19 | PGP1B_ARATH | 1.32E-161 |
| DN22624_c4_g1_i10 | -2.72 | 5.04 | 1.2E-21 | 8.2E-19 | MOSA_MAIZE | 3.28E-20 |
| DN17467_c6_g3_i1 | 10.42 | 2.25 | 2.6E-21 | 1.7E-18 | NDUS1_SOLTU | 2.4E-57 |
| DN15312_c1_g1_i1 | 11.14 | 2.97 | 4.0E-21 | 2.6E-18 | ZEAM_MAIZE | 1.18E-99 |
| DN20830_c0_g1_i20 | 8.06 | 3.10 | 5.1E-21 | 3.3E-18 | PERK4_ARATH | 4.13E-120 |
| DN15252_c0_g2_i1 | 8.45 | 2.65 | 5.3E-21 | 3.4E-18 | #N/A | #N/A |
| DN22411_c0_g3_i8 | 10.92 | 2.76 | 5.4E-21 | 3.4E-18 | C3H28_ORYSJ | 1.71E-99 |
| DN13819_c0_g1_i3 | 10.46 | 2.30 | 6.0E-21 | 3.8E-18 | #N/A | #N/A |
| DN21438_c0_g3_i1 | 11.63 | 3.46 | 1.2E-20 | 7.2E-18 | SBDS_MOUSE | 5.62E-75 |
| DN19711_c0_g1_i7 | 10.58 | 2.42 | 1.2E-20 | 7.3E-18 | #N/A | #N/A |
| DN21684_c0_g1_i14 | 3.81 | 4.94 | 1.3E-20 | 7.7E-18 | ZEP_ORYSJ | 3.13E-77 |
| DN15917_c0_g1_i17 | 7.38 | 2.43 | 1.4E-20 | 8.7E-18 | TAS_SHIFL | 1.47E-21 |
| DN18231_c0_g1_i7 | -10.72 | 1.57 | 1.9E-20 | 1.1E-17 | PB27A_ARATH | 5.54E-52 |
| DN21224_c0_g1_i14 | 10.48 | 2.32 | 2.1E-20 | 1.3E-17 | M3K1_ARATH | 6.27E-110 |
| DN21041_c0_g4_i1 | -7.44 | 2.35 | 2.3E-20 | 1.4E-17 | #N/A | #N/A |
| DN18760_c1_g1_i7 | 10.42 | 2.26 | 2.8E-20 | 1.6E-17 | #N/A | #N/A |
| DN15532_c0_g1_i1 | -10.63 | 1.48 | 4.8E-20 | 2.8E-17 | #N/A | #N/A |
| DN20660_c0_g2_i5 | 11.15 | 2.98 | 6.5E-20 | 3.7E-17 | #N/A | #N/A |
| DN16105_c1_g3_i4 | 11.54 | 3.36 | 1.4E-19 | 7.8E-17 | BCHB_ARATH | 0 |
| DN13668_c1_g2_i1 | 11.57 | 3.40 | 1.5E-19 | 8.7E-17 | #N/A | #N/A |
| DN21388_c0_g1_i1 | -10.54 | 1.40 | 2.0E-19 | 1.1E-16 | WNK1_ORYSJ | 0 |
| DN17649_c0_g1_i11 | 6.37 | 2.86 | 2.0E-19 | 1.1E-16 | AP1S2_ARATH | 5.22E-80 |
| DN18859_c0_g1_i23 | 10.79 | 2.62 | 2.4E-19 | 1.3E-16 | BI1L_ARATH | 6.21E-86 |
| DN19026_c0_g1_i1 | 10.37 | 2.21 | 2.5E-19 | 1.3E-16 | #N/A | #N/A |
| DN19076_c0_g1_i10 | 10.35 | 2.19 | 2.5E-19 | 1.4E-16 | TBP1_ORYSJ | 0 |
| DN20721_c1_g1_i15 | 10.52 | 2.35 | 2.5E-19 | 1.4E-16 | #N/A | #N/A |
| DN19290_c1_g1_i13 | -9.20 | 1.59 | 2.8E-19 | 1.5E-16 | Y5102_ARATH | 4.09E-32 |
| DN18330_c0_g1_i6 | 10.39 | 2.23 | 2.9E-19 | 1.5E-16 | NAA16_MOUSE | 3.47E-139 |
| DN17483_c0_g1_i10 | -10.54 | 1.40 | 3.0E-19 | 1.6E-16 | #N/A | #N/A |
| DN14994_c0_g3_i10 | -10.84 | 1.69 | 5.6E-19 | 2.9E-16 | CAR7_ARATH | 7.87E-44 |
| DN18274_c0_g5_i2 | -11.94 | 2.77 | 6.1E-19 | 3.1E-16 | ODPB3_ORYSJ | 0 |
| DN19502_c1_g1_i27 | -10.98 | 1.83 | 6.4E-19 | 3.2E-16 | #N/A | #N/A |
| DN22058_c1_g1_i7 | 10.10 | 7.86 | 9.9E-19 | 5.0E-16 | HSP7S_PEA | 0 |
| DN19774_c0_g2_i3 | 3.49 | 4.77 | 1.0E-18 | 5.0E-16 | IFRH_TOBAC | 5.26E-90 |
| DN17980_c0_g4_i6 | 10.35 | 2.19 | 1.5E-18 | 7.2E-16 | #N/A | #N/A |
| DN17980_c0_g4_i2 | 11.03 | 2.86 | 1.5E-18 | 7.4E-16 | #N/A | #N/A |
| DN22025_c1_g1_i8 | -10.99 | 1.83 | 1.7E-18 | 8.5E-16 | AB4F_ARATH | 0 |
| DN20721_c1_g1_i9 | -2.33 | 4.85 | 1.9E-18 | 9.1E-16 | #N/A | #N/A |
| DN20043_c0_g2_i4 | 13.69 | 5.51 | 2.3E-18 | 1.1E-15 | PSY_MAIZE | 2.51E-133 |
| DN20555_c0_g1_i20 | -11.16 | 2.00 | 2.9E-18 | 1.4E-15 | #N/A | #N/A |
| DN21928_c1_g5_i3 | 10.34 | 2.18 | 5.0E-18 | 2.4E-15 | IMDH_VIGUN | 0 |
| DN20350_c0_g1_i5 | 12.03 | 3.86 | 5.6E-18 | 2.6E-15 | #N/A | #N/A |
| DN22140_c1_g5_i1 | -10.48 | 1.34 | 5.9E-18 | 2.7E-15 | RAP24_ARATH | 2.16E-26 |
| DN22571_c0_g1_i11 | -8.55 | 1.69 | 6.1E-18 | 2.8E-15 | BIG5_ARATH | 0 |
| DN22756_c3_g1_i10 | 10.93 | 2.76 | 8.7E-18 | 4.0E-15 | #N/A | #N/A |
| DN21745_c1_g1_i6 | -10.38 | 1.24 | 1.1E-17 | 5.1E-15 | CDPK2_ORYSJ | 0 |
| DN19390_c2_g1_i1 | 12.04 | 7.10 | 1.2E-17 | 5.4E-15 | UCRIA_WHEAT | 6.5E-72 |
| DN16895_c0_g1_i11 | -10.52 | 1.38 | 1.5E-17 | 6.6E-15 | ENT3_ARATH | 1.11E-174 |
| DN16873_c0_g2_i11 | 10.97 | 2.80 | 1.6E-17 | 7.1E-15 | U83A1_ARATH | 1.87E-112 |
| DN17233_c1_g1_i1 | 10.63 | 2.47 | 1.6E-17 | 7.1E-15 | TI222_ARATH | 2.72E-38 |
| DN17364_c1_g6_i1 | 7.88 | 2.09 | 1.8E-17 | 7.8E-15 | #N/A | #N/A |
| DN19507_c0_g1_i1 | 10.56 | 2.40 | 1.8E-17 | 7.8E-15 | MED27_ARATH | 1.43E-140 |
| DN19415_c0_g1_i15 | -10.28 | 1.14 | 2.1E-17 | 8.9E-15 | BEM46_SCHPO | 9.49E-55 |
| DN16149_c1_g2_i3 | 10.75 | 2.59 | 2.8E-17 | 1.2E-14 | PFD4_AVEFA | 5.98E-64 |
| DN16776_c0_g2_i2 | -10.31 | 1.17 | 2.9E-17 | 1.2E-14 | CDPK7_ARATH | 0 |
| DN18612_c0_g1_i1 | -10.28 | 1.15 | 3.1E-17 | 1.3E-14 | PNSB2_ARATH | 8.19E-85 |
| DN22466_c0_g1_i3 | 6.88 | 2.45 | 3.1E-17 | 1.3E-14 | XPO2_ARATH | 0 |
| DN19502_c1_g1_i14 | -10.38 | 1.24 | 5.1E-17 | 2.1E-14 | #N/A | #N/A |
| DN22057_c1_g2_i2 | 6.74 | 2.32 | 6.0E-17 | 2.5E-14 | #N/A | #N/A |
| DN17148_c1_g2_i11 | 12.93 | 4.75 | 6.0E-17 | 2.5E-14 | PSBY_SPIOL | 5.19E-21 |
| DN21831_c0_g1_i9 | -10.22 | 1.08 | 6.4E-17 | 2.6E-14 | #N/A | #N/A |
| DN21818_c1_g3_i3 | 13.21 | 5.03 | 2.0E-16 | 8.0E-14 | #N/A | #N/A |
| DN18608_c1_g1_i9 | 6.66 | 3.04 | 2.5E-16 | 1.0E-13 | IP5PA_ARATH | 8.46E-156 |
| DN16316_c0_g2_i10 | 12.40 | 4.21 | 3.0E-16 | 1.2E-13 | HBPL1_ARATH | 3.56E-70 |
| DN19128_c0_g2_i9 | 10.43 | 2.27 | 3.6E-16 | 1.4E-13 | #N/A | #N/A |
| DN17364_c1_g1_i3 | 8.47 | 2.67 | 3.7E-16 | 1.5E-13 | KNOS2_ORYSJ | 4.24E-179 |
| DN21891_c0_g1_i2 | 10.23 | 2.07 | 4.7E-16 | 1.9E-13 | #N/A | #N/A |
| DN15487_c1_g1_i6 | -10.16 | 1.03 | 5.2E-16 | 2.0E-13 | #N/A | #N/A |
| DN13182_c0_g1_i6 | -3.05 | 4.12 | 1.4E-15 | 5.4E-13 | PHP5_ORYSJ | 2.85E-87 |
| DN22454_c0_g1_i12 | 12.22 | 4.04 | 2.0E-15 | 7.7E-13 | OHK4_ORYSJ | 0 |
| DN16357_c0_g1_i2 | -10.37 | 1.23 | 2.1E-15 | 8.1E-13 | AN13B_HUMAN | 1.34E-29 |
| DN19602_c0_g2_i21 | -10.87 | 1.72 | 2.3E-15 | 9.0E-13 | GGP3_ARATH | 3.6E-63 |
| DN22700_c4_g3_i13 | 11.08 | 2.91 | 2.7E-15 | 1.0E-12 | HFA2E_ORYSJ | 2.06E-112 |
| DN16041_c0_g2_i4 | 9.83 | 1.68 | 2.7E-15 | 1.0E-12 | #N/A | #N/A |
| DN14092_c0_g1_i5 | 10.37 | 2.21 | 2.9E-15 | 1.1E-12 | DGP14_ARATH | 2.17E-148 |
| DN19502_c1_g1_i18 | -8.66 | 2.67 | 3.3E-15 | 1.2E-12 | #N/A | #N/A |
| DN17979_c0_g3_i14 | 9.76 | 1.61 | 3.8E-15 | 1.4E-12 | CFM2_ARATH | 2.33E-139 |
| DN18505_c0_g1_i4 | 9.90 | 1.75 | 4.5E-15 | 1.7E-12 | ASP1_ORYSJ | 2.53E-111 |
| DN19513_c0_g4_i2 | 12.51 | 4.33 | 4.5E-15 | 1.7E-12 | SLU7_ORYSJ | 0 |
| DN17502_c0_g2_i6 | -10.11 | 0.98 | 6.1E-15 | 2.2E-12 | TI172_ARATH | 1.09E-70 |
| DN17212_c0_g3_i11 | 3.15 | 4.31 | 6.8E-15 | 2.5E-12 | DHBK_SOLLC | 1.36E-130 |
| DN20592_c0_g4_i3 | 11.13 | 2.96 | 7.0E-15 | 2.5E-12 | OPT4_ARATH | 0 |
| DN17773_c0_g1_i3 | 11.73 | 3.55 | 7.7E-15 | 2.8E-12 | DNJH1_ALLPO | 3.8E-146 |
| DN18120_c0_g4_i1 | 12.11 | 3.94 | 8.0E-15 | 2.9E-12 | PP163_ARATH | 0 |
| DN16636_c2_g1_i5 | 12.02 | 3.84 | 9.1E-15 | 3.2E-12 | #N/A | #N/A |
| DN18888_c0_g2_i19 | 11.18 | 3.01 | 9.6E-15 | 3.4E-12 | ARSB_DICDI | 1.12E-34 |
| DN20663_c0_g1_i4 | -9.88 | 0.76 | 1.0E-14 | 3.7E-12 | DPNPM_ARATH | 1.01E-38 |
| DN20830_c0_g1_i4 | -2.20 | 4.43 | 1.2E-14 | 4.1E-12 | PERK4_ARATH | 4.13E-120 |
| DN16679_c1_g1_i11 | 10.09 | 1.94 | 1.4E-14 | 4.7E-12 | SAPK3_ORYSJ | 3.47E-21 |
| DN15985_c0_g2_i2 | 10.69 | 2.52 | 1.4E-14 | 4.7E-12 | #N/A | #N/A |
| DN18105_c2_g1_i12 | -9.82 | 0.71 | 1.5E-14 | 5.0E-12 | #N/A | #N/A |
| DN20364_c1_g2_i5 | 10.19 | 2.03 | 1.6E-14 | 5.5E-12 | MPV17_DANRE | 1.06E-26 |
| DN15627_c0_g3_i2 | -10.22 | 1.09 | 1.6E-14 | 5.5E-12 | CB4A_ARATH | 9.42E-154 |
| DN22255_c0_g2_i3 | -9.89 | 0.77 | 1.7E-14 | 5.8E-12 | #N/A | #N/A |
| DN19722_c0_g5_i2 | 10.57 | 2.41 | 1.7E-14 | 5.9E-12 | #N/A | #N/A |
| DN20684_c1_g1_i14 | 13.21 | 5.03 | 1.8E-14 | 5.9E-12 | PPOC_ORYSJ | 0 |
| DN22756_c3_g1_i22 | 10.54 | 2.38 | 2.8E-14 | 9.4E-12 | #N/A | #N/A |
| DN17139_c0_g1_i7 | -10.04 | 0.91 | 2.9E-14 | 9.6E-12 | RKP_ARATH | 0 |
| DN19247_c0_g1_i1 | -2.74 | 3.44 | 3.0E-14 | 9.8E-12 | BZIP9_ARATH | 7.5E-26 |
| DN20387_c0_g2_i2 | 11.46 | 3.29 | 3.0E-14 | 9.8E-12 | PP1_MEDSV | 0 |
| DN15367_c0_g4_i6 | 10.01 | 1.86 | 3.3E-14 | 1.1E-11 | CAAT1_ARATH | 0 |
| DN15465_c2_g1_i7 | 9.62 | 1.47 | 3.3E-14 | 1.1E-11 | YODA_ARATH | 1.31E-61 |
| DN17231_c1_g1_i4 | 10.16 | 2.01 | 3.5E-14 | 1.1E-11 | SUV3M_ORYSJ | 0 |
| DN15449_c0_g5_i2 | 7.31 | 2.35 | 3.6E-14 | 1.1E-11 | #N/A | #N/A |
| DN19423_c0_g1_i5 | 5.87 | 2.37 | 3.7E-14 | 1.2E-11 | NOP14_MOUSE | 2.91E-23 |
| DN22439_c1_g2_i2 | 13.57 | 5.39 | 4.4E-14 | 1.4E-11 | PSBO_SOLTU | 3.61E-45 |
| DN20294_c0_g1_i11 | 9.93 | 1.78 | 4.5E-14 | 1.4E-11 | EIL3_ARATH | 1.74E-131 |
| DN20294_c0_g1_i18 | -9.77 | 0.65 | 5.0E-14 | 1.6E-11 | EIL3_ARATH | 2.98E-131 |
| DN19907_c0_g4_i3 | 10.27 | 2.12 | 5.3E-14 | 1.7E-11 | MIP2_ARATH | 0 |
| DN14927_c1_g2_i14 | 9.69 | 1.55 | 5.3E-14 | 1.7E-11 | #N/A | #N/A |
| DN21721_c0_g2_i3 | 11.86 | 3.68 | 7.9E-14 | 2.4E-11 | SAG39_ORYSI | 1.72E-110 |
| DN20133_c0_g2_i13 | 9.77 | 1.62 | 8.0E-14 | 2.5E-11 | CSPLH_MAIZE | 1.59E-70 |
| DN20788_c0_g1_i4 | 12.55 | 4.37 | 8.2E-14 | 2.5E-11 | #N/A | #N/A |
| DN19271_c2_g1_i16 | 9.46 | 1.32 | 8.8E-14 | 2.7E-11 | WRK74_ARATH | 1.59E-60 |
| DN19411_c1_g3_i1 | 2.54 | 5.03 | 9.7E-14 | 2.9E-11 | BGH3B_BACO1 | 3.62E-82 |
| DN22274_c1_g1_i6 | -9.86 | 0.74 | 1.1E-13 | 3.4E-11 | FRS5_ARATH | 2.87E-102 |
| DN22274_c1_g1_i12 | -9.72 | 0.61 | 1.3E-13 | 3.9E-11 | FRS5_ARATH | 4.21E-103 |
| DN16418_c1_g2_i6 | -9.70 | 0.59 | 1.4E-13 | 4.3E-11 | ARFW_ORYSJ | 3.9E-149 |
| DN16942_c0_g3_i6 | 13.22 | 5.04 | 1.4E-13 | 4.3E-11 | IDS3_HORVU | 4.26E-179 |
| DN14321_c0_g2_i1 | 7.32 | 1.54 | 1.5E-13 | 4.4E-11 | #N/A | #N/A |
| DN19271_c2_g1_i11 | 9.47 | 1.33 | 1.5E-13 | 4.4E-11 | WRK74_ARATH | 6.5E-28 |
| DN21928_c1_g5_i12 | 9.85 | 1.70 | 1.6E-13 | 4.7E-11 | IMDH2_ARATH | 3.65E-157 |
| DN13899_c0_g1_i2 | -9.79 | 0.67 | 1.6E-13 | 4.7E-11 | #N/A | #N/A |
| DN21009_c2_g1_i2 | -9.65 | 0.55 | 1.6E-13 | 4.8E-11 | #N/A | #N/A |
| DN15535_c1_g7_i2 | 2.42 | 5.59 | 1.7E-13 | 4.8E-11 | **RHM1_ARATH** | 0 |
| DN13811_c0_g1_i3 | 6.53 | 1.63 | 1.7E-13 | 4.9E-11 | CNR6_MAIZE | 3.46E-103 |
| DN18249_c2_g1_i5 | 9.42 | 1.29 | 1.7E-13 | 5.0E-11 | C81E1_GLYEC | 7.19E-74 |
| DN21041_c0_g2_i3 | -2.37 | 5.70 | 1.9E-13 | 5.4E-11 | CB12_SOLLC | 5.09E-48 |
| DN22392_c0_g1_i23 | 12.18 | 4.00 | 2.0E-13 | 5.6E-11 | NLP2_ORYSJ | 0 |
| DN18047_c0_g2_i2 | -9.67 | 0.56 | 2.0E-13 | 5.8E-11 | WDR12_SALSA | 1.81E-54 |
| DN17483_c0_g1_i19 | 9.59 | 1.45 | 2.2E-13 | 6.1E-11 | #N/A | #N/A |
| DN18269_c0_g1_i11 | 12.70 | 4.52 | 2.3E-13 | 6.5E-11 | CYSKP_SOLTU | 6.57E-174 |
| DN16210_c0_g4_i5 | -9.59 | 0.48 | 2.3E-13 | 6.5E-11 | #N/A | #N/A |
| DN16232_c1_g2_i12 | 4.28 | 5.96 | 2.3E-13 | 6.5E-11 | ODBA2_ARATH | 0 |
| DN15949_c0_g1_i11 | 9.35 | 1.21 | 2.6E-13 | 7.3E-11 | #N/A | #N/A |
| DN14175_c0_g1_i4 | -1.75 | 6.50 | 2.7E-13 | 7.5E-11 | #N/A | #N/A |
| DN21684_c0_g1_i7 | 4.33 | 4.58 | 2.9E-13 | 8.1E-11 | #N/A | #N/A |
| DN18607_c1_g2_i3 | 6.32 | 2.27 | 3.0E-13 | 8.3E-11 | #N/A | #N/A |
| DN21717_c3_g2_i19 | 12.61 | 4.43 | 3.4E-13 | 9.2E-11 | PIP_ARATH | 0 |
| DN19913_c1_g3_i9 | 9.78 | 1.63 | 3.4E-13 | 9.2E-11 | BPM4_ARATH | 8.95E-155 |
| DN19513_c0_g3_i2 | 12.48 | 4.30 | 3.4E-13 | 9.2E-11 | RL354_ARATH | 3.26E-61 |
| DN22255_c0_g2_i4 | 9.54 | 1.40 | 3.5E-13 | 9.5E-11 | #N/A | #N/A |
| DN20304_c0_g1_i2 | -9.51 | 0.41 | 4.4E-13 | 1.2E-10 | #N/A | #N/A |
| DN18060_c0_g4_i8 | -9.52 | 0.42 | 4.5E-13 | 1.2E-10 | #N/A | #N/A |
| DN20824_c1_g2_i1 | -10.64 | 1.49 | 4.6E-13 | 1.2E-10 | #N/A | #N/A |
| DN17033_c2_g2_i4 | 9.95 | 1.79 | 4.7E-13 | 1.2E-10 | #N/A | #N/A |
| DN19676_c0_g2_i4 | 10.00 | 1.85 | 4.8E-13 | 1.3E-10 | 2A5N_ARATH | 0 |
| DN17955_c1_g1_i5 | 5.92 | 1.94 | 5.7E-13 | 1.5E-10 | #N/A | #N/A |
| DN15058_c0_g4_i3 | -9.50 | 0.40 | 5.9E-13 | 1.5E-10 | #N/A | #N/A |
| DN20915_c3_g2_i5 | 11.58 | 3.40 | 6.0E-13 | 1.6E-10 | #N/A | #N/A |
| DN20835_c3_g1_i1 | 10.05 | 1.90 | 6.3E-13 | 1.6E-10 | #N/A | #N/A |
| DN16340_c3_g1_i10 | 11.16 | 2.99 | 7.1E-13 | 1.8E-10 | RS52_ARATH | 5.51E-123 |
| DN18116_c0_g1_i4 | -9.49 | 0.39 | 7.1E-13 | 1.8E-10 | RBP45_NICPL | 4.59E-55 |
| DN19235_c0_g1_i7 | -9.93 | 0.81 | 7.8E-13 | 2.0E-10 | #N/A | #N/A |
| DN15308_c0_g1_i1 | -2.10 | 5.55 | 7.9E-13 | 2.0E-10 | DBNBT_TAXCA | 6.42E-52 |
| DN17547_c1_g1_i5 | 3.59 | 2.58 | 8.5E-13 | 2.2E-10 | #N/A | #N/A |
| DN14910_c0_g1_i4 | 11.35 | 5.51 | 9.2E-13 | 2.3E-10 | G3PC2_MAIZE | 4.13E-76 |
| DN14810_c5_g3_i8 | 5.76 | 2.46 | 9.8E-13 | 2.5E-10 | #N/A | #N/A |
| DN17071_c3_g4_i4 | -9.57 | 0.46 | 1.1E-12 | 2.6E-10 | #N/A | #N/A |
| DN21210_c0_g1_i15 | 2.15 | 5.76 | 1.1E-12 | 2.7E-10 | IFRD1_MOUSE | 4.5E-28 |
| DN18069_c1_g1_i5 | 6.13 | 2.08 | 1.2E-12 | 3.1E-10 | PUX1_ARATH | 2.55E-53 |
| DN17201_c0_g1_i23 | 9.99 | 1.84 | 1.3E-12 | 3.2E-10 | #N/A | #N/A |
| DN19588_c0_g1_i10 | 12.14 | 3.96 | 1.4E-12 | 3.4E-10 | FER5_MAIZE | 5.59E-66 |
| DN20830_c0_g1_i7 | 7.08 | 2.13 | 1.4E-12 | 3.4E-10 | PERK4_ARATH | 7.26E-113 |
| DN19536_c1_g2_i18 | -10.18 | 1.05 | 1.4E-12 | 3.5E-10 | FRS5_ARATH | 2.4E-94 |
| DN15553_c0_g4_i16 | 12.18 | 4.00 | 1.6E-12 | 3.9E-10 | DPNP_ORYSJ | 0 |
| DN22334_c1_g1_i6 | 10.96 | 2.80 | 1.6E-12 | 3.9E-10 | PEX6_ARATH | 0 |
| DN22123_c0_g4_i5 | 9.71 | 1.57 | 1.8E-12 | 4.4E-10 | #N/A | #N/A |
| DN18776_c0_g1_i10 | -2.91 | 2.91 | 1.8E-12 | 4.4E-10 | GCP5_MACFA | 5.83E-31 |
| DN16442_c0_g1_i19 | 11.98 | 3.80 | 1.9E-12 | 4.5E-10 | #N/A | #N/A |
| DN18377_c0_g3_i13 | -11.25 | 2.09 | 1.9E-12 | 4.6E-10 | #N/A | #N/A |
| DN18255_c0_g4_i5 | -9.42 | 0.33 | 2.0E-12 | 4.9E-10 | #N/A | #N/A |
| DN20555_c0_g1_i22 | -9.89 | 0.77 | 2.1E-12 | 5.0E-10 | #N/A | #N/A |
| DN17090_c0_g2_i10 | -3.32 | 1.86 | 2.5E-12 | 6.0E-10 | #N/A | #N/A |
| DN20015_c0_g1_i13 | 6.01 | 2.49 | 2.6E-12 | 6.2E-10 | CGEP_ORYSJ | 0 |
| DN14843_c0_g3_i6 | -2.10 | 7.71 | 2.8E-12 | 6.5E-10 | #N/A | #N/A |
| DN15007_c0_g1_i11 | -9.39 | 0.29 | 2.9E-12 | 6.8E-10 | Y5738_ARATH | 6.93E-179 |
| DN13983_c0_g1_i2 | 7.42 | 1.63 | 2.9E-12 | 6.8E-10 | ACPM3_ARATH | 6.86E-31 |
| DN17441_c0_g3_i4 | 10.39 | 2.23 | 3.0E-12 | 6.9E-10 | YIPL1_ARATH | 1.11E-47 |
| DN20243_c0_g1_i10 | -9.46 | 0.36 | 4.2E-12 | 9.7E-10 | #N/A | #N/A |
| DN18184_c1_g2_i3 | -6.25 | 0.83 | 4.2E-12 | 9.7E-10 | #N/A | #N/A |
| DN21597_c2_g1_i7 | 11.42 | 3.25 | 4.3E-12 | 9.8E-10 | CHLI_ORYSJ | 0 |
| DN20411_c0_g1_i3 | 12.13 | 3.96 | 4.4E-12 | 1.0E-09 | PGKH_WHEAT | 1.62E-50 |
| DN20499_c0_g1_i24 | 9.52 | 1.37 | 4.5E-12 | 1.0E-09 | #N/A | #N/A |
| DN15553_c0_g4_i12 | 11.55 | 3.37 | 5.0E-12 | 1.1E-09 | DPNP_ORYSJ | 0 |
| DN21014_c1_g3_i2 | 12.29 | 4.11 | 5.1E-12 | 1.2E-09 | #N/A | #N/A |
| DN19404_c0_g1_i11 | 10.00 | 1.84 | 5.5E-12 | 1.3E-09 | #N/A | #N/A |
| DN18931_c0_g1_i2 | 9.27 | 1.14 | 6.3E-12 | 1.4E-09 | #N/A | #N/A |
| DN22700_c4_g3_i14 | 9.66 | 1.52 | 6.3E-12 | 1.4E-09 | HFA2E_ORYSJ | 8.04E-111 |
| DN20244_c2_g1_i10 | -9.77 | 0.65 | 6.5E-12 | 1.5E-09 | #N/A | #N/A |
| DN19566_c0_g1_i4 | -9.35 | 0.26 | 7.1E-12 | 1.6E-09 | NCBP1_ORYSJ | 0 |
| DN21193_c0_g3_i6 | 6.28 | 1.96 | 7.4E-12 | 1.6E-09 | PARP3_ORYSJ | 0 |
| DN17272_c0_g1_i12 | 11.62 | 3.44 | 7.7E-12 | 1.7E-09 | ARFX_ORYSJ | 0 |
| DN15276_c2_g2_i15 | 11.45 | 3.28 | 7.8E-12 | 1.7E-09 | MPPA_SOLTU | 0 |
| DN22422_c0_g1_i3 | 9.32 | 1.19 | 8.0E-12 | 1.8E-09 | FIPS5_ARATH | 1.68E-69 |
| DN19104_c0_g3_i4 | 10.10 | 5.09 | 8.0E-12 | 1.8E-09 | #N/A | #N/A |
| DN20693_c0_g2_i3 | -3.07 | 2.04 | 8.2E-12 | 1.8E-09 | ORR29_ORYSI | 1.13E-40 |
| DN18226_c0_g1_i5 | -9.56 | 0.46 | 8.8E-12 | 1.9E-09 | GPT2_ARATH | 7.94E-124 |
| DN16873_c0_g2_i14 | -2.73 | 3.02 | 9.5E-12 | 2.1E-09 | U83A1_ARATH | 4.39E-112 |
| DN21084_c0_g1_i26 | 11.11 | 2.94 | 1.1E-11 | 2.3E-09 | #N/A | #N/A |
| DN15625_c1_g1_i12 | 10.47 | 2.31 | 1.2E-11 | 2.5E-09 | APX8_ORYSJ | 1.76E-59 |
| DN20325_c1_g1_i14 | 10.73 | 2.56 | 1.2E-11 | 2.5E-09 | #N/A | #N/A |
| DN22526_c0_g2_i2 | 11.10 | 2.93 | 1.2E-11 | 2.5E-09 | #N/A | #N/A |
| DN21041_c0_g5_i1 | -9.36 | 0.26 | 1.2E-11 | 2.6E-09 | #N/A | #N/A |
| DN16418_c1_g2_i7 | 9.39 | 1.25 | 1.2E-11 | 2.6E-09 | ARFW_ORYSJ | 7.76E-147 |
| DN22123_c0_g4_i3 | -9.84 | 0.72 | 1.3E-11 | 2.7E-09 | #N/A | #N/A |
| DN21891_c1_g1_i6 | 3.26 | 3.96 | 1.3E-11 | 2.8E-09 | #N/A | #N/A |
| DN20960_c0_g1_i1 | 9.27 | 1.14 | 1.4E-11 | 3.0E-09 | #N/A | #N/A |
| DN16175_c0_g1_i19 | 9.53 | 1.39 | 1.7E-11 | 3.6E-09 | #N/A | #N/A |
| DN16837_c0_g2_i3 | -9.26 | 0.18 | 1.8E-11 | 3.7E-09 | RH15_ORYSJ | 1.23E-139 |
| DN20922_c1_g6_i1 | -9.65 | 0.55 | 1.8E-11 | 3.7E-09 | DPNPM_ARATH | 2.21E-24 |
| DN18295_c0_g1_i2 | 9.76 | 1.61 | 1.9E-11 | 3.9E-09 | COX11_ARATH | 9.1E-109 |
| DN14952_c0_g1_i9 | 9.46 | 1.32 | 1.9E-11 | 3.9E-09 | FDFT_ARATH | 5.84E-129 |
| DN19128_c0_g8_i2 | -9.60 | 0.49 | 1.9E-11 | 4.0E-09 | MIP3_ARATH | 0 |
| DN18418_c0_g1_i17 | 3.63 | 4.00 | 2.0E-11 | 4.2E-09 | **CHS2_MAIZE** | 0 |
| DN16156_c0_g1_i5 | 11.36 | 3.19 | 2.1E-11 | 4.4E-09 | P24B3_ARATH | 2.17E-83 |
| DN22123_c0_g4_i2 | -9.25 | 0.16 | 2.1E-11 | 4.4E-09 | #N/A | #N/A |
| DN16431_c0_g1_i35 | -1.67 | 6.39 | 2.3E-11 | 4.7E-09 | PGP1B_ARATH | 2.31E-161 |
| DN21604_c3_g1_i3 | -9.23 | 0.14 | 2.3E-11 | 4.7E-09 | NUD14_ARATH | 2.03E-110 |
| DN17931_c0_g3_i4 | 10.50 | 2.33 | 2.6E-11 | 5.2E-09 | COX6A_ARATH | 5.5E-32 |
| DN19859_c0_g1_i8 | 11.36 | 3.19 | 2.7E-11 | 5.4E-09 | #N/A | #N/A |
| DN22392_c0_g1_i20 | 10.60 | 2.43 | 2.7E-11 | 5.5E-09 | NLP2_ORYSJ | 0 |
| DN14175_c0_g1_i1 | -1.91 | 5.09 | 2.9E-11 | 5.7E-09 | #N/A | #N/A |
| DN18554_c0_g3_i1 | 9.19 | 4.19 | 3.0E-11 | 5.9E-09 | GCSH_ORYSJ | 2.2E-77 |
| DN15352_c0_g3_i18 | 10.97 | 5.95 | 3.1E-11 | 6.2E-09 | #N/A | #N/A |
| DN21785_c0_g2_i2 | 10.25 | 2.09 | 3.2E-11 | 6.3E-09 | WAK5_ARATH | 2.24E-132 |
| DN20069_c0_g1_i10 | -1.79 | 4.30 | 3.3E-11 | 6.5E-09 | VPS39_MOUSE | 4.66E-72 |
| DN21365_c0_g1_i9 | 10.95 | 2.78 | 3.5E-11 | 6.8E-09 | YLS3_ARATH | 4.2E-28 |
| DN17392_c0_g2_i1 | -1.66 | 6.51 | 3.5E-11 | 6.9E-09 | AGUA_ARATH | 9.84E-177 |
| DN22760_c1_g1_i6 | 9.14 | 1.01 | 3.8E-11 | 7.5E-09 | VIP2_NICBE | 1.86E-54 |
| DN22085_c0_g1_i9 | 9.27 | 1.14 | 3.9E-11 | 7.6E-09 | #N/A | #N/A |
| DN15625_c1_g1_i9 | 9.74 | 1.59 | 4.1E-11 | 8.1E-09 | APX8_ORYSJ | 0 |
| DN16926_c0_g2_i3 | 10.00 | 1.85 | 4.2E-11 | 8.1E-09 | BAT1_ORYSJ | 1.7E-137 |
| DN22762_c1_g2_i5 | -9.57 | 0.46 | 4.2E-11 | 8.1E-09 | #N/A | #N/A |
| DN18806_c1_g1_i9 | 6.95 | 1.18 | 4.3E-11 | 8.3E-09 | FDH1_ORYSJ | 2E-164 |
| DN20804_c0_g1_i5 | 9.32 | 1.19 | 4.4E-11 | 8.5E-09 | EGY1_ORYSJ | 3.1E-120 |
| DN14926_c0_g1_i1 | 12.73 | 4.54 | 4.6E-11 | 8.8E-09 | RLA1_MAIZE | 2.54E-37 |
| DN21180_c1_g4_i1 | -7.44 | 0.58 | 4.9E-11 | 9.3E-09 | #N/A | #N/A |
| DN19420_c2_g4_i8 | -9.21 | 0.13 | 5.0E-11 | 9.5E-09 | SCAM1_ORYSJ | 6.57E-69 |
| DN18529_c0_g1_i21 | -9.61 | 0.50 | 5.4E-11 | 1.0E-08 | NAS3_ORYSJ | 5.36E-36 |
| DN15175_c0_g2_i13 | 2.82 | 4.57 | 5.9E-11 | 1.1E-08 | DTX19_ARATH | 1.31E-145 |
| DN8983_c0_g1_i1 | 4.92 | 2.11 | 5.9E-11 | 1.1E-08 | #N/A | #N/A |
| DN19634_c0_g3_i9 | 6.02 | 4.33 | 6.0E-11 | 1.1E-08 | D27_ARATH | 8.05E-22 |
| DN19015_c0_g2_i6 | 10.30 | 2.14 | 6.6E-11 | 1.2E-08 | PDX11_ORYSJ | 0 |
| DN20017_c1_g1_i7 | 9.14 | 1.01 | 6.8E-11 | 1.3E-08 | Y3982_ORYSJ | 1.52E-47 |
| DN15838_c0_g1_i10 | 9.09 | 0.96 | 7.1E-11 | 1.3E-08 | ATG10_ARATH | 7.06E-28 |
| DN22653_c0_g1_i6 | 8.92 | 0.80 | 7.4E-11 | 1.4E-08 | #N/A | #N/A |
| DN22149_c0_g1_i8 | -9.18 | 0.10 | 8.3E-11 | 1.5E-08 | PGMC1_MAIZE | 0 |
| DN16213_c0_g1_i8 | 6.12 | 2.06 | 8.5E-11 | 1.6E-08 | ATG16_ARATH | 0 |
| DN17013_c2_g3_i4 | 3.65 | 2.75 | 8.6E-11 | 1.6E-08 | #N/A | #N/A |
| DN22572_c2_g3_i20 | -1.39 | 5.81 | 8.7E-11 | 1.6E-08 | #N/A | #N/A |
| DN18179_c0_g1_i6 | 10.43 | 2.27 | 9.0E-11 | 1.7E-08 | ARAD1_ARATH | 8.9E-80 |
| DN19206_c0_g1_i7 | -10.36 | 1.22 | 9.4E-11 | 1.7E-08 | PPA27_ARATH | 4.55E-102 |
| DN15028_c3_g3_i5 | 13.07 | 4.89 | 9.7E-11 | 1.8E-08 | EF1G2_ORYSJ | 6.35E-165 |
| DN22267_c0_g1_i14 | 9.32 | 1.19 | 9.8E-11 | 1.8E-08 | RH21_ORYSJ | 0 |
| DN15157_c0_g1_i2 | 12.83 | 4.65 | 1.0E-10 | 1.8E-08 | #N/A | #N/A |
| DN17144_c0_g2_i11 | 11.06 | 2.88 | 1.1E-10 | 1.9E-08 | #N/A | #N/A |
| DN22320_c0_g1_i23 | 10.58 | 2.41 | 1.1E-10 | 2.0E-08 | #N/A | #N/A |
| DN17903_c0_g2_i3 | 9.53 | 7.83 | 1.3E-10 | 2.3E-08 | RLA3_MAIZE | 1.02E-32 |
| DN21970_c1_g1_i2 | 11.75 | 3.58 | 1.3E-10 | 2.3E-08 | VDAC2_ORYSJ | 1.08E-145 |
| DN20777_c0_g2_i6 | 9.85 | 1.70 | 1.4E-10 | 2.5E-08 | YBEY_SYNPX | 9.62E-20 |
| DN16576_c0_g8_i1 | 13.18 | 4.99 | 1.4E-10 | 2.5E-08 | SYA_ARATH | 0 |
| DN21808_c2_g1_i17 | -2.29 | 5.58 | 1.5E-10 | 2.6E-08 | #N/A | #N/A |
| DN16870_c0_g2_i5 | 2.30 | 4.59 | 1.5E-10 | 2.7E-08 | BAN_ARATH | 1.77E-35 |
| DN21084_c0_g1_i4 | 10.75 | 2.59 | 1.5E-10 | 2.7E-08 | #N/A | #N/A |
| DN17148_c1_g2_i17 | 9.90 | 1.75 | 1.6E-10 | 2.8E-08 | #N/A | #N/A |
| DN22579_c1_g1_i16 | 10.73 | 2.57 | 1.6E-10 | 2.8E-08 | BRE1A_ORYSJ | 0 |
| DN17072_c1_g1_i3 | -7.47 | 0.71 | 1.6E-10 | 2.9E-08 | #N/A | #N/A |
| DN17272_c0_g1_i1 | 11.54 | 3.37 | 1.7E-10 | 3.0E-08 | ARFX_ORYSJ | 0 |
| DN22370_c1_g1_i16 | 10.09 | 1.93 | 1.8E-10 | 3.2E-08 | HOS1_ARATH | 0 |
| DN17160_c1_g4_i13 | -9.42 | 0.32 | 1.8E-10 | 3.2E-08 | #N/A | #N/A |
| DN15565_c0_g1_i2 | 9.26 | 1.13 | 1.9E-10 | 3.4E-08 | #N/A | #N/A |
| DN20772_c0_g2_i1 | 9.18 | 1.05 | 2.0E-10 | 3.4E-08 | TRN1_ARATH | 0 |
| DN10549_c0_g1_i1 | 9.19 | 1.06 | 2.0E-10 | 3.4E-08 | #N/A | #N/A |
| DN21288_c1_g1_i2 | -1.42 | 5.09 | 2.1E-10 | 3.6E-08 | #N/A | #N/A |
| DN17705_c0_g3_i5 | -9.17 | 0.09 | 2.1E-10 | 3.6E-08 | #N/A | #N/A |
| DN16151_c4_g2_i6 | -9.12 | 0.04 | 2.1E-10 | 3.6E-08 | NHX6_ARATH | 3.43E-95 |
| DN20802_c0_g3_i1 | 8.97 | 0.85 | 2.1E-10 | 3.7E-08 | #N/A | #N/A |
| DN15330_c0_g1_i1 | 12.23 | 4.05 | 2.2E-10 | 3.7E-08 | R13A4_ARATH | 1.89E-116 |
| DN19327_c0_g2_i4 | 10.93 | 2.76 | 2.2E-10 | 3.7E-08 | C3H33_ORYSJ | 0 |
| DN22320_c0_g1_i10 | 10.96 | 2.79 | 2.2E-10 | 3.7E-08 | #N/A | #N/A |
| DN22275_c0_g1_i2 | 9.19 | 1.06 | 2.5E-10 | 4.2E-08 | LOX4_ORYSJ | 0 |
| DN19937_c3_g1_i20 | 5.73 | 1.82 | 2.9E-10 | 4.8E-08 | #N/A | #N/A |
| DN22429_c0_g2_i4 | 12.37 | 4.19 | 2.9E-10 | 4.9E-08 | PUR4_ARATH | 0 |
| DN19937_c3_g1_i17 | 8.87 | 0.75 | 3.1E-10 | 5.2E-08 | THIC_ARATH | 0 |
| DN20777_c0_g2_i14 | 7.03 | 3.13 | 3.1E-10 | 5.2E-08 | YBEY_SYNPX | 4.54E-20 |
| DN18165_c3_g1_i8 | -9.46 | 0.36 | 3.3E-10 | 5.5E-08 | WRK46_HORVU | 0 |
| DN21874_c0_g1_i6 | 9.69 | 1.55 | 3.3E-10 | 5.5E-08 | XPF_ARATH | 0 |
| DN15580_c0_g1_i6 | 8.78 | 0.67 | 3.3E-10 | 5.5E-08 | #N/A | #N/A |
| DN19296_c0_g1_i1 | 9.06 | 0.93 | 3.4E-10 | 5.7E-08 | DEAHB_ARATH | 0 |
| DN19502_c1_g1_i12 | -2.54 | 2.58 | 3.6E-10 | 5.9E-08 | #N/A | #N/A |
| DN15381_c1_g3_i4 | -2.16 | 5.77 | 3.7E-10 | 6.0E-08 | CBSX3_ARATH | 3.19E-97 |
| DN16895_c0_g1_i1 | 1.99 | 4.56 | 3.7E-10 | 6.1E-08 | ENT3_ARATH | 6.04E-175 |
| DN22536_c0_g2_i3 | 5.94 | 1.52 | 3.8E-10 | 6.3E-08 | P2C43_ORYSJ | 7.1E-22 |
| DN16462_c0_g3_i4 | 7.88 | 3.78 | 3.9E-10 | 6.3E-08 | CYT12_ORYSJ | 3.47E-49 |
| DN21464_c0_g2_i14 | 9.81 | 1.66 | 3.9E-10 | 6.4E-08 | VPE1_PHAVU | 0 |
| DN20684_c1_g1_i25 | 9.56 | 1.41 | 4.0E-10 | 6.5E-08 | PPOC_ORYSJ | 0 |
| DN14752_c0_g2_i2 | 9.91 | 1.76 | 4.0E-10 | 6.5E-08 | DRIP2_ARATH | 1.19E-39 |
| DN18093_c0_g2_i11 | -9.58 | 0.47 | 4.1E-10 | 6.7E-08 | #N/A | #N/A |
| DN21643_c1_g2_i15 | 11.57 | 3.39 | 4.3E-10 | 6.9E-08 | #N/A | #N/A |
| DN18303_c0_g1_i2 | -9.08 | 0.01 | 4.5E-10 | 7.3E-08 | LORF2_HUMAN | 7.45E-39 |
| DN17382_c2_g2_i1 | -9.23 | 0.15 | 4.8E-10 | 7.8E-08 | FAX2_ARATH | 9.44E-21 |
| DN19322_c0_g3_i3 | 12.08 | 3.90 | 5.0E-10 | 8.0E-08 | MAD14_ORYSJ | 7.07E-80 |
| DN20831_c1_g3_i4 | -4.02 | 1.80 | 5.0E-10 | 8.1E-08 | DRG3_ARATH | 3.23E-123 |
| DN22700_c4_g3_i16 | 9.27 | 1.14 | 5.1E-10 | 8.1E-08 | HFA2E_ORYSJ | 6.09E-111 |
| DN20028_c0_g1_i8 | 11.60 | 3.42 | 5.2E-10 | 8.3E-08 | RT14_VICFA | 1.04E-42 |
| DN19658_c0_g1_i8 | 11.27 | 3.10 | 5.3E-10 | 8.4E-08 | RPK2_ARATH | 0 |
| DN17534_c0_g2_i5 | 10.34 | 2.18 | 5.3E-10 | 8.4E-08 | FHIT_BOVIN | 6.74E-29 |
| DN19718_c0_g1_i16 | -9.41 | 0.31 | 5.3E-10 | 8.4E-08 | PS13B_ARATH | 0 |
| DN22791_c0_g1_i4 | 10.13 | 1.97 | 5.4E-10 | 8.5E-08 | #N/A | #N/A |
| DN21199_c1_g2_i1 | 8.95 | 0.83 | 5.5E-10 | 8.6E-08 | #N/A | #N/A |
| DN22154_c0_g1_i18 | 4.22 | 1.66 | 5.8E-10 | 9.1E-08 | #N/A | #N/A |
| DN20709_c2_g3_i11 | -9.37 | 0.27 | 5.8E-10 | 9.1E-08 | RPM1_ARATH | 9.88E-26 |
| DN21387_c1_g1_i8 | 11.37 | 3.20 | 5.8E-10 | 9.1E-08 | SEBP2_ARATH | 1.59E-52 |
| DN20467_c1_g1_i19 | -9.25 | 0.16 | 6.3E-10 | 9.7E-08 | ERGI3_DANRE | 1.27E-70 |
| DN20002_c3_g1_i1 | 6.92 | 1.20 | 6.3E-10 | 9.8E-08 | PLSB_CUCSA | 0 |
| DN18226_c0_g1_i12 | 1.73 | 5.75 | 6.5E-10 | 1.0E-07 | GPT2_ARATH | 5.88E-162 |
| DN15848_c1_g1_i7 | 10.27 | 2.11 | 6.7E-10 | 1.0E-07 | NAC83_ARATH | 5.44E-47 |
| DN17707_c0_g1_i10 | 4.28 | 1.75 | 6.8E-10 | 1.0E-07 | PPD2_ARATH | 3.96E-30 |
| DN20302_c0_g1_i8 | 8.66 | 0.56 | 6.9E-10 | 1.1E-07 | GDI2_ARATH | 0 |
| DN16888_c0_g1_i6 | 9.17 | 1.04 | 7.0E-10 | 1.1E-07 | BPA1_ARATH | 2.38E-42 |
| DN19128_c0_g2_i6 | 8.78 | 0.67 | 7.1E-10 | 1.1E-07 | DRB2_ORYSJ | 1.37E-28 |
| DN18040_c3_g2_i2 | 11.28 | 3.11 | 7.1E-10 | 1.1E-07 | #N/A | #N/A |
| DN16473_c0_g1_i4 | 4.30 | 2.36 | 7.1E-10 | 1.1E-07 | YDJ7_SCHPO | 9.34E-63 |
| DN21923_c0_g2_i14 | 8.69 | 0.58 | 7.3E-10 | 1.1E-07 | #N/A | #N/A |
| DN17918_c1_g2_i4 | 5.97 | 2.87 | 7.4E-10 | 1.1E-07 | #N/A | #N/A |
| DN22636_c0_g1_i12 | 6.89 | 4.31 | 7.5E-10 | 1.1E-07 | SC31B_ARATH | 0 |
| DN20573_c2_g1_i15 | -9.09 | 0.01 | 7.5E-10 | 1.1E-07 | ZRAB3_MOUSE | 2.2E-105 |
| DN16324_c0_g1_i15 | 8.74 | 0.63 | 7.6E-10 | 1.1E-07 | DBR1_ORYSJ | 8.86E-122 |
| DN16319_c0_g2_i5 | -9.07 | 0.00 | 8.1E-10 | 1.2E-07 | #N/A | #N/A |
| DN19577_c0_g1_i5 | 8.66 | 0.56 | 8.6E-10 | 1.3E-07 | MDHP_ARATH | 0 |
| DN21608_c0_g1_i4 | 11.04 | 2.87 | 8.7E-10 | 1.3E-07 | EDM2_ARATH | 1.76E-81 |
| DN21229_c0_g2_i18 | 2.22 | 4.08 | 8.9E-10 | 1.3E-07 | Y1733_ARATH | 9.27E-52 |
| DN18370_c1_g1_i2 | -2.18 | 4.65 | 9.0E-10 | 1.3E-07 | PSAN_MAIZE | 5.44E-55 |
| DN22280_c1_g2_i8 | 9.14 | 1.01 | 9.0E-10 | 1.3E-07 | DNJH_CUCSA | 8.78E-28 |
| DN15625_c1_g1_i20 | 13.04 | 4.86 | 9.3E-10 | 1.4E-07 | APX8_ORYSJ | 0 |
| DN22553_c3_g2_i7 | 8.91 | 0.80 | 9.8E-10 | 1.4E-07 | P2C58_ORYSJ | 0 |
| DN21041_c0_g3_i4 | -9.04 | -0.03 | 1.1E-09 | 1.5E-07 | #N/A | #N/A |
| DN22458_c1_g1_i15 | 10.41 | 2.25 | 1.1E-09 | 1.6E-07 | #N/A | #N/A |
| DN17565_c2_g3_i2 | 8.63 | 0.53 | 1.1E-09 | 1.6E-07 | #N/A | #N/A |
| DN20820_c1_g1_i14 | 8.62 | 0.51 | 1.1E-09 | 1.6E-07 | PIN3A_ORYSJ | 4.94E-27 |
| DN18497_c0_g1_i3 | 9.42 | 1.28 | 1.3E-09 | 1.9E-07 | UGDH5_ORYSJ | 0 |
| DN19427_c0_g1_i9 | 9.65 | 1.50 | 1.3E-09 | 1.9E-07 | #N/A | #N/A |
| DN18873_c0_g2_i8 | 3.71 | 2.40 | 1.4E-09 | 2.1E-07 | #N/A | #N/A |
| DN19034_c0_g1_i6 | 9.13 | 1.00 | 1.5E-09 | 2.1E-07 | #N/A | #N/A |
| DN15735_c0_g2_i3 | -9.74 | 0.62 | 1.5E-09 | 2.2E-07 | #N/A | #N/A |
| DN15968_c2_g1_i2 | 5.62 | 3.02 | 1.5E-09 | 2.2E-07 | PHB3_ARATH | 9.53E-131 |
| DN18585_c0_g1_i5 | 9.00 | 0.88 | 1.5E-09 | 2.2E-07 | #N/A | #N/A |
| DN21717_c3_g2_i14 | 11.98 | 3.80 | 1.6E-09 | 2.2E-07 | PIP_ARATH | 0 |
| DN21106_c0_g5_i14 | 9.00 | 0.88 | 1.6E-09 | 2.2E-07 | IF1A_WHEAT | 1.02E-61 |
| DN18125_c1_g5_i3 | 7.96 | 4.38 | 1.6E-09 | 2.2E-07 | ROC1_SPIOL | 8.82E-28 |
| DN16412_c0_g5_i4 | 10.83 | 2.66 | 1.6E-09 | 2.3E-07 | CP20C_ARATH | 1.31E-96 |
| DN20655_c0_g3_i14 | 8.81 | 0.70 | 1.6E-09 | 2.3E-07 | #N/A | #N/A |
| DN21355_c1_g2_i2 | 9.70 | 1.55 | 1.6E-09 | 2.3E-07 | ITPK1_ORYSI | 1.35E-80 |
| DN18507_c1_g3_i12 | -9.01 | -0.06 | 1.7E-09 | 2.4E-07 | QKY_ARATH | 5.88E-23 |
| DN22079_c0_g1_i12 | 8.99 | 0.87 | 1.8E-09 | 2.5E-07 | PIF3_ARATH | 1.16E-31 |
| DN16149_c1_g2_i2 | 9.15 | 1.02 | 1.8E-09 | 2.6E-07 | PFD4_AVEFA | 4.36E-65 |
| DN14365_c0_g1_i6 | 7.21 | 5.33 | 1.9E-09 | 2.6E-07 | TRXM_MAIZE | 6.19E-84 |
| DN15352_c0_g3_i1 | 6.21 | 5.56 | 2.0E-09 | 2.8E-07 | #N/A | #N/A |
| DN21205_c1_g4_i1 | 8.86 | 0.74 | 2.1E-09 | 3.0E-07 | GOGC2_ARATH | 5.78E-30 |
| DN18645_c0_g1_i11 | -9.00 | -0.07 | 2.3E-09 | 3.2E-07 | YPOL_THETH | 2.06E-21 |
| DN19502_c1_g1_i19 | 1.34 | 6.45 | 2.3E-09 | 3.3E-07 | #N/A | #N/A |
| DN21891_c1_g1_i2 | -1.54 | 6.52 | 2.4E-09 | 3.3E-07 | #N/A | #N/A |
| DN20506_c0_g3_i5 | 11.27 | 3.10 | 2.4E-09 | 3.3E-07 | FTSH1_ORYSJ | 0 |
| DN16340_c3_g1_i1 | 8.39 | 2.58 | 2.4E-09 | 3.4E-07 | RS51_ARATH | 2.16E-92 |
| DN21010_c0_g1_i25 | -8.95 | -0.12 | 2.5E-09 | 3.4E-07 | E131_ARATH | 3.77E-159 |
| DN15886_c2_g1_i1 | -3.68 | 1.92 | 2.5E-09 | 3.4E-07 | PIP12_MAIZE | 6.51E-117 |
| DN18156_c1_g2_i10 | 12.10 | 3.92 | 2.6E-09 | 3.6E-07 | ACR9_ARATH | 1.88E-156 |
| DN21072_c2_g1_i11 | 9.02 | 0.89 | 2.6E-09 | 3.6E-07 | DUS1_ARATH | 6.5E-30 |
| DN15065_c0_g3_i2 | 9.69 | 1.55 | 2.7E-09 | 3.7E-07 | AKR1_SOYBN | 8.69E-158 |
| DN17072_c1_g1_i7 | 8.64 | 0.53 | 2.7E-09 | 3.7E-07 | #N/A | #N/A |
| DN20061_c0_g1_i12 | 10.91 | 2.74 | 2.8E-09 | 3.8E-07 | TERC_ARATH | 8.67E-92 |
| DN18697_c1_g1_i1 | 10.51 | 2.34 | 2.8E-09 | 3.8E-07 | #N/A | #N/A |
| DN19180_c0_g2_i6 | 11.02 | 2.85 | 2.8E-09 | 3.9E-07 | #N/A | #N/A |
| DN22085_c0_g1_i1 | 8.58 | 0.48 | 2.9E-09 | 3.9E-07 | #N/A | #N/A |
| DN22536_c0_g2_i4 | 9.19 | 1.07 | 3.1E-09 | 4.2E-07 | P2C43_ORYSJ | 3.9E-21 |
| DN13894_c0_g1_i3 | 11.52 | 3.34 | 3.4E-09 | 4.6E-07 | ANM10_ORYSJ | 0 |
| DN20051_c0_g1_i5 | -9.73 | 0.61 | 3.4E-09 | 4.6E-07 | MDHM_EUCGU | 1.94E-120 |
| DN21431_c0_g1_i15 | -8.92 | -0.14 | 3.5E-09 | 4.7E-07 | Y1534_ARATH | 1.7E-89 |
| DN14322_c0_g1_i6 | 10.94 | 2.77 | 3.6E-09 | 4.8E-07 | UEV1A_ARATH | 5.76E-74 |
| DN17832_c0_g1_i3 | -8.92 | -0.14 | 3.6E-09 | 4.8E-07 | FUCO1_ARATH | 2.45E-145 |
| DN20684_c1_g1_i8 | 9.98 | 1.83 | 3.7E-09 | 4.9E-07 | PPOC_ORYSJ | 0 |
| DN19767_c0_g1_i14 | 10.72 | 2.56 | 3.7E-09 | 5.0E-07 | IPYR4_ARATH | 3.95E-101 |
| DN17314_c0_g3_i1 | 8.92 | 0.80 | 3.8E-09 | 5.1E-07 | #N/A | #N/A |
| DN16569_c0_g1_i12 | 9.05 | 0.93 | 3.8E-09 | 5.1E-07 | CD48B_ARATH | 1.12E-128 |
| DN19507_c0_g1_i6 | 10.60 | 2.44 | 3.9E-09 | 5.1E-07 | MED27_ARATH | 1.27E-140 |
| DN18031_c0_g5_i4 | 9.64 | 1.50 | 3.9E-09 | 5.2E-07 | PSB7A_ARATH | 1.15E-167 |
| DN14321_c0_g1_i1 | 9.84 | 1.69 | 4.1E-09 | 5.4E-07 | PB27B_ARATH | 6.97E-48 |
| DN17617_c1_g2_i5 | -7.56 | 0.07 | 4.4E-09 | 5.7E-07 | #N/A | #N/A |
| DN19382_c1_g1_i3 | -1.85 | 3.58 | 4.5E-09 | 5.9E-07 | #N/A | #N/A |
| DN19667_c1_g1_i14 | 8.86 | 0.75 | 4.5E-09 | 5.9E-07 | WTR24_ARATH | 8.99E-65 |
| DN19383_c0_g2_i1 | 8.44 | 0.35 | 4.8E-09 | 6.2E-07 | RR9_SPIOL | 9.53E-40 |
| DN19672_c0_g3_i4 | 9.36 | 1.22 | 4.9E-09 | 6.4E-07 | 2A5G_ARATH | 0 |
| DN20660_c0_g4_i3 | -2.40 | 5.28 | 5.1E-09 | 6.7E-07 | #N/A | #N/A |
| DN15330_c0_g3_i2 | 5.28 | 4.73 | 5.2E-09 | 6.8E-07 | R13A4_ARATH | 4.51E-115 |
| DN15553_c0_g4_i3 | 9.59 | 1.45 | 5.4E-09 | 7.0E-07 | DPNP_ORYSJ | 0 |
| DN18507_c1_g3_i1 | -8.92 | -0.14 | 5.4E-09 | 7.0E-07 | QKY_ARATH | 4.28E-24 |
| DN14150_c0_g1_i9 | 8.53 | 0.43 | 5.5E-09 | 7.1E-07 | #N/A | #N/A |
| DN13483_c0_g1_i5 | -8.89 | -0.17 | 5.8E-09 | 7.4E-07 | NNJA5_MAIZE | 1.2E-61 |
| DN16126_c0_g1_i1 | 8.94 | 0.82 | 6.1E-09 | 7.9E-07 | RSC5_DICDI | 1.47E-26 |
| DN18370_c1_g1_i9 | 8.71 | 2.88 | 6.4E-09 | 8.2E-07 | PSAN_MAIZE | 6.91E-55 |
| DN13597_c0_g1_i1 | 8.52 | 0.42 | 6.4E-09 | 8.2E-07 | PUR4_ARATH | 6.16E-41 |
| DN16358_c4_g2_i3 | 9.81 | 1.66 | 6.8E-09 | 8.7E-07 | MLO5_ARATH | 0 |
| DN15601_c1_g4_i2 | 8.92 | 0.81 | 6.9E-09 | 8.8E-07 | #N/A | #N/A |
| DN21417_c2_g1_i1 | -8.87 | -0.18 | 7.0E-09 | 8.9E-07 | #N/A | #N/A |
| DN20885_c0_g1_i12 | 10.98 | 2.81 | 7.2E-09 | 9.1E-07 | #N/A | #N/A |
| DN16904_c0_g1_i7 | 10.05 | 1.89 | 7.2E-09 | 9.2E-07 | RL30_MAIZE | 1.01E-76 |
| DN22104_c0_g4_i4 | 9.04 | 0.92 | 7.5E-09 | 9.5E-07 | #N/A | #N/A |
| DN18376_c1_g2_i10 | 12.21 | 4.03 | 7.7E-09 | 9.7E-07 | 5NG4_PINTA | 4.16E-85 |
| DN22491_c1_g2_i3 | 7.92 | 4.55 | 7.7E-09 | 9.8E-07 | Y1669_ARATH | 5.21E-105 |
| DN18761_c0_g1_i12 | -1.76 | 4.27 | 7.8E-09 | 9.8E-07 | #N/A | #N/A |
| DN14960_c0_g1_i1 | 9.19 | 1.06 | 8.8E-09 | 1.1E-06 | AB19B_ARATH | 0 |
| DN15313_c2_g1_i5 | -3.14 | 3.22 | 9.2E-09 | 1.2E-06 | ISCAP_ARATH | 8.54E-61 |
| DN21096_c0_g5_i3 | 9.98 | 1.82 | 9.2E-09 | 1.2E-06 | ADO1_ORYSJ | 0 |
| DN21254_c0_g1_i2 | -8.85 | -0.21 | 9.8E-09 | 1.2E-06 | SCYL2_HUMAN | 1.34E-46 |
| DN22057_c1_g2_i1 | 5.07 | 1.78 | 1.0E-08 | 1.3E-06 | #N/A | #N/A |
| DN18939_c0_g1_i14 | 8.44 | 0.35 | 1.1E-08 | 1.4E-06 | AK1_ARATH | 4.36E-113 |
| DN21274_c1_g2_i8 | 8.87 | 0.76 | 1.1E-08 | 1.4E-06 | #N/A | #N/A |
| DN22082_c0_g1_i2 | 11.39 | 3.22 | 1.2E-08 | 1.4E-06 | #N/A | #N/A |
| DN18760_c1_g1_i2 | 8.83 | 0.72 | 1.2E-08 | 1.5E-06 | #N/A | #N/A |
| DN14345_c0_g1_i2 | 11.52 | 3.35 | 1.3E-08 | 1.5E-06 | #N/A | #N/A |
| DN17019_c1_g2_i1 | -2.62 | 2.07 | 1.3E-08 | 1.6E-06 | FLP4_ORYSJ | 2.28E-37 |
| DN19402_c0_g2_i3 | 9.82 | 1.67 | 1.3E-08 | 1.6E-06 | #N/A | #N/A |
| DN16684_c0_g1_i6 | 8.69 | 0.59 | 1.3E-08 | 1.6E-06 | MBD4L_ARATH | 2.77E-39 |
| DN18143_c0_g2_i6 | 9.14 | 1.02 | 1.3E-08 | 1.6E-06 | #N/A | #N/A |
| DN21331_c0_g1_i13 | 10.23 | 2.07 | 1.3E-08 | 1.6E-06 | AB12A_ARATH | 0 |
| DN18075_c4_g1_i4 | -9.47 | 0.36 | 1.4E-08 | 1.7E-06 | RK18_ORYSJ | 1.65E-50 |
| DN20066_c0_g3_i1 | 10.09 | 1.93 | 1.4E-08 | 1.7E-06 | AKRCA_ARATH | 1.77E-142 |
| DN22422_c0_g1_i8 | 8.28 | 0.20 | 1.4E-08 | 1.7E-06 | FIPS5_ARATH | 4.48E-58 |
| DN21993_c0_g1_i21 | 10.06 | 1.91 | 1.4E-08 | 1.7E-06 | #N/A | #N/A |
| DN17946_c1_g1_i6 | 9.88 | 1.73 | 1.4E-08 | 1.7E-06 | UTP7_SCHPO | 4.13E-102 |
| DN16081_c0_g2_i1 | -1.75 | 4.53 | 1.5E-08 | 1.8E-06 | C3H54_ORYSJ | 7.24E-101 |
| DN19431_c1_g2_i12 | 9.02 | 0.90 | 1.5E-08 | 1.8E-06 | #N/A | #N/A |
| DN21309_c1_g1_i20 | -8.84 | -0.21 | 1.5E-08 | 1.8E-06 | SMG8_BOVIN | 8.93E-22 |
| DN16505_c0_g4_i2 | 8.54 | 0.44 | 1.6E-08 | 1.9E-06 | #N/A | #N/A |
| DN18441_c0_g1_i10 | -7.13 | 1.05 | 1.7E-08 | 2.0E-06 | KMS1_ARATH | 3.25E-156 |
| DN20110_c0_g1_i20 | 8.29 | 0.21 | 1.7E-08 | 2.0E-06 | TTC4_DICDI | 3.73E-27 |
| DN14807_c0_g6_i1 | 8.64 | 0.54 | 1.7E-08 | 2.1E-06 | LIAS_MAIZE | 3.01E-21 |
| DN17408_c0_g2_i11 | 8.74 | 0.63 | 1.8E-08 | 2.1E-06 | #N/A | #N/A |
| DN21034_c1_g1_i5 | 9.74 | 1.59 | 1.9E-08 | 2.2E-06 | POLIB_ORYSJ | 5.69E-29 |
| DN16210_c0_g4_i7 | -7.63 | 5.87 | 1.9E-08 | 2.2E-06 | #N/A | #N/A |
| DN9072_c0_g1_i2 | 9.00 | 0.88 | 1.9E-08 | 2.2E-06 | **URT1_FRAAN** | 1.06E-45 |
| DN19672_c0_g3_i11 | 10.64 | 2.48 | 1.9E-08 | 2.2E-06 | 2A5G_ARATH | 0 |
| DN21008_c0_g1_i9 | -1.43 | 7.08 | 1.9E-08 | 2.3E-06 | ZEP_ORYSJ | 0 |
| DN21210_c0_g1_i7 | 1.68 | 4.32 | 2.0E-08 | 2.3E-06 | IFRD1_MOUSE | 8.82E-26 |
| DN19080_c1_g1_i4 | 8.60 | 0.50 | 2.0E-08 | 2.4E-06 | #N/A | #N/A |
| DN19970_c0_g3_i9 | 6.02 | 1.18 | 2.0E-08 | 2.4E-06 | RRF_BACHD | 9.81E-33 |
| DN16099_c2_g1_i4 | 8.38 | 0.29 | 2.1E-08 | 2.5E-06 | #N/A | #N/A |
| DN14970_c0_g4_i2 | -1.86 | 6.93 | 2.1E-08 | 2.5E-06 | #N/A | #N/A |
| DN20475_c0_g2_i3 | 8.46 | 0.37 | 2.1E-08 | 2.5E-06 | RRP3_HORVU | 7.96E-54 |
| DN14731_c0_g8_i3 | 12.09 | 3.91 | 2.1E-08 | 2.5E-06 | CHMP5_DICDI | 1.89E-57 |
| DN16820_c1_g2_i2 | 11.44 | 3.26 | 2.2E-08 | 2.5E-06 | PER51_ARATH | 7.48E-98 |
| DN20126_c0_g1_i14 | 9.05 | 0.93 | 2.2E-08 | 2.5E-06 | COB21_ORYSJ | 2.87E-54 |
| DN19035_c1_g2_i14 | 9.11 | 0.99 | 2.2E-08 | 2.5E-06 | Y4230_ARATH | 1.3E-22 |
| DN17104_c0_g2_i15 | -8.84 | -0.21 | 2.2E-08 | 2.6E-06 | #N/A | #N/A |
| DN15276_c2_g2_i7 | 11.59 | 3.42 | 2.4E-08 | 2.8E-06 | MPPA_SOLTU | 0 |
| DN15593_c1_g3_i8 | -9.39 | 0.29 | 2.4E-08 | 2.8E-06 | #N/A | #N/A |
| DN17090_c0_g2_i12 | -1.90 | 3.05 | 2.4E-08 | 2.8E-06 | CATB_PONAB | 1.74E-61 |
| DN14234_c0_g1_i3 | 8.31 | 0.23 | 2.5E-08 | 2.8E-06 | #N/A | #N/A |
| DN19331_c0_g2_i6 | 11.55 | 3.37 | 2.5E-08 | 2.8E-06 | #N/A | #N/A |
| DN16386_c0_g2_i1 | 7.93 | 3.44 | 2.5E-08 | 2.9E-06 | RS29_WHEAT | 3.06E-30 |
| DN22079_c0_g1_i14 | 8.77 | 0.66 | 2.6E-08 | 3.0E-06 | PIF3_ARATH | 1.69E-31 |
| DN18118_c0_g3_i11 | 9.68 | 1.53 | 2.8E-08 | 3.2E-06 | IQD31_ARATH | 1.6E-87 |
| DN20850_c1_g1_i1 | 10.67 | 2.50 | 2.8E-08 | 3.2E-06 | #N/A | #N/A |
| DN21529_c0_g1_i2 | 10.13 | 1.98 | 2.8E-08 | 3.2E-06 | CIR1A_MOUSE | 1.82E-40 |
| DN16400_c0_g1_i6 | 8.74 | 3.89 | 3.0E-08 | 3.4E-06 | H2AV3_ORYSJ | 3.58E-76 |
| DN13709_c0_g1_i4 | -8.76 | -0.29 | 3.1E-08 | 3.4E-06 | #N/A | #N/A |
| DN16797_c0_g2_i4 | -8.76 | -0.29 | 3.1E-08 | 3.4E-06 | #N/A | #N/A |
| DN17535_c0_g2_i12 | 8.24 | 0.16 | 3.1E-08 | 3.5E-06 | #N/A | #N/A |
| DN20804_c0_g1_i3 | 8.58 | 0.48 | 3.2E-08 | 3.6E-06 | EGY1_ORYSJ | 5.23E-172 |
| DN17241_c0_g2_i4 | 8.43 | 0.34 | 3.2E-08 | 3.6E-06 | #N/A | #N/A |
| DN18595_c0_g1_i8 | 8.97 | 0.85 | 3.2E-08 | 3.6E-06 | #N/A | #N/A |
| DN18848_c1_g1_i18 | 10.49 | 2.33 | 3.3E-08 | 3.7E-06 | #N/A | #N/A |
| DN19309_c0_g1_i10 | 10.69 | 2.52 | 3.3E-08 | 3.7E-06 | #N/A | #N/A |
| DN21198_c1_g5_i1 | 8.58 | 3.73 | 3.4E-08 | 3.8E-06 | #N/A | #N/A |
| DN21570_c1_g1_i17 | 6.83 | 1.06 | 3.5E-08 | 3.9E-06 | #N/A | #N/A |
| DN20453_c0_g1_i1 | -9.03 | -0.04 | 3.6E-08 | 4.0E-06 | NOG1_ARATH | 0 |
| DN20298_c4_g4_i1 | 9.98 | 1.83 | 3.7E-08 | 4.1E-06 | RH14_ORYSJ | 3.55E-28 |
| DN19070_c0_g2_i5 | 8.40 | 0.31 | 3.8E-08 | 4.2E-06 | #N/A | #N/A |
| DN22678_c1_g1_i8 | 10.16 | 2.00 | 4.0E-08 | 4.4E-06 | CLASP_ARATH | 0 |
| DN15205_c0_g7_i2 | 8.26 | 0.18 | 4.0E-08 | 4.4E-06 | CSCLC_ARATH | 2.74E-171 |
| DN18376_c1_g2_i7 | 6.95 | 2.85 | 4.1E-08 | 4.5E-06 | 5NG4_PINTA | 7.35E-45 |
| DN15825_c1_g2_i1 | 9.64 | 1.49 | 4.1E-08 | 4.5E-06 | TRPC_ARATH | 2.51E-159 |
| DN20192_c0_g1_i10 | -10.56 | 1.41 | 4.1E-08 | 4.5E-06 | RBM39_PONAB | 1.94E-53 |
| DN22700_c4_g3_i11 | -2.40 | 4.36 | 4.3E-08 | 4.7E-06 | HFA2E_ORYSJ | 6.93E-112 |
| DN18826_c0_g1_i11 | 9.57 | 1.43 | 4.3E-08 | 4.7E-06 | XB33_ORYSJ | 0 |
| DN21072_c2_g1_i4 | -1.68 | 4.53 | 4.3E-08 | 4.7E-06 | DUS1_ARATH | 6.65E-33 |
| DN22375_c1_g1_i5 | -8.70 | -0.34 | 4.5E-08 | 4.9E-06 | UFC_ARATH | 4.89E-26 |
| DN19226_c1_g3_i9 | 7.66 | 3.24 | 4.6E-08 | 5.0E-06 | HT1_ARATH | 9.29E-53 |
| DN21131_c0_g1_i11 | 10.01 | 1.86 | 4.7E-08 | 5.1E-06 | #N/A | #N/A |
| DN18608_c1_g1_i8 | 8.37 | 0.28 | 4.9E-08 | 5.3E-06 | IP5P7_ARATH | 3.91E-37 |
| DN18418_c0_g1_i14 | 2.81 | 3.85 | 5.0E-08 | 5.4E-06 | **CHS2_MAIZE** | 0 |
| DN20721_c1_g1_i21 | -2.61 | 2.28 | 5.0E-08 | 5.4E-06 | #N/A | #N/A |
| DN21434_c0_g1_i5 | -2.07 | 8.90 | 5.1E-08 | 5.5E-06 | #N/A | #N/A |
| DN20497_c1_g2_i6 | 10.58 | 2.42 | 5.2E-08 | 5.6E-06 | RMR1_ORYSJ | 0 |
| DN18761_c0_g1_i9 | -1.81 | 3.46 | 5.3E-08 | 5.6E-06 | #N/A | #N/A |
| DN21434_c0_g1_i7 | -1.73 | 8.76 | 5.3E-08 | 5.6E-06 | #N/A | #N/A |
| DN17781_c3_g1_i2 | 8.37 | 0.28 | 5.4E-08 | 5.8E-06 | #N/A | #N/A |
| DN18758_c0_g2_i6 | 9.31 | 1.17 | 5.5E-08 | 5.9E-06 | #N/A | #N/A |
| DN22154_c0_g1_i10 | 8.80 | 0.68 | 5.6E-08 | 6.0E-06 | #N/A | #N/A |
| DN20777_c0_g2_i1 | 10.30 | 2.14 | 5.6E-08 | 6.0E-06 | YBEY_SYNPX | 3.75E-20 |
| DN15492_c0_g1_i5 | 10.58 | 2.41 | 5.7E-08 | 6.1E-06 | HEXO1_ARATH | 0 |
| DN16401_c0_g5_i1 | -2.28 | 3.59 | 5.7E-08 | 6.1E-06 | #N/A | #N/A |
| DN16817_c0_g5_i6 | 11.08 | 2.91 | 5.8E-08 | 6.1E-06 | CP41B_ARATH | 9.57E-174 |
| DN16214_c1_g4_i2 | 8.33 | 0.24 | 5.8E-08 | 6.1E-06 | IDH2_ARATH | 6.18E-41 |
| DN15899_c0_g2_i1 | 10.66 | 2.49 | 5.9E-08 | 6.2E-06 | C81E1_GLYEC | 3.02E-43 |
| DN19937_c3_g1_i7 | 9.10 | 0.97 | 5.9E-08 | 6.2E-06 | THIC_ARATH | 0 |
| DN19846_c1_g1_i4 | 9.62 | 1.47 | 6.0E-08 | 6.3E-06 | VP13E_DICDI | 1.96E-37 |
| DN21735_c1_g1_i5 | -9.15 | 0.06 | 6.0E-08 | 6.3E-06 | #N/A | #N/A |
| DN14414_c0_g1_i3 | 9.98 | 1.82 | 6.2E-08 | 6.5E-06 | #N/A | #N/A |
| DN19544_c1_g2_i1 | -1.56 | 4.01 | 6.3E-08 | 6.6E-06 | #N/A | #N/A |
| DN18052_c0_g1_i7 | 11.97 | 3.79 | 6.3E-08 | 6.6E-06 | ALFC_ORYSJ | 4.25E-86 |
| DN22313_c0_g1_i6 | -8.65 | -0.39 | 6.4E-08 | 6.6E-06 | RNP1_ARATH | 3.69E-69 |
| DN15876_c0_g1_i8 | 8.54 | 0.45 | 6.6E-08 | 6.9E-06 | AAP7_ARATH | 7.72E-32 |
| DN19586_c3_g1_i8 | 5.37 | 2.29 | 6.6E-08 | 6.9E-06 | EML3_ARATH | 1.67E-78 |
| DN19233_c0_g1_i2 | 10.66 | 2.49 | 6.6E-08 | 6.9E-06 | HFA2C_ORYSJ | 6.81E-109 |
| DN14155_c0_g1_i5 | -8.68 | -0.36 | 6.7E-08 | 6.9E-06 | C16B1_PICSI | 2.55E-106 |
| DN20960_c0_g1_i3 | 5.75 | 0.83 | 6.7E-08 | 7.0E-06 | #N/A | #N/A |
| DN16241_c4_g1_i3 | 7.95 | 2.14 | 6.7E-08 | 7.0E-06 | #N/A | #N/A |
| DN18055_c0_g1_i8 | 9.45 | 1.31 | 6.9E-08 | 7.1E-06 | #N/A | #N/A |
| DN16873_c0_g2_i15 | 8.93 | 0.82 | 7.0E-08 | 7.2E-06 | U83A1_ARATH | 1.48E-64 |
| DN15428_c0_g1_i6 | 10.61 | 2.44 | 7.1E-08 | 7.3E-06 | PMP22_ARATH | 1.71E-39 |
| DN20927_c0_g1_i11 | 7.98 | 2.98 | 7.2E-08 | 7.4E-06 | GT644_ARATH | 1.46E-100 |
| DN21848_c0_g1_i10 | 11.19 | 3.02 | 7.5E-08 | 7.7E-06 | SND1_RAT | 1.84E-147 |
| DN19943_c0_g1_i10 | 8.86 | 0.75 | 7.8E-08 | 8.0E-06 | #N/A | #N/A |
| DN14997_c0_g4_i1 | 10.27 | 2.11 | 7.8E-08 | 8.0E-06 | RS23_FRAAN | 5.14E-96 |
| DN18311_c0_g2_i10 | 8.52 | 0.43 | 7.9E-08 | 8.1E-06 | ART1_ORYSJ | 1.43E-127 |
| DN18831_c0_g1_i12 | 8.88 | 0.76 | 8.0E-08 | 8.1E-06 | CRD1_HORVU | 1.36E-167 |
| DN15015_c0_g1_i3 | -1.24 | 5.01 | 8.0E-08 | 8.1E-06 | #N/A | #N/A |
| DN16664_c3_g3_i16 | -8.66 | -0.38 | 8.2E-08 | 8.3E-06 | NADE_ORYSJ | 0 |
| DN18222_c0_g1_i2 | 9.96 | 1.81 | 8.2E-08 | 8.4E-06 | MPK12_ORYSJ | 0 |
| DN20228_c0_g2_i15 | -2.77 | 1.77 | 8.5E-08 | 8.6E-06 | AVP_VIGRR | 0 |
| DN18776_c0_g1_i8 | 5.45 | 2.85 | 8.5E-08 | 8.6E-06 | GCP5_MACFA | 3.93E-31 |
| DN22392_c0_g1_i6 | 11.31 | 3.14 | 8.5E-08 | 8.6E-06 | NLP2_ORYSJ | 0 |
| DN16567_c0_g2_i7 | -8.79 | -0.25 | 8.6E-08 | 8.7E-06 | SRP68_CANLF | 4.99E-68 |
| DN22275_c0_g1_i11 | 4.47 | 2.18 | 8.7E-08 | 8.7E-06 | LOX4_ORYSJ | 0 |
| DN18760_c1_g1_i4 | 8.26 | 0.18 | 8.7E-08 | 8.8E-06 | #N/A | #N/A |
| DN17220_c0_g2_i5 | -8.61 | -0.42 | 8.9E-08 | 8.9E-06 | ALFL9_ORYSI | 4.74E-71 |
| DN19635_c0_g2_i8 | 4.56 | 5.93 | 8.9E-08 | 8.9E-06 | KADC_MAIZE | 4.51E-123 |
| DN17034_c0_g1_i2 | 10.20 | 2.04 | 8.9E-08 | 8.9E-06 | #N/A | #N/A |
| DN16104_c1_g2_i9 | 10.48 | 2.32 | 9.1E-08 | 9.1E-06 | #N/A | #N/A |
| DN15407_c0_g1_i8 | 8.22 | 0.15 | 9.6E-08 | 9.5E-06 | CML11_ORYSJ | 3.63E-70 |
| DN22104_c0_g2_i1 | 8.41 | 0.32 | 9.8E-08 | 9.8E-06 | #N/A | #N/A |
| DN16412_c0_g5_i5 | 9.68 | 1.54 | 9.9E-08 | 9.8E-06 | CP20C_ARATH | 3.81E-96 |
| DN15449_c0_g2_i3 | -8.69 | -0.34 | 9.9E-08 | 9.9E-06 | WNK4_ARATH | 9.43E-119 |
| DN18609_c0_g4_i6 | 9.77 | 1.62 | 1.0E-07 | 9.9E-06 | #N/A | #N/A |
| DN21023_c2_g1_i7 | 8.93 | 0.81 | 1.0E-07 | 9.9E-06 | #N/A | #N/A |
| DN20664_c0_g1_i4 | 9.42 | 1.28 | 1.0E-07 | 1.0E-05 | ASPR1_ORYSJ | 0 |
| DN18806_c1_g1_i8 | 8.33 | 0.24 | 1.0E-07 | 1.0E-05 | FDH1_ORYSJ | 0 |
| DN17621_c1_g2_i2 | 10.25 | 2.09 | 1.0E-07 | 1.0E-05 | RAR1_ORYSJ | 2.77E-119 |
| DN22085_c0_g1_i6 | 9.70 | 1.55 | 1.0E-07 | 1.0E-05 | #N/A | #N/A |
| DN17764_c1_g1_i13 | 8.45 | 2.63 | 1.0E-07 | 1.0E-05 | VATL_ORYSJ | 8.47E-61 |
| DN19870_c0_g1_i6 | 12.15 | 3.97 | 1.0E-07 | 1.0E-05 | GGT2_ARATH | 0 |
| DN16959_c0_g3_i2 | 4.49 | 2.77 | 1.0E-07 | 1.0E-05 | LHT2_ARATH | 4.68E-60 |
| DN15829_c2_g1_i7 | -8.62 | -0.40 | 1.0E-07 | 1.0E-05 | #N/A | #N/A |
| DN15683_c3_g4_i1 | 9.12 | 0.99 | 1.1E-07 | 1.0E-05 | MYBP_MAIZE | 2.95E-44 |
| DN16401_c0_g1_i5 | 9.13 | 1.00 | 1.1E-07 | 1.1E-05 | #N/A | #N/A |
| DN19984_c0_g1_i1 | -7.01 | 1.95 | 1.1E-07 | 1.1E-05 | EF1A_MANES | 6.24E-111 |
| DN22526_c0_g1_i2 | 7.69 | 2.70 | 1.1E-07 | 1.1E-05 | #N/A | #N/A |
| DN17960_c1_g4_i7 | 8.30 | 0.21 | 1.2E-07 | 1.1E-05 | #N/A | #N/A |
| DN17489_c1_g1_i16 | -8.60 | -0.43 | 1.2E-07 | 1.1E-05 | APEH_MOUSE | 8.64E-58 |
| DN22057_c1_g2_i3 | -2.69 | 1.53 | 1.2E-07 | 1.1E-05 | #N/A | #N/A |
| DN16909_c1_g5_i1 | -1.28 | 7.20 | 1.2E-07 | 1.2E-05 | MYB59_ARATH | 1.76E-28 |
| DN18295_c1_g1_i4 | -8.58 | -0.44 | 1.2E-07 | 1.2E-05 | #N/A | #N/A |
| DN16097_c0_g2_i13 | -9.10 | 0.03 | 1.2E-07 | 1.2E-05 | SUI1_MAIZE | 4.41E-39 |
| DN20149_c0_g1_i11 | -1.41 | 4.85 | 1.2E-07 | 1.2E-05 | GOLS2_SOLLC | 1.94E-115 |
| DN19035_c1_g2_i29 | 3.30 | 1.85 | 1.2E-07 | 1.2E-05 | CRK19_ARATH | 1.92E-29 |
| DN19270_c1_g1_i5 | 8.23 | 0.16 | 1.3E-07 | 1.2E-05 | BH074_ARATH | 2.02E-48 |
| DN19882_c0_g3_i1 | 8.38 | 0.29 | 1.3E-07 | 1.2E-05 | #N/A | #N/A |
| DN19404_c0_g1_i16 | 9.84 | 1.69 | 1.3E-07 | 1.2E-05 | #N/A | #N/A |
| DN17078_c0_g1_i1 | 8.08 | 0.01 | 1.3E-07 | 1.3E-05 | LEC_ONOVI | 3.53E-30 |
| DN21305_c0_g2_i6 | 10.30 | 2.14 | 1.3E-07 | 1.3E-05 | #N/A | #N/A |
| DN18241_c2_g7_i6 | 8.37 | 2.55 | 1.3E-07 | 1.3E-05 | #N/A | #N/A |
| DN18900_c0_g1_i1 | -1.54 | 3.78 | 1.4E-07 | 1.3E-05 | #N/A | #N/A |
| DN16044_c0_g3_i2 | 8.17 | 0.10 | 1.4E-07 | 1.3E-05 | #N/A | #N/A |
| DN21631_c1_g1_i21 | 8.36 | 0.28 | 1.5E-07 | 1.4E-05 | Y1181_ARATH | 1.33E-77 |
| DN22454_c0_g1_i8 | -1.33 | 4.83 | 1.5E-07 | 1.4E-05 | OHK4_ORYSJ | 0 |
| DN15896_c0_g1_i3 | 8.65 | 0.55 | 1.6E-07 | 1.5E-05 | #N/A | #N/A |
| DN15954_c3_g9_i1 | -8.60 | -0.43 | 1.6E-07 | 1.5E-05 | #N/A | #N/A |
| DN16099_c2_g1_i14 | 9.53 | 1.39 | 1.6E-07 | 1.5E-05 | #N/A | #N/A |
| DN13987_c0_g1_i1 | -2.42 | 3.81 | 1.6E-07 | 1.5E-05 | DAO_ORYSJ | 5.75E-120 |
| DN20015_c0_g1_i22 | 8.62 | 0.52 | 1.6E-07 | 1.5E-05 | CGEP_ORYSJ | 0 |
| DN18179_c0_g1_i9 | 8.23 | 0.16 | 1.6E-07 | 1.5E-05 | ARAD1_ARATH | 2.03E-120 |
| DN15985_c0_g2_i4 | -1.61 | 4.07 | 1.6E-07 | 1.5E-05 | #N/A | #N/A |
| DN19535_c0_g3_i2 | 6.45 | 0.70 | 1.6E-07 | 1.5E-05 | #N/A | #N/A |
| DN20659_c0_g1_i1 | 1.90 | 5.10 | 1.7E-07 | 1.5E-05 | HMNGT_SORBI | 2.93E-153 |
| DN17903_c0_g4_i6 | 10.23 | 2.07 | 1.7E-07 | 1.6E-05 | RLA3_MAIZE | 6.58E-26 |
| DN20953_c0_g1_i10 | 2.42 | 4.00 | 1.7E-07 | 1.6E-05 | #N/A | #N/A |
| DN19775_c0_g3_i2 | 8.40 | 0.31 | 1.7E-07 | 1.6E-05 | TGS1_RAT | 1.19E-64 |
| DN17860_c2_g1_i9 | 8.54 | 0.44 | 1.7E-07 | 1.6E-05 | #N/A | #N/A |
| DN15650_c1_g1_i4 | -8.53 | -0.49 | 1.8E-07 | 1.7E-05 | ROC2_ORYSJ | 5.73E-119 |
| DN20776_c1_g1_i6 | 9.05 | 0.93 | 1.8E-07 | 1.7E-05 | TAF4B_ARATH | 8.02E-40 |
| DN21156_c0_g5_i3 | 8.55 | 0.45 | 1.9E-07 | 1.7E-05 | HAK1_ORYSJ | 0 |
| DN19179_c0_g2_i1 | 8.52 | 0.43 | 1.9E-07 | 1.7E-05 | G3PC1_MAIZE | 4.66E-67 |
| DN19525_c0_g3_i1 | -3.63 | 1.13 | 1.9E-07 | 1.7E-05 | PEAM1_ARATH | 0 |
| DN20040_c0_g1_i19 | 10.22 | 2.06 | 1.9E-07 | 1.8E-05 | RS242_ARATH | 1.09E-71 |
| DN20704_c2_g2_i8 | 8.09 | 0.02 | 1.9E-07 | 1.8E-05 | CML4_ORYSJ | 4.68E-97 |
| DN15384_c0_g1_i8 | 1.60 | 5.23 | 2.0E-07 | 1.8E-05 | OBGC2_ORYSJ | 0 |
| DN17679_c1_g4_i2 | -8.50 | -0.52 | 2.0E-07 | 1.8E-05 | FBK28_ARATH | 8.91E-79 |
| DN21366_c0_g3_i1 | -8.50 | -0.52 | 2.0E-07 | 1.8E-05 | #N/A | #N/A |
| DN18027_c1_g1_i12 | 8.40 | 0.31 | 2.0E-07 | 1.8E-05 | #N/A | #N/A |
| DN16546_c1_g3_i21 | 8.24 | 0.17 | 2.0E-07 | 1.9E-05 | FBT7_ARATH | 1.1E-91 |
| DN17629_c0_g1_i5 | 9.01 | 0.89 | 2.1E-07 | 1.9E-05 | S35F2_HUMAN | 5.79E-39 |
| DN18735_c1_g1_i2 | 8.62 | 0.52 | 2.1E-07 | 1.9E-05 | MYB44_ARATH | 9.01E-34 |
| DN19349_c2_g3_i4 | 8.15 | 0.08 | 2.2E-07 | 2.0E-05 | #N/A | #N/A |
| DN20902_c0_g1_i10 | 10.06 | 1.90 | 2.2E-07 | 2.0E-05 | SUVH1_TOBAC | 0 |
| DN22359_c0_g3_i4 | 11.17 | 3.00 | 2.2E-07 | 2.0E-05 | LHT2_ARATH | 3.34E-102 |
| DN18103_c0_g3_i5 | 7.95 | 3.82 | 2.4E-07 | 2.2E-05 | FABG3_BRANA | 1.25E-130 |
| DN14080_c0_g1_i5 | 10.04 | 1.88 | 2.4E-07 | 2.2E-05 | Y4833_ARATH | 3.49E-33 |
| DN20961_c2_g2_i1 | 8.64 | 0.54 | 2.4E-07 | 2.2E-05 | #N/A | #N/A |
| DN19767_c0_g1_i12 | 5.71 | 4.61 | 2.5E-07 | 2.2E-05 | IPYR4_ARATH | 3.22E-99 |
| DN18255_c0_g4_i4 | -8.47 | -0.55 | 2.5E-07 | 2.3E-05 | #N/A | #N/A |
| DN20693_c0_g2_i1 | -2.63 | 2.26 | 2.5E-07 | 2.3E-05 | ORR29_ORYSI | 1.79E-79 |
| DN18236_c0_g1_i7 | 8.08 | 0.01 | 2.7E-07 | 2.4E-05 | Y3078_ARATH | 1.71E-179 |
| DN20335_c1_g2_i1 | 8.71 | 0.60 | 2.8E-07 | 2.5E-05 | PP351_ARATH | 9.33E-84 |
| DN20830_c0_g1_i15 | 8.70 | 0.59 | 2.8E-07 | 2.5E-05 | PERK4_ARATH | 1.5E-112 |
| DN12753_c0_g1_i1 | 8.11 | 0.05 | 2.8E-07 | 2.5E-05 | HOX15_ORYSI | 4.23E-21 |
| DN22477_c2_g1_i15 | -8.89 | -0.16 | 2.9E-07 | 2.6E-05 | FBX14_ARATH | 0 |
| DN20158_c1_g3_i3 | -8.47 | -0.54 | 2.9E-07 | 2.6E-05 | #N/A | #N/A |
| DN18890_c0_g1_i11 | 8.49 | 0.40 | 2.9E-07 | 2.6E-05 | #N/A | #N/A |
| DN15848_c1_g1_i1 | 10.14 | 1.98 | 2.9E-07 | 2.6E-05 | #N/A | #N/A |
| DN19714_c0_g1_i8 | 9.68 | 1.53 | 2.9E-07 | 2.6E-05 | PBS1_ARATH | 0 |
| DN15580_c0_g1_i4 | 8.49 | 0.40 | 3.1E-07 | 2.7E-05 | #N/A | #N/A |
| DN17251_c0_g1_i1 | 8.26 | 0.18 | 3.1E-07 | 2.8E-05 | FANCL_MOUSE | 6.33E-48 |
| DN17137_c0_g3_i1 | 9.88 | 1.73 | 3.1E-07 | 2.8E-05 | #N/A | #N/A |
| DN18629_c0_g1_i16 | 9.22 | 1.09 | 3.1E-07 | 2.8E-05 | CCX4_ARATH | 0 |
| DN18870_c2_g1_i4 | 9.21 | 1.08 | 3.2E-07 | 2.8E-05 | RPAP2_ORYSJ | 0 |
| DN21994_c0_g1_i20 | 1.50 | 5.07 | 3.2E-07 | 2.8E-05 | YCHF_BACSU | 2.4E-104 |
| DN20555_c0_g1_i17 | -8.42 | -0.59 | 3.3E-07 | 2.9E-05 | #N/A | #N/A |
| DN21994_c0_g1_i16 | -1.65 | 4.06 | 3.4E-07 | 3.0E-05 | YCHF_BACSU | 2.12E-87 |
| DN17776_c0_g2_i2 | 1.32 | 4.83 | 3.5E-07 | 3.0E-05 | AMT11_ORYSJ | 0 |
| DN19072_c0_g1_i16 | 3.72 | 4.29 | 3.5E-07 | 3.0E-05 | #N/A | #N/A |
| DN19892_c0_g2_i2 | 10.10 | 1.95 | 3.5E-07 | 3.1E-05 | #N/A | #N/A |
| DN20370_c0_g2_i3 | 9.08 | 0.96 | 3.5E-07 | 3.1E-05 | TBCD8_MOUSE | 4.91E-42 |
| DN14992_c0_g1_i6 | -8.43 | -0.59 | 3.5E-07 | 3.1E-05 | BGL08_ORYSJ | 2.16E-136 |
| DN18158_c5_g2_i2 | 8.93 | 0.81 | 3.6E-07 | 3.1E-05 | UGAL1_ARATH | 3.77E-168 |
| DN19990_c0_g3_i10 | -8.42 | -0.59 | 3.6E-07 | 3.1E-05 | #N/A | #N/A |
| DN19095_c0_g1_i25 | 1.21 | 5.61 | 3.6E-07 | 3.1E-05 | T184C_XENLA | 1.76E-34 |
| DN14607_c0_g3_i6 | -1.68 | 4.73 | 3.6E-07 | 3.2E-05 | MEE14_ARATH | 2.81E-30 |
| DN15852_c0_g3_i7 | 9.28 | 1.15 | 3.7E-07 | 3.2E-05 | HMGCL_ARATH | 1.13E-77 |
| DN21843_c1_g1_i10 | 9.55 | 1.41 | 3.8E-07 | 3.2E-05 | SWI3D_ARATH | 5.16E-126 |
| DN22411_c0_g2_i13 | 8.38 | 0.29 | 3.8E-07 | 3.2E-05 | UBP26_ORYSI | 1.09E-42 |
| DN21353_c0_g1_i1 | -8.39 | -0.62 | 3.9E-07 | 3.3E-05 | #N/A | #N/A |
| DN21848_c0_g1_i9 | 8.64 | 0.53 | 3.9E-07 | 3.3E-05 | SND1_RAT | 1.2E-120 |
| DN15731_c0_g4_i4 | 8.86 | 0.75 | 4.1E-07 | 3.5E-05 | #N/A | #N/A |
| DN20344_c3_g1_i1 | -3.02 | 3.82 | 4.2E-07 | 3.6E-05 | #N/A | #N/A |
| DN15450_c0_g1_i8 | 9.93 | 1.78 | 4.2E-07 | 3.6E-05 | CSPLC_MAIZE | 2.64E-87 |
| DN19034_c0_g1_i18 | 8.69 | 0.58 | 4.2E-07 | 3.6E-05 | #N/A | #N/A |
| DN18461_c1_g4_i3 | 10.07 | 1.91 | 4.2E-07 | 3.6E-05 | OGG1_ARATH | 4.9E-119 |
| DN16401_c0_g1_i3 | 8.44 | 0.35 | 4.3E-07 | 3.6E-05 | #N/A | #N/A |
| DN21296_c0_g1_i17 | 9.17 | 1.04 | 4.3E-07 | 3.6E-05 | #N/A | #N/A |
| DN21309_c1_g1_i13 | 9.70 | 1.55 | 4.3E-07 | 3.6E-05 | SMG8_BOVIN | 8.53E-22 |
| DN20133_c0_g2_i7 | 8.48 | 0.39 | 4.4E-07 | 3.7E-05 | CSPLH_MAIZE | 1.42E-47 |
| DN21563_c1_g1_i1 | 8.07 | 0.00 | 4.4E-07 | 3.7E-05 | #N/A | #N/A |
| DN21010_c0_g1_i6 | 5.41 | 1.00 | 4.4E-07 | 3.8E-05 | #N/A | #N/A |
| DN21881_c2_g1_i3 | 9.10 | 0.98 | 4.5E-07 | 3.8E-05 | VIP4_ARATH | 3.71E-43 |
| DN15245_c1_g1_i20 | 8.40 | 0.31 | 4.7E-07 | 4.0E-05 | PIGU_HUMAN | 6.39E-37 |
| DN18370_c1_g1_i3 | -1.41 | 8.47 | 4.8E-07 | 4.0E-05 | PSAN_MAIZE | 2.23E-59 |
| DN18377_c0_g6_i2 | 1.44 | 5.70 | 4.8E-07 | 4.0E-05 | #N/A | #N/A |
| DN16562_c0_g4_i9 | 9.88 | 1.73 | 4.9E-07 | 4.1E-05 | P2C34_ORYSJ | 0 |
| DN22392_c0_g1_i12 | -2.13 | 5.92 | 4.9E-07 | 4.1E-05 | NLP2_ORYSJ | 0 |
| DN15592_c1_g3_i2 | 9.45 | 1.31 | 4.9E-07 | 4.1E-05 | #N/A | #N/A |
| DN19463_c0_g1_i11 | 8.11 | 0.04 | 4.9E-07 | 4.1E-05 | AT18D_ARATH | 0 |
| DN15532_c0_g1_i7 | -6.90 | 0.59 | 5.0E-07 | 4.2E-05 | #N/A | #N/A |
| DN20060_c0_g3_i5 | 8.51 | 0.42 | 5.0E-07 | 4.2E-05 | SWC4_ARATH | 2.97E-106 |
| DN18060_c0_g4_i10 | -8.35 | -0.65 | 5.2E-07 | 4.3E-05 | #N/A | #N/A |
| DN20041_c0_g2_i2 | -2.78 | 2.20 | 5.2E-07 | 4.4E-05 | #N/A | #N/A |
| DN22199_c0_g1_i11 | 9.61 | 1.47 | 5.3E-07 | 4.4E-05 | SIZ1_ORYSJ | 0 |
| DN21434_c0_g1_i1 | -1.14 | 7.33 | 5.4E-07 | 4.5E-05 | TSJT1_TOBAC | 1.93E-29 |
| DN16161_c0_g1_i2 | 10.06 | 1.90 | 5.4E-07 | 4.5E-05 | #N/A | #N/A |
| DN15826_c0_g2_i15 | -8.34 | -0.66 | 5.5E-07 | 4.6E-05 | PURU1_ARATH | 3.68E-88 |
| DN22228_c1_g2_i2 | -8.34 | -0.66 | 5.5E-07 | 4.6E-05 | IF5A_MAIZE | 9.43E-110 |
| DN21205_c1_g4_i8 | -8.41 | -0.60 | 5.6E-07 | 4.6E-05 | GOGC2_ARATH | 7.62E-56 |
| DN19937_c3_g1_i2 | 8.93 | 0.81 | 5.7E-07 | 4.7E-05 | THIC_ARATH | 0 |
| DN22280_c1_g2_i6 | 6.97 | 3.77 | 5.9E-07 | 4.9E-05 | DNJH2_ALLPO | 1.31E-164 |
| DN12577_c0_g1_i1 | -8.32 | -0.68 | 5.9E-07 | 4.9E-05 | HS188_ORYSJ | 7.18E-30 |
| DN19965_c0_g2_i1 | 9.94 | 1.79 | 6.0E-07 | 4.9E-05 | RPM1_ARATH | 4.06E-57 |
| DN15659_c0_g2_i3 | 10.05 | 1.90 | 6.0E-07 | 4.9E-05 | #N/A | #N/A |
| DN16636_c2_g4_i3 | 9.60 | 1.46 | 6.1E-07 | 5.0E-05 | #N/A | #N/A |
| DN15447_c1_g1_i7 | 10.18 | 2.02 | 6.2E-07 | 5.1E-05 | INVA_MAIZE | 0 |
| DN13816_c0_g1_i2 | 1.84 | 3.45 | 6.2E-07 | 5.1E-05 | #N/A | #N/A |
| DN14767_c0_g1_i2 | -8.32 | -0.68 | 6.3E-07 | 5.1E-05 | 6PGD1_ORYSJ | 0 |
| DN18653_c0_g1_i12 | 10.39 | 2.23 | 6.3E-07 | 5.2E-05 | ADCS_ORYSJ | 0 |
| DN21016_c0_g3_i12 | 9.23 | 1.10 | 6.4E-07 | 5.2E-05 | #N/A | #N/A |
| DN14934_c0_g1_i1 | -8.65 | -0.38 | 6.6E-07 | 5.4E-05 | MACP1_ARATH | 3.42E-173 |
| DN20705_c0_g2_i2 | 4.79 | 1.30 | 6.6E-07 | 5.4E-05 | WRK19_ARATH | 7.41E-59 |
| DN16791_c0_g1_i6 | 9.36 | 1.22 | 6.7E-07 | 5.4E-05 | #N/A | #N/A |
| DN19975_c0_g1_i14 | 8.45 | 0.36 | 6.7E-07 | 5.4E-05 | B3GTF_ARATH | 9.39E-179 |
| DN18967_c0_g2_i13 | -8.37 | -0.64 | 6.8E-07 | 5.5E-05 | #N/A | #N/A |
| DN22552_c0_g1_i16 | 9.45 | 1.31 | 6.8E-07 | 5.5E-05 | #N/A | #N/A |
| DN18900_c0_g1_i4 | -2.11 | 3.04 | 6.9E-07 | 5.5E-05 | #N/A | #N/A |
| DN20243_c0_g1_i1 | -8.58 | -0.45 | 6.9E-07 | 5.6E-05 | #N/A | #N/A |
| DN12042_c0_g1_i2 | 9.06 | 0.94 | 6.9E-07 | 5.6E-05 | #N/A | #N/A |
| DN19271_c2_g1_i5 | 8.82 | 0.71 | 6.9E-07 | 5.6E-05 | #N/A | #N/A |
| DN18802_c1_g2_i17 | 9.07 | 0.94 | 7.0E-07 | 5.6E-05 | #N/A | #N/A |
| DN18055_c0_g1_i4 | 10.39 | 2.23 | 7.2E-07 | 5.7E-05 | #N/A | #N/A |
| DN20015_c0_g1_i6 | 8.71 | 0.60 | 7.2E-07 | 5.7E-05 | CGEP_ORYSJ | 0 |
| DN20953_c0_g1_i1 | 2.71 | 6.74 | 7.3E-07 | 5.9E-05 | ELI9_HORVU | 5.97E-50 |
| DN22429_c0_g2_i8 | 2.25 | 2.70 | 7.5E-07 | 6.0E-05 | PUR4_ARATH | 0 |
| DN15357_c0_g1_i2 | 8.98 | 0.86 | 7.7E-07 | 6.1E-05 | GDPD6_ARATH | 3.37E-172 |
| DN21210_c0_g1_i18 | -8.31 | -0.68 | 7.8E-07 | 6.2E-05 | #N/A | #N/A |
| DN19324_c1_g1_i6 | 8.70 | 0.59 | 7.9E-07 | 6.3E-05 | SBT25_ARATH | 2.37E-165 |
| DN22053_c0_g1_i12 | -8.28 | -0.71 | 8.0E-07 | 6.4E-05 | PP199_ARATH | 0 |
| DN17144_c0_g2_i4 | 10.22 | 2.06 | 8.1E-07 | 6.4E-05 | #N/A | #N/A |
| DN18928_c0_g1_i1 | 6.20 | 1.25 | 8.2E-07 | 6.5E-05 | ALB31_ARATH | 3.56E-152 |
| DN22468_c1_g3_i14 | 9.39 | 1.25 | 8.2E-07 | 6.5E-05 | BRE1B_ORYSJ | 0 |
| DN531_c0_g1_i3 | 9.51 | 1.37 | 8.3E-07 | 6.6E-05 | TBL19_ARATH | 1.63E-97 |
| DN22335_c1_g1_i4 | 8.69 | 0.58 | 8.3E-07 | 6.6E-05 | KAT2_ORYSJ | 4.6E-80 |
| DN16638_c2_g1_i2 | 9.03 | 0.91 | 8.3E-07 | 6.6E-05 | #N/A | #N/A |
| DN20821_c0_g1_i2 | -8.57 | -0.47 | 8.4E-07 | 6.6E-05 | PYRG_DICDI | 3.33E-116 |
| DN14365_c0_g1_i7 | -4.43 | 5.65 | 8.5E-07 | 6.7E-05 | TRXM_MAIZE | 5.91E-76 |
| DN18919_c0_g2_i1 | 2.18 | 2.69 | 8.5E-07 | 6.7E-05 | DCE3_ARATH | 3.15E-66 |
| DN14916_c0_g1_i7 | 9.96 | 1.81 | 8.6E-07 | 6.8E-05 | #N/A | #N/A |
| DN18428_c1_g2_i1 | 7.92 | -0.13 | 8.7E-07 | 6.9E-05 | #N/A | #N/A |
| DN20192_c0_g1_i2 | -1.13 | 4.96 | 8.8E-07 | 6.9E-05 | RBM39_PONAB | 3.57E-53 |
| DN18175_c0_g2_i4 | 9.54 | 1.40 | 8.9E-07 | 6.9E-05 | TMCO1_RAT | 2.69E-35 |
| DN17090_c0_g2_i4 | -8.49 | 6.70 | 8.9E-07 | 6.9E-05 | CATB_PONAB | 9.35E-77 |
| DN18778_c1_g1_i1 | 9.67 | 1.53 | 9.3E-07 | 7.3E-05 | CRK23_ARATH | 3.54E-86 |
| DN14365_c0_g1_i3 | -1.40 | 4.38 | 9.5E-07 | 7.4E-05 | TRXM_MAIZE | 2.7E-100 |
| DN18132_c1_g5_i1 | 8.66 | 0.55 | 9.5E-07 | 7.4E-05 | ZFP1_WHEAT | 1.35E-59 |
| DN14926_c0_g1_i11 | 5.07 | 1.86 | 9.6E-07 | 7.4E-05 | RLA1_MAIZE | 7.25E-37 |
| DN20318_c0_g2_i11 | 6.58 | 0.82 | 9.6E-07 | 7.5E-05 | CCB4_ARATH | 1.49E-53 |
| DN21176_c0_g1_i15 | 8.16 | 0.09 | 9.7E-07 | 7.5E-05 | RENT3_ARATH | 2.17E-62 |
| DN21391_c0_g1_i13 | 4.66 | 1.37 | 9.7E-07 | 7.5E-05 | PAP2_ORYSJ | 4.61E-73 |
| DN21425_c0_g3_i3 | 2.34 | 2.62 | 1.0E-06 | 7.8E-05 | PRL1_ARATH | 0 |
| DN20949_c1_g1_i19 | -3.06 | 2.06 | 1.0E-06 | 7.9E-05 | #N/A | #N/A |
| DN21009_c2_g1_i5 | -8.28 | -0.71 | 1.0E-06 | 8.0E-05 | #N/A | #N/A |
| DN20636_c0_g3_i5 | 1.60 | 4.44 | 1.1E-06 | 8.1E-05 | AROG_ORYSJ | 0 |
| DN15762_c0_g1_i9 | 9.89 | 1.74 | 1.1E-06 | 8.2E-05 | #N/A | #N/A |
| DN18452_c0_g1_i17 | 10.81 | 2.64 | 1.1E-06 | 8.3E-05 | HAK22_ORYSJ | 0 |
| DN21011_c2_g1_i7 | -2.66 | 2.28 | 1.1E-06 | 8.4E-05 | #N/A | #N/A |
| DN22053_c0_g1_i9 | -8.25 | -0.73 | 1.1E-06 | 8.4E-05 | PP199_ARATH | 0 |
| DN16216_c0_g5_i6 | -2.04 | 3.09 | 1.1E-06 | 8.4E-05 | #N/A | #N/A |
| DN21261_c1_g1_i4 | 3.22 | 6.17 | 1.1E-06 | 8.4E-05 | NDHO_ARATH | 1.48E-48 |
| DN15630_c0_g1_i12 | 8.55 | 0.45 | 1.1E-06 | 8.4E-05 | GPN1_DICDI | 4.6E-63 |
| DN18236_c0_g1_i4 | -1.48 | 4.31 | 1.1E-06 | 8.6E-05 | Y3078_ARATH | 1.61E-179 |
| DN16173_c1_g3_i6 | 8.10 | 0.03 | 1.1E-06 | 8.7E-05 | UTR4_ARATH | 2.45E-102 |
| DN19048_c3_g2_i6 | -8.26 | -0.73 | 1.1E-06 | 8.7E-05 | FB304_ARATH | 0 |
| DN21964_c0_g1_i12 | 2.46 | 3.48 | 1.1E-06 | 8.7E-05 | ILVD_ARATH | 0 |
| DN15009_c0_g8_i6 | 1.39 | 4.84 | 1.2E-06 | 8.9E-05 | MAEA_DANRE | 7.28E-71 |
| DN17984_c0_g1_i2 | 6.94 | 2.66 | 1.2E-06 | 9.0E-05 | NAC67_ORYSJ | 1.25E-39 |
| DN14424_c0_g1_i12 | 6.70 | 0.93 | 1.2E-06 | 9.0E-05 | #N/A | #N/A |
| DN17221_c1_g1_i4 | 7.90 | -0.15 | 1.2E-06 | 9.0E-05 | #N/A | #N/A |
| DN17115_c0_g1_i1 | 9.99 | 1.84 | 1.2E-06 | 9.1E-05 | APRL6_ORYSJ | 2.84E-105 |
| DN19852_c0_g2_i7 | 9.61 | 1.47 | 1.2E-06 | 9.1E-05 | #N/A | #N/A |
| DN21463_c0_g2_i2 | 1.93 | 3.67 | 1.2E-06 | 9.2E-05 | PAL1_ORYSJ | 1.29E-162 |
| DN16365_c0_g1_i2 | 9.55 | 1.41 | 1.2E-06 | 9.2E-05 | #N/A | #N/A |
| DN22085_c0_g1_i12 | 8.37 | 0.29 | 1.2E-06 | 9.4E-05 | #N/A | #N/A |
| DN14772_c0_g1_i11 | 10.05 | 1.89 | 1.3E-06 | 9.8E-05 | Y3589_ARATH | 7.48E-90 |
| DN21684_c0_g1_i6 | -1.25 | 8.56 | 1.3E-06 | 1.0E-04 | ZEP_ORYSJ | 1.76E-137 |
| DN20360_c2_g1_i8 | 9.79 | 1.64 | 1.3E-06 | 1.0E-04 | THD1_ARATH | 0 |
| DN21308_c0_g6_i3 | 7.99 | -0.07 | 1.3E-06 | 1.0E-04 | #N/A | #N/A |
| DN20737_c0_g1_i16 | -1.23 | 7.96 | 1.4E-06 | 1.0E-04 | #N/A | #N/A |
| DN16175_c0_g1_i21 | -1.37 | 4.61 | 1.4E-06 | 1.0E-04 | #N/A | #N/A |
| DN18226_c0_g1_i13 | 7.97 | -0.09 | 1.4E-06 | 1.0E-04 | GPT2_ARATH | 1.09E-123 |
| DN20849_c1_g3_i3 | 8.49 | 0.40 | 1.4E-06 | 1.0E-04 | #N/A | #N/A |
| DN17290_c0_g5_i3 | 2.11 | 3.38 | 1.4E-06 | 1.0E-04 | Y1154_ARATH | 0 |
| DN18760_c1_g1_i3 | 8.80 | 0.69 | 1.4E-06 | 1.1E-04 | #N/A | #N/A |
| DN15476_c1_g1_i1 | 8.20 | 0.12 | 1.4E-06 | 1.1E-04 | #N/A | #N/A |
| DN19358_c0_g4_i13 | 9.13 | 1.01 | 1.4E-06 | 1.1E-04 | #N/A | #N/A |
| DN18047_c0_g2_i1 | -8.22 | -0.77 | 1.4E-06 | 1.1E-04 | WDR12_CHLRE | 2.07E-71 |
| DN21162_c0_g3_i4 | -8.24 | -0.74 | 1.4E-06 | 1.1E-04 | SEC23_ASHGO | 2.75E-32 |
| DN18735_c1_g1_i5 | 8.43 | 0.34 | 1.4E-06 | 1.1E-04 | MYB44_ARATH | 2.12E-31 |
| DN16595_c0_g4_i3 | 2.82 | 1.78 | 1.5E-06 | 1.1E-04 | DCE_PETHY | 0 |
| DN21434_c0_g1_i3 | -2.56 | 2.39 | 1.5E-06 | 1.1E-04 | #N/A | #N/A |
| DN15762_c0_g1_i4 | 11.04 | 2.87 | 1.5E-06 | 1.1E-04 | #N/A | #N/A |
| DN15058_c0_g3_i1 | -8.37 | -0.64 | 1.5E-06 | 1.1E-04 | #N/A | #N/A |
| DN21481_c0_g10_i1 | 4.76 | 0.40 | 1.5E-06 | 1.1E-04 | #N/A | #N/A |
| DN19579_c0_g2_i4 | -1.22 | 6.60 | 1.6E-06 | 1.1E-04 | #N/A | #N/A |
| DN22053_c0_g1_i25 | 1.69 | 3.83 | 1.6E-06 | 1.1E-04 | PP199_ARATH | 0 |
| DN21994_c0_g1_i10 | 9.90 | 1.75 | 1.6E-06 | 1.2E-04 | #N/A | #N/A |
| DN22280_c1_g2_i11 | -1.23 | 5.37 | 1.6E-06 | 1.2E-04 | DNJH_CUCSA | 6.87E-92 |
| DN22012_c1_g2_i25 | 13.86 | 5.68 | 1.6E-06 | 1.2E-04 | #N/A | #N/A |
| DN15580_c0_g1_i8 | 8.87 | 0.76 | 1.6E-06 | 1.2E-04 | #N/A | #N/A |
| DN14234_c0_g1_i1 | -1.16 | 5.31 | 1.6E-06 | 1.2E-04 | #N/A | #N/A |
| DN16759_c1_g1_i1 | 8.27 | 0.19 | 1.6E-06 | 1.2E-04 | BAHL1_ORYSJ | 0 |
| DN19989_c0_g1_i3 | 7.97 | -0.08 | 1.6E-06 | 1.2E-04 | MLH1_ARATH | 1.28E-60 |
| DN20721_c1_g1_i26 | 9.19 | 1.06 | 1.6E-06 | 1.2E-04 | #N/A | #N/A |
| DN14922_c0_g1_i5 | 8.21 | 0.13 | 1.6E-06 | 1.2E-04 | #N/A | #N/A |
| DN21312_c1_g1_i1 | 9.99 | 1.84 | 1.7E-06 | 1.2E-04 | EBP_ORYSJ | 8.1E-120 |
| DN14896_c1_g2_i4 | 7.99 | -0.06 | 1.7E-06 | 1.2E-04 | #N/A | #N/A |
| DN16248_c0_g3_i4 | 5.77 | 2.79 | 1.7E-06 | 1.2E-04 | #N/A | #N/A |
| DN21312_c1_g2_i3 | 7.66 | 4.06 | 1.7E-06 | 1.2E-04 | TL1Y_ARATH | 1.22E-35 |
| DN22761_c0_g1_i3 | 9.16 | 1.04 | 1.7E-06 | 1.2E-04 | VCR_ARATH | 1.53E-148 |
| DN17358_c2_g1_i1 | -8.15 | -0.83 | 1.8E-06 | 1.3E-04 | #N/A | #N/A |
| DN21992_c0_g1_i17 | -8.19 | -0.80 | 1.8E-06 | 1.3E-04 | #N/A | #N/A |
| DN15995_c0_g4_i1 | 4.02 | 1.58 | 1.8E-06 | 1.3E-04 | LOX3_ORYSJ | 0 |
| DN19935_c2_g2_i13 | 8.56 | 0.46 | 1.8E-06 | 1.3E-04 | #N/A | #N/A |
| DN22012_c1_g2_i7 | -1.16 | 6.98 | 1.9E-06 | 1.4E-04 | #N/A | #N/A |
| DN18627_c1_g1_i4 | -1.56 | 3.59 | 2.0E-06 | 1.4E-04 | #N/A | #N/A |
| DN20902_c0_g1_i14 | 9.75 | 1.60 | 2.0E-06 | 1.4E-04 | SUVH1_TOBAC | 0 |
| DN15838_c0_g1_i6 | 8.18 | 0.10 | 2.0E-06 | 1.4E-04 | ATG10_ARATH | 1.41E-30 |
| DN20495_c0_g2_i11 | -1.31 | 3.94 | 2.0E-06 | 1.4E-04 | #N/A | #N/A |
| DN15604_c0_g2_i5 | 1.40 | 4.62 | 2.0E-06 | 1.4E-04 | #N/A | #N/A |
| DN18977_c2_g3_i4 | 12.92 | 4.74 | 2.1E-06 | 1.5E-04 | Y2104_ARATH | 3.4E-138 |
| DN19977_c0_g1_i9 | 9.54 | 1.40 | 2.1E-06 | 1.5E-04 | #N/A | #N/A |
| DN18469_c0_g3_i7 | 5.47 | 3.10 | 2.1E-06 | 1.5E-04 | CLPD1_ORYSJ | 3.64E-165 |
| DN15021_c0_g2_i8 | -3.20 | 1.38 | 2.1E-06 | 1.5E-04 | SWET4_ORYSJ | 1.56E-136 |
| DN18456_c0_g1_i4 | 8.09 | 0.03 | 2.1E-06 | 1.5E-04 | GUN10_ORYSJ | 2.04E-122 |
| DN22341_c1_g1_i15 | 2.83 | 1.91 | 2.2E-06 | 1.5E-04 | #N/A | #N/A |
| DN16097_c0_g2_i2 | -8.54 | 7.54 | 2.2E-06 | 1.5E-04 | SUI1_MAIZE | 8.19E-65 |
| DN16981_c2_g1_i7 | 9.83 | 1.68 | 2.2E-06 | 1.5E-04 | #N/A | #N/A |
| DN18864_c2_g1_i2 | 9.32 | 3.48 | 2.2E-06 | 1.5E-04 | #N/A | #N/A |
| DN14769_c0_g2_i5 | -1.76 | 4.53 | 2.2E-06 | 1.6E-04 | #N/A | #N/A |
| DN21803_c0_g1_i4 | -2.62 | 4.08 | 2.2E-06 | 1.6E-04 | C14B3_MAIZE | 0 |
| DN16059_c4_g2_i12 | -3.03 | 5.20 | 2.2E-06 | 1.6E-04 | CLPP2_ARATH | 9.68E-94 |
| DN22491_c1_g2_i9 | 2.28 | 6.48 | 2.2E-06 | 1.6E-04 | Y1669_ARATH | 5.1E-108 |
| DN16783_c3_g2_i13 | -7.45 | 1.73 | 2.3E-06 | 1.6E-04 | #N/A | #N/A |
| DN19809_c1_g1_i15 | 8.26 | 0.18 | 2.3E-06 | 1.6E-04 | #N/A | #N/A |
| DN19516_c0_g5_i2 | -8.10 | -0.87 | 2.3E-06 | 1.6E-04 | #N/A | #N/A |
| DN15470_c0_g1_i1 | 11.68 | 3.50 | 2.4E-06 | 1.6E-04 | PAP6_ARATH | 6.96E-36 |
| DN17183_c2_g1_i3 | 8.36 | 0.27 | 2.4E-06 | 1.7E-04 | PPA18_ARATH | 5E-104 |
| DN18096_c1_g1_i7 | 2.18 | 2.69 | 2.4E-06 | 1.7E-04 | #N/A | #N/A |
| DN15738_c1_g4_i4 | 7.81 | -0.24 | 2.4E-06 | 1.7E-04 | #N/A | #N/A |
| DN22086_c0_g2_i1 | 2.74 | 1.86 | 2.4E-06 | 1.7E-04 | VP35B_ARATH | 0 |
| DN15848_c1_g1_i8 | 8.86 | 0.74 | 2.4E-06 | 1.7E-04 | NAC83_ARATH | 1.55E-40 |
| DN19637_c1_g3_i7 | 10.64 | 2.48 | 2.4E-06 | 1.7E-04 | P2C50_ORYSJ | 1.43E-83 |
| DN20148_c0_g1_i14 | 9.89 | 1.74 | 2.5E-06 | 1.7E-04 | UBC22_ARATH | 4.28E-99 |
| DN14953_c0_g3_i1 | -8.08 | -0.89 | 2.5E-06 | 1.7E-04 | #N/A | #N/A |
| DN20003_c0_g1_i3 | 7.80 | -0.24 | 2.5E-06 | 1.7E-04 | FAHD1_BOVIN | 2.19E-44 |
| DN21160_c1_g1_i15 | -1.13 | 6.70 | 2.6E-06 | 1.8E-04 | NIA1_MAIZE | 0 |
| DN17887_c1_g2_i1 | -1.00 | 6.81 | 2.6E-06 | 1.8E-04 | CSLE6_ORYSJ | 0 |
| DN17834_c0_g2_i8 | 3.22 | 2.86 | 2.7E-06 | 1.9E-04 | LCAT1_ARATH | 4.89E-147 |
| DN20338_c0_g1_i29 | 8.87 | 0.75 | 2.7E-06 | 1.9E-04 | MENE_ARATH | 1.41E-104 |
| DN20664_c0_g1_i10 | 8.61 | 0.51 | 2.7E-06 | 1.9E-04 | ASPR1_ORYSJ | 0 |
| DN22129_c0_g1_i18 | -1.33 | 6.21 | 2.7E-06 | 1.9E-04 | TSJT1_TOBAC | 3.64E-21 |
| DN19676_c0_g2_i2 | -1.17 | 4.87 | 2.8E-06 | 1.9E-04 | 2A5N_ARATH | 0 |
| DN16824_c2_g2_i5 | 8.87 | 0.76 | 2.8E-06 | 1.9E-04 | #N/A | #N/A |
| DN19376_c1_g7_i3 | -7.13 | -0.34 | 2.9E-06 | 2.0E-04 | #N/A | #N/A |
| DN17845_c0_g1_i2 | 8.93 | 0.81 | 3.0E-06 | 2.1E-04 | TLP8_ORYSJ | 1.69E-33 |
| DN16628_c0_g3_i2 | 8.27 | 0.19 | 3.1E-06 | 2.1E-04 | PGLR_VITVI | 1.53E-70 |
| DN11500_c0_g1_i2 | -4.89 | -0.09 | 3.1E-06 | 2.1E-04 | #N/A | #N/A |
| DN16527_c0_g2_i7 | 9.28 | 1.15 | 3.2E-06 | 2.2E-04 | #N/A | #N/A |
| DN15422_c0_g4_i1 | -1.67 | 3.00 | 3.2E-06 | 2.2E-04 | RPM1_ARATH | 6.21E-36 |
| DN18575_c1_g1_i6 | 8.44 | 0.35 | 3.2E-06 | 2.2E-04 | #N/A | #N/A |
| DN17522_c0_g2_i7 | -1.21 | 6.08 | 3.2E-06 | 2.2E-04 | PSAG_HORVU | 3.79E-55 |
| DN15184_c0_g3_i2 | 7.79 | -0.25 | 3.2E-06 | 2.2E-04 | #N/A | #N/A |
| DN21808_c2_g1_i5 | -1.68 | 3.60 | 3.3E-06 | 2.2E-04 | #N/A | #N/A |
| DN14604_c0_g1_i1 | -7.99 | -0.96 | 3.3E-06 | 2.2E-04 | #N/A | #N/A |
| DN19400_c1_g1_i3 | 6.17 | 2.52 | 3.3E-06 | 2.2E-04 | #N/A | #N/A |
| DN19047_c1_g6_i2 | 8.65 | 0.54 | 3.3E-06 | 2.3E-04 | AROG_ARATH | 1.35E-38 |
| DN21742_c1_g2_i1 | -8.07 | -0.89 | 3.4E-06 | 2.3E-04 | #N/A | #N/A |
| DN21839_c0_g5_i2 | -1.35 | 6.61 | 3.4E-06 | 2.3E-04 | NPY2_ARATH | 0 |
| DN20039_c1_g2_i9 | 6.72 | 1.89 | 3.4E-06 | 2.3E-04 | RNP1_ARATH | 2.55E-38 |
| DN19835_c0_g1_i25 | 5.36 | 0.95 | 3.4E-06 | 2.3E-04 | C3H46_ORYSJ | 1.2E-109 |
| DN17545_c1_g3_i1 | 3.09 | 3.23 | 3.5E-06 | 2.3E-04 | OPT7_ARATH | 0 |
| DN20681_c0_g1_i6 | 8.24 | 0.16 | 3.5E-06 | 2.3E-04 | BCAT5_ARATH | 1.5E-152 |
| DN21643_c1_g2_i28 | 8.41 | 0.32 | 3.5E-06 | 2.4E-04 | GBA2_MOUSE | 6.23E-134 |
| DN17535_c0_g4_i1 | 8.04 | -0.02 | 3.6E-06 | 2.4E-04 | Y2165_ARATH | 3.74E-63 |
| DN21521_c0_g4_i1 | 4.74 | 3.58 | 3.6E-06 | 2.4E-04 | RL30_WHEAT | 4.1E-42 |
| DN17810_c0_g1_i5 | 2.10 | 3.13 | 3.6E-06 | 2.4E-04 | RBX1A_ARATH | 9.38E-62 |
| DN16085_c2_g1_i15 | -8.00 | -0.95 | 3.7E-06 | 2.5E-04 | #N/A | #N/A |
| DN17690_c0_g1_i5 | 1.26 | 7.96 | 3.7E-06 | 2.5E-04 | NDK1_ORYSJ | 9.69E-71 |
| DN17436_c0_g1_i2 | 7.94 | -0.12 | 3.7E-06 | 2.5E-04 | PTR2_ARATH | 3.99E-54 |
| DN22391_c2_g5_i1 | 9.29 | 1.16 | 3.7E-06 | 2.5E-04 | #N/A | #N/A |
| DN17315_c2_g3_i22 | 7.99 | -0.07 | 3.8E-06 | 2.5E-04 | NSUN5_HUMAN | 2.92E-69 |
| DN17662_c2_g11_i1 | -2.38 | 1.67 | 3.8E-06 | 2.5E-04 | IFRH_MAIZE | 2.71E-38 |
| DN21426_c2_g1_i3 | -2.68 | 5.02 | 3.8E-06 | 2.5E-04 | CCD4_ARATH | 1.28E-150 |
| DN20375_c1_g1_i7 | 5.74 | 0.09 | 3.9E-06 | 2.6E-04 | #N/A | #N/A |
| DN15350_c0_g6_i4 | 7.30 | 1.50 | 3.9E-06 | 2.6E-04 | Y1669_ARATH | 5.6E-27 |
| DN18156_c1_g2_i5 | 9.25 | 1.12 | 3.9E-06 | 2.6E-04 | ACR9_ARATH | 2.51E-149 |
| DN20721_c1_g1_i11 | 3.67 | 1.40 | 3.9E-06 | 2.6E-04 | #N/A | #N/A |
| DN18728_c2_g3_i1 | 8.26 | 0.18 | 4.0E-06 | 2.7E-04 | ORR6_ORYSI | 1.12E-59 |
| DN15899_c0_g2_i2 | 9.91 | 1.76 | 4.0E-06 | 2.7E-04 | C81E1_GLYEC | 3.36E-54 |
| DN17975_c0_g1_i3 | 9.47 | 1.33 | 4.1E-06 | 2.7E-04 | #N/A | #N/A |
| DN19502_c1_g1_i26 | 1.27 | 5.33 | 4.1E-06 | 2.7E-04 | #N/A | #N/A |
| DN18051_c0_g1_i12 | 8.39 | 0.30 | 4.1E-06 | 2.7E-04 | #N/A | #N/A |
| DN17719_c0_g1_i12 | 8.95 | 0.83 | 4.1E-06 | 2.7E-04 | #N/A | #N/A |
| DN21785_c0_g2_i10 | 10.20 | 2.04 | 4.1E-06 | 2.7E-04 | WAK5_ARATH | 3.1E-129 |
| DN15459_c0_g1_i5 | 8.63 | 0.53 | 4.1E-06 | 2.7E-04 | CSK2B_ARATH | 8.72E-42 |
| DN15344_c0_g3_i5 | 8.99 | 0.87 | 4.2E-06 | 2.7E-04 | GT51_ORYSJ | 1.57E-58 |
| DN22812_c3_g1_i6 | 10.51 | 2.35 | 4.2E-06 | 2.8E-04 | NDK2_TOBAC | 3.19E-83 |
| DN20552_c1_g2_i9 | -2.80 | 0.65 | 4.3E-06 | 2.8E-04 | SPHK1_ARATH | 4.45E-60 |
| DN18060_c0_g4_i2 | -7.94 | -1.00 | 4.4E-06 | 2.9E-04 | #N/A | #N/A |
| DN22085_c0_g1_i11 | 6.06 | 1.61 | 4.4E-06 | 2.9E-04 | #N/A | #N/A |
| DN15986_c1_g1_i2 | 9.85 | 1.70 | 4.4E-06 | 2.9E-04 | DIV_ANTMA | 7.3E-25 |
| DN19895_c0_g1_i22 | 8.40 | 0.31 | 4.5E-06 | 2.9E-04 | SAC2_ARATH | 3.19E-30 |
| DN20686_c1_g2_i6 | 7.85 | -0.20 | 4.5E-06 | 2.9E-04 | GPI8_YEAST | 4.99E-106 |
| DN18596_c1_g2_i12 | -7.92 | -1.02 | 4.6E-06 | 3.0E-04 | LOG_ORYSJ | 2.78E-30 |
| DN20895_c0_g1_i13 | -7.92 | -1.02 | 4.6E-06 | 3.0E-04 | #N/A | #N/A |
| DN16352_c0_g3_i4 | 9.21 | 1.08 | 4.6E-06 | 3.0E-04 | TIF3_ORYSJ | 4.21E-40 |
| DN9072_c0_g1_i3 | 2.48 | 5.04 | 4.7E-06 | 3.0E-04 | **URT1_FRAAN** | 5.49E-72 |
| DN19935_c2_g4_i2 | 10.01 | 1.86 | 4.8E-06 | 3.1E-04 | #N/A | #N/A |
| DN15492_c0_g1_i1 | 5.35 | 1.59 | 4.8E-06 | 3.1E-04 | HEXO1_ARATH | 0 |
| DN22574_c2_g1_i3 | 6.84 | 2.57 | 4.9E-06 | 3.2E-04 | MGLL_HUMAN | 1.74E-23 |
| DN15578_c0_g2_i3 | 9.83 | 1.68 | 5.0E-06 | 3.2E-04 | #N/A | #N/A |
| DN18941_c2_g3_i2 | 8.33 | 0.25 | 5.0E-06 | 3.3E-04 | NDK4_ARATH | 1.95E-81 |
| DN21255_c0_g1_i3 | 8.21 | 0.13 | 5.0E-06 | 3.3E-04 | AVT1_YEAST | 6.85E-26 |
| DN16393_c0_g1_i5 | 9.26 | 1.13 | 5.2E-06 | 3.4E-04 | KCY1_ORYSJ | 1.49E-99 |
| DN16324_c0_g1_i6 | 8.94 | 0.82 | 5.2E-06 | 3.4E-04 | DBR1_ORYSJ | 2.8E-167 |
| DN18072_c0_g3_i7 | 8.59 | 0.49 | 5.3E-06 | 3.4E-04 | PHL11_ARATH | 3.52E-29 |
| DN17918_c1_g2_i1 | 8.75 | 0.64 | 5.4E-06 | 3.5E-04 | #N/A | #N/A |
| DN19431_c1_g1_i1 | -7.88 | -1.05 | 5.5E-06 | 3.5E-04 | VATE_CITUN | 7.79E-37 |
| DN17710_c0_g1_i4 | 1.52 | 4.07 | 5.5E-06 | 3.5E-04 | #N/A | #N/A |
| DN15364_c0_g2_i6 | 5.81 | 1.73 | 5.6E-06 | 3.6E-04 | #N/A | #N/A |
| DN18213_c0_g1_i12 | 8.47 | 0.38 | 5.6E-06 | 3.6E-04 | RQL3_ARATH | 5.98E-177 |
| DN22318_c1_g1_i8 | 9.94 | 1.79 | 5.6E-06 | 3.6E-04 | KSG4_ARATH | 6.61E-66 |
| DN18926_c0_g5_i2 | 8.85 | 0.73 | 5.7E-06 | 3.6E-04 | GAT18_ORYSJ | 1.21E-96 |
| DN19874_c0_g1_i2 | 8.17 | 0.10 | 5.7E-06 | 3.6E-04 | #N/A | #N/A |
| DN16039_c0_g4_i2 | 10.14 | 1.99 | 5.7E-06 | 3.6E-04 | #N/A | #N/A |
| DN16506_c2_g4_i1 | -1.77 | 5.80 | 5.9E-06 | 3.7E-04 | #N/A | #N/A |
| DN22624_c4_g1_i2 | -1.31 | 3.63 | 5.9E-06 | 3.7E-04 | MOSA_MAIZE | 1.89E-21 |
| DN15959_c0_g9_i2 | 9.17 | 1.04 | 5.9E-06 | 3.8E-04 | FAS2_ORYSJ | 0 |
| DN20636_c0_g3_i9 | 1.45 | 4.27 | 6.0E-06 | 3.8E-04 | AROG_ORYSJ | 0 |
| DN19388_c0_g2_i3 | 9.37 | 1.24 | 6.0E-06 | 3.8E-04 | GLUA1_ORYSJ | 4.65E-21 |
| DN17010_c0_g1_i2 | -7.86 | -1.07 | 6.1E-06 | 3.9E-04 | GRF5_ORYSJ | 1.15E-123 |
| DN16359_c0_g1_i6 | 10.22 | 2.06 | 6.1E-06 | 3.9E-04 | #N/A | #N/A |
| DN18981_c0_g1_i2 | 9.25 | 1.12 | 6.2E-06 | 3.9E-04 | #N/A | #N/A |
| DN18831_c0_g1_i5 | -1.58 | 3.82 | 6.3E-06 | 4.0E-04 | CRD1_GOSHI | 2.66E-95 |
| DN21290_c0_g3_i3 | -2.37 | 2.82 | 6.3E-06 | 4.0E-04 | #N/A | #N/A |
| DN21808_c2_g1_i12 | -3.14 | 4.74 | 6.3E-06 | 4.0E-04 | #N/A | #N/A |
| DN22331_c0_g1_i4 | 9.04 | 0.92 | 6.3E-06 | 4.0E-04 | RH40_ARATH | 0 |
| DN20824_c1_g1_i11 | -3.39 | 2.16 | 6.4E-06 | 4.0E-04 | #N/A | #N/A |
| DN16092_c1_g2_i7 | 10.41 | 2.24 | 6.4E-06 | 4.1E-04 | GEK1_ARATH | 4.29E-156 |
| DN17918_c1_g2_i7 | -1.83 | 3.67 | 6.5E-06 | 4.1E-04 | #N/A | #N/A |
| DN19159_c0_g1_i8 | 3.12 | 1.24 | 6.6E-06 | 4.1E-04 | PDI52_ORYSJ | 0 |
| DN21763_c0_g1_i8 | -3.17 | 0.71 | 6.6E-06 | 4.1E-04 | #N/A | #N/A |
| DN22141_c0_g1_i17 | 4.72 | 0.36 | 6.6E-06 | 4.1E-04 | SEC6_ARATH | 0 |
| DN22123_c0_g4_i10 | 6.35 | 0.59 | 6.6E-06 | 4.1E-04 | #N/A | #N/A |
| DN19725_c0_g3_i2 | 9.42 | 1.29 | 6.7E-06 | 4.2E-04 | RBL14_ARATH | 1.96E-51 |
| DN22082_c0_g8_i2 | 8.37 | 0.28 | 6.7E-06 | 4.2E-04 | #N/A | #N/A |
| DN22396_c1_g3_i2 | -3.65 | 3.55 | 6.7E-06 | 4.2E-04 | SALT_ORYSJ | 6.92E-33 |
| DN17771_c0_g2_i5 | 8.42 | 0.32 | 6.8E-06 | 4.2E-04 | FAS1_ORYSJ | 1.67E-68 |
| DN15172_c1_g5_i2 | 9.27 | 1.14 | 6.8E-06 | 4.3E-04 | #N/A | #N/A |
| DN18757_c0_g1_i10 | 10.54 | 2.38 | 6.9E-06 | 4.3E-04 | #N/A | #N/A |
| DN20957_c0_g1_i3 | 1.73 | 4.64 | 6.9E-06 | 4.3E-04 | HSP7C_PETHY | 0 |
| DN22791_c0_g1_i2 | -2.87 | 1.14 | 6.9E-06 | 4.3E-04 | #N/A | #N/A |
| DN17502_c0_g2_i1 | 1.58 | 4.33 | 7.0E-06 | 4.3E-04 | TI172_ARATH | 3.48E-68 |
| DN22756_c3_g1_i3 | 8.27 | 0.19 | 7.0E-06 | 4.3E-04 | #N/A | #N/A |
| DN14321_c0_g1_i5 | 9.68 | 1.54 | 7.1E-06 | 4.4E-04 | PB27B_ARATH | 1.84E-45 |
| DN20934_c0_g2_i4 | 5.33 | 0.54 | 7.1E-06 | 4.4E-04 | CAR5_ARATH | 1.69E-23 |
| DN17853_c0_g1_i4 | 3.20 | 3.22 | 7.2E-06 | 4.4E-04 | TLP14_ORYSJ | 0 |
| DN21890_c0_g1_i25 | 8.62 | 0.51 | 7.2E-06 | 4.4E-04 | RHL1_ARATH | 1.16E-55 |
| DN15184_c0_g3_i4 | -7.82 | -1.10 | 7.2E-06 | 4.5E-04 | #N/A | #N/A |
| DN22003_c1_g7_i1 | -1.25 | 5.57 | 7.2E-06 | 4.5E-04 | CIPK7_ORYSJ | 0 |
| DN20948_c0_g2_i1 | 7.37 | 1.56 | 7.2E-06 | 4.5E-04 | #N/A | #N/A |
| DN17677_c0_g2_i1 | 6.32 | 1.85 | 7.3E-06 | 4.5E-04 | SKU5_ARATH | 4.56E-176 |
| DN20997_c0_g1_i2 | 7.69 | -0.34 | 7.4E-06 | 4.5E-04 | ISOA1_ARATH | 3.19E-176 |
| DN18027_c1_g1_i3 | 8.08 | 0.01 | 7.4E-06 | 4.6E-04 | #N/A | #N/A |
| DN18364_c0_g1_i1 | 7.72 | -0.31 | 7.7E-06 | 4.7E-04 | LPE1_MAIZE | 1.04E-42 |
| DN18497_c0_g1_i1 | 8.51 | 0.41 | 7.7E-06 | 4.7E-04 | UGDH5_ORYSJ | 0 |
| DN18977_c2_g4_i5 | 7.92 | -0.13 | 7.8E-06 | 4.8E-04 | IP5PB_ARATH | 9.76E-114 |
| DN16226_c0_g1_i2 | 8.71 | 0.60 | 7.8E-06 | 4.8E-04 | #N/A | #N/A |
| DN18049_c0_g1_i10 | 8.22 | 0.15 | 7.8E-06 | 4.8E-04 | #N/A | #N/A |
| DN18241_c2_g7_i7 | 8.28 | 0.20 | 7.8E-06 | 4.8E-04 | #N/A | #N/A |
| DN18118_c0_g3_i8 | 8.41 | 0.32 | 7.9E-06 | 4.8E-04 | IQD31_ARATH | 9.33E-88 |
| DN22349_c0_g2_i1 | 9.32 | 1.18 | 8.0E-06 | 4.9E-04 | ALG13_RAT | 1.77E-23 |
| DN21209_c1_g1_i3 | 7.78 | -0.26 | 8.1E-06 | 4.9E-04 | #N/A | #N/A |
| DN15986_c1_g1_i18 | 8.88 | 0.76 | 8.1E-06 | 4.9E-04 | DIV_ANTMA | 6.96E-25 |
| DN18751_c0_g5_i3 | 7.86 | -0.19 | 8.1E-06 | 4.9E-04 | #N/A | #N/A |
| DN13344_c0_g1_i4 | 8.71 | 0.60 | 8.2E-06 | 4.9E-04 | PLCD2_ARATH | 1.28E-110 |
| DN14686_c0_g2_i2 | 8.77 | 0.66 | 8.2E-06 | 4.9E-04 | #N/A | #N/A |
| DN18572_c0_g1_i12 | 10.21 | 2.05 | 8.3E-06 | 5.0E-04 | AB25B_ORYSJ | 7.51E-149 |
| DN17206_c0_g2_i2 | -0.95 | 6.57 | 8.4E-06 | 5.1E-04 | DIN1_RAPSA | 8.58E-41 |
| DN21891_c1_g1_i7 | 8.28 | 0.20 | 8.4E-06 | 5.1E-04 | #N/A | #N/A |
| DN20469_c0_g2_i10 | 7.34 | 3.22 | 8.5E-06 | 5.1E-04 | ASPRX_ORYSJ | 0 |
| DN20444_c0_g2_i3 | 8.98 | 0.86 | 8.7E-06 | 5.2E-04 | #N/A | #N/A |
| DN17037_c0_g1_i1 | 8.84 | 0.73 | 8.8E-06 | 5.3E-04 | #N/A | #N/A |
| DN17450_c2_g2_i11 | 8.97 | 0.85 | 8.9E-06 | 5.3E-04 | SPPA1_ARATH | 0 |
| DN15367_c0_g8_i1 | 8.70 | 3.67 | 8.9E-06 | 5.3E-04 | #N/A | #N/A |
| DN14843_c0_g3_i2 | -2.18 | 2.39 | 8.9E-06 | 5.4E-04 | #N/A | #N/A |
| DN17903_c0_g2_i7 | 8.56 | 0.46 | 9.0E-06 | 5.4E-04 | #N/A | #N/A |
| DN19347_c0_g1_i2 | -1.84 | 3.53 | 9.1E-06 | 5.4E-04 | ALA2_PANMI | 2.36E-86 |
| DN22439_c1_g2_i5 | -3.10 | 0.61 | 9.1E-06 | 5.5E-04 | PSBO_WHEAT | 3.58E-41 |
| DN15829_c2_g4_i2 | 7.88 | -0.17 | 9.2E-06 | 5.5E-04 | #N/A | #N/A |
| DN18418_c0_g1_i15 | 9.17 | 1.04 | 9.2E-06 | 5.5E-04 | CHS1_MAIZE | 2.67E-35 |
| DN22801_c2_g2_i4 | 8.42 | 0.33 | 9.2E-06 | 5.5E-04 | #N/A | #N/A |
| DN18803_c0_g1_i9 | 9.93 | 1.78 | 9.3E-06 | 5.6E-04 | #N/A | #N/A |
| DN20271_c0_g1_i1 | 9.03 | 0.90 | 9.4E-06 | 5.6E-04 | Y005_SYNY3 | 6.03E-86 |
| DN19103_c0_g1_i4 | 9.06 | 0.93 | 9.5E-06 | 5.7E-04 | MYOB2_ARATH | 7.08E-52 |
| DN17027_c0_g1_i1 | -2.32 | 3.10 | 9.6E-06 | 5.7E-04 | #N/A | #N/A |
| DN20285_c0_g1_i20 | 7.82 | -0.22 | 9.6E-06 | 5.7E-04 | PSF2_ARATH | 5.53E-35 |
| DN20039_c1_g2_i11 | 6.49 | 1.52 | 9.8E-06 | 5.8E-04 | RNP1_ARATH | 2.63E-51 |
| DN14927_c1_g2_i6 | 7.71 | -0.33 | 9.8E-06 | 5.8E-04 | #N/A | #N/A |
| DN18432_c0_g1_i17 | -1.67 | 5.79 | 1.0E-05 | 5.9E-04 | MCA1_ARATH | 1.56E-86 |
| DN21506_c1_g1_i3 | 8.67 | 0.56 | 1.0E-05 | 5.9E-04 | NUP88_ARATH | 6.2E-180 |
| DN17754_c0_g2_i1 | 7.79 | -0.25 | 1.0E-05 | 5.9E-04 | #N/A | #N/A |
| DN12852_c0_g1_i2 | 1.71 | 4.04 | 1.0E-05 | 5.9E-04 | #N/A | #N/A |
| DN14831_c0_g1_i2 | 8.16 | 0.09 | 1.0E-05 | 5.9E-04 | #N/A | #N/A |
| DN22154_c0_g1_i3 | 8.27 | 0.19 | 1.0E-05 | 6.0E-04 | #N/A | #N/A |
| DN18927_c1_g3_i1 | -1.71 | 6.71 | 1.0E-05 | 6.1E-04 | #N/A | #N/A |
| DN16629_c1_g1_i4 | -1.01 | 5.97 | 1.0E-05 | 6.1E-04 | PFPA_RICCO | 2.42E-118 |
| DN20713_c0_g2_i8 | 8.52 | 0.42 | 1.0E-05 | 6.2E-04 | RPN2_ORYSJ | 0 |
| DN17872_c1_g6_i7 | -1.41 | 4.47 | 1.1E-05 | 6.2E-04 | #N/A | #N/A |
| DN17373_c0_g2_i5 | 3.05 | 1.56 | 1.1E-05 | 6.2E-04 | #N/A | #N/A |
| DN17539_c1_g1_i9 | 9.27 | 1.14 | 1.1E-05 | 6.2E-04 | AP2_ARATH | 3.03E-85 |
| DN16124_c0_g1_i14 | -1.03 | 5.03 | 1.1E-05 | 6.2E-04 | TRL12_ORYSJ | 1.12E-101 |
| DN21011_c2_g1_i1 | -1.52 | 3.31 | 1.1E-05 | 6.3E-04 | #N/A | #N/A |
| DN22006_c2_g2_i13 | -1.19 | 7.19 | 1.1E-05 | 6.3E-04 | PPDK1_MAIZE | 9.3E-22 |
| DN21994_c0_g1_i13 | 9.42 | 1.28 | 1.1E-05 | 6.3E-04 | #N/A | #N/A |
| DN18148_c0_g1_i4 | 7.81 | -0.23 | 1.1E-05 | 6.3E-04 | #N/A | #N/A |
| DN21128_c3_g3_i6 | 2.43 | 3.55 | 1.1E-05 | 6.3E-04 | #N/A | #N/A |
| DN16777_c0_g1_i2 | 7.79 | 1.97 | 1.1E-05 | 6.4E-04 | #N/A | #N/A |
| DN21114_c0_g2_i23 | 11.31 | 3.13 | 1.1E-05 | 6.4E-04 | HEM6_ORYSJ | 0 |
| DN18158_c5_g2_i1 | 9.88 | 1.73 | 1.1E-05 | 6.4E-04 | UGAL1_ARATH | 0 |
| DN18070_c1_g1_i23 | 1.16 | 6.56 | 1.1E-05 | 6.5E-04 | ACSS_MAIZE | 0 |
| DN18348_c3_g3_i1 | 8.64 | 0.54 | 1.1E-05 | 6.6E-04 | GAUT8_ARATH | 0 |
| DN20141_c3_g1_i8 | 9.13 | 1.00 | 1.1E-05 | 6.6E-04 | POD1_ARATH | 1.25E-156 |
| DN19018_c0_g3_i15 | 8.29 | 0.21 | 1.1E-05 | 6.6E-04 | #N/A | #N/A |
| DN14795_c0_g1_i7 | 1.65 | 3.26 | 1.1E-05 | 6.6E-04 | LECH_HORVU | 1.58E-25 |
| DN17931_c0_g3_i6 | 7.84 | -0.21 | 1.1E-05 | 6.6E-04 | #N/A | #N/A |
| DN17415_c0_g1_i3 | 12.00 | 3.82 | 1.1E-05 | 6.6E-04 | #N/A | #N/A |
| DN18585_c0_g1_i8 | 8.17 | 0.09 | 1.2E-05 | 6.7E-04 | #N/A | #N/A |
| DN16636_c2_g1_i7 | -3.87 | 0.36 | 1.2E-05 | 6.8E-04 | #N/A | #N/A |
| DN19516_c0_g4_i2 | -1.33 | 4.14 | 1.2E-05 | 6.8E-04 | IAA17_ORYSJ | 1.49E-92 |
| DN22536_c0_g2_i10 | 8.10 | 0.03 | 1.2E-05 | 6.9E-04 | P2C43_ORYSJ | 8.85E-22 |
| DN19841_c1_g9_i2 | 8.45 | 0.36 | 1.2E-05 | 6.9E-04 | #N/A | #N/A |
| DN17834_c0_g2_i2 | 3.11 | 3.59 | 1.2E-05 | 6.9E-04 | LCAT1_ARATH | 2.18E-93 |
| DN16384_c0_g4_i1 | 8.61 | 0.51 | 1.2E-05 | 6.9E-04 | RNP1_ARATH | 6.03E-40 |
| DN20425_c0_g1_i5 | 9.17 | 1.04 | 1.2E-05 | 6.9E-04 | #N/A | #N/A |
| DN18241_c2_g10_i2 | 8.24 | 0.16 | 1.2E-05 | 6.9E-04 | #N/A | #N/A |
| DN19953_c0_g1_i1 | 3.68 | 3.34 | 1.2E-05 | 7.0E-04 | #N/A | #N/A |
| DN19496_c0_g3_i9 | 3.86 | 0.24 | 1.3E-05 | 7.2E-04 | #N/A | #N/A |
| DN15952_c0_g2_i7 | 3.36 | 0.97 | 1.3E-05 | 7.2E-04 | HSP23_MAIZE | 1.06E-87 |
| DN22184_c0_g1_i1 | -1.09 | 4.72 | 1.3E-05 | 7.2E-04 | #N/A | #N/A |
| DN16311_c0_g2_i2 | -1.38 | 4.74 | 1.3E-05 | 7.3E-04 | YCF3_SACHY | 1.33E-119 |
| DN19163_c1_g1_i2 | 8.77 | 0.66 | 1.3E-05 | 7.3E-04 | RFA3_ORYSJ | 2.35E-38 |
| DN18226_c0_g1_i1 | 3.29 | 1.69 | 1.3E-05 | 7.5E-04 | GPT2_ARATH | 2.67E-82 |
| DN22700_c4_g3_i9 | 8.81 | 0.70 | 1.3E-05 | 7.5E-04 | HFA2E_ORYSJ | 8.92E-111 |
| DN21756_c0_g1_i8 | 4.50 | 1.59 | 1.3E-05 | 7.6E-04 | EIF3E_ARATH | 1.81E-22 |
| DN18209_c1_g4_i2 | 7.76 | -0.28 | 1.4E-05 | 7.8E-04 | #N/A | #N/A |
| DN20554_c2_g2_i4 | 9.63 | 1.49 | 1.4E-05 | 7.9E-04 | #N/A | #N/A |
| DN14155_c0_g1_i4 | 1.51 | 4.41 | 1.4E-05 | 7.9E-04 | C16B1_PICSI | 1.04E-105 |
| DN21229_c0_g2_i17 | 2.69 | 2.85 | 1.4E-05 | 7.9E-04 | Y4370_ARATH | 7.54E-40 |
| DN22062_c0_g1_i16 | 10.92 | 2.75 | 1.4E-05 | 7.9E-04 | MIRO1_ARATH | 0 |
| DN20429_c0_g2_i1 | 8.87 | 0.75 | 1.4E-05 | 8.1E-04 | ACT_PINCO | 6.27E-25 |
| DN15405_c3_g4_i1 | 8.61 | 0.51 | 1.4E-05 | 8.1E-04 | FB270_ARATH | 4.08E-74 |
| DN13126_c0_g1_i5 | 7.88 | -0.17 | 1.4E-05 | 8.2E-04 | ARP6_ORYSJ | 2.02E-131 |
| DN18608_c1_g1_i2 | 5.83 | 0.90 | 1.5E-05 | 8.2E-04 | IP5PA_ARATH | 1.9E-151 |
| DN22076_c1_g1_i8 | 2.79 | 4.73 | 1.5E-05 | 8.5E-04 | #N/A | #N/A |
| DN17948_c0_g1_i5 | 2.16 | 4.40 | 1.5E-05 | 8.5E-04 | MORF8_ARATH | 1.93E-72 |
| DN15690_c1_g2_i10 | 7.80 | -0.24 | 1.5E-05 | 8.6E-04 | #N/A | #N/A |
| DN18936_c0_g2_i7 | 7.86 | -0.18 | 1.6E-05 | 8.8E-04 | Y1561_ARATH | 3.83E-70 |
| DN12195_c0_g1_i1 | 7.67 | -0.36 | 1.6E-05 | 8.8E-04 | FLS_PETHY | 6.87E-128 |
| DN14927_c1_g2_i11 | 8.50 | 0.41 | 1.6E-05 | 8.9E-04 | #N/A | #N/A |
| DN17828_c0_g1_i2 | 1.71 | 4.94 | 1.6E-05 | 9.0E-04 | BGL14_ORYSJ | 0 |
| DN20679_c0_g1_i14 | 5.91 | 1.46 | 1.6E-05 | 9.0E-04 | #N/A | #N/A |
| DN18431_c0_g1_i12 | 8.42 | 0.33 | 1.6E-05 | 9.0E-04 | #N/A | #N/A |
| DN22284_c0_g2_i1 | -1.60 | 4.80 | 1.6E-05 | 9.1E-04 | #N/A | #N/A |
| DN19987_c1_g3_i5 | -1.94 | 3.03 | 1.6E-05 | 9.1E-04 | ACS_ARATH | 0 |
| DN19197_c0_g3_i7 | 8.24 | 0.16 | 1.6E-05 | 9.1E-04 | #N/A | #N/A |
| DN17307_c1_g1_i12 | -1.39 | 3.82 | 1.6E-05 | 9.2E-04 | #N/A | #N/A |
| DN14979_c0_g8_i1 | 9.66 | 1.51 | 1.7E-05 | 9.3E-04 | ZCIS_MAIZE | 1.58E-147 |
| DN20088_c0_g2_i10 | -8.07 | 2.95 | 1.7E-05 | 9.3E-04 | AGD5_ARATH | 9.69E-116 |
| DN16667_c1_g3_i20 | 3.43 | 4.10 | 1.7E-05 | 9.6E-04 | PITP1_DICDI | 4.38E-57 |
| DN10655_c0_g1_i4 | 1.56 | 3.70 | 1.8E-05 | 9.9E-04 | DLO2_ARATH | 1.04E-51 |
| DN22794_c3_g4_i1 | 1.60 | 3.44 | 1.8E-05 | 1.0E-03 | HPPD_HORVU | 8.07E-26 |
| DN18565_c0_g1_i18 | 10.51 | 2.34 | 1.8E-05 | 1.0E-03 | CKX4_ORYSJ | 0 |
| DN21046_c0_g2_i2 | 2.92 | 1.29 | 1.8E-05 | 1.0E-03 | #N/A | #N/A |
| DN21391_c0_g1_i18 | 7.86 | -0.19 | 1.9E-05 | 1.0E-03 | #N/A | #N/A |
| DN21944_c0_g1_i8 | 8.79 | 0.68 | 1.9E-05 | 1.0E-03 | SDR1_ARATH | 6.9E-33 |
| DN20642_c0_g1_i14 | -1.75 | 3.96 | 1.9E-05 | 1.0E-03 | #N/A | #N/A |
| DN16302_c0_g5_i1 | 3.94 | 0.77 | 1.9E-05 | 1.0E-03 | UGT2_GARJA | 5.49E-167 |
| DN20686_c1_g1_i5 | 1.11 | 5.22 | 1.9E-05 | 1.1E-03 | NEET_ARATH | 9.52E-33 |
| DN21423_c0_g1_i16 | 4.48 | 1.52 | 1.9E-05 | 1.1E-03 | SRK2E_ARATH | 1.41E-133 |
| DN15555_c1_g4_i5 | -4.66 | -0.23 | 2.0E-05 | 1.1E-03 | TL203_ARATH | 1.38E-80 |
| DN17662_c2_g9_i1 | -2.21 | 2.51 | 2.0E-05 | 1.1E-03 | IFRH_SOLTU | 1.27E-44 |
| DN19637_c1_g3_i4 | 9.46 | 1.32 | 2.0E-05 | 1.1E-03 | P2C50_ORYSJ | 6.04E-85 |
| DN16099_c2_g1_i6 | 3.71 | 2.34 | 2.0E-05 | 1.1E-03 | #N/A | #N/A |
| DN18856_c0_g5_i1 | -1.78 | 2.40 | 2.0E-05 | 1.1E-03 | #N/A | #N/A |
| DN15986_c1_g1_i21 | 9.24 | 1.11 | 2.0E-05 | 1.1E-03 | DIV_ANTMA | 1.09E-25 |
| DN21640_c1_g1_i5 | 1.05 | 5.08 | 2.0E-05 | 1.1E-03 | PUX3_ARATH | 8.93E-79 |
| DN15673_c0_g1_i4 | 9.43 | 1.29 | 2.0E-05 | 1.1E-03 | #N/A | #N/A |
| DN13135_c0_g1_i2 | -1.11 | 4.70 | 2.1E-05 | 1.1E-03 | #N/A | #N/A |
| DN18441_c0_g1_i7 | -5.40 | 0.66 | 2.1E-05 | 1.1E-03 | KMS1_ARATH | 4.47E-135 |
| DN19524_c1_g1_i12 | 7.54 | -0.48 | 2.1E-05 | 1.1E-03 | #N/A | #N/A |
| DN20607_c1_g3_i7 | 7.54 | -0.47 | 2.1E-05 | 1.2E-03 | #N/A | #N/A |
| DN15465_c2_g1_i4 | 8.89 | 0.78 | 2.1E-05 | 1.2E-03 | YODA_ARATH | 1.33E-61 |
| DN18291_c0_g2_i12 | 8.66 | 0.55 | 2.2E-05 | 1.2E-03 | TLP9_ORYSJ | 3.09E-107 |
| DN19324_c0_g1_i2 | -4.04 | -0.35 | 2.2E-05 | 1.2E-03 | GEML8_ARATH | 1.57E-39 |
| DN14659_c0_g1_i6 | 9.46 | 1.32 | 2.2E-05 | 1.2E-03 | SAT5_ORYSJ | 2.89E-136 |
| DN14758_c0_g1_i6 | 7.76 | -0.28 | 2.2E-05 | 1.2E-03 | #N/A | #N/A |
| DN17007_c0_g1_i10 | 8.83 | 0.72 | 2.2E-05 | 1.2E-03 | SAMH1_CHICK | 1.51E-34 |
| DN18980_c0_g1_i10 | 12.07 | 3.89 | 2.2E-05 | 1.2E-03 | SCAM6_ORYSJ | 0 |
| DN17239_c2_g4_i3 | 5.54 | 2.36 | 2.2E-05 | 1.2E-03 | DCAF8_MOUSE | 6.55E-80 |
| DN21890_c0_g1_i19 | 7.95 | -0.10 | 2.2E-05 | 1.2E-03 | RHL1_ARATH | 1.09E-55 |
| DN19559_c0_g1_i2 | 8.03 | -0.03 | 2.2E-05 | 1.2E-03 | SKI15_ARATH | 1.04E-83 |
| DN14092_c0_g1_i2 | 7.96 | -0.10 | 2.2E-05 | 1.2E-03 | DGP14_ARATH | 2.01E-136 |
| DN20994_c0_g4_i1 | 8.04 | -0.02 | 2.2E-05 | 1.2E-03 | TPS10_ARATH | 2.11E-148 |
| DN16509_c1_g1_i2 | 4.65 | 1.15 | 2.3E-05 | 1.2E-03 | GENL2_ORYSJ | 0 |
| DN15213_c1_g5_i1 | -1.29 | 3.99 | 2.3E-05 | 1.3E-03 | U83A1_ARATH | 4.13E-113 |
| DN18873_c0_g6_i1 | 3.37 | 0.81 | 2.3E-05 | 1.3E-03 | #N/A | #N/A |
| DN21011_c2_g1_i2 | 2.33 | 1.75 | 2.3E-05 | 1.3E-03 | #N/A | #N/A |
| DN22439_c1_g4_i1 | -1.05 | 9.45 | 2.4E-05 | 1.3E-03 | PSBO_TOBAC | 3.91E-60 |
| DN19400_c1_g1_i8 | 8.26 | 0.18 | 2.4E-05 | 1.3E-03 | #N/A | #N/A |
| DN15121_c0_g8_i3 | -1.47 | 6.16 | 2.4E-05 | 1.3E-03 | #N/A | #N/A |
| DN22308_c0_g3_i1 | -1.19 | 4.46 | 2.4E-05 | 1.3E-03 | COL16_ARATH | 7.96E-20 |
| DN14080_c0_g1_i2 | 9.38 | 1.24 | 2.4E-05 | 1.3E-03 | Y4833_ARATH | 9.99E-31 |
| DN14545_c0_g1_i5 | 1.49 | 4.62 | 2.4E-05 | 1.3E-03 | PHLB_PHLPR | 2.92E-59 |
| DN18508_c0_g2_i1 | 8.74 | 0.63 | 2.4E-05 | 1.3E-03 | LWD1_ARATH | 1.03E-172 |
| DN15829_c2_g1_i23 | 8.10 | 0.03 | 2.4E-05 | 1.3E-03 | #N/A | #N/A |
| DN20831_c1_g3_i1 | 1.58 | 5.82 | 2.5E-05 | 1.3E-03 | DRG3_ARATH | 0 |
| DN22301_c1_g1_i1 | 9.00 | 0.88 | 2.5E-05 | 1.3E-03 | #N/A | #N/A |
| DN21438_c0_g3_i3 | 8.31 | 0.23 | 2.5E-05 | 1.3E-03 | SBDS_MOUSE | 4.58E-59 |
| DN15180_c0_g1_i4 | -5.63 | -0.96 | 2.5E-05 | 1.3E-03 | #N/A | #N/A |
| DN20078_c0_g1_i6 | -0.89 | 10.38 | 2.5E-05 | 1.3E-03 | LOX23_HORVU | 0 |
| DN16532_c1_g4_i2 | 8.82 | 0.71 | 2.5E-05 | 1.4E-03 | #N/A | #N/A |
| DN17387_c4_g1_i13 | 8.21 | 0.13 | 2.6E-05 | 1.4E-03 | #N/A | #N/A |
| DN36404_c0_g1_i1 | 3.08 | 3.38 | 2.6E-05 | 1.4E-03 | **RHM1_ARATH** | 0 |
| DN16836_c3_g2_i3 | 7.58 | -0.44 | 2.6E-05 | 1.4E-03 | TIM50_ARATH | 2.23E-46 |
| DN22798_c2_g1_i5 | 7.68 | -0.35 | 2.6E-05 | 1.4E-03 | BH148_ORYSJ | 3.34E-70 |
| DN16866_c1_g4_i4 | 7.74 | -0.30 | 2.6E-05 | 1.4E-03 | DCMC_HUMAN | 1.4E-67 |
| DN16783_c3_g2_i5 | 2.01 | 5.84 | 2.6E-05 | 1.4E-03 | #N/A | #N/A |
| DN21597_c2_g6_i2 | -1.73 | 3.04 | 2.7E-05 | 1.4E-03 | #N/A | #N/A |
| DN21183_c4_g2_i3 | 9.05 | 0.92 | 2.7E-05 | 1.4E-03 | #N/A | #N/A |
| DN19082_c0_g1_i15 | 5.60 | -0.03 | 2.7E-05 | 1.4E-03 | #N/A | #N/A |
| DN16266_c0_g1_i17 | 7.75 | -0.28 | 2.8E-05 | 1.5E-03 | MLO9_ARATH | 9.52E-88 |
| DN21164_c0_g1_i17 | 9.41 | 1.27 | 2.8E-05 | 1.5E-03 | SFR2_ORYSJ | 0 |
| DN15015_c0_g1_i2 | -1.78 | 4.31 | 2.8E-05 | 1.5E-03 | #N/A | #N/A |
| DN5393_c0_g1_i1 | 7.59 | -0.43 | 2.8E-05 | 1.5E-03 | #N/A | #N/A |
| DN20721_c1_g1_i1 | -1.15 | 7.59 | 2.9E-05 | 1.5E-03 | #N/A | #N/A |
| DN21736_c0_g1_i11 | -2.21 | 1.50 | 2.9E-05 | 1.5E-03 | COQ5_ORYSJ | 1.51E-24 |
| DN14605_c0_g1_i5 | 7.56 | -0.46 | 2.9E-05 | 1.5E-03 | QORH_ARATH | 2.45E-91 |
| DN15535_c1_g7_i1 | 8.25 | 0.18 | 3.0E-05 | 1.6E-03 | **RHM1_ARATH** | 0 |
| DN19294_c1_g1_i15 | 3.76 | 1.45 | 3.0E-05 | 1.6E-03 | CSK2A_MAIZE | 3.46E-113 |
| DN20953_c0_g1_i24 | 1.52 | 6.57 | 3.0E-05 | 1.6E-03 | ELI9_HORVU | 1.61E-37 |
| DN17172_c0_g3_i7 | -1.12 | 5.84 | 3.1E-05 | 1.6E-03 | IP5P2_ARATH | 6.29E-169 |
| DN16098_c3_g2_i5 | 8.01 | 2.99 | 3.1E-05 | 1.6E-03 | SDAF2_ARATH | 1.39E-61 |
| DN15715_c0_g3_i2 | 7.53 | -0.48 | 3.1E-05 | 1.6E-03 | #N/A | #N/A |
| DN21876_c0_g1_i2 | 8.84 | 0.72 | 3.1E-05 | 1.6E-03 | TNPO1_ORYSJ | 0 |
| DN16558_c0_g2_i2 | 8.63 | 0.52 | 3.1E-05 | 1.6E-03 | #N/A | #N/A |
| DN20932_c0_g1_i4 | 3.56 | 2.56 | 3.2E-05 | 1.7E-03 | SYCM_ARATH | 0 |
| DN15041_c0_g1_i1 | 6.72 | 0.94 | 3.2E-05 | 1.7E-03 | PDX11_ORYSJ | 7.21E-51 |
| DN16150_c0_g3_i6 | -1.15 | 8.10 | 3.2E-05 | 1.7E-03 | SALR_PAPBR | 9.46E-60 |
| DN14719_c0_g1_i11 | -2.12 | 2.04 | 3.2E-05 | 1.7E-03 | AAP8_ARATH | 1.86E-163 |
| DN22012_c1_g2_i26 | -0.97 | 6.11 | 3.3E-05 | 1.7E-03 | #N/A | #N/A |
| DN21808_c2_g1_i15 | -1.98 | 3.07 | 3.3E-05 | 1.7E-03 | #N/A | #N/A |
| DN20738_c1_g1_i16 | 7.55 | -0.47 | 3.3E-05 | 1.7E-03 | Y3028_ARATH | 8.78E-53 |
| DN21771_c1_g1_i17 | 2.16 | 2.58 | 3.3E-05 | 1.7E-03 | #N/A | #N/A |
| DN18493_c0_g2_i1 | 7.86 | -0.19 | 3.3E-05 | 1.7E-03 | #N/A | #N/A |
| DN20885_c0_g1_i1 | 2.92 | 4.56 | 3.3E-05 | 1.7E-03 | #N/A | #N/A |
| DN15671_c0_g4_i2 | -1.40 | 5.02 | 3.4E-05 | 1.7E-03 | #N/A | #N/A |
| DN18319_c0_g1_i9 | -1.45 | 4.05 | 3.4E-05 | 1.7E-03 | C3H63_ORYSJ | 0 |
| DN17905_c1_g1_i6 | 9.06 | 0.94 | 3.4E-05 | 1.8E-03 | LHW_ARATH | 7.66E-56 |
| DN19544_c1_g2_i4 | -1.03 | 6.58 | 3.5E-05 | 1.8E-03 | #N/A | #N/A |
| DN20732_c0_g1_i18 | 11.70 | 3.52 | 3.5E-05 | 1.8E-03 | NIP22_MAIZE | 0 |
| DN20210_c2_g2_i11 | -2.04 | 2.05 | 3.5E-05 | 1.8E-03 | CKX11_ORYSJ | 0 |
| DN17148_c1_g2_i10 | 10.75 | 2.59 | 3.5E-05 | 1.8E-03 | #N/A | #N/A |
| DN19163_c1_g1_i5 | 8.49 | 0.39 | 3.6E-05 | 1.8E-03 | RFA3_ORYSJ | 1.31E-37 |
| DN20789_c0_g3_i11 | 8.61 | 0.50 | 3.6E-05 | 1.9E-03 | MAN7_ORYSJ | 1.23E-157 |
| DN18413_c0_g1_i8 | 8.80 | 0.69 | 3.6E-05 | 1.9E-03 | Y3544_ARATH | 7.77E-40 |
| DN17238_c0_g1_i2 | 1.14 | 5.50 | 3.7E-05 | 1.9E-03 | #N/A | #N/A |
| DN20261_c0_g1_i8 | 3.10 | 1.05 | 3.7E-05 | 1.9E-03 | ACA8_ARATH | 0 |
| DN20007_c0_g1_i16 | 9.28 | 1.14 | 3.7E-05 | 1.9E-03 | MNS1_ARATH | 0 |
| DN21009_c2_g1_i7 | 1.68 | 3.05 | 3.7E-05 | 1.9E-03 | #N/A | #N/A |
| DN15886_c2_g1_i14 | -1.17 | 4.28 | 3.8E-05 | 1.9E-03 | #N/A | #N/A |
| DN16786_c1_g2_i9 | 7.82 | -0.22 | 3.9E-05 | 2.0E-03 | UVB31_ARATH | 2.8E-49 |
| DN16636_c2_g1_i1 | -2.09 | 7.05 | 3.9E-05 | 2.0E-03 | #N/A | #N/A |
| DN19859_c0_g1_i6 | 5.58 | -0.05 | 3.9E-05 | 2.0E-03 | #N/A | #N/A |
| DN17019_c1_g2_i3 | -2.04 | 3.68 | 3.9E-05 | 2.0E-03 | FLP4_ORYSJ | 4.58E-39 |
| DN16876_c2_g4_i3 | 7.76 | -0.28 | 3.9E-05 | 2.0E-03 | #N/A | #N/A |
| DN15796_c2_g3_i7 | 7.47 | -0.53 | 3.9E-05 | 2.0E-03 | #N/A | #N/A |
| DN17979_c0_g3_i2 | 8.82 | 0.70 | 3.9E-05 | 2.0E-03 | CFM2_ARATH | 1.51E-83 |
| DN19125_c0_g1_i1 | 9.16 | 1.03 | 3.9E-05 | 2.0E-03 | TRA1_MAIZE | 1.35E-34 |
| DN16051_c0_g7_i2 | -1.18 | 5.36 | 3.9E-05 | 2.0E-03 | C70B2_ARATH | 3.21E-161 |
| DN18897_c0_g1_i6 | 7.72 | -0.32 | 4.0E-05 | 2.0E-03 | C74A1_ORYSJ | 0 |
| DN11500_c0_g1_i1 | -4.49 | 0.73 | 4.0E-05 | 2.0E-03 | #N/A | #N/A |
| DN18856_c0_g2_i10 | 1.07 | 6.28 | 4.0E-05 | 2.0E-03 | #N/A | #N/A |
| DN19722_c0_g2_i6 | 2.66 | 1.03 | 4.0E-05 | 2.0E-03 | #N/A | #N/A |
| DN10425_c0_g1_i1 | 1.48 | 4.95 | 4.1E-05 | 2.1E-03 | HS24M_ORYSJ | 2.6E-81 |
| DN21626_c3_g5_i6 | 9.70 | 1.55 | 4.1E-05 | 2.1E-03 | #N/A | #N/A |
| DN16216_c0_g5_i14 | 8.19 | 0.12 | 4.1E-05 | 2.1E-03 | #N/A | #N/A |
| DN18255_c0_g3_i4 | 7.95 | -0.11 | 4.1E-05 | 2.1E-03 | BH113_ARATH | 1.49E-27 |
| DN21952_c2_g2_i12 | 6.01 | 2.20 | 4.1E-05 | 2.1E-03 | #N/A | #N/A |
| DN20466_c2_g3_i2 | 8.71 | 0.60 | 4.1E-05 | 2.1E-03 | #N/A | #N/A |
| DN21034_c1_g4_i1 | 8.96 | 0.84 | 4.1E-05 | 2.1E-03 | BPA1_ARATH | 3.03E-61 |
| DN20399_c0_g1_i9 | 7.99 | -0.07 | 4.2E-05 | 2.1E-03 | TELO2_HUMAN | 4.1E-26 |
| DN17679_c1_g4_i1 | 4.11 | 2.90 | 4.2E-05 | 2.1E-03 | FBK28_ARATH | 1.77E-78 |
| DN17364_c1_g6_i2 | -1.42 | 2.93 | 4.3E-05 | 2.1E-03 | #N/A | #N/A |
| DN18611_c0_g1_i3 | 8.48 | 2.63 | 4.3E-05 | 2.2E-03 | #N/A | #N/A |
| DN19509_c0_g3_i1 | -1.24 | 4.16 | 4.4E-05 | 2.2E-03 | #N/A | #N/A |
| DN21490_c0_g5_i4 | 2.48 | 1.26 | 4.4E-05 | 2.2E-03 | #N/A | #N/A |
| DN21928_c1_g5_i9 | -1.52 | 3.50 | 4.4E-05 | 2.2E-03 | IMDH_VIGUN | 0 |
| DN17144_c0_g2_i14 | 9.25 | 1.11 | 4.4E-05 | 2.2E-03 | #N/A | #N/A |
| DN14675_c0_g1_i2 | -2.57 | 0.34 | 4.5E-05 | 2.2E-03 | #N/A | #N/A |
| DN18281_c0_g1_i2 | 8.53 | 0.44 | 4.5E-05 | 2.3E-03 | #N/A | #N/A |
| DN20864_c0_g3_i2 | 8.35 | 0.27 | 4.6E-05 | 2.3E-03 | #N/A | #N/A |
| DN19803_c0_g5_i4 | 7.52 | -0.50 | 4.6E-05 | 2.3E-03 | Y5129_ARATH | 3.65E-116 |
| DN17090_c0_g1_i2 | 1.10 | 4.38 | 4.7E-05 | 2.3E-03 | #N/A | #N/A |
| DN17313_c0_g3_i1 | 7.73 | -0.30 | 4.7E-05 | 2.4E-03 | #N/A | #N/A |
| DN21355_c1_g2_i9 | 8.87 | 0.75 | 4.8E-05 | 2.4E-03 | ITPK3_ORYSJ | 3.23E-77 |
| DN18714_c0_g1_i1 | 9.84 | 1.69 | 4.8E-05 | 2.4E-03 | #N/A | #N/A |
| DN15967_c0_g2_i2 | 0.88 | 7.23 | 4.8E-05 | 2.4E-03 | #N/A | #N/A |
| DN18470_c1_g1_i14 | 10.92 | 2.75 | 4.9E-05 | 2.4E-03 | MTP1_ORYSJ | 4.94E-81 |
| DN18848_c1_g1_i14 | 8.28 | 0.20 | 4.9E-05 | 2.4E-03 | #N/A | #N/A |
| DN18578_c0_g1_i5 | 8.47 | 0.37 | 5.0E-05 | 2.5E-03 | APG_ORYSJ | 5.56E-25 |
| DN19635_c0_g2_i2 | -0.98 | 8.72 | 5.0E-05 | 2.5E-03 | KADC_MAIZE | 6.83E-159 |
| DN21100_c0_g3_i2 | -1.79 | 7.02 | 5.1E-05 | 2.5E-03 | HD3A_ORYSJ | 5.42E-103 |
| DN19347_c0_g1_i19 | -2.41 | 1.92 | 5.1E-05 | 2.5E-03 | ALA2_PANMI | 0 |
| DN15384_c0_g1_i1 | 8.83 | 0.71 | 5.1E-05 | 2.5E-03 | RMT2_SCHPO | 3.75E-50 |
| DN22392_c0_g1_i1 | 9.53 | 1.39 | 5.1E-05 | 2.5E-03 | NLP2_ORYSJ | 0 |
| DN12365_c0_g1_i3 | -1.99 | 1.68 | 5.1E-05 | 2.5E-03 | Y1669_ARATH | 3.4E-179 |
| DN18156_c1_g2_i6 | 3.57 | 0.43 | 5.2E-05 | 2.5E-03 | ACR9_ARATH | 1.53E-135 |
| DN20675_c0_g1_i3 | 8.53 | 0.43 | 5.2E-05 | 2.6E-03 | #N/A | #N/A |
| DN18075_c3_g1_i4 | -6.15 | -0.52 | 5.2E-05 | 2.6E-03 | #N/A | #N/A |
| DN16895_c0_g1_i2 | 4.83 | 0.45 | 5.2E-05 | 2.6E-03 | ENT4_ARATH | 7.81E-125 |
| DN22149_c0_g1_i1 | 6.06 | 3.17 | 5.3E-05 | 2.6E-03 | PGMC1_MAIZE | 0 |
| DN16470_c2_g1_i3 | 7.60 | -0.42 | 5.4E-05 | 2.6E-03 | RNE_ARATH | 8.67E-58 |
| DN19320_c1_g1_i28 | 1.44 | 3.70 | 5.4E-05 | 2.6E-03 | #N/A | #N/A |
| DN16434_c0_g1_i5 | 7.94 | -0.12 | 5.4E-05 | 2.6E-03 | #N/A | #N/A |
| DN15707_c0_g1_i1 | 7.97 | -0.08 | 5.4E-05 | 2.6E-03 | #N/A | #N/A |
| DN16715_c0_g2_i1 | 3.99 | 1.26 | 5.4E-05 | 2.6E-03 | DNAJ_METFK | 1.8E-30 |
| DN17423_c2_g5_i3 | 1.18 | 4.49 | 5.4E-05 | 2.7E-03 | MSRB3_ORYSJ | 1.28E-79 |
| DN19180_c0_g2_i3 | 9.66 | 1.52 | 5.5E-05 | 2.7E-03 | #N/A | #N/A |
| DN19852_c0_g2_i10 | 9.08 | 0.95 | 5.6E-05 | 2.8E-03 | #N/A | #N/A |
| DN19213_c2_g2_i1 | 5.55 | -0.07 | 5.7E-05 | 2.8E-03 | #N/A | #N/A |
| DN21950_c0_g2_i7 | 8.04 | -0.03 | 5.7E-05 | 2.8E-03 | #N/A | #N/A |
| DN19557_c3_g3_i2 | 1.91 | 2.94 | 5.7E-05 | 2.8E-03 | #N/A | #N/A |
| DN15759_c1_g2_i10 | -2.00 | 3.60 | 5.7E-05 | 2.8E-03 | #N/A | #N/A |
| DN14876_c0_g3_i6 | 10.50 | 2.34 | 5.7E-05 | 2.8E-03 | LKHA4_ORYSJ | 0 |
| DN17773_c0_g3_i1 | -0.91 | 7.55 | 5.8E-05 | 2.8E-03 | DNJH2_ALLPO | 9.47E-123 |
| DN15621_c0_g1_i12 | 10.48 | 2.32 | 5.8E-05 | 2.8E-03 | #N/A | #N/A |
| DN19726_c1_g1_i2 | 7.87 | -0.17 | 6.0E-05 | 2.9E-03 | #N/A | #N/A |
| DN20173_c0_g4_i2 | 1.93 | 3.49 | 6.1E-05 | 2.9E-03 | TFB5_ARATH | 5.66E-33 |
| DN16274_c0_g6_i1 | -2.69 | 1.20 | 6.1E-05 | 2.9E-03 | #N/A | #N/A |
| DN21479_c0_g4_i20 | 9.13 | 1.00 | 6.1E-05 | 3.0E-03 | ZDHC2_ARATH | 4.05E-173 |
| DN16163_c0_g3_i7 | 7.65 | -0.38 | 6.1E-05 | 3.0E-03 | HESO1_ARATH | 1.39E-67 |
| DN18952_c0_g3_i1 | -1.06 | 4.49 | 6.1E-05 | 3.0E-03 | #N/A | #N/A |
| DN16141_c7_g12_i1 | -2.92 | 0.63 | 6.2E-05 | 3.0E-03 | #N/A | #N/A |
| DN15619_c0_g1_i11 | -1.57 | 3.06 | 6.2E-05 | 3.0E-03 | #N/A | #N/A |
| DN22204_c1_g1_i5 | 8.51 | 0.42 | 6.2E-05 | 3.0E-03 | DXR_ORYSJ | 8.99E-141 |
| DN21527_c1_g1_i5 | 7.88 | -0.17 | 6.2E-05 | 3.0E-03 | AFC1_ARATH | 1.3E-152 |
| DN15570_c2_g1_i1 | -0.80 | 7.10 | 6.2E-05 | 3.0E-03 | DNJ20_ARATH | 5.54E-30 |
| DN20649_c0_g1_i4 | 4.43 | 1.40 | 6.2E-05 | 3.0E-03 | #N/A | #N/A |
| DN19586_c3_g1_i9 | -2.20 | 1.75 | 6.3E-05 | 3.0E-03 | EML3_ARATH | 2.22E-42 |
| DN21969_c0_g1_i11 | 1.43 | 3.64 | 6.3E-05 | 3.0E-03 | #N/A | #N/A |
| DN22488_c1_g1_i10 | 1.24 | 5.80 | 6.3E-05 | 3.0E-03 | #N/A | #N/A |
| DN16309_c0_g1_i8 | 2.16 | 2.89 | 6.4E-05 | 3.1E-03 | #N/A | #N/A |
| DN16734_c0_g1_i4 | 8.70 | 0.60 | 6.5E-05 | 3.1E-03 | PP157_ARATH | 3.27E-20 |
| DN16173_c1_g1_i9 | 1.40 | 6.03 | 6.5E-05 | 3.1E-03 | CUT1B_ARATH | 9.59E-26 |
| DN15571_c4_g5_i1 | -2.66 | 1.99 | 6.5E-05 | 3.1E-03 | #N/A | #N/A |
| DN21331_c0_g1_i7 | 7.78 | -0.26 | 6.5E-05 | 3.1E-03 | AB7A_ARATH | 4.32E-49 |
| DN22464_c0_g1_i9 | 8.56 | 0.46 | 6.6E-05 | 3.1E-03 | ASHR1_ARATH | 2.73E-39 |
| DN21839_c0_g5_i1 | -2.06 | 1.35 | 6.6E-05 | 3.2E-03 | #N/A | #N/A |
| DN20397_c2_g3_i1 | -2.43 | 4.30 | 6.7E-05 | 3.2E-03 | CHUP1_ARATH | 1.29E-136 |
| DN18536_c0_g2_i5 | 2.88 | 1.40 | 6.7E-05 | 3.2E-03 | #N/A | #N/A |
| DN17887_c1_g2_i3 | -0.89 | 8.93 | 6.8E-05 | 3.2E-03 | CSLE6_ORYSJ | 0 |
| DN21244_c3_g9_i1 | 3.38 | 0.72 | 6.8E-05 | 3.2E-03 | **CHS2_MAIZE** | 1.39E-62 |
| DN18507_c1_g3_i9 | 2.07 | 1.98 | 6.8E-05 | 3.2E-03 | QKY_ARATH | 6.42E-24 |
| DN15690_c1_g2_i3 | 7.52 | -0.49 | 6.9E-05 | 3.3E-03 | #N/A | #N/A |
| DN18591_c0_g1_i15 | 2.45 | 2.72 | 6.9E-05 | 3.3E-03 | GONS3_ARATH | 1.04E-158 |
| DN20849_c1_g3_i6 | 2.99 | 3.75 | 7.0E-05 | 3.3E-03 | #N/A | #N/A |
| DN19500_c0_g12_i3 | -0.88 | 6.42 | 7.1E-05 | 3.3E-03 | PRSP1_SPIOL | 7.64E-82 |
| DN18593_c0_g1_i5 | 8.68 | 0.57 | 7.1E-05 | 3.4E-03 | MORC6_ARATH | 2.97E-158 |
| DN21928_c1_g5_i10 | 2.80 | 1.48 | 7.3E-05 | 3.4E-03 | IMDH_VIGUN | 0 |
| DN22153_c2_g1_i13 | 1.76 | 3.78 | 7.3E-05 | 3.4E-03 | Y1770_SYNY3 | 1.55E-86 |
| DN17864_c0_g1_i2 | 2.51 | 2.12 | 7.3E-05 | 3.4E-03 | GDL9_ARATH | 2.98E-56 |
| DN17383_c2_g3_i1 | 9.09 | 0.97 | 7.3E-05 | 3.5E-03 | #N/A | #N/A |
| DN15446_c0_g1_i4 | 2.65 | 2.15 | 7.4E-05 | 3.5E-03 | CHI1_ORYSJ | 8.77E-166 |
| DN17745_c0_g5_i2 | 8.38 | 0.29 | 7.4E-05 | 3.5E-03 | #N/A | #N/A |
| DN14697_c0_g4_i3 | 9.13 | 1.00 | 7.4E-05 | 3.5E-03 | #N/A | #N/A |
| DN22199_c0_g1_i32 | 9.43 | 1.29 | 7.4E-05 | 3.5E-03 | SIZ1_ORYSJ | 0 |
| DN21292_c0_g1_i10 | -2.79 | 2.45 | 7.5E-05 | 3.5E-03 | #N/A | #N/A |
| DN17494_c0_g2_i1 | -1.98 | 2.20 | 7.5E-05 | 3.5E-03 | ODP25_ARATH | 6.49E-87 |
| DN21554_c0_g1_i6 | 4.91 | 0.05 | 7.7E-05 | 3.6E-03 | #N/A | #N/A |
| DN15276_c2_g2_i10 | 7.96 | -0.10 | 7.7E-05 | 3.6E-03 | MPPA_SOLTU | 1.11E-101 |
| DN14927_c1_g2_i9 | 9.02 | 0.90 | 7.7E-05 | 3.6E-03 | #N/A | #N/A |
| DN19035_c1_g2_i18 | -6.19 | 0.41 | 7.8E-05 | 3.7E-03 | CRK19_ARATH | 1.59E-29 |
| DN19767_c0_g1_i5 | 1.87 | 4.70 | 7.8E-05 | 3.7E-03 | IPYR4_ARATH | 1.59E-100 |
| DN19223_c1_g3_i1 | 7.68 | -0.35 | 7.9E-05 | 3.7E-03 | PROF4_MAIZE | 1.71E-22 |
| DN19466_c0_g1_i6 | 7.78 | -0.26 | 7.9E-05 | 3.7E-03 | #N/A | #N/A |
| DN20821_c0_g2_i1 | -1.21 | 3.67 | 8.3E-05 | 3.9E-03 | PYRG2_XENLA | 3.32E-68 |
| DN15447_c1_g1_i6 | 9.41 | 1.28 | 8.3E-05 | 3.9E-03 | INVA_MAIZE | 0 |
| DN22678_c1_g1_i1 | 8.74 | 0.63 | 8.5E-05 | 4.0E-03 | CLASP_ARATH | 1.03E-31 |
| DN22552_c0_g1_i6 | 7.73 | -0.31 | 8.5E-05 | 4.0E-03 | #N/A | #N/A |
| DN20885_c0_g1_i16 | 5.50 | 3.49 | 8.6E-05 | 4.0E-03 | #N/A | #N/A |
| DN17989_c1_g1_i1 | 10.53 | 2.37 | 8.6E-05 | 4.0E-03 | SALR_PAPBR | 6.15E-72 |
| DN18403_c0_g1_i10 | 8.15 | 0.08 | 8.7E-05 | 4.0E-03 | ALG13_RAT | 1.67E-28 |
| DN22035_c0_g2_i3 | -1.72 | 3.73 | 8.7E-05 | 4.0E-03 | AAMT2_MAIZE | 0 |
| DN15917_c0_g2_i2 | -2.47 | 0.92 | 8.7E-05 | 4.1E-03 | #N/A | #N/A |
| DN18452_c0_g1_i11 | 9.99 | 1.84 | 9.0E-05 | 4.2E-03 | HAK22_ORYSJ | 0 |
| DN15979_c0_g1_i3 | 8.15 | 0.08 | 9.0E-05 | 4.2E-03 | #N/A | #N/A |
| DN16363_c0_g6_i2 | -1.48 | 4.02 | 9.0E-05 | 4.2E-03 | RVE2_ARATH | 1.04E-36 |
| DN20315_c0_g6_i1 | -0.88 | 5.06 | 9.0E-05 | 4.2E-03 | ACCO1_ORYSJ | 2.7E-97 |
| DN16099_c2_g2_i2 | 9.06 | 0.93 | 9.1E-05 | 4.2E-03 | GEML1_ARATH | 2.32E-73 |
| DN14794_c0_g1_i1 | -2.40 | 1.55 | 9.2E-05 | 4.2E-03 | FBK77_ARATH | 4.13E-149 |
| DN18585_c0_g1_i4 | 8.58 | 0.48 | 9.2E-05 | 4.2E-03 | #N/A | #N/A |
| DN18032_c0_g5_i1 | 11.00 | 2.83 | 9.2E-05 | 4.3E-03 | #N/A | #N/A |
| DN21649_c0_g1_i1 | -2.54 | 1.61 | 9.3E-05 | 4.3E-03 | KOR1_ORYSJ | 0 |
| DN16241_c4_g1_i1 | 5.57 | 2.01 | 9.4E-05 | 4.3E-03 | #N/A | #N/A |
| DN14605_c0_g1_i1 | 1.30 | 5.56 | 9.4E-05 | 4.4E-03 | QORH_ARATH | 1.71E-132 |
| DN18656_c1_g1_i5 | 8.55 | 0.45 | 9.5E-05 | 4.4E-03 | #N/A | #N/A |
| DN15199_c2_g1_i3 | 1.33 | 3.83 | 9.5E-05 | 4.4E-03 | LOGL9_ORYSJ | 1.53E-86 |
| DN18648_c0_g2_i13 | 7.57 | -0.45 | 9.7E-05 | 4.5E-03 | #N/A | #N/A |
| DN17709_c0_g2_i5 | 7.72 | -0.32 | 9.7E-05 | 4.5E-03 | DBNBT_TAXCA | 2.96E-43 |
| DN14782_c0_g1_i5 | 8.35 | 0.26 | 9.8E-05 | 4.5E-03 | IMPA4_ARATH | 9.32E-74 |
| DN16124_c0_g1_i12 | -0.93 | 5.21 | 9.8E-05 | 4.5E-03 | TRL12_ORYSJ | 1.92E-118 |
| DN22454_c0_g1_i21 | -1.59 | 6.23 | 9.9E-05 | 4.5E-03 | OHK4_ORYSJ | 0 |
| DN12897_c0_g1_i1 | 1.04 | 6.65 | 9.9E-05 | 4.5E-03 | CDSP_ORYSJ | 2.16E-133 |
| DN19635_c0_g2_i13 | -0.98 | 6.73 | 9.9E-05 | 4.5E-03 | KADC_MAIZE | 7.85E-159 |
| DN17486_c1_g5_i1 | -1.25 | 6.09 | 9.9E-05 | 4.5E-03 | SRG1_ARATH | 8.49E-69 |
| DN21597_c2_g1_i1 | 10.91 | 2.75 | 1.0E-04 | 4.6E-03 | CHLI_ORYSJ | 0 |
| DN22330_c2_g1_i4 | 3.68 | 0.19 | 1.0E-04 | 4.6E-03 | NDUS1_SOLTU | 9.38E-75 |
| DN15817_c0_g1_i12 | -1.26 | 4.48 | 1.0E-04 | 4.6E-03 | #N/A | #N/A |
| DN18335_c1_g1_i4 | 8.75 | 0.64 | 1.0E-04 | 4.7E-03 | PRP8_MOUSE | 6.53E-121 |
| DN15312_c1_g2_i1 | 3.03 | 1.03 | 1.0E-04 | 4.7E-03 | TLP_ORYSJ | 7.95E-82 |
| DN18890_c0_g1_i3 | 8.54 | 0.44 | 1.0E-04 | 4.7E-03 | #N/A | #N/A |
| DN14843_c0_g3_i5 | -1.83 | 2.57 | 1.0E-04 | 4.7E-03 | #N/A | #N/A |
| DN15341_c2_g3_i2 | 6.43 | 1.45 | 1.0E-04 | 4.8E-03 | NUG2_ORYSJ | 0 |
| DN22488_c1_g1_i2 | 1.56 | 3.24 | 1.0E-04 | 4.8E-03 | #N/A | #N/A |
| DN19070_c0_g2_i8 | -1.91 | 2.00 | 1.0E-04 | 4.8E-03 | #N/A | #N/A |
| DN19277_c1_g1_i3 | -1.08 | 4.17 | 1.1E-04 | 4.8E-03 | GT14B_ARATH | 2.76E-84 |
| DN17522_c0_g2_i5 | -1.23 | 5.50 | 1.1E-04 | 4.8E-03 | PSAG_HORVU | 1.3E-55 |
| DN16386_c0_g2_i9 | 7.70 | -0.33 | 1.1E-04 | 4.8E-03 | #N/A | #N/A |
| DN21510_c2_g2_i7 | 1.08 | 5.54 | 1.1E-04 | 4.8E-03 | APR1_ORYSJ | 0 |
| DN21329_c1_g1_i16 | 7.15 | 3.74 | 1.1E-04 | 4.8E-03 | EXEC2_ORYSJ | 0 |
| DN20485_c0_g2_i1 | -1.63 | 2.83 | 1.1E-04 | 4.9E-03 | #N/A | #N/A |
| DN18821_c0_g6_i2 | -1.65 | 3.24 | 1.1E-04 | 4.9E-03 | HFA2E_ORYSJ | 1.99E-42 |
| DN15090_c2_g2_i6 | 7.75 | -0.29 | 1.1E-04 | 4.9E-03 | ASHR2_ARATH | 7.8E-85 |
| DN19811_c0_g4_i1 | -1.15 | 5.71 | 1.1E-04 | 5.0E-03 | FAB1B_ARATH | 0 |
| DN15018_c0_g1_i7 | 7.70 | -0.33 | 1.1E-04 | 5.0E-03 | #N/A | #N/A |
| DN18824_c2_g1_i19 | 8.62 | 0.52 | 1.1E-04 | 5.0E-03 | MVD2_ARATH | 0 |
| DN19790_c1_g1_i2 | 7.49 | -0.52 | 1.1E-04 | 5.0E-03 | IPO5_HUMAN | 2.53E-140 |
| DN15973_c0_g2_i2 | 11.42 | 3.24 | 1.1E-04 | 5.1E-03 | #N/A | #N/A |
| DN19931_c2_g2_i14 | 5.28 | 3.48 | 1.1E-04 | 5.1E-03 | Y5390_ARATH | 2.04E-154 |
| DN15869_c0_g6_i5 | -2.42 | 0.53 | 1.1E-04 | 5.1E-03 | WOX1A_ORYSJ | 1.41E-39 |
| DN18924_c0_g1_i2 | 1.80 | 4.04 | 1.1E-04 | 5.1E-03 | C86B1_ARATH | 2.2E-133 |
| DN19062_c0_g3_i4 | 0.92 | 5.60 | 1.1E-04 | 5.1E-03 | #N/A | #N/A |
| DN22618_c1_g3_i1 | -1.08 | 5.50 | 1.1E-04 | 5.2E-03 | TPS7_ARATH | 0 |
| DN14994_c0_g3_i6 | -2.30 | 2.23 | 1.1E-04 | 5.2E-03 | CAR7_ARATH | 4.95E-44 |
| DN22100_c2_g1_i3 | 1.44 | 4.02 | 1.2E-04 | 5.2E-03 | AGO4A_ORYSJ | 0 |
| DN21363_c1_g3_i1 | 2.87 | 1.10 | 1.2E-04 | 5.2E-03 | #N/A | #N/A |
| DN20081_c1_g3_i4 | 5.40 | 0.49 | 1.2E-04 | 5.2E-03 | #N/A | #N/A |
| DN20179_c2_g4_i1 | -1.38 | 3.37 | 1.2E-04 | 5.2E-03 | BEBT_CLABR | 8.87E-42 |
| DN17911_c1_g1_i4 | -0.77 | 7.61 | 1.2E-04 | 5.2E-03 | PIP12_MAIZE | 0 |
| DN15358_c0_g1_i1 | 9.28 | 1.15 | 1.2E-04 | 5.3E-03 | DGP3_ARATH | 5.88E-77 |
| DN21890_c0_g1_i26 | 9.20 | 1.07 | 1.2E-04 | 5.3E-03 | RHL1_ARATH | 2.96E-57 |
| DN19192_c0_g1_i2 | 7.93 | -0.12 | 1.2E-04 | 5.3E-03 | CCA1_YEAST | 2.73E-37 |
| DN19635_c0_g2_i12 | 9.18 | 1.05 | 1.2E-04 | 5.3E-03 | KADC_MAIZE | 1.68E-31 |
| DN16755_c0_g1_i13 | -0.87 | 5.64 | 1.2E-04 | 5.3E-03 | CDF2_ARATH | 8.55E-49 |
| DN21954_c0_g3_i1 | -2.21 | 0.88 | 1.2E-04 | 5.3E-03 | EIN3_ARATH | 1.22E-158 |
| DN20350_c0_g1_i1 | 8.10 | 0.03 | 1.2E-04 | 5.3E-03 | #N/A | #N/A |
| DN20013_c0_g1_i4 | -2.70 | 0.31 | 1.2E-04 | 5.4E-03 | ODP25_ARATH | 2.63E-38 |
| DN18453_c0_g3_i1 | 8.02 | -0.05 | 1.2E-04 | 5.4E-03 | #N/A | #N/A |
| DN15244_c1_g2_i6 | -1.04 | 7.76 | 1.2E-04 | 5.5E-03 | EF1D1_ORYSJ | 3.51E-101 |
| DN14970_c0_g4_i1 | -1.54 | 2.76 | 1.2E-04 | 5.5E-03 | #N/A | #N/A |
| DN22795_c6_g4_i1 | -3.31 | -0.10 | 1.2E-04 | 5.5E-03 | PORA_ORYSJ | 1.1E-68 |
| DN17987_c1_g1_i8 | -1.73 | 1.82 | 1.2E-04 | 5.5E-03 | #N/A | #N/A |
| DN20066_c0_g3_i17 | 7.72 | -0.31 | 1.3E-04 | 5.5E-03 | AKRCA_ARATH | 4.92E-116 |
| DN16088_c0_g2_i5 | 2.31 | 2.43 | 1.3E-04 | 5.6E-03 | ATPBM_MAIZE | 0 |
| DN17950_c2_g2_i11 | 1.81 | 6.30 | 1.3E-04 | 5.6E-03 | PSAD_CUCSA | 4.35E-97 |
| DN16684_c0_g1_i10 | 7.84 | -0.21 | 1.3E-04 | 5.6E-03 | MBD4L_ARATH | 1.87E-39 |
| DN21649_c0_g1_i14 | -2.50 | 2.28 | 1.3E-04 | 5.6E-03 | KOR1_ORYSJ | 0 |
| DN20556_c0_g2_i8 | -1.81 | 2.36 | 1.3E-04 | 5.7E-03 | RER6_ARATH | 0 |
| DN16358_c4_g2_i2 | -2.97 | -0.37 | 1.3E-04 | 5.7E-03 | MLO5_ARATH | 0 |
| DN22801_c3_g1_i5 | 4.84 | 0.51 | 1.3E-04 | 5.7E-03 | #N/A | #N/A |
| DN21077_c1_g4_i2 | -1.57 | 3.92 | 1.3E-04 | 5.7E-03 | #N/A | #N/A |
| DN15196_c0_g1_i6 | 1.99 | 2.32 | 1.3E-04 | 5.7E-03 | #N/A | #N/A |
| DN15037_c0_g1_i7 | 9.13 | 1.00 | 1.3E-04 | 5.8E-03 | ELOC_RAT | 2.31E-27 |
| DN22312_c0_g1_i1 | 5.31 | -0.36 | 1.3E-04 | 5.8E-03 | #N/A | #N/A |
| DN18904_c0_g1_i16 | 7.99 | -0.07 | 1.3E-04 | 5.8E-03 | #N/A | #N/A |
| DN19915_c0_g4_i2 | 7.56 | -0.45 | 1.3E-04 | 5.9E-03 | YODA_ARATH | 1.75E-43 |
| DN19302_c0_g2_i14 | 3.57 | 1.56 | 1.3E-04 | 5.9E-03 | YQJG_ECOLI | 1.21E-97 |
| DN15407_c0_g3_i2 | -5.67 | 0.68 | 1.3E-04 | 5.9E-03 | DIT21_ARATH | 1.39E-160 |
| DN16097_c0_g2_i12 | 0.93 | 9.47 | 1.4E-04 | 5.9E-03 | SUI1_MAIZE | 7.78E-65 |
| DN19380_c0_g1_i5 | -1.33 | 7.80 | 1.4E-04 | 5.9E-03 | 12KD_FRAAN | 7.58E-28 |
| DN19916_c0_g3_i1 | 1.83 | 3.90 | 1.4E-04 | 5.9E-03 | PPCK2_ARATH | 5.94E-63 |
| DN16570_c3_g7_i3 | 7.73 | -0.31 | 1.4E-04 | 6.0E-03 | CBDAS_CANSA | 3.58E-58 |
| DN19550_c0_g3_i2 | 10.43 | 2.26 | 1.4E-04 | 6.1E-03 | PP230_ARATH | 1.13E-106 |
| DN17887_c1_g2_i2 | -1.04 | 4.70 | 1.4E-04 | 6.1E-03 | CSLE6_ORYSJ | 0 |
| DN22791_c0_g1_i8 | 8.50 | 0.40 | 1.4E-04 | 6.1E-03 | #N/A | #N/A |
| DN18622_c1_g2_i5 | 9.30 | 1.17 | 1.4E-04 | 6.1E-03 | #N/A | #N/A |
| DN17066_c1_g1_i3 | 11.48 | 3.30 | 1.4E-04 | 6.1E-03 | RS24_ARATH | 6.57E-128 |
| DN12365_c0_g1_i1 | -2.57 | 1.17 | 1.4E-04 | 6.1E-03 | Y1669_ARATH | 1.37E-137 |
| DN19497_c0_g2_i7 | -1.37 | 3.33 | 1.4E-04 | 6.1E-03 | #N/A | #N/A |
| DN16470_c2_g1_i7 | 7.47 | -0.54 | 1.4E-04 | 6.2E-03 | RNE_ARATH | 3.03E-96 |
| DN20001_c0_g1_i19 | 7.77 | -0.27 | 1.4E-04 | 6.2E-03 | CSPL8_SORBI | 8.66E-88 |
| DN21619_c0_g1_i3 | 9.52 | 1.38 | 1.4E-04 | 6.3E-03 | #N/A | #N/A |
| DN8490_c0_g1_i2 | -3.53 | 0.40 | 1.4E-04 | 6.3E-03 | KINB1_ARATH | 3.2E-87 |
| DN15536_c0_g1_i5 | 9.75 | 1.60 | 1.5E-04 | 6.3E-03 | #N/A | #N/A |
| DN16932_c0_g1_i5 | 1.55 | 3.06 | 1.5E-04 | 6.3E-03 | PALY_MAIZE | 5.78E-150 |
| DN14934_c0_g1_i7 | -3.13 | 2.95 | 1.5E-04 | 6.3E-03 | MACP1_ARATH | 4.9E-99 |
| DN22713_c1_g2_i5 | 1.37 | 6.27 | 1.5E-04 | 6.3E-03 | PAL2_ORYSI | 0 |
| DN15593_c1_g3_i1 | 1.98 | 2.83 | 1.5E-04 | 6.4E-03 | #N/A | #N/A |
| DN15535_c1_g7_i3 | 4.32 | 1.03 | 1.5E-04 | 6.4E-03 | **RHM1_ARATH** | 0 |
| DN14742_c1_g1_i4 | 4.88 | 2.27 | 1.5E-04 | 6.5E-03 | #N/A | #N/A |
| DN17139_c0_g1_i5 | 1.72 | 3.82 | 1.5E-04 | 6.6E-03 | RKP_ARATH | 0 |
| DN21005_c0_g1_i5 | 7.65 | -0.38 | 1.5E-04 | 6.6E-03 | BH082_ARATH | 4.43E-46 |
| DN18051_c0_g1_i2 | 2.53 | 3.79 | 1.5E-04 | 6.6E-03 | #N/A | #N/A |
| DN21298_c0_g1_i2 | 8.52 | 0.42 | 1.5E-04 | 6.6E-03 | ODP22_ARATH | 0 |
| DN16820_c1_g2_i1 | 8.90 | 0.79 | 1.5E-04 | 6.6E-03 | PER51_ARATH | 1.44E-97 |
| DN16285_c0_g1_i9 | 7.96 | -0.10 | 1.6E-04 | 6.7E-03 | AGD8_ARATH | 3.77E-23 |
| DN14698_c2_g1_i11 | 12.88 | 4.70 | 1.6E-04 | 6.7E-03 | TRNHF_ARATH | 7.3E-75 |
| DN17892_c2_g6_i1 | 9.05 | 0.93 | 1.6E-04 | 6.7E-03 | #N/A | #N/A |
| DN19325_c0_g2_i9 | -2.30 | 1.14 | 1.6E-04 | 6.7E-03 | #N/A | #N/A |
| DN21822_c0_g2_i9 | 9.03 | 0.90 | 1.6E-04 | 6.8E-03 | #N/A | #N/A |
| DN22347_c0_g3_i1 | 8.83 | 0.72 | 1.6E-04 | 6.8E-03 | Y1561_ARATH | 4.53E-132 |
| DN21682_c0_g2_i1 | 7.83 | -0.21 | 1.6E-04 | 6.8E-03 | AFC2_ARATH | 2.04E-46 |
| DN15624_c0_g1_i3 | 2.97 | 3.32 | 1.6E-04 | 6.8E-03 | CCR1_ARATH | 4.67E-124 |
| DN20908_c0_g1_i2 | 8.42 | 0.33 | 1.6E-04 | 6.8E-03 | PP155_ARATH | 2.87E-62 |
| DN20957_c0_g5_i1 | 9.12 | 0.99 | 1.6E-04 | 6.9E-03 | HSP72_SOLLC | 2.52E-110 |
| DN15382_c0_g2_i2 | -6.05 | -0.61 | 1.6E-04 | 6.9E-03 | #N/A | #N/A |
| DN18919_c0_g1_i8 | 1.96 | 3.58 | 1.6E-04 | 7.0E-03 | DCE1_ARATH | 0 |
| DN18341_c0_g3_i7 | 1.86 | 3.52 | 1.7E-04 | 7.1E-03 | C93A1_SOYBN | 1.24E-141 |
| DN22596_c1_g1_i5 | 2.27 | 2.40 | 1.7E-04 | 7.1E-03 | MSI1_ORYSJ | 6.97E-154 |
| DN22115_c4_g1_i14 | -1.66 | 2.28 | 1.7E-04 | 7.1E-03 | NFD4_ARATH | 1.16E-22 |
| DN21041_c0_g2_i1 | -0.97 | 6.75 | 1.7E-04 | 7.2E-03 | LHCA2_ARATH | 8.21E-63 |
| DN19260_c0_g4_i1 | 1.69 | 4.72 | 1.7E-04 | 7.2E-03 | COR2_PAPSO | 1.78E-113 |
| DN22069_c2_g1_i1 | 1.59 | 3.66 | 1.7E-04 | 7.3E-03 | WRK19_ARATH | 9.02E-58 |
| DN22626_c3_g1_i20 | -2.23 | 0.61 | 1.7E-04 | 7.3E-03 | PDK_ARATH | 6.35E-51 |
| DN15425_c0_g1_i1 | 7.91 | -0.15 | 1.7E-04 | 7.3E-03 | BEBT_TOBAC | 1.1E-54 |
| DN18583_c0_g1_i10 | 8.06 | 0.00 | 1.7E-04 | 7.4E-03 | #N/A | #N/A |
| DN16887_c1_g3_i1 | 4.31 | 0.69 | 1.8E-04 | 7.4E-03 | #N/A | #N/A |
| DN17783_c1_g1_i15 | -3.19 | 1.52 | 1.8E-04 | 7.4E-03 | ELP5_ARATH | 2.44E-61 |
| DN22188_c1_g2_i16 | -2.48 | 0.22 | 1.8E-04 | 7.4E-03 | IP5PF_ARATH | 1.63E-147 |
| DN15210_c0_g2_i5 | -1.00 | 4.96 | 1.8E-04 | 7.4E-03 | CDKAL_XENTR | 6.77E-180 |
| DN21598_c5_g1_i7 | 7.39 | -0.61 | 1.8E-04 | 7.4E-03 | #N/A | #N/A |
| DN21357_c0_g1_i3 | 0.92 | 5.43 | 1.8E-04 | 7.5E-03 | DNJ21_ARATH | 0 |
| DN19459_c0_g2_i6 | 1.70 | 2.63 | 1.8E-04 | 7.5E-03 | #N/A | #N/A |
| DN21510_c2_g2_i6 | -6.52 | 2.60 | 1.8E-04 | 7.5E-03 | APR1_ORYSJ | 0 |
| DN16783_c3_g2_i6 | -2.13 | 3.25 | 1.8E-04 | 7.5E-03 | #N/A | #N/A |
| DN15359_c0_g1_i8 | -1.86 | 2.61 | 1.8E-04 | 7.5E-03 | SSG1B_HORVU | 7.59E-146 |
| DN1158_c0_g1_i1 | -2.72 | 0.07 | 1.8E-04 | 7.5E-03 | #N/A | #N/A |
| DN20757_c0_g1_i4 | 8.71 | 0.60 | 1.8E-04 | 7.7E-03 | CRWN4_ARATH | 1.87E-130 |
| DN22673_c1_g2_i1 | 7.33 | -0.66 | 1.8E-04 | 7.7E-03 | #N/A | #N/A |
| DN18656_c1_g1_i4 | 8.52 | 0.42 | 1.8E-04 | 7.8E-03 | #N/A | #N/A |
| DN16408_c0_g1_i11 | 1.63 | 2.61 | 1.9E-04 | 7.8E-03 | EXLA3_ORYSJ | 2.89E-105 |
| DN16638_c1_g1_i11 | -1.14 | 4.73 | 1.9E-04 | 7.8E-03 | #N/A | #N/A |
| DN22526_c0_g2_i5 | 5.33 | 2.07 | 1.9E-04 | 7.8E-03 | #N/A | #N/A |
| DN16355_c0_g1_i27 | 2.02 | 4.01 | 1.9E-04 | 7.9E-03 | P2A13_ARATH | 1.26E-56 |
| DN20058_c0_g2_i1 | -1.31 | 3.10 | 1.9E-04 | 7.9E-03 | #N/A | #N/A |
| DN17144_c0_g2_i2 | 4.95 | 1.45 | 1.9E-04 | 7.9E-03 | #N/A | #N/A |
| DN17033_c2_g2_i3 | 5.79 | 2.04 | 1.9E-04 | 7.9E-03 | #N/A | #N/A |
| DN18413_c0_g1_i7 | 8.72 | 0.61 | 1.9E-04 | 7.9E-03 | Y3544_ARATH | 3.99E-40 |
| DN13084_c0_g1_i2 | -3.45 | -0.60 | 1.9E-04 | 8.0E-03 | #N/A | #N/A |
| DN16998_c0_g2_i6 | 8.04 | -0.02 | 1.9E-04 | 8.0E-03 | #N/A | #N/A |
| DN18416_c0_g2_i4 | 8.63 | 0.53 | 1.9E-04 | 8.0E-03 | #N/A | #N/A |
| DN22070_c0_g2_i7 | 2.84 | 1.54 | 1.9E-04 | 8.1E-03 | BCH_GENLU | 2.55E-90 |
| DN14769_c0_g2_i7 | -1.56 | 3.48 | 1.9E-04 | 8.1E-03 | #N/A | #N/A |
| DN15993_c2_g4_i6 | -1.89 | 1.63 | 1.9E-04 | 8.1E-03 | RB1BV_BETVU | 3.7E-55 |
| DN15172_c1_g5_i5 | 6.47 | 0.70 | 2.0E-04 | 8.1E-03 | #N/A | #N/A |
| DN16150_c0_g3_i9 | 8.15 | 3.90 | 2.0E-04 | 8.1E-03 | SDR2B_ARATH | 9.57E-61 |
| DN22602_c1_g1_i10 | 4.13 | 0.87 | 2.0E-04 | 8.2E-03 | #N/A | #N/A |
| DN18323_c1_g1_i4 | 8.68 | 0.58 | 2.0E-04 | 8.2E-03 | ASSY_ARATH | 1.68E-23 |
| DN21005_c0_g1_i16 | 8.73 | 0.62 | 2.0E-04 | 8.2E-03 | #N/A | #N/A |
| DN16532_c1_g8_i3 | 9.29 | 1.16 | 2.0E-04 | 8.2E-03 | #N/A | #N/A |
| DN20922_c1_g6_i3 | 2.43 | 2.43 | 2.0E-04 | 8.3E-03 | DPNPM_ARATH | 5.25E-39 |
| DN22732_c2_g2_i3 | 10.53 | 2.36 | 2.0E-04 | 8.3E-03 | UBC2_WHEAT | 2.41E-72 |
| DN20022_c1_g3_i7 | 8.60 | 0.50 | 2.0E-04 | 8.3E-03 | PRP18_BOVIN | 3.81E-36 |
| DN20368_c0_g1_i24 | -1.25 | 5.85 | 2.1E-04 | 8.5E-03 | #N/A | #N/A |
| DN22444_c1_g2_i15 | 7.80 | -0.25 | 2.1E-04 | 8.5E-03 | #N/A | #N/A |
| DN17525_c2_g1_i3 | -1.84 | 1.24 | 2.1E-04 | 8.6E-03 | ARP_ARATH | 1.66E-129 |
| DN16625_c0_g1_i6 | 3.18 | 0.47 | 2.1E-04 | 8.6E-03 | #N/A | #N/A |
| DN22539_c0_g2_i7 | 7.75 | -0.29 | 2.1E-04 | 8.6E-03 | #N/A | #N/A |
| DN15647_c1_g1_i3 | 8.53 | 0.43 | 2.1E-04 | 8.6E-03 | NAC92_ARATH | 1.03E-73 |
| DN16119_c1_g2_i10 | 1.35 | 5.33 | 2.1E-04 | 8.6E-03 | TCMO_POPKI | 3.98E-122 |
| DN22427_c1_g2_i2 | 1.17 | 4.34 | 2.1E-04 | 8.6E-03 | PNSB3_ARATH | 4.44E-36 |
| DN17212_c0_g3_i7 | -1.14 | 4.70 | 2.1E-04 | 8.7E-03 | DHBK_SOLLC | 2.95E-130 |
| DN15203_c1_g1_i4 | 2.64 | 1.34 | 2.1E-04 | 8.7E-03 | PTI5_SOLLC | 4.72E-27 |
| DN16119_c1_g2_i5 | 1.21 | 5.46 | 2.1E-04 | 8.8E-03 | TCMO_CATRO | 0 |
| DN20304_c0_g1_i14 | -3.11 | 2.21 | 2.2E-04 | 8.9E-03 | HMA6_ARATH | 6.32E-21 |
| DN16978_c0_g2_i11 | 7.46 | -0.55 | 2.2E-04 | 8.9E-03 | KCO3_ORYSJ | 0 |
| DN16894_c2_g1_i16 | 7.93 | -0.13 | 2.2E-04 | 8.9E-03 | #N/A | #N/A |
| DN21992_c0_g6_i1 | 1.50 | 3.20 | 2.2E-04 | 8.9E-03 | ALFC3_ARATH | 0 |
| DN15121_c0_g8_i1 | 4.91 | 3.25 | 2.2E-04 | 8.9E-03 | #N/A | #N/A |
| DN18470_c1_g1_i7 | 9.26 | 1.12 | 2.2E-04 | 9.0E-03 | MTP1_ORYSJ | 3.1E-79 |
| DN21554_c0_g4_i2 | 8.04 | -0.02 | 2.2E-04 | 9.1E-03 | DWRF8_MAIZE | 3.9E-61 |
| DN20397_c2_g3_i8 | -1.03 | 8.77 | 2.2E-04 | 9.1E-03 | CHUP1_ARATH | 9.02E-155 |
| DN19675_c1_g1_i8 | -1.95 | 0.97 | 2.2E-04 | 9.1E-03 | TMK1_ARATH | 0 |
| DN19092_c0_g2_i19 | 7.36 | -0.63 | 2.2E-04 | 9.2E-03 | #N/A | #N/A |
| DN20704_c2_g2_i4 | -1.98 | 2.04 | 2.2E-04 | 9.2E-03 | CML4_ORYSJ | 9.26E-97 |
| DN21097_c0_g2_i7 | 9.00 | 0.87 | 2.3E-04 | 9.2E-03 | #N/A | #N/A |
| DN16896_c0_g1_i5 | 8.07 | 0.01 | 2.3E-04 | 9.2E-03 | #N/A | #N/A |
| DN18679_c0_g3_i11 | 8.07 | 0.01 | 2.3E-04 | 9.2E-03 | Y1491_ARATH | 9.29E-78 |
| DN16975_c3_g3_i7 | -0.95 | 5.93 | 2.3E-04 | 9.3E-03 | CER1_ARATH | 0 |
| DN18542_c1_g1_i1 | 8.12 | 0.05 | 2.3E-04 | 9.3E-03 | #N/A | #N/A |
| DN22255_c0_g1_i3 | 1.98 | 1.86 | 2.3E-04 | 9.3E-03 | #N/A | #N/A |
| DN19452_c0_g3_i5 | -1.47 | 4.37 | 2.3E-04 | 9.3E-03 | #N/A | #N/A |
| DN14807_c0_g4_i1 | -1.02 | 6.32 | 2.3E-04 | 9.4E-03 | #N/A | #N/A |
| DN15580_c0_g1_i5 | -2.50 | 2.57 | 2.3E-04 | 9.4E-03 | #N/A | #N/A |
| DN19981_c0_g2_i19 | -1.37 | 3.72 | 2.3E-04 | 9.4E-03 | Y1500_ARATH | 1.94E-22 |
| DN22427_c1_g2_i6 | -6.41 | 0.81 | 2.3E-04 | 9.5E-03 | #N/A | #N/A |
| DN17252_c2_g3_i7 | 9.83 | 1.68 | 2.4E-04 | 9.6E-03 | PFD2_ARATH | 5.34E-60 |
| DN21605_c0_g3_i18 | 3.23 | 1.85 | 2.4E-04 | 9.6E-03 | **AB11G_ARATH** | 0 |
| DN18697_c1_g1_i3 | 2.50 | 3.74 | 2.4E-04 | 9.8E-03 | #N/A | #N/A |
| DN22257_c0_g1_i8 | 1.30 | 5.47 | 2.4E-04 | 9.9E-03 | RPB2_SOLLC | 0 |
| DN16268_c0_g9_i1 | 7.64 | -0.39 | 2.4E-04 | 9.9E-03 | #N/A | #N/A |
| DN18090_c0_g2_i6 | 3.09 | 3.64 | 2.5E-04 | 9.9E-03 | #N/A | #N/A |
| DN19568_c0_g1_i8 | -1.36 | 5.61 | 2.5E-04 | 9.9E-03 | #N/A | #N/A |
| DN19719_c0_g2_i12 | 0.98 | 4.74 | 2.5E-04 | 1.0E-02 | SYNO_ARATH | 0 |
| DN15473_c1_g2_i5 | 8.39 | 4.22 | 2.5E-04 | 1.0E-02 | Y3475_ARATH | 9.16E-151 |
| DN13589_c0_g1_i3 | -0.92 | 9.16 | 2.5E-04 | 1.0E-02 | PSAH_MAIZE | 9.31E-46 |
| DN17017_c1_g1_i5 | 7.81 | -0.23 | 2.5E-04 | 1.0E-02 | GRF6_ORYSJ | 1.04E-77 |
| DN16687_c0_g2_i10 | 0.81 | 7.46 | 2.5E-04 | 1.0E-02 | BGL31_ORYSJ | 0 |
| DN17267_c0_g4_i6 | 2.68 | 1.42 | 2.5E-04 | 1.0E-02 | #N/A | #N/A |
| DN15634_c1_g1_i11 | 10.05 | 1.89 | 2.5E-04 | 1.0E-02 | CDPK1_ARATH | 1.14E-94 |
| DN19486_c0_g2_i10 | 5.92 | 0.28 | 2.5E-04 | 1.0E-02 | DHQS_ACTCH | 1.74E-165 |
| DN16614_c0_g1_i6 | 7.62 | -0.40 | 2.6E-04 | 1.0E-02 | SWC2_ARATH | 3.88E-61 |
| DN18877_c1_g1_i18 | 10.26 | 2.11 | 2.6E-04 | 1.0E-02 | #N/A | #N/A |
| DN7706_c0_g1_i1 | 8.42 | 0.33 | 2.6E-04 | 1.0E-02 | #N/A | #N/A |
| DN16778_c0_g2_i6 | 7.43 | -0.57 | 2.6E-04 | 1.0E-02 | #N/A | #N/A |
| DN17868_c3_g1_i2 | -0.89 | 4.63 | 2.6E-04 | 1.0E-02 | NU4C_SACHY | 0 |
| DN21863_c1_g2_i3 | 0.92 | 6.12 | 2.6E-04 | 1.1E-02 | HPPD_HORVU | 4.18E-166 |
| DN20824_c1_g1_i25 | 8.44 | 0.35 | 2.7E-04 | 1.1E-02 | #N/A | #N/A |
| DN18543_c1_g1_i25 | -1.42 | 3.14 | 2.7E-04 | 1.1E-02 | CRK10_ARATH | 1.03E-67 |
| DN22439_c1_g7_i1 | -0.93 | 6.60 | 2.7E-04 | 1.1E-02 | PSBO_FRIAG | 3.57E-38 |
| DN21837_c0_g1_i2 | 8.09 | 0.03 | 2.7E-04 | 1.1E-02 | ZIFL1_ARATH | 4.74E-92 |
| DN20467_c1_g1_i7 | 8.27 | 0.19 | 2.7E-04 | 1.1E-02 | ERGI3_DANRE | 8.4E-73 |
| DN17773_c0_g1_i4 | 1.24 | 3.91 | 2.7E-04 | 1.1E-02 | DNJH_CUCSA | 2.43E-70 |
| DN17670_c1_g1_i9 | 9.52 | 1.38 | 2.8E-04 | 1.1E-02 | #N/A | #N/A |
| DN14925_c0_g3_i6 | -1.81 | 4.62 | 2.8E-04 | 1.1E-02 | #N/A | #N/A |
| DN20724_c1_g1_i3 | 1.97 | 4.26 | 2.8E-04 | 1.1E-02 | RFS2_ARATH | 0 |
| DN19135_c2_g2_i7 | 1.65 | 8.54 | 2.8E-04 | 1.1E-02 | FRI1_MAIZE | 3.32E-138 |
| DN18464_c1_g3_i2 | 8.13 | 0.06 | 2.8E-04 | 1.1E-02 | #N/A | #N/A |
| DN21759_c0_g1_i3 | 7.84 | -0.20 | 2.8E-04 | 1.1E-02 | #N/A | #N/A |
| DN21781_c2_g2_i6 | 3.81 | 0.41 | 2.8E-04 | 1.1E-02 | #N/A | #N/A |
| DN21901_c0_g1_i24 | 1.27 | 3.43 | 2.8E-04 | 1.1E-02 | #N/A | #N/A |
| DN16381_c1_g2_i4 | 8.50 | 0.40 | 2.8E-04 | 1.1E-02 | RHF2A_ARATH | 2.14E-58 |
| DN17258_c0_g3_i7 | -1.41 | 4.92 | 2.8E-04 | 1.1E-02 | GLR34_ARATH | 0 |
| DN21008_c0_g1_i1 | 2.05 | 3.96 | 2.9E-04 | 1.1E-02 | ZEP_ORYSJ | 0 |
| DN16932_c0_g1_i3 | 3.18 | 0.11 | 2.9E-04 | 1.1E-02 | PALY_MAIZE | 8.84E-147 |
| DN14037_c0_g1_i2 | 2.60 | 1.14 | 2.9E-04 | 1.1E-02 | #N/A | #N/A |
| DN16158_c0_g3_i4 | 9.90 | 1.75 | 2.9E-04 | 1.1E-02 | #N/A | #N/A |
| DN22063_c0_g1_i10 | 9.76 | 1.61 | 2.9E-04 | 1.1E-02 | AAP3_ARATH | 9.55E-158 |
| DN21326_c0_g1_i9 | 1.04 | 5.67 | 2.9E-04 | 1.1E-02 | HSP70_MAIZE | 1.71E-142 |
| DN14716_c0_g1_i1 | 11.87 | 3.69 | 2.9E-04 | 1.1E-02 | #N/A | #N/A |
| DN20655_c0_g1_i3 | 4.40 | 0.67 | 2.9E-04 | 1.2E-02 | VIP2_ARATH | 8.91E-87 |
| DN16961_c1_g5_i4 | 8.22 | 0.14 | 3.0E-04 | 1.2E-02 | #N/A | #N/A |
| DN20819_c0_g1_i8 | -1.22 | 3.56 | 3.0E-04 | 1.2E-02 | #N/A | #N/A |
| DN17059_c0_g1_i12 | 1.68 | 6.92 | 3.0E-04 | 1.2E-02 | F3PH_ARATH | 0 |
| DN16735_c0_g1_i1 | 1.12 | 5.88 | 3.0E-04 | 1.2E-02 | GSH1B_ORYSJ | 0 |
| DN16471_c0_g1_i4 | -1.45 | 5.74 | 3.0E-04 | 1.2E-02 | FBW2_ARATH | 1.82E-90 |
| DN19841_c1_g1_i13 | -1.99 | 1.88 | 3.0E-04 | 1.2E-02 | #N/A | #N/A |
| DN21122_c0_g1_i7 | 2.10 | 1.28 | 3.0E-04 | 1.2E-02 | WRK51_ARATH | 1.75E-36 |
| DN20126_c0_g1_i7 | 8.02 | -0.04 | 3.0E-04 | 1.2E-02 | COB21_ORYSJ | 3.94E-54 |
| DN21626_c3_g5_i4 | 9.49 | 1.35 | 3.1E-04 | 1.2E-02 | #N/A | #N/A |
| DN19576_c0_g5_i3 | 7.93 | -0.12 | 3.1E-04 | 1.2E-02 | #N/A | #N/A |
| DN17567_c1_g3_i3 | 9.16 | 1.03 | 3.1E-04 | 1.2E-02 | #N/A | #N/A |
| DN17545_c1_g3_i4 | 3.47 | 3.02 | 3.1E-04 | 1.2E-02 | OPT7_ARATH | 0 |
| DN20043_c0_g2_i9 | -1.31 | 6.77 | 3.1E-04 | 1.2E-02 | PSY_MAIZE | 2.97E-133 |
| DN15984_c1_g3_i5 | -1.12 | 4.61 | 3.1E-04 | 1.2E-02 | PHO13_ORYSJ | 0 |
| DN21806_c2_g3_i8 | -0.94 | 6.96 | 3.2E-04 | 1.2E-02 | RK28_ARATH | 6.79E-46 |
| DN18503_c0_g2_i3 | 3.26 | 2.76 | 3.2E-04 | 1.3E-02 | #N/A | #N/A |
| DN15691_c2_g1_i10 | 8.11 | 0.04 | 3.2E-04 | 1.3E-02 | #N/A | #N/A |
| DN20501_c0_g1_i23 | -1.05 | 3.70 | 3.2E-04 | 1.3E-02 | #N/A | #N/A |
| DN12015_c0_g1_i2 | 8.24 | 0.16 | 3.3E-04 | 1.3E-02 | NQR2_ORYSJ | 1.43E-65 |
| DN19054_c0_g3_i1 | -1.35 | 3.69 | 3.3E-04 | 1.3E-02 | DTX42_ARATH | 0 |
| DN21591_c0_g3_i2 | 1.62 | 2.29 | 3.3E-04 | 1.3E-02 | #N/A | #N/A |
| DN16081_c0_g3_i1 | -1.03 | 6.32 | 3.3E-04 | 1.3E-02 | #N/A | #N/A |
| DN16732_c0_g4_i3 | -3.03 | -0.10 | 3.3E-04 | 1.3E-02 | #N/A | #N/A |
| DN18540_c0_g1_i12 | -0.81 | 5.16 | 3.3E-04 | 1.3E-02 | #N/A | #N/A |
| DN20469_c0_g2_i7 | 2.77 | 5.44 | 3.3E-04 | 1.3E-02 | ASPRX_ORYSJ | 1.68E-89 |
| DN16400_c0_g1_i3 | -1.38 | 5.88 | 3.3E-04 | 1.3E-02 | H2AV3_ORYSJ | 2.86E-76 |
| DN16219_c2_g1_i7 | 2.31 | 1.25 | 3.3E-04 | 1.3E-02 | PALY_MAIZE | 2.47E-67 |
| DN22357_c1_g1_i9 | 7.14 | 1.46 | 3.3E-04 | 1.3E-02 | #N/A | #N/A |
| DN19339_c0_g1_i4 | 2.32 | 2.86 | 3.4E-04 | 1.3E-02 | FTSH8_ORYSJ | 0 |
| DN14810_c5_g3_i6 | -1.16 | 3.62 | 3.4E-04 | 1.3E-02 | #N/A | #N/A |
| DN18094_c2_g1_i3 | 9.03 | 0.91 | 3.4E-04 | 1.3E-02 | #N/A | #N/A |
| DN21041_c0_g3_i8 | -1.01 | 6.26 | 3.4E-04 | 1.3E-02 | CB12_SOLLC | 2.02E-35 |
| DN14944_c0_g1_i15 | 7.74 | -0.30 | 3.4E-04 | 1.3E-02 | DNJ49_ARATH | 7.1E-66 |
| DN22287_c1_g1_i1 | 0.97 | 5.12 | 3.4E-04 | 1.3E-02 | TLC1_SOLTU | 0 |
| DN21827_c0_g2_i17 | -1.13 | 3.78 | 3.4E-04 | 1.3E-02 | EDM2_ARATH | 8.13E-104 |
| DN20389_c0_g1_i9 | -3.36 | 0.86 | 3.4E-04 | 1.3E-02 | SYHM_ARATH | 4.68E-97 |
| DN12577_c0_g1_i4 | 2.54 | 1.83 | 3.4E-04 | 1.3E-02 | HS188_ORYSJ | 2.12E-29 |
| DN8359_c0_g1_i1 | -2.52 | 0.31 | 3.4E-04 | 1.3E-02 | #N/A | #N/A |
| DN16944_c0_g3_i7 | 5.65 | 1.84 | 3.4E-04 | 1.3E-02 | U80B1_ARATH | 0 |
| DN22335_c1_g1_i10 | 7.99 | -0.07 | 3.4E-04 | 1.3E-02 | KAT2_ORYSJ | 2.27E-80 |
| DN22564_c0_g1_i3 | 4.38 | 0.65 | 3.5E-04 | 1.3E-02 | AB48G_ORYSJ | 0 |
| DN18418_c0_g1_i9 | 3.10 | 6.75 | 3.5E-04 | 1.3E-02 | **CHS2_MAIZE** | 0 |
| DN19348_c0_g1_i6 | -3.08 | 0.27 | 3.5E-04 | 1.3E-02 | HRR25_YEAST | 6.77E-32 |
| DN19041_c1_g1_i23 | 3.39 | 1.15 | 3.5E-04 | 1.3E-02 | #N/A | #N/A |
| DN19035_c1_g2_i1 | 2.79 | 2.29 | 3.5E-04 | 1.3E-02 | CRK19_ARATH | 1.28E-36 |
| DN16907_c0_g1_i3 | 7.51 | -0.50 | 3.5E-04 | 1.3E-02 | #N/A | #N/A |
| DN17144_c0_g2_i6 | 4.72 | 0.34 | 3.5E-04 | 1.3E-02 | #N/A | #N/A |
| DN16097_c0_g2_i9 | 2.91 | 1.96 | 3.5E-04 | 1.3E-02 | SUI1_MAIZE | 4.97E-63 |
| DN21266_c0_g4_i2 | 7.90 | -0.15 | 3.5E-04 | 1.4E-02 | #N/A | #N/A |
| DN18837_c0_g2_i8 | 2.39 | 4.16 | 3.6E-04 | 1.4E-02 | U73C5_ARATH | 8.39E-131 |
| DN15165_c2_g2_i12 | 7.66 | -0.37 | 3.6E-04 | 1.4E-02 | #N/A | #N/A |
| DN16340_c3_g1_i3 | -2.29 | 2.16 | 3.6E-04 | 1.4E-02 | RS51_ARATH | 3.11E-124 |
| DN18838_c0_g1_i3 | -0.93 | 4.28 | 3.6E-04 | 1.4E-02 | TI10B_ORYSI | 7.88E-90 |
| DN18059_c0_g2_i3 | 8.58 | 0.48 | 3.6E-04 | 1.4E-02 | #N/A | #N/A |
| DN18607_c1_g2_i1 | -0.94 | 5.25 | 3.6E-04 | 1.4E-02 | BLH4_ARATH | 2.08E-123 |
| DN15873_c0_g1_i3 | 2.05 | 2.57 | 3.6E-04 | 1.4E-02 | #N/A | #N/A |
| DN14090_c0_g1_i2 | -1.12 | 3.67 | 3.7E-04 | 1.4E-02 | #N/A | #N/A |
| DN15720_c0_g4_i4 | 4.19 | 1.93 | 3.7E-04 | 1.4E-02 | ADF7_ORYSJ | 3.95E-58 |
| DN21729_c1_g1_i11 | 7.59 | -0.43 | 3.7E-04 | 1.4E-02 | UBP27_ARATH | 1.3E-39 |
| DN19876_c0_g3_i9 | 8.49 | 0.40 | 3.7E-04 | 1.4E-02 | #N/A | #N/A |
| DN21265_c0_g1_i7 | 5.50 | 1.06 | 3.7E-04 | 1.4E-02 | SWT13_ORYSJ | 1.73E-56 |
| DN15401_c0_g1_i1 | -1.45 | 6.01 | 3.7E-04 | 1.4E-02 | #N/A | #N/A |
| DN16546_c1_g3_i20 | 9.21 | 1.08 | 3.7E-04 | 1.4E-02 | FBT7_ARATH | 1.06E-91 |
| DN22784_c3_g4_i1 | -0.99 | 4.78 | 3.8E-04 | 1.4E-02 | #N/A | #N/A |
| DN16534_c1_g1_i1 | -1.45 | 8.17 | 3.8E-04 | 1.4E-02 | ZCD_CROSA | 2.92E-109 |
| DN16351_c0_g1_i3 | -0.80 | 6.97 | 3.8E-04 | 1.4E-02 | CAD8B_ORYSJ | 0 |
| DN16232_c1_g1_i2 | 1.23 | 4.00 | 3.8E-04 | 1.4E-02 | #N/A | #N/A |
| DN19347_c0_g1_i3 | -1.32 | 5.23 | 3.8E-04 | 1.4E-02 | ALA2_PANMI | 0 |
| DN18784_c2_g1_i12 | 8.75 | 0.64 | 3.8E-04 | 1.5E-02 | PP2A3_ORYSJ | 2.08E-129 |
| DN15315_c0_g7_i3 | -0.89 | 8.51 | 3.8E-04 | 1.5E-02 | #N/A | #N/A |
| DN16073_c1_g3_i3 | 8.37 | 0.28 | 3.9E-04 | 1.5E-02 | #N/A | #N/A |
| DN21417_c2_g2_i6 | -1.98 | 1.13 | 3.9E-04 | 1.5E-02 | #N/A | #N/A |
| DN14271_c0_g1_i1 | -1.55 | 4.28 | 3.9E-04 | 1.5E-02 | STR18_ARATH | 1.49E-24 |
| DN21786_c0_g1_i13 | 7.78 | -0.26 | 3.9E-04 | 1.5E-02 | #N/A | #N/A |
| DN17521_c0_g10_i2 | 0.89 | 6.84 | 3.9E-04 | 1.5E-02 | H1_MAIZE | 9.16E-20 |
| DN21062_c0_g1_i13 | -2.37 | 0.13 | 3.9E-04 | 1.5E-02 | MAPT_ARATH | 0 |
| DN20532_c0_g1_i9 | 5.85 | 0.12 | 3.9E-04 | 1.5E-02 | #N/A | #N/A |
| DN21111_c0_g5_i4 | 1.10 | 4.72 | 3.9E-04 | 1.5E-02 | #N/A | #N/A |
| DN14795_c0_g1_i2 | 1.92 | 4.82 | 3.9E-04 | 1.5E-02 | LECH_HORVU | 1.7E-26 |
| DN16748_c0_g1_i8 | 3.08 | 1.12 | 4.0E-04 | 1.5E-02 | #N/A | #N/A |
| DN22674_c1_g1_i1 | 8.11 | 0.04 | 4.0E-04 | 1.5E-02 | #N/A | #N/A |
| DN14807_c0_g6_i2 | 4.12 | -0.09 | 4.0E-04 | 1.5E-02 | LIAS_MAIZE | 9.44E-23 |
| DN15176_c0_g2_i3 | -1.06 | 5.83 | 4.0E-04 | 1.5E-02 | GLPT1_ARATH | 0 |
| DN19711_c0_g1_i4 | -1.03 | 3.61 | 4.0E-04 | 1.5E-02 | #N/A | #N/A |
| DN21357_c0_g1_i7 | -5.21 | 2.87 | 4.0E-04 | 1.5E-02 | DNJ21_ARATH | 0 |
| DN21726_c0_g1_i2 | 9.19 | 1.06 | 4.0E-04 | 1.5E-02 | CRTSO_DAUCA | 3.24E-44 |
| DN16109_c0_g2_i1 | 3.20 | 3.36 | 4.0E-04 | 1.5E-02 | DHAR2_ARATH | 1.01E-64 |
| DN16995_c0_g1_i2 | 2.68 | 0.42 | 4.0E-04 | 1.5E-02 | PSD8A_ARATH | 3.41E-102 |
| DN15274_c3_g1_i1 | 5.52 | -0.18 | 4.0E-04 | 1.5E-02 | #N/A | #N/A |
| DN17302_c1_g2_i1 | 7.44 | -0.57 | 4.0E-04 | 1.5E-02 | #N/A | #N/A |
| DN15973_c0_g1_i2 | 1.38 | 3.65 | 4.0E-04 | 1.5E-02 | #N/A | #N/A |
| DN20896_c0_g1_i16 | 7.38 | -0.62 | 4.0E-04 | 1.5E-02 | AUG5_ARATH | 0 |
| DN20296_c0_g1_i4 | 2.50 | 2.54 | 4.1E-04 | 1.5E-02 | PP399_ARATH | 0 |
| DN14612_c1_g4_i7 | -1.39 | 2.66 | 4.1E-04 | 1.5E-02 | G6PD_SOLTU | 3.65E-111 |
| DN20394_c0_g3_i1 | 8.53 | 0.43 | 4.1E-04 | 1.5E-02 | #N/A | #N/A |
| DN15470_c0_g3_i4 | 1.09 | 6.31 | 4.1E-04 | 1.5E-02 | OPR11_ORYSJ | 0 |
| DN22058_c1_g1_i1 | -0.91 | 8.98 | 4.1E-04 | 1.5E-02 | HSP7S_PEA | 0 |
| DN21463_c0_g3_i2 | 1.76 | 2.81 | 4.1E-04 | 1.5E-02 | PAL2_ORYSI | 8.1E-117 |
| DN19603_c0_g1_i9 | 9.60 | 1.45 | 4.1E-04 | 1.5E-02 | GPDL4_ARATH | 3.33E-129 |
| DN15296_c0_g3_i8 | 4.20 | -0.13 | 4.1E-04 | 1.5E-02 | #N/A | #N/A |
| DN17295_c0_g1_i3 | 1.15 | 7.28 | 4.2E-04 | 1.5E-02 | CLH2_ARATH | 2.91E-76 |
| DN22145_c3_g1_i11 | -0.95 | 4.99 | 4.2E-04 | 1.5E-02 | CIPKL_ORYSJ | 0 |
| DN18513_c1_g1_i10 | -2.43 | 0.04 | 4.2E-04 | 1.5E-02 | NFYB3_ARATH | 2.96E-61 |
| DN15690_c1_g2_i5 | 10.52 | 2.36 | 4.2E-04 | 1.5E-02 | #N/A | #N/A |
| DN18032_c0_g5_i2 | -0.71 | 6.52 | 4.2E-04 | 1.6E-02 | #N/A | #N/A |
| DN22667_c2_g2_i10 | 2.04 | 4.05 | 4.2E-04 | 1.6E-02 | #N/A | #N/A |
| DN20555_c0_g1_i18 | 3.64 | 0.41 | 4.2E-04 | 1.6E-02 | #N/A | #N/A |
| DN18609_c0_g4_i14 | 8.05 | -0.01 | 4.3E-04 | 1.6E-02 | #N/A | #N/A |
| DN19819_c0_g1_i17 | 3.37 | 0.52 | 4.3E-04 | 1.6E-02 | #N/A | #N/A |
| DN19110_c0_g2_i2 | 13.67 | 5.48 | 4.3E-04 | 1.6E-02 | TR164_ORYSJ | 1E-129 |
| DN20729_c0_g1_i1 | 1.40 | 4.46 | 4.3E-04 | 1.6E-02 | XLG3_ARATH | 0 |
| DN22042_c4_g2_i1 | 7.90 | -0.15 | 4.3E-04 | 1.6E-02 | #N/A | #N/A |
| DN21311_c0_g1_i9 | 8.23 | 0.16 | 4.3E-04 | 1.6E-02 | ARI8_ARATH | 3.28E-70 |
| DN20475_c0_g2_i14 | -0.96 | 7.43 | 4.4E-04 | 1.6E-02 | RRP3_HORVU | 6.45E-67 |
| DN17587_c0_g1_i2 | -6.05 | 0.47 | 4.4E-04 | 1.6E-02 | RB3GP_XENLA | 1.13E-40 |
| DN21072_c2_g1_i9 | -0.95 | 6.49 | 4.4E-04 | 1.6E-02 | DUS1_ARATH | 2.01E-32 |
| DN15446_c0_g1_i1 | 1.91 | 5.10 | 4.4E-04 | 1.6E-02 | CHI11_ORYSJ | 6.22E-133 |
| DN16130_c0_g1_i4 | -2.20 | 0.76 | 4.4E-04 | 1.6E-02 | #N/A | #N/A |
| DN19569_c2_g3_i4 | 7.70 | 3.17 | 4.4E-04 | 1.6E-02 | RL73_ARATH | 1.75E-41 |
| DN21756_c0_g1_i1 | 5.18 | 2.01 | 4.4E-04 | 1.6E-02 | EIF3E_ARATH | 6.29E-24 |
| DN14888_c0_g5_i7 | 8.70 | 0.59 | 4.5E-04 | 1.6E-02 | AMT33_ORYSJ | 0 |
| DN14546_c0_g1_i1 | 1.06 | 5.75 | 4.5E-04 | 1.6E-02 | EGY3_ORYSJ | 0 |
| DN22411_c0_g3_i4 | -1.39 | 3.18 | 4.5E-04 | 1.7E-02 | C3H28_ORYSJ | 1.97E-102 |
| DN17230_c0_g3_i6 | 7.55 | -0.47 | 4.5E-04 | 1.7E-02 | #N/A | #N/A |
| DN17856_c0_g3_i4 | 3.04 | 1.21 | 4.6E-04 | 1.7E-02 | RBP47_NICPL | 5.33E-88 |
| DN20925_c2_g2_i7 | -2.10 | 0.91 | 4.6E-04 | 1.7E-02 | #N/A | #N/A |
| DN17230_c0_g3_i5 | 7.51 | -0.50 | 4.6E-04 | 1.7E-02 | #N/A | #N/A |
| DN16633_c0_g1_i6 | -1.63 | 1.71 | 4.6E-04 | 1.7E-02 | #N/A | #N/A |
| DN20015_c0_g1_i5 | 8.07 | 0.01 | 4.6E-04 | 1.7E-02 | CGEP_ORYSJ | 0 |
| DN22524_c0_g1_i7 | 3.17 | -0.09 | 4.6E-04 | 1.7E-02 | HSP83_ORYSJ | 1.5E-121 |
| DN19035_c1_g2_i20 | 1.68 | 2.90 | 4.6E-04 | 1.7E-02 | Y4230_ARATH | 1.26E-22 |
| DN19929_c0_g1_i9 | -0.95 | 4.14 | 4.6E-04 | 1.7E-02 | FRS7_ARATH | 7.94E-27 |
| DN18332_c0_g3_i10 | 7.88 | -0.17 | 4.6E-04 | 1.7E-02 | #N/A | #N/A |
| DN22018_c0_g1_i9 | -1.12 | 3.46 | 4.7E-04 | 1.7E-02 | SPSA3_ORYSJ | 0 |
| DN18188_c0_g4_i5 | -1.13 | 6.44 | 4.7E-04 | 1.7E-02 | #N/A | #N/A |
| DN16213_c0_g1_i2 | -2.13 | 1.79 | 4.7E-04 | 1.7E-02 | ATG16_ARATH | 6.05E-177 |
| DN15838_c0_g1_i9 | 8.05 | -0.02 | 4.7E-04 | 1.7E-02 | ATG10_ARATH | 7.16E-28 |
| DN16567_c0_g6_i2 | 1.26 | 4.66 | 4.8E-04 | 1.7E-02 | #N/A | #N/A |
| DN21367_c0_g2_i5 | 4.39 | 1.59 | 4.8E-04 | 1.7E-02 | PT225_ARATH | 1.77E-38 |
| DN18130_c0_g1_i17 | -0.71 | 7.64 | 4.8E-04 | 1.8E-02 | ALLN_ALLCE | 6.05E-101 |
| DN19431_c1_g3_i9 | -5.83 | -0.19 | 4.8E-04 | 1.8E-02 | #N/A | #N/A |
| DN15681_c2_g2_i4 | 9.72 | 1.57 | 4.9E-04 | 1.8E-02 | GALDH_ARATH | 5.23E-170 |
| DN16320_c1_g1_i7 | 2.95 | 2.96 | 4.9E-04 | 1.8E-02 | RBG3_ARATH | 6.01E-26 |
| DN15008_c3_g4_i11 | -1.36 | 3.72 | 4.9E-04 | 1.8E-02 | FAD1_MOUSE | 1.89E-30 |
| DN21040_c0_g2_i8 | 7.85 | -0.19 | 4.9E-04 | 1.8E-02 | #N/A | #N/A |
| DN18090_c0_g2_i1 | 4.28 | 1.16 | 4.9E-04 | 1.8E-02 | #N/A | #N/A |
| DN15536_c0_g1_i9 | 9.58 | 1.43 | 4.9E-04 | 1.8E-02 | #N/A | #N/A |
| DN22454_c0_g1_i5 | 1.50 | 3.06 | 4.9E-04 | 1.8E-02 | OHK4_ORYSJ | 0 |
| DN14406_c0_g1_i2 | 8.19 | 0.12 | 5.0E-04 | 1.8E-02 | #N/A | #N/A |
| DN20043_c0_g2_i6 | 2.19 | 3.42 | 5.0E-04 | 1.8E-02 | PSY_MAIZE | 0 |
| DN16613_c0_g1_i1 | -6.25 | 0.86 | 5.0E-04 | 1.8E-02 | FH13_ORYSJ | 0 |
| DN16786_c1_g2_i10 | 0.76 | 8.48 | 5.0E-04 | 1.8E-02 | UVB31_ARATH | 1.36E-137 |
| DN2870_c0_g1_i1 | 5.56 | 0.80 | 5.0E-04 | 1.8E-02 | LAC25_ORYSJ | 1.77E-76 |
| DN21841_c0_g2_i16 | 9.39 | 1.25 | 5.0E-04 | 1.8E-02 | SIP1_ORYSJ | 6.32E-25 |
| DN21094_c1_g1_i3 | 10.04 | 1.89 | 5.0E-04 | 1.8E-02 | P2C35_ORYSJ | 5.71E-146 |
| DN16897_c1_g5_i11 | 8.65 | 0.55 | 5.1E-04 | 1.8E-02 | SDR1_ARATH | 1.06E-36 |
| DN19984_c0_g1_i4 | 0.90 | 7.95 | 5.1E-04 | 1.8E-02 | EF1A_ORYSJ | 0 |
| DN19185_c0_g3_i1 | -1.57 | 2.14 | 5.1E-04 | 1.8E-02 | #N/A | #N/A |
| DN15090_c2_g7_i1 | 1.25 | 5.29 | 5.1E-04 | 1.8E-02 | UGT1_GARJA | 4.96E-88 |
| DN20992_c0_g7_i1 | -1.70 | 3.90 | 5.1E-04 | 1.8E-02 | TF26_SCHPO | 6.11E-149 |
| DN21243_c0_g1_i7 | -1.21 | 3.61 | 5.2E-04 | 1.8E-02 | #N/A | #N/A |
| DN21439_c1_g3_i18 | 1.89 | 4.90 | 5.2E-04 | 1.8E-02 | CARM1_ORYSJ | 0 |
| DN20110_c0_g1_i15 | 7.93 | -0.12 | 5.2E-04 | 1.8E-02 | TTC4_DICDI | 1.97E-23 |
| DN17457_c0_g3_i1 | -1.02 | 6.31 | 5.2E-04 | 1.9E-02 | #N/A | #N/A |
| DN17677_c0_g2_i2 | 9.25 | 1.12 | 5.2E-04 | 1.9E-02 | SKU5_ARATH | 0 |
| DN21463_c0_g8_i1 | 2.04 | 1.98 | 5.2E-04 | 1.9E-02 | PAL1_ORYSJ | 6.53E-158 |
| DN19411_c1_g3_i4 | 2.80 | 0.86 | 5.2E-04 | 1.9E-02 | BGH3B_BACO1 | 8.89E-81 |
| DN19811_c0_g1_i5 | -1.85 | 2.56 | 5.3E-04 | 1.9E-02 | FAB1C_ARATH | 0 |
| DN14842_c0_g1_i4 | -0.83 | 6.42 | 5.3E-04 | 1.9E-02 | F16P1_ORYSJ | 0 |
| DN21163_c3_g4_i6 | -0.84 | 9.54 | 5.3E-04 | 1.9E-02 | PDRP1_MAIZE | 0 |
| DN15487_c1_g4_i3 | 3.68 | 0.44 | 5.3E-04 | 1.9E-02 | C3H18_ORYSJ | 6.03E-45 |
| DN16415_c0_g1_i1 | 3.62 | 0.59 | 5.3E-04 | 1.9E-02 | Y3544_ARATH | 9.72E-34 |
| DN18678_c2_g7_i4 | -0.78 | 7.00 | 5.4E-04 | 1.9E-02 | #N/A | #N/A |
| DN19811_c0_g1_i2 | -1.27 | 4.46 | 5.4E-04 | 1.9E-02 | FAB1C_ARATH | 0 |
| DN21529_c0_g1_i7 | 7.61 | -0.41 | 5.4E-04 | 1.9E-02 | UTP4_SCHPO | 1.61E-25 |
| DN22507_c2_g1_i3 | 1.97 | 2.89 | 5.4E-04 | 1.9E-02 | #N/A | #N/A |
| DN16368_c0_g4_i2 | 8.30 | 0.22 | 5.4E-04 | 1.9E-02 | YI31B_YEAST | 5.88E-42 |
| DN19637_c1_g3_i5 | 5.79 | 2.28 | 5.4E-04 | 1.9E-02 | P2C50_ORYSJ | 1.52E-83 |
| DN20013_c0_g1_i8 | -1.48 | 2.60 | 5.4E-04 | 1.9E-02 | ODP25_ARATH | 2.08E-113 |
| DN17015_c0_g3_i6 | 1.78 | 3.27 | 5.4E-04 | 1.9E-02 | G2OX8_ARATH | 1.24E-81 |
| DN14970_c0_g4_i5 | -1.40 | 2.16 | 5.5E-04 | 1.9E-02 | #N/A | #N/A |
| DN16804_c0_g1_i1 | -0.81 | 5.00 | 5.5E-04 | 1.9E-02 | RPT2_ARATH | 2.33E-48 |
| DN15804_c0_g4_i2 | -1.24 | 3.02 | 5.5E-04 | 1.9E-02 | #N/A | #N/A |
| DN16061_c0_g1_i2 | -2.05 | 1.31 | 5.5E-04 | 1.9E-02 | GEML4_ARATH | 1.86E-55 |
| DN17783_c1_g1_i14 | 7.57 | -0.45 | 5.5E-04 | 1.9E-02 | ELP5_ARATH | 1.24E-58 |
| DN21453_c0_g2_i2 | 4.53 | 2.71 | 5.5E-04 | 1.9E-02 | GLU2B_ORYSI | 3.98E-145 |
| DN18302_c0_g1_i6 | -2.03 | 2.62 | 5.5E-04 | 1.9E-02 | #N/A | #N/A |
| DN21963_c0_g1_i7 | 3.10 | 2.20 | 5.5E-04 | 1.9E-02 | #N/A | #N/A |
| DN15817_c0_g1_i10 | -1.28 | 4.17 | 5.5E-04 | 2.0E-02 | #N/A | #N/A |
| DN17039_c5_g9_i2 | 7.37 | -0.63 | 5.6E-04 | 2.0E-02 | #N/A | #N/A |
| DN18257_c0_g3_i11 | 2.33 | 2.21 | 5.6E-04 | 2.0E-02 | TYPA_BACSU | 1.52E-61 |
| DN17964_c1_g3_i13 | -1.17 | 7.19 | 5.6E-04 | 2.0E-02 | #N/A | #N/A |
| DN16699_c2_g1_i13 | 7.60 | 3.97 | 5.6E-04 | 2.0E-02 | DER_NOSS1 | 2.9E-155 |
| DN14942_c1_g1_i2 | 3.55 | 1.56 | 5.7E-04 | 2.0E-02 | #N/A | #N/A |
| DN19544_c1_g2_i6 | -0.89 | 7.07 | 5.7E-04 | 2.0E-02 | #N/A | #N/A |
| DN17530_c0_g2_i4 | 0.99 | 5.21 | 5.7E-04 | 2.0E-02 | RS33_ARATH | 5.03E-140 |
| DN21016_c0_g3_i1 | 7.72 | -0.32 | 5.8E-04 | 2.0E-02 | #N/A | #N/A |
| DN17714_c5_g2_i1 | -1.48 | 2.49 | 5.9E-04 | 2.1E-02 | #N/A | #N/A |
| DN21530_c1_g1_i20 | -1.96 | 0.71 | 5.9E-04 | 2.1E-02 | SQD2_ARATH | 1.33E-70 |
| DN18093_c0_g2_i7 | -5.72 | -0.49 | 5.9E-04 | 2.1E-02 | #N/A | #N/A |
| DN14718_c0_g1_i7 | -1.53 | 1.87 | 5.9E-04 | 2.1E-02 | NCS1_COPJA | 1.39E-97 |
| DN16802_c0_g3_i3 | 3.70 | 1.42 | 5.9E-04 | 2.1E-02 | PR1A3_ARATH | 1.43E-72 |
| DN21248_c1_g4_i1 | -3.65 | -0.04 | 6.0E-04 | 2.1E-02 | CAN1_ORYSJ | 2.93E-93 |
| DN20972_c1_g4_i1 | 1.13 | 6.61 | 6.0E-04 | 2.1E-02 | TI10C_ORYSJ | 6.51E-47 |
| DN18656_c1_g1_i3 | 6.47 | 1.47 | 6.0E-04 | 2.1E-02 | #N/A | #N/A |
| DN17752_c0_g1_i1 | 1.25 | 6.17 | 6.0E-04 | 2.1E-02 | LTD_ORYSJ | 1.3E-82 |
| DN20922_c1_g6_i14 | -1.04 | 3.73 | 6.0E-04 | 2.1E-02 | DPNPM_ARATH | 3.39E-62 |
| DN22200_c0_g1_i4 | 8.19 | 0.11 | 6.1E-04 | 2.1E-02 | OML2_ORYSJ | 0 |
| DN19617_c2_g5_i1 | 7.36 | -0.64 | 6.1E-04 | 2.1E-02 | GSTX2_MAIZE | 3.55E-146 |
| DN20350_c0_g1_i4 | 4.96 | 0.07 | 6.1E-04 | 2.1E-02 | #N/A | #N/A |
| DN19834_c0_g1_i5 | 2.11 | 4.25 | 6.1E-04 | 2.1E-02 | RS141_MAIZE | 5.01E-84 |
| DN22079_c0_g1_i1 | -1.41 | 2.62 | 6.1E-04 | 2.1E-02 | PIF3_ARATH | 8.06E-32 |
| DN17950_c2_g2_i9 | -0.69 | 9.05 | 6.2E-04 | 2.1E-02 | PSAD_CUCSA | 7.68E-97 |
| DN21647_c1_g1_i5 | 7.30 | -0.69 | 6.2E-04 | 2.1E-02 | #N/A | #N/A |
| DN19233_c0_g1_i6 | -1.56 | 2.81 | 6.2E-04 | 2.1E-02 | HFA2C_ORYSJ | 2.16E-114 |
| DN22667_c2_g2_i9 | 1.94 | 4.23 | 6.2E-04 | 2.2E-02 | #N/A | #N/A |
| DN21096_c0_g5_i1 | 3.94 | 2.46 | 6.3E-04 | 2.2E-02 | ADO1_ORYSJ | 0 |
| DN15841_c0_g2_i8 | -0.90 | 4.33 | 6.3E-04 | 2.2E-02 | WEB1_ARATH | 1.02E-151 |
| DN18543_c1_g1_i10 | -2.20 | 1.13 | 6.3E-04 | 2.2E-02 | GSHB_BRAJU | 1.45E-115 |
| DN20475_c0_g2_i11 | -1.90 | 0.39 | 6.3E-04 | 2.2E-02 | RRP3_HORVU | 4.78E-57 |
| DN17618_c0_g2_i1 | 4.26 | 0.55 | 6.3E-04 | 2.2E-02 | #N/A | #N/A |
| DN16487_c0_g2_i2 | 3.17 | 3.42 | 6.4E-04 | 2.2E-02 | CFI3_ARATH | 6.84E-41 |
| DN14875_c0_g1_i7 | 7.50 | -0.51 | 6.4E-04 | 2.2E-02 | #N/A | #N/A |
| DN21356_c1_g1_i7 | -1.77 | 4.41 | 6.4E-04 | 2.2E-02 | PHYA1_MAIZE | 0 |
| DN16447_c0_g1_i1 | 2.16 | 2.74 | 6.4E-04 | 2.2E-02 | PAL2_ORYSI | 9.43E-152 |
| DN16562_c0_g2_i1 | 9.46 | 1.32 | 6.4E-04 | 2.2E-02 | P2C34_ORYSJ | 2.78E-92 |
| DN21291_c0_g1_i15 | 8.45 | 0.35 | 6.4E-04 | 2.2E-02 | ULP1C_ARATH | 3.64E-69 |
| DN22427_c1_g2_i5 | 1.89 | 2.71 | 6.4E-04 | 2.2E-02 | PNSB3_ARATH | 2.3E-55 |
| DN22221_c0_g6_i3 | -1.50 | 3.90 | 6.5E-04 | 2.2E-02 | #N/A | #N/A |
| DN19619_c1_g1_i20 | -1.42 | 2.37 | 6.5E-04 | 2.2E-02 | #N/A | #N/A |
| DN22462_c1_g3_i3 | 7.96 | -0.09 | 6.5E-04 | 2.2E-02 | #N/A | #N/A |
| DN17306_c0_g2_i2 | -0.72 | 7.01 | 6.6E-04 | 2.3E-02 | BBD2_ORYSJ | 0 |
| DN20112_c0_g3_i4 | 9.70 | 1.55 | 6.6E-04 | 2.3E-02 | ADO3_ORYSJ | 0 |
| DN22624_c4_g1_i8 | -3.43 | 0.82 | 6.6E-04 | 2.3E-02 | #N/A | #N/A |
| DN18656_c1_g1_i2 | 7.86 | -0.18 | 6.6E-04 | 2.3E-02 | #N/A | #N/A |
| DN19918_c2_g5_i1 | -3.00 | -0.49 | 6.6E-04 | 2.3E-02 | LRK41_ARATH | 3.49E-118 |
| DN20821_c0_g1_i24 | -3.63 | 0.00 | 6.6E-04 | 2.3E-02 | PYRG_DICDI | 0 |
| DN19981_c0_g2_i15 | -1.00 | 5.78 | 6.7E-04 | 2.3E-02 | Y1500_ARATH | 7.83E-90 |
| DN17408_c0_g2_i1 | -0.84 | 4.92 | 6.7E-04 | 2.3E-02 | TM147_XENLA | 2.04E-27 |
| DN18970_c3_g1_i3 | 4.49 | 0.74 | 6.7E-04 | 2.3E-02 | NAC10_ORYSJ | 2.18E-42 |
| DN15885_c0_g2_i1 | 4.59 | 1.94 | 6.8E-04 | 2.3E-02 | #N/A | #N/A |
| DN16527_c0_g4_i2 | -0.95 | 4.97 | 6.8E-04 | 2.3E-02 | #N/A | #N/A |
| DN19680_c0_g6_i1 | 2.01 | 3.89 | 6.8E-04 | 2.3E-02 | UGT1_GARJA | 3.67E-81 |
| DN18966_c1_g2_i13 | -0.89 | 6.14 | 6.9E-04 | 2.4E-02 | PSAE_HORVU | 1E-52 |
| DN14849_c0_g1_i7 | 7.81 | -0.23 | 6.9E-04 | 2.4E-02 | #N/A | #N/A |
| DN14888_c0_g5_i4 | 8.51 | 0.42 | 6.9E-04 | 2.4E-02 | AMT33_ORYSJ | 0 |
| DN19726_c1_g1_i15 | -1.10 | 5.08 | 6.9E-04 | 2.4E-02 | #N/A | #N/A |
| DN20949_c1_g1_i17 | -1.53 | 4.21 | 6.9E-04 | 2.4E-02 | #N/A | #N/A |
| DN22280_c0_g3_i2 | -0.90 | 6.69 | 6.9E-04 | 2.4E-02 | DNJH_CUCSA | 4.25E-121 |
| DN15466_c0_g5_i4 | -2.55 | 3.89 | 7.0E-04 | 2.4E-02 | NFYC4_ARATH | 1.65E-57 |
| DN13924_c0_g2_i2 | 3.99 | 0.03 | 7.0E-04 | 2.4E-02 | #N/A | #N/A |
| DN17880_c1_g2_i3 | -1.98 | 1.08 | 7.0E-04 | 2.4E-02 | #N/A | #N/A |
| DN19554_c5_g5_i2 | 4.59 | 0.21 | 7.1E-04 | 2.4E-02 | #N/A | #N/A |
| DN13248_c0_g1_i1 | 1.26 | 3.31 | 7.1E-04 | 2.4E-02 | PP185_ARATH | 6.72E-129 |
| DN18849_c0_g2_i2 | 4.55 | 0.86 | 7.1E-04 | 2.4E-02 | #N/A | #N/A |
| DN22135_c0_g2_i5 | -5.36 | 1.56 | 7.1E-04 | 2.4E-02 | #N/A | #N/A |
| DN18441_c0_g1_i8 | 1.18 | 3.37 | 7.1E-04 | 2.4E-02 | KMS1_ARATH | 1.26E-155 |
| DN18831_c0_g1_i3 | -1.63 | 1.83 | 7.1E-04 | 2.4E-02 | CRD1_HORVU | 5.07E-122 |
| DN22843_c4_g2_i6 | -2.12 | 0.39 | 7.1E-04 | 2.4E-02 | PI3K1_SOYBN | 0 |
| DN19892_c0_g4_i2 | 7.51 | -0.50 | 7.1E-04 | 2.4E-02 | OE64C_ARATH | 3.47E-144 |
| DN13885_c0_g1_i2 | 4.90 | 0.71 | 7.1E-04 | 2.4E-02 | RAD4_ARATH | 1.16E-123 |
| DN19379_c0_g2_i1 | -1.36 | 2.38 | 7.2E-04 | 2.4E-02 | #N/A | #N/A |
| DN20916_c0_g1_i6 | -1.67 | 2.32 | 7.2E-04 | 2.4E-02 | SKI32_ARATH | 2.99E-136 |
| DN22257_c0_g1_i5 | 1.20 | 3.87 | 7.3E-04 | 2.5E-02 | RPB2_SOLLC | 0 |
| DN18313_c0_g1_i1 | -1.95 | 1.27 | 7.3E-04 | 2.5E-02 | UPL5_ARATH | 1.07E-160 |
| DN20400_c0_g1_i9 | -1.41 | 4.01 | 7.3E-04 | 2.5E-02 | LMLN_DROPS | 3.07E-49 |
| DN20339_c0_g3_i1 | -2.26 | 2.64 | 7.4E-04 | 2.5E-02 | #N/A | #N/A |
| DN19964_c3_g1_i10 | 7.96 | -0.09 | 7.4E-04 | 2.5E-02 | EDR1_ARATH | 2.29E-73 |
| DN14612_c1_g1_i13 | 7.91 | -0.14 | 7.4E-04 | 2.5E-02 | #N/A | #N/A |
| DN20745_c0_g1_i3 | 1.00 | 5.29 | 7.4E-04 | 2.5E-02 | #N/A | #N/A |
| DN20067_c1_g1_i18 | -1.95 | 1.10 | 7.5E-04 | 2.5E-02 | GIGAN_ORYSJ | 0 |
| DN17037_c0_g4_i1 | -1.18 | 5.77 | 7.5E-04 | 2.5E-02 | #N/A | #N/A |
| DN20539_c0_g1_i23 | 8.08 | 0.01 | 7.5E-04 | 2.5E-02 | TET2_ARATH | 5.46E-65 |
| DN15648_c0_g4_i4 | -0.96 | 6.42 | 7.6E-04 | 2.5E-02 | HIR1_ARATH | 1.93E-168 |
| DN16990_c0_g1_i3 | 9.52 | 1.38 | 7.6E-04 | 2.5E-02 | ORYA_ORYSJ | 1.2E-71 |
| DN19270_c1_g2_i3 | 1.07 | 4.24 | 7.6E-04 | 2.6E-02 | GSTUH_ARATH | 2.65E-56 |
| DN16457_c1_g1_i1 | -1.18 | 4.10 | 7.7E-04 | 2.6E-02 | YCF36_PORPU | 3.54E-23 |
| DN22665_c1_g1_i5 | -3.41 | 0.04 | 7.7E-04 | 2.6E-02 | **PMA2_SOLLC** | 3.04E-80 |
| DN18653_c0_g2_i4 | 9.34 | 1.21 | 7.7E-04 | 2.6E-02 | DCTP1_MOUSE | 1.93E-28 |
| DN16600_c0_g1_i1 | -1.40 | 2.83 | 7.8E-04 | 2.6E-02 | #N/A | #N/A |
| DN21891_c1_g1_i5 | 8.01 | -0.05 | 7.8E-04 | 2.6E-02 | #N/A | #N/A |
| DN20642_c0_g1_i9 | -1.61 | 4.14 | 7.8E-04 | 2.6E-02 | #N/A | #N/A |
| DN19964_c3_g1_i11 | 7.97 | -0.09 | 7.8E-04 | 2.6E-02 | EDR1_ARATH | 2.27E-73 |
| DN18927_c1_g3_i2 | -1.71 | 3.76 | 7.8E-04 | 2.6E-02 | #N/A | #N/A |
| DN18752_c1_g2_i10 | 1.51 | 4.46 | 7.8E-04 | 2.6E-02 | #N/A | #N/A |
| DN16636_c2_g1_i11 | -3.41 | 3.08 | 7.9E-04 | 2.6E-02 | #N/A | #N/A |
| DN16412_c0_g5_i1 | 8.09 | 0.03 | 7.9E-04 | 2.6E-02 | **CYPB_VICFA** | 9.13E-42 |
| DN18888_c0_g2_i23 | -1.00 | 4.57 | 7.9E-04 | 2.6E-02 | ARSB_DICDI | 1.16E-34 |
| DN17973_c0_g1_i2 | 1.01 | 6.82 | 7.9E-04 | 2.6E-02 | #N/A | #N/A |
| DN19377_c0_g5_i6 | 5.52 | -0.07 | 7.9E-04 | 2.6E-02 | TIF6B_ORYSJ | 4.54E-49 |
| DN19873_c0_g4_i3 | 7.68 | -0.35 | 7.9E-04 | 2.6E-02 | #N/A | #N/A |
| DN14712_c0_g6_i2 | 2.13 | 1.93 | 7.9E-04 | 2.6E-02 | #N/A | #N/A |
| DN18376_c1_g2_i6 | 8.45 | 0.36 | 8.0E-04 | 2.7E-02 | 5NG4_PINTA | 1.66E-41 |
| DN12409_c0_g2_i1 | 2.37 | 2.89 | 8.0E-04 | 2.7E-02 | **708A6_MAIZE** | 2.63E-144 |
| DN18683_c1_g2_i3 | -0.70 | 5.90 | 8.0E-04 | 2.7E-02 | COL5_ARATH | 6.97E-28 |
| DN20821_c0_g1_i5 | -2.11 | 1.51 | 8.0E-04 | 2.7E-02 | PYRG_DICDI | 5.59E-116 |
| DN18661_c0_g4_i7 | -2.41 | 0.31 | 8.1E-04 | 2.7E-02 | #N/A | #N/A |
| DN16538_c1_g1_i3 | 6.15 | 0.40 | 8.1E-04 | 2.7E-02 | #N/A | #N/A |
| DN15885_c0_g2_i5 | -1.15 | 3.45 | 8.1E-04 | 2.7E-02 | #N/A | #N/A |
| DN16959_c0_g3_i13 | 8.19 | 0.11 | 8.1E-04 | 2.7E-02 | LHT2_ARATH | 2.76E-116 |
| DN16954_c0_g4_i7 | 0.96 | 4.44 | 8.2E-04 | 2.7E-02 | RNZC_ARATH | 5.28E-152 |
| DN22713_c1_g3_i1 | 1.93 | 2.70 | 8.2E-04 | 2.7E-02 | PAL2_ORYSI | 1.84E-50 |
| DN16957_c0_g2_i1 | 6.59 | 5.11 | 8.2E-04 | 2.7E-02 | CSCLD_ARATH | 0 |
| DN20854_c2_g3_i1 | 1.01 | 6.04 | 8.2E-04 | 2.7E-02 | PTR3_ARATH | 0 |
| DN18452_c0_g3_i1 | 8.20 | 0.12 | 8.2E-04 | 2.7E-02 | #N/A | #N/A |
| DN16959_c0_g7_i1 | -0.84 | 6.19 | 8.2E-04 | 2.7E-02 | AB3C_ARATH | 0 |
| DN22756_c2_g1_i1 | 2.70 | 1.75 | 8.2E-04 | 2.7E-02 | #N/A | #N/A |
| DN20604_c0_g7_i3 | 7.66 | -0.37 | 8.2E-04 | 2.7E-02 | #N/A | #N/A |
| DN16220_c0_g3_i2 | 8.73 | 0.62 | 8.3E-04 | 2.7E-02 | #N/A | #N/A |
| DN19241_c1_g4_i7 | 3.78 | 0.11 | 8.3E-04 | 2.7E-02 | RAE1_ARATH | 0 |
| DN15226_c1_g3_i12 | 7.51 | -0.51 | 8.3E-04 | 2.7E-02 | RISA_SCHPO | 4.44E-49 |
| DN21680_c0_g1_i12 | 1.85 | 1.81 | 8.3E-04 | 2.7E-02 | NHX3_ARATH | 9.81E-103 |
| DN21261_c1_g1_i3 | 2.14 | 3.39 | 8.4E-04 | 2.8E-02 | NDHO_ARATH | 5.04E-47 |
| DN19524_c1_g1_i10 | -1.47 | 1.81 | 8.4E-04 | 2.8E-02 | #N/A | #N/A |
| DN18864_c2_g1_i4 | 6.99 | 2.08 | 8.5E-04 | 2.8E-02 | #N/A | #N/A |
| DN19070_c0_g2_i1 | -2.32 | 0.97 | 8.5E-04 | 2.8E-02 | #N/A | #N/A |
| DN16005_c0_g1_i1 | -1.59 | 2.62 | 8.5E-04 | 2.8E-02 | #N/A | #N/A |
| DN19554_c5_g2_i4 | 7.54 | -0.47 | 8.6E-04 | 2.8E-02 | #N/A | #N/A |
| DN19009_c0_g1_i12 | -0.94 | 5.88 | 8.6E-04 | 2.8E-02 | NUD26_ARATH | 1.35E-37 |
| DN18596_c1_g2_i4 | -0.94 | 5.47 | 8.6E-04 | 2.8E-02 | LOG_ORYSJ | 5.31E-130 |
| DN16616_c1_g1_i4 | 7.62 | -0.41 | 8.6E-04 | 2.8E-02 | #N/A | #N/A |
| DN20737_c0_g1_i17 | -1.21 | 5.36 | 8.7E-04 | 2.8E-02 | #N/A | #N/A |
| DN19466_c0_g1_i1 | 4.74 | -0.12 | 8.7E-04 | 2.8E-02 | #N/A | #N/A |
| DN19876_c0_g1_i3 | 7.87 | -0.17 | 8.7E-04 | 2.9E-02 | #N/A | #N/A |
| DN20953_c0_g1_i20 | 8.85 | 7.07 | 8.7E-04 | 2.9E-02 | ELI9_HORVU | 2.89E-32 |
| DN13901_c0_g1_i4 | 2.77 | 1.12 | 8.7E-04 | 2.9E-02 | GT4_ORYSJ | 7.24E-71 |
| DN15759_c1_g2_i25 | -2.62 | 1.31 | 8.8E-04 | 2.9E-02 | #N/A | #N/A |
| DN21823_c1_g4_i2 | 1.94 | 3.13 | 8.8E-04 | 2.9E-02 | #N/A | #N/A |
| DN17763_c0_g1_i2 | 8.60 | 0.50 | 8.8E-04 | 2.9E-02 | #N/A | #N/A |
| DN12189_c0_g1_i2 | -1.39 | 4.28 | 8.9E-04 | 2.9E-02 | #N/A | #N/A |
| DN18669_c0_g1_i4 | -1.04 | 4.25 | 8.9E-04 | 2.9E-02 | D14_ORYSJ | 4.57E-148 |
| DN20953_c0_g1_i9 | 8.12 | 8.64 | 8.9E-04 | 2.9E-02 | ELI9_HORVU | 2.31E-31 |
| DN18633_c1_g1_i7 | 2.58 | 3.67 | 8.9E-04 | 2.9E-02 | PXG1_AVESA | 6.02E-21 |
| DN17740_c0_g2_i4 | 9.55 | 1.41 | 8.9E-04 | 2.9E-02 | TRA1_MAIZE | 1E-71 |
| DN17166_c0_g1_i2 | 7.37 | -0.62 | 8.9E-04 | 2.9E-02 | ATL80_ARATH | 7.04E-21 |
| DN21118_c0_g3_i2 | -6.64 | 1.57 | 8.9E-04 | 2.9E-02 | P2C62_ORYSJ | 1.42E-117 |
| DN16882_c0_g4_i1 | 0.84 | 5.73 | 8.9E-04 | 2.9E-02 | #N/A | #N/A |
| DN16631_c0_g5_i11 | 5.40 | -0.19 | 9.0E-04 | 2.9E-02 | #N/A | #N/A |
| DN16099_c2_g1_i9 | -1.52 | 4.09 | 9.0E-04 | 2.9E-02 | #N/A | #N/A |
| DN16642_c0_g1_i8 | 8.24 | 0.16 | 9.0E-04 | 2.9E-02 | NB5R1_ARATH | 1.55E-53 |
| DN18694_c0_g1_i13 | -3.60 | 0.51 | 9.0E-04 | 2.9E-02 | CLCD_ARATH | 0 |
| DN21312_c1_g2_i1 | 10.69 | 2.53 | 9.0E-04 | 2.9E-02 | EBP_ORYSJ | 5.91E-39 |
| DN15570_c2_g1_i5 | 5.07 | -0.49 | 9.0E-04 | 2.9E-02 | DNJ20_ARATH | 7.63E-28 |
| DN18554_c0_g3_i2 | -1.06 | 5.49 | 9.1E-04 | 3.0E-02 | GCSH_ORYSJ | 4.41E-77 |
| DN17903_c0_g2_i5 | 5.81 | 3.79 | 9.1E-04 | 3.0E-02 | RLA3_MAIZE | 1.39E-32 |
| DN18728_c2_g1_i6 | -1.24 | 3.70 | 9.1E-04 | 3.0E-02 | ORR6_ORYSI | 7.42E-72 |
| DN18653_c0_g3_i1 | 0.82 | 7.12 | 9.2E-04 | 3.0E-02 | APX1_ORYSI | 1.07E-108 |
| DN17593_c0_g2_i1 | -0.87 | 4.49 | 9.2E-04 | 3.0E-02 | ROC5_ORYSJ | 0 |
| DN22320_c0_g1_i27 | 10.46 | 2.30 | 9.2E-04 | 3.0E-02 | #N/A | #N/A |
| DN17885_c0_g1_i3 | 1.75 | 2.66 | 9.2E-04 | 3.0E-02 | #N/A | #N/A |
| DN18527_c0_g1_i1 | 8.56 | 0.46 | 9.3E-04 | 3.0E-02 | #N/A | #N/A |
| DN15473_c1_g2_i6 | 9.37 | 1.23 | 9.3E-04 | 3.0E-02 | Y3475_ARATH | 9.41E-168 |
| DN22340_c1_g1_i12 | 2.17 | 2.44 | 9.4E-04 | 3.0E-02 | OML4_ORYSJ | 0 |
| DN14756_c0_g1_i2 | 7.52 | -0.49 | 9.4E-04 | 3.0E-02 | #N/A | #N/A |
| DN16975_c3_g3_i4 | -1.80 | 1.39 | 9.4E-04 | 3.0E-02 | CER1_ARATH | 1.51E-122 |
| DN21083_c2_g1_i3 | -1.21 | 3.70 | 9.5E-04 | 3.1E-02 | #N/A | #N/A |
| DN18569_c0_g1_i6 | 10.16 | 4.27 | 9.5E-04 | 3.1E-02 | RLA2A_MAIZE | 7.49E-38 |
| DN18714_c0_g1_i2 | 7.57 | -0.45 | 9.5E-04 | 3.1E-02 | #N/A | #N/A |
| DN16642_c0_g1_i20 | 7.95 | -0.10 | 9.6E-04 | 3.1E-02 | NB5R1_ARATH | 1.17E-53 |
| DN21118_c0_g3_i1 | 1.18 | 3.20 | 9.6E-04 | 3.1E-02 | P2C62_ORYSJ | 1.24E-157 |
| DN18420_c0_g5_i1 | 2.99 | 1.05 | 9.6E-04 | 3.1E-02 | BLH9_ARATH | 3.26E-69 |
| DN18341_c0_g1_i1 | 1.03 | 4.52 | 9.7E-04 | 3.1E-02 | **RHM1_ARATH** | 0 |
| DN17066_c1_g1_i7 | 10.41 | 2.25 | 9.7E-04 | 3.1E-02 | RS24_ARATH | 3.38E-139 |
| DN16595_c0_g4_i1 | 1.69 | 2.56 | 9.8E-04 | 3.1E-02 | DCE1_ARATH | 0 |
| DN18850_c1_g2_i2 | 2.43 | 0.26 | 9.8E-04 | 3.1E-02 | #N/A | #N/A |
| DN19622_c0_g4_i1 | 8.84 | 0.72 | 9.8E-04 | 3.1E-02 | SAPK8_ORYSJ | 0 |
| DN20724_c1_g5_i1 | 2.07 | 2.69 | 9.8E-04 | 3.1E-02 | RFS6_ARATH | 0 |
| DN22427_c1_g2_i7 | -0.86 | 7.40 | 9.8E-04 | 3.2E-02 | PNSB3_ARATH | 3.49E-60 |
| DN22572_c2_g4_i2 | 7.93 | -0.13 | 9.9E-04 | 3.2E-02 | #N/A | #N/A |
| DN20495_c0_g2_i19 | -1.42 | 2.26 | 9.9E-04 | 3.2E-02 | #N/A | #N/A |
| DN20845_c0_g4_i2 | 1.03 | 4.64 | 9.9E-04 | 3.2E-02 | #N/A | #N/A |
| DN19900_c0_g2_i2 | -0.75 | 6.94 | 1.0E-03 | 3.2E-02 | PSAF_HORVU | 1.56E-62 |
| DN20216_c0_g3_i9 | 7.74 | -0.30 | 1.0E-03 | 3.2E-02 | #N/A | #N/A |
| DN22466_c0_g1_i6 | -1.13 | 3.01 | 1.0E-03 | 3.2E-02 | XPO2_ARATH | 0 |
| DN19088_c0_g1_i1 | 8.87 | 0.76 | 1.0E-03 | 3.2E-02 | #N/A | #N/A |
| DN19997_c0_g2_i8 | -1.24 | 3.32 | 1.0E-03 | 3.2E-02 | PPR68_ARATH | 1.63E-174 |
| DN14451_c0_g1_i2 | -1.41 | 3.03 | 1.0E-03 | 3.2E-02 | #N/A | #N/A |
| DN18860_c1_g2_i3 | -1.95 | 1.53 | 1.0E-03 | 3.2E-02 | #N/A | #N/A |
| DN18617_c0_g3_i2 | 2.54 | 2.90 | 1.0E-03 | 3.2E-02 | QRT2_ARATH | 2.66E-28 |
| DN16496_c1_g1_i1 | 7.91 | -0.15 | 1.0E-03 | 3.2E-02 | #N/A | #N/A |
| DN22267_c0_g1_i2 | 4.76 | 0.72 | 1.0E-03 | 3.3E-02 | RH21_ORYSJ | 0 |
| DN15342_c0_g1_i2 | -0.86 | 5.18 | 1.0E-03 | 3.3E-02 | QCR7_SOLTU | 2.14E-49 |
| DN17918_c1_g2_i8 | 8.03 | -0.02 | 1.0E-03 | 3.3E-02 | #N/A | #N/A |
| DN15313_c2_g1_i6 | -1.03 | 6.33 | 1.0E-03 | 3.3E-02 | ISCAP_ARATH | 1.93E-57 |
| DN30854_c0_g1_i1 | 8.18 | 0.11 | 1.0E-03 | 3.3E-02 | #N/A | #N/A |
| DN17922_c0_g2_i5 | -1.27 | 3.40 | 1.0E-03 | 3.3E-02 | #N/A | #N/A |
| DN13899_c0_g1_i1 | 1.37 | 3.20 | 1.0E-03 | 3.3E-02 | #N/A | #N/A |
| DN20655_c0_g4_i1 | -1.93 | 0.65 | 1.0E-03 | 3.3E-02 | #N/A | #N/A |
| DN19247_c0_g1_i3 | -2.90 | 1.23 | 1.0E-03 | 3.3E-02 | #N/A | #N/A |
| DN18200_c0_g1_i2 | -2.69 | 5.51 | 1.0E-03 | 3.3E-02 | CB2D_SOLLC | 1.66E-35 |
| DN20953_c0_g1_i13 | 1.33 | 9.92 | 1.0E-03 | 3.3E-02 | #N/A | #N/A |
| DN15914_c0_g1_i11 | -1.52 | 2.04 | 1.0E-03 | 3.3E-02 | CCS1_ORYSJ | 5.15E-133 |
| DN22012_c1_g2_i12 | -1.35 | 4.30 | 1.0E-03 | 3.3E-02 | #N/A | #N/A |
| DN17486_c1_g5_i9 | 1.96 | 3.11 | 1.1E-03 | 3.3E-02 | SRG1_ARATH | 7.58E-51 |
| DN18128_c1_g1_i7 | 8.22 | 0.14 | 1.1E-03 | 3.3E-02 | #N/A | #N/A |
| DN9748_c0_g1_i1 | 2.62 | 1.00 | 1.1E-03 | 3.3E-02 | #N/A | #N/A |
| DN15952_c0_g2_i11 | 2.62 | 1.21 | 1.1E-03 | 3.4E-02 | HSP23_MAIZE | 7.76E-69 |
| DN16093_c1_g1_i5 | -1.08 | 3.71 | 1.1E-03 | 3.4E-02 | PYRG_DICDI | 0 |
| DN22280_c1_g2_i10 | 0.80 | 6.78 | 1.1E-03 | 3.4E-02 | DNJH2_ALLPO | 1.69E-165 |
| DN16388_c1_g4_i12 | 2.29 | 1.39 | 1.1E-03 | 3.4E-02 | #N/A | #N/A |
| DN21039_c1_g1_i2 | -1.97 | 1.57 | 1.1E-03 | 3.4E-02 | #N/A | #N/A |
| DN19559_c0_g1_i1 | 7.55 | -0.46 | 1.1E-03 | 3.4E-02 | SKI15_ARATH | 2.71E-99 |
| DN17402_c0_g2_i8 | 7.92 | -0.13 | 1.1E-03 | 3.4E-02 | CML48_ARATH | 7.96E-28 |
| DN22698_c1_g1_i12 | 2.96 | 1.13 | 1.1E-03 | 3.4E-02 | FTSZ1_ARATH | 0 |
| DN16892_c0_g3_i8 | 4.58 | 0.55 | 1.1E-03 | 3.4E-02 | WAK5_ARATH | 5.52E-58 |
| DN22536_c0_g2_i9 | 7.64 | -0.39 | 1.1E-03 | 3.4E-02 | P2C43_ORYSJ | 3.37E-22 |
| DN20177_c1_g3_i14 | 6.57 | 5.27 | 1.1E-03 | 3.4E-02 | AMPL2_ORYSJ | 0 |
| DN20006_c0_g2_i7 | -3.50 | -0.51 | 1.1E-03 | 3.4E-02 | #N/A | #N/A |
| DN21411_c1_g3_i1 | 7.40 | -0.60 | 1.1E-03 | 3.5E-02 | #N/A | #N/A |
| DN17473_c0_g1_i4 | 7.25 | -0.73 | 1.1E-03 | 3.5E-02 | #N/A | #N/A |
| DN15825_c1_g1_i2 | -1.36 | 4.57 | 1.1E-03 | 3.5E-02 | FRO7_ARATH | 0 |
| DN21605_c0_g1_i4 | -1.38 | 3.18 | 1.1E-03 | 3.5E-02 | **AB11G_ARATH** | 3.39E-93 |
| DN16327_c1_g4_i5 | 8.99 | 0.87 | 1.1E-03 | 3.5E-02 | LOR8_ARATH | 1.52E-41 |
| DN13033_c0_g1_i1 | 1.68 | 3.67 | 1.1E-03 | 3.5E-02 | SAP17_ORYSJ | 3.56E-62 |
| DN17243_c1_g2_i2 | -2.25 | 2.00 | 1.1E-03 | 3.5E-02 | TOR_ORYSJ | 0 |
| DN21041_c0_g2_i5 | -0.85 | 6.54 | 1.1E-03 | 3.5E-02 | CB12_PETHY | 4.99E-46 |
| DN17716_c0_g2_i13 | -5.77 | 0.22 | 1.1E-03 | 3.6E-02 | EMB8_PICGL | 1.41E-42 |
| DN22566_c0_g1_i5 | -2.07 | 2.25 | 1.1E-03 | 3.6E-02 | #N/A | #N/A |
| DN17469_c5_g1_i5 | 4.04 | 1.57 | 1.1E-03 | 3.6E-02 | #N/A | #N/A |
| DN21602_c0_g1_i11 | 1.02 | 4.73 | 1.1E-03 | 3.6E-02 | GATP1_ORYSI | 0 |
| DN15296_c0_g2_i5 | 8.43 | 0.34 | 1.1E-03 | 3.6E-02 | TRXH_ORYSI | 2.05E-50 |
| DN18557_c0_g1_i1 | 4.16 | 1.04 | 1.1E-03 | 3.6E-02 | ACR6_ARATH | 1E-82 |
| DN21394_c1_g3_i3 | 5.77 | 2.87 | 1.1E-03 | 3.6E-02 | SPXM2_ORYSJ | 0 |
| DN18308_c0_g2_i1 | 7.48 | -0.53 | 1.1E-03 | 3.6E-02 | #N/A | #N/A |
| DN21709_c0_g5_i7 | 1.22 | 4.35 | 1.2E-03 | 3.6E-02 | U2AFB_ORYSJ | 1.52E-79 |
| DN18358_c0_g3_i13 | 7.83 | -0.22 | 1.2E-03 | 3.6E-02 | REV3_ARATH | 1.28E-174 |
| DN16534_c0_g1_i3 | -1.80 | 2.92 | 1.2E-03 | 3.6E-02 | #N/A | #N/A |
| DN17290_c0_g5_i1 | 1.29 | 4.10 | 1.2E-03 | 3.6E-02 | Y1154_ARATH | 0 |
| DN16629_c1_g1_i11 | -0.82 | 4.90 | 1.2E-03 | 3.6E-02 | PFPA_RICCO | 0 |
| DN17307_c1_g1_i4 | -0.82 | 4.85 | 1.2E-03 | 3.6E-02 | #N/A | #N/A |
| DN15367_c0_g1_i9 | -5.69 | -0.32 | 1.2E-03 | 3.6E-02 | #N/A | #N/A |
| DN16324_c0_g1_i20 | 8.16 | 0.09 | 1.2E-03 | 3.6E-02 | DBR1_ORYSJ | 6.35E-121 |
| DN19270_c1_g1_i2 | -2.53 | -0.18 | 1.2E-03 | 3.6E-02 | BH074_ARATH | 9.73E-49 |
| DN18527_c0_g1_i15 | 6.02 | 0.28 | 1.2E-03 | 3.6E-02 | #N/A | #N/A |
| DN16090_c0_g3_i5 | -1.14 | 4.54 | 1.2E-03 | 3.6E-02 | ALDO2_MAIZE | 0 |
| DN18126_c2_g3_i4 | -1.13 | 6.95 | 1.2E-03 | 3.6E-02 | AAED1_ARATH | 1.02E-88 |
| DN21041_c0_g1_i2 | -1.17 | 3.53 | 1.2E-03 | 3.6E-02 | #N/A | #N/A |
| DN15848_c1_g1_i3 | 10.18 | 2.02 | 1.2E-03 | 3.7E-02 | NAC83_ARATH | 1.01E-46 |
| DN16184_c1_g1_i2 | 7.66 | -0.36 | 1.2E-03 | 3.7E-02 | #N/A | #N/A |
| DN17402_c0_g3_i2 | 1.54 | 3.64 | 1.2E-03 | 3.7E-02 | AEE7_ARATH | 0 |
| DN17393_c1_g1_i9 | 2.27 | 1.93 | 1.2E-03 | 3.7E-02 | LPA1_ORYSJ | 0 |
| DN15773_c2_g1_i4 | -3.13 | 3.43 | 1.2E-03 | 3.7E-02 | CB22_MAIZE | 1.57E-93 |
| DN15982_c0_g4_i5 | 7.63 | -0.40 | 1.2E-03 | 3.7E-02 | G2OX1_ARATH | 2.19E-61 |
| DN20517_c0_g2_i10 | 7.86 | 2.82 | 1.2E-03 | 3.7E-02 | #N/A | #N/A |
| DN19479_c1_g1_i4 | -1.17 | 5.17 | 1.2E-03 | 3.7E-02 | C7D55_HYOMU | 4.77E-148 |
| DN17019_c1_g1_i10 | -1.05 | 8.42 | 1.2E-03 | 3.7E-02 | CHLP_ORYSJ | 0 |
| DN20022_c1_g3_i4 | 2.41 | 2.07 | 1.2E-03 | 3.7E-02 | PRP18_DANRE | 6.12E-54 |
| DN19588_c0_g1_i6 | 7.61 | 7.04 | 1.2E-03 | 3.7E-02 | FER2_MAIZE | 4.75E-74 |
| DN18635_c0_g1_i3 | 7.51 | -0.50 | 1.2E-03 | 3.7E-02 | RPB5A_ARATH | 3.27E-39 |
| DN14303_c0_g1_i7 | -1.44 | 4.51 | 1.2E-03 | 3.7E-02 | AT18F_ARATH | 8.89E-61 |
| DN20934_c0_g3_i4 | -1.62 | 1.56 | 1.2E-03 | 3.7E-02 | #N/A | #N/A |
| DN20911_c0_g1_i14 | 1.56 | 1.92 | 1.2E-03 | 3.7E-02 | #N/A | #N/A |
| DN17462_c0_g1_i17 | -1.10 | 3.64 | 1.2E-03 | 3.8E-02 | #N/A | #N/A |
| DN21439_c1_g3_i17 | 0.92 | 4.78 | 1.2E-03 | 3.8E-02 | CARM1_ORYSJ | 0 |
| DN15198_c0_g1_i1 | 1.82 | 3.04 | 1.2E-03 | 3.8E-02 | #N/A | #N/A |
| DN21917_c0_g1_i15 | 1.83 | 2.31 | 1.2E-03 | 3.8E-02 | ERCC8_BOVIN | 2.9E-47 |
| DN20244_c2_g1_i1 | -0.84 | 4.89 | 1.2E-03 | 3.8E-02 | #N/A | #N/A |
| DN13489_c0_g1_i4 | 3.68 | 0.44 | 1.3E-03 | 3.8E-02 | DAPB3_PSEMX | 1.42E-20 |
| DN20040_c0_g1_i15 | 3.67 | 1.17 | 1.3E-03 | 3.9E-02 | RS242_ARATH | 7.8E-71 |
| DN21827_c0_g1_i4 | -0.91 | 3.92 | 1.3E-03 | 3.9E-02 | #N/A | #N/A |
| DN21356_c1_g1_i5 | -0.80 | 5.28 | 1.3E-03 | 3.9E-02 | PHYA1_MAIZE | 0 |
| DN19674_c0_g4_i8 | 7.53 | -0.49 | 1.3E-03 | 3.9E-02 | ILR3_ARATH | 2.85E-25 |
| DN15555_c1_g3_i3 | 0.79 | 7.30 | 1.3E-03 | 3.9E-02 | MSR21_ORYSJ | 7.91E-109 |
| DN18257_c0_g3_i3 | 4.86 | 2.86 | 1.3E-03 | 3.9E-02 | TYPA_BACSU | 5.43E-153 |
| DN16390_c0_g6_i1 | -1.09 | 3.51 | 1.3E-03 | 3.9E-02 | #N/A | #N/A |
| DN16904_c0_g1_i1 | 7.43 | -0.57 | 1.3E-03 | 3.9E-02 | RL30_MAIZE | 1.01E-76 |
| DN17279_c0_g4_i1 | 2.48 | 0.28 | 1.3E-03 | 3.9E-02 | #N/A | #N/A |
| DN15917_c0_g1_i10 | 0.85 | 8.18 | 1.3E-03 | 3.9E-02 | TAS_SHIFL | 3.91E-95 |
| DN20039_c1_g2_i13 | 4.61 | -0.09 | 1.3E-03 | 3.9E-02 | RNP1_ARATH | 9.74E-38 |
| DN13829_c0_g1_i3 | 7.48 | -0.53 | 1.3E-03 | 3.9E-02 | SDI2_ARATH | 9.17E-122 |
| DN20495_c0_g1_i8 | 7.83 | -0.21 | 1.3E-03 | 3.9E-02 | SCP17_ARATH | 1.83E-84 |
| DN14750_c1_g1_i5 | -0.76 | 5.63 | 1.3E-03 | 3.9E-02 | Y1181_ARATH | 3.27E-116 |
| DN20592_c0_g4_i4 | 3.06 | 3.98 | 1.3E-03 | 3.9E-02 | OPT4_ARATH | 0 |
| DN15751_c0_g1_i1 | 3.77 | 0.33 | 1.3E-03 | 3.9E-02 | #N/A | #N/A |
| DN15159_c0_g1_i2 | -6.23 | 1.14 | 1.3E-03 | 3.9E-02 | NNJA5_MAIZE | 5.51E-57 |
| DN21892_c0_g1_i15 | 1.61 | 2.29 | 1.3E-03 | 3.9E-02 | #N/A | #N/A |
| DN20858_c0_g1_i15 | 4.38 | 0.75 | 1.3E-03 | 3.9E-02 | LPCT2_ARATH | 7.74E-56 |
| DN15513_c2_g1_i3 | 8.56 | 0.46 | 1.3E-03 | 4.0E-02 | DRL4_ARATH | 2.23E-56 |
| DN21265_c0_g1_i14 | 5.08 | -0.48 | 1.3E-03 | 4.0E-02 | SWT14_ORYSI | 3.35E-48 |
| DN11408_c0_g1_i1 | -2.41 | 0.78 | 1.3E-03 | 4.0E-02 | WTR31_ARATH | 1.98E-102 |
| DN18578_c0_g1_i1 | -1.83 | 2.06 | 1.3E-03 | 4.0E-02 | #N/A | #N/A |
| DN15064_c0_g1_i5 | 1.98 | 1.63 | 1.3E-03 | 4.0E-02 | #N/A | #N/A |
| DN21649_c0_g1_i3 | -2.16 | 2.67 | 1.3E-03 | 4.0E-02 | KOR1_ORYSJ | 0 |
| DN18772_c2_g1_i1 | -5.48 | -0.64 | 1.3E-03 | 4.0E-02 | THF1_ORYSJ | 1.11E-107 |
| DN15598_c0_g7_i1 | 2.89 | 0.66 | 1.3E-03 | 4.0E-02 | #N/A | #N/A |
| DN22659_c1_g1_i3 | 0.86 | 5.45 | 1.3E-03 | 4.0E-02 | R27A3_ARATH | 6.69E-75 |
| DN18241_c2_g7_i5 | 7.69 | -0.34 | 1.3E-03 | 4.0E-02 | #N/A | #N/A |
| DN18957_c0_g2_i8 | 8.65 | 0.55 | 1.3E-03 | 4.0E-02 | Y8219_ORYSJ | 0 |
| DN18654_c3_g7_i2 | 1.57 | 3.59 | 1.3E-03 | 4.0E-02 | COBL1_ORYSJ | 0 |
| DN16247_c0_g3_i1 | 2.21 | 2.11 | 1.3E-03 | 4.0E-02 | ADT5_ARATH | 9.43E-99 |
| DN17436_c0_g1_i1 | 7.71 | -0.33 | 1.3E-03 | 4.0E-02 | PTR2_ARATH | 0 |
| DN20193_c0_g1_i4 | -1.85 | 1.86 | 1.3E-03 | 4.0E-02 | #N/A | #N/A |
| DN16232_c1_g2_i3 | 2.95 | 3.12 | 1.3E-03 | 4.1E-02 | ODBA2_ARATH | 0 |
| DN14612_c1_g1_i20 | 2.40 | 2.46 | 1.4E-03 | 4.1E-02 | #N/A | #N/A |
| DN16097_c0_g1_i8 | 2.74 | 1.56 | 1.4E-03 | 4.1E-02 | #N/A | #N/A |
| DN15145_c0_g1_i1 | 4.44 | 1.45 | 1.4E-03 | 4.1E-02 | #N/A | #N/A |
| DN15825_c1_g2_i4 | -4.93 | 4.52 | 1.4E-03 | 4.1E-02 | TRPC_ARATH | 7.45E-163 |
| DN20397_c2_g6_i3 | -1.23 | 2.93 | 1.4E-03 | 4.1E-02 | MPK17_ORYSJ | 0 |
| DN15904_c0_g3_i1 | -1.60 | 2.62 | 1.4E-03 | 4.1E-02 | THRC1_ARATH | 0 |
| DN19636_c0_g1_i9 | 0.87 | 6.08 | 1.4E-03 | 4.1E-02 | #N/A | #N/A |
| DN18522_c2_g2_i2 | 9.19 | 1.05 | 1.4E-03 | 4.2E-02 | GSO1_ARATH | 9.26E-44 |
| DN13668_c1_g2_i2 | -0.79 | 5.10 | 1.4E-03 | 4.2E-02 | #N/A | #N/A |
| DN18331_c2_g1_i5 | 3.00 | -0.08 | 1.4E-03 | 4.2E-02 | PANK1_ORYSJ | 6.47E-113 |
| DN17893_c0_g1_i2 | -5.63 | -0.17 | 1.4E-03 | 4.2E-02 | LRK41_ARATH | 0 |
| DN15013_c0_g1_i4 | -0.73 | 5.92 | 1.4E-03 | 4.2E-02 | #N/A | #N/A |
| DN18069_c1_g1_i8 | -4.81 | 0.48 | 1.4E-03 | 4.2E-02 | PUX1_ARATH | 6.41E-52 |
| DN20902_c0_g1_i23 | 8.15 | 0.07 | 1.4E-03 | 4.2E-02 | SUVH1_TOBAC | 0 |
| DN20041_c0_g3_i2 | 1.15 | 3.87 | 1.4E-03 | 4.2E-02 | #N/A | #N/A |
| DN21089_c0_g6_i2 | 9.83 | 1.68 | 1.4E-03 | 4.2E-02 | GSTU1_ORYSJ | 7.2E-54 |
| DN16319_c0_g2_i6 | 4.31 | 1.33 | 1.4E-03 | 4.2E-02 | #N/A | #N/A |
| DN21987_c0_g2_i5 | 9.12 | 0.99 | 1.4E-03 | 4.2E-02 | #N/A | #N/A |
| DN22411_c0_g3_i33 | 7.61 | -0.41 | 1.4E-03 | 4.2E-02 | C3H28_ORYSJ | 1.77E-100 |
| DN20679_c0_g7_i1 | 1.69 | 1.65 | 1.4E-03 | 4.2E-02 | #N/A | #N/A |
| DN22801_c2_g2_i2 | 7.65 | -0.37 | 1.4E-03 | 4.2E-02 | #N/A | #N/A |
| DN22762_c1_g3_i6 | 7.78 | -0.26 | 1.4E-03 | 4.3E-02 | #N/A | #N/A |
| DN14660_c0_g1_i5 | -0.98 | 5.22 | 1.4E-03 | 4.3E-02 | #N/A | #N/A |
| DN20405_c0_g4_i4 | 7.77 | 1.93 | 1.4E-03 | 4.3E-02 | GATP1_ORYSI | 1.17E-25 |
| DN20824_c1_g1_i27 | 8.06 | -0.01 | 1.4E-03 | 4.3E-02 | #N/A | #N/A |
| DN17314_c0_g2_i6 | 1.04 | 7.12 | 1.4E-03 | 4.3E-02 | MDAR_SOLLC | 0 |
| DN14911_c0_g4_i1 | -2.47 | 1.48 | 1.4E-03 | 4.3E-02 | #N/A | #N/A |
| DN14159_c0_g1_i1 | 1.32 | 4.21 | 1.4E-03 | 4.3E-02 | #N/A | #N/A |
| DN18919_c0_g1_i7 | 9.09 | 0.97 | 1.5E-03 | 4.3E-02 | DCE1_ARATH | 0 |
| DN19347_c0_g1_i7 | -1.64 | 4.73 | 1.5E-03 | 4.3E-02 | ALA2_PANMI | 0 |
| DN19347_c0_g1_i1 | -1.54 | 10.46 | 1.5E-03 | 4.3E-02 | ALA2_PANMI | 0 |
| DN14638_c0_g1_i5 | 8.19 | 0.11 | 1.5E-03 | 4.3E-02 | #N/A | #N/A |
| DN16827_c1_g1_i1 | 7.44 | -0.56 | 1.5E-03 | 4.3E-02 | AGD12_ARATH | 4.34E-141 |
| DN21130_c1_g1_i28 | -1.75 | 0.99 | 1.5E-03 | 4.4E-02 | CHMO_ARATH | 2.24E-71 |
| DN18153_c2_g3_i1 | 1.35 | 7.97 | 1.5E-03 | 4.4E-02 | #N/A | #N/A |
| DN20127_c0_g1_i5 | 2.19 | 1.68 | 1.5E-03 | 4.4E-02 | #N/A | #N/A |
| DN17491_c0_g2_i9 | 1.80 | 3.13 | 1.5E-03 | 4.4E-02 | FLXL1_ARATH | 4.52E-35 |
| DN15589_c0_g1_i5 | 3.01 | 0.50 | 1.5E-03 | 4.4E-02 | PP362_ARATH | 0 |
| DN15343_c0_g1_i13 | -1.23 | 2.76 | 1.5E-03 | 4.4E-02 | #N/A | #N/A |
| DN21010_c0_g1_i15 | 2.57 | 0.57 | 1.5E-03 | 4.4E-02 | MAP1B_ARATH | 2.5E-29 |
| DN16120_c0_g1_i1 | 7.25 | 2.22 | 1.5E-03 | 4.4E-02 | #N/A | #N/A |
| DN17545_c1_g3_i5 | 2.18 | 5.25 | 1.5E-03 | 4.4E-02 | OPT7_ARATH | 0 |
| DN21788_c1_g1_i1 | -1.26 | 9.22 | 1.5E-03 | 4.4E-02 | PSAO_ARATH | 1.75E-59 |
| DN20772_c0_g2_i3 | -1.64 | 2.89 | 1.5E-03 | 4.4E-02 | TRN1_ARATH | 0 |
| DN19554_c5_g5_i1 | 1.94 | 1.95 | 1.5E-03 | 4.4E-02 | #N/A | #N/A |
| DN21229_c0_g2_i9 | 3.96 | 1.14 | 1.5E-03 | 4.4E-02 | #N/A | #N/A |
| DN20375_c1_g1_i8 | 5.26 | -0.42 | 1.5E-03 | 4.4E-02 | #N/A | #N/A |
| DN21685_c0_g1_i6 | 3.93 | 0.47 | 1.5E-03 | 4.4E-02 | SEUSS_ARATH | 1.15E-84 |
| DN20899_c0_g2_i2 | -1.91 | 4.30 | 1.5E-03 | 4.5E-02 | ARMC8_BOVIN | 6.71E-31 |
| DN17570_c0_g2_i12 | 1.49 | 2.91 | 1.5E-03 | 4.5E-02 | POED1_ORYSJ | 0 |
| DN19619_c1_g1_i12 | -1.24 | 3.77 | 1.5E-03 | 4.5E-02 | #N/A | #N/A |
| DN21891_c0_g1_i3 | -1.22 | 3.28 | 1.5E-03 | 4.5E-02 | #N/A | #N/A |
| DN21717_c3_g2_i4 | 8.98 | 0.86 | 1.5E-03 | 4.5E-02 | PIP_ARATH | 0 |
| DN21803_c0_g2_i1 | -2.66 | 1.11 | 1.5E-03 | 4.5E-02 | C14B3_MAIZE | 1.16E-109 |
| DN20947_c6_g7_i1 | -2.07 | 2.57 | 1.5E-03 | 4.6E-02 | #N/A | #N/A |
| DN19070_c0_g2_i4 | -1.77 | 0.46 | 1.6E-03 | 4.6E-02 | #N/A | #N/A |
| DN17407_c0_g1_i1 | 7.63 | -0.39 | 1.6E-03 | 4.6E-02 | #N/A | #N/A |
| DN21426_c2_g1_i8 | -1.30 | 8.20 | 1.6E-03 | 4.6E-02 | CCD4_ARATH | 1.07E-163 |
| DN18544_c0_g1_i3 | 2.19 | 4.81 | 1.6E-03 | 4.6E-02 | P2C05_ORYSJ | 2.67E-80 |
| DN20542_c1_g7_i1 | -1.68 | 3.03 | 1.6E-03 | 4.6E-02 | #N/A | #N/A |
| DN18540_c0_g1_i13 | -0.79 | 4.65 | 1.6E-03 | 4.6E-02 | #N/A | #N/A |
| DN18572_c0_g1_i1 | 8.98 | 0.86 | 1.6E-03 | 4.6E-02 | AB25B_ORYSJ | 0 |
| DN15938_c2_g1_i14 | -0.72 | 6.72 | 1.6E-03 | 4.6E-02 | #N/A | #N/A |
| DN19618_c0_g1_i15 | -1.45 | 4.49 | 1.6E-03 | 4.6E-02 | SUT31_ARATH | 0 |
| DN20572_c3_g1_i3 | 7.40 | 2.62 | 1.6E-03 | 4.6E-02 | BOLA4_ARATH | 2.84E-36 |
| DN22669_c0_g1_i10 | 3.76 | 0.83 | 1.6E-03 | 4.6E-02 | RAD23_ORYSJ | 1.11E-62 |
| DN18886_c0_g1_i5 | 1.72 | 2.25 | 1.6E-03 | 4.6E-02 | KATL2_XENTR | 3.5E-121 |
| DN19044_c0_g3_i8 | 1.27 | 3.47 | 1.6E-03 | 4.6E-02 | EFTU_TOBAC | 9.19E-25 |
| DN20737_c0_g1_i12 | -0.74 | 8.44 | 1.6E-03 | 4.6E-02 | #N/A | #N/A |
| DN20174_c1_g2_i6 | 1.87 | 5.09 | 1.6E-03 | 4.7E-02 | #N/A | #N/A |
| DN14960_c0_g1_i8 | 3.57 | 0.33 | 1.6E-03 | 4.7E-02 | #N/A | #N/A |
| DN16518_c0_g2_i16 | 1.49 | 3.66 | 1.6E-03 | 4.7E-02 | TAF12_ARATH | 2.24E-44 |
| DN21972_c1_g1_i15 | 5.03 | 2.60 | 1.6E-03 | 4.7E-02 | COPB1_ORYSJ | 0 |
| DN16422_c4_g1_i7 | 2.32 | 1.52 | 1.6E-03 | 4.7E-02 | CSK2E_ARATH | 1.83E-81 |
| DN21556_c0_g1_i4 | 0.89 | 5.09 | 1.6E-03 | 4.7E-02 | METE2_ORYSJ | 0 |
| DN17720_c0_g1_i9 | 2.34 | 2.55 | 1.6E-03 | 4.7E-02 | ADCK1_HUMAN | 5.21E-47 |
| DN14607_c0_g3_i4 | -2.91 | -0.20 | 1.6E-03 | 4.7E-02 | MEE14_ARATH | 1.21E-23 |
| DN17649_c0_g1_i8 | -0.77 | 5.13 | 1.6E-03 | 4.8E-02 | AP1S1_ARATH | 6.33E-105 |
| DN18438_c0_g1_i1 | 4.12 | 1.64 | 1.6E-03 | 4.8E-02 | #N/A | #N/A |
| DN17448_c1_g2_i7 | -1.71 | 2.89 | 1.6E-03 | 4.8E-02 | MAD14_ORYSI | 5.19E-31 |
| DN16694_c0_g1_i11 | 3.79 | 1.46 | 1.7E-03 | 4.8E-02 | NAC8_ARATH | 3.89E-138 |
| DN14679_c0_g1_i2 | -1.07 | 3.32 | 1.7E-03 | 4.8E-02 | #N/A | #N/A |
| DN17390_c0_g2_i11 | -2.02 | 0.44 | 1.7E-03 | 4.8E-02 | #N/A | #N/A |
| DN18341_c0_g2_i1 | 1.00 | 4.86 | 1.7E-03 | 4.8E-02 | RMLCD_ARATH | 6.06E-84 |
| DN19083_c4_g4_i1 | 3.92 | -0.26 | 1.7E-03 | 4.9E-02 | HSFB1_ORYSJ | 5.72E-97 |
| DN19919_c1_g1_i19 | -2.13 | 1.60 | 1.7E-03 | 4.9E-02 | RPM1_ARATH | 6.57E-56 |
| DN17525_c2_g2_i5 | -0.97 | 4.07 | 1.7E-03 | 4.9E-02 | #N/A | #N/A |
| DN13617_c0_g1_i2 | -1.02 | 3.83 | 1.7E-03 | 4.9E-02 | GGH2_ARATH | 6.48E-70 |
| DN17717_c3_g1_i8 | -1.36 | 2.15 | 1.7E-03 | 4.9E-02 | JMJ25_ARATH | 6.69E-92 |
| DN16957_c0_g1_i12 | -1.03 | 3.67 | 1.7E-03 | 5.0E-02 | #N/A | #N/A |
| DN17489_c1_g1_i18 | -0.72 | 6.43 | 1.7E-03 | 5.0E-02 | APEH_MOUSE | 2.37E-102 |
| DN12026_c0_g1_i1 | -1.72 | 2.01 | 1.7E-03 | 5.0E-02 | #N/A | #N/A |
| DN14609_c0_g1_i3 | 1.14 | 3.71 | 1.7E-03 | 5.0E-02 | C81E1_GLYEC | 8.43E-51 |
| DN14414_c0_g1_i2 | 7.38 | -0.62 | 1.7E-03 | 5.0E-02 | #N/A | #N/A |
| DN17394_c0_g2_i2 | 1.00 | 5.33 | 1.8E-03 | 5.0E-02 | 4CL2_ORYSJ | 0 |
| DN21357_c0_g1_i9 | -4.53 | 2.82 | 1.8E-03 | 5.1E-02 | DNJ21_ARATH | 0 |
| DN15779_c0_g1_i5 | -1.19 | 2.94 | 1.8E-03 | 5.1E-02 | PMTH_ARATH | 0 |
| DN20769_c0_g2_i14 | -0.84 | 4.23 | 1.8E-03 | 5.1E-02 | ORR21_ORYSJ | 3.11E-137 |
| DN18735_c1_g1_i9 | -1.75 | 1.33 | 1.8E-03 | 5.1E-02 | MYB44_ARATH | 2.07E-31 |
| DN22828_c1_g1_i10 | -1.53 | 1.98 | 1.8E-03 | 5.1E-02 | #N/A | #N/A |
| DN17780_c0_g4_i3 | -1.87 | 2.97 | 1.8E-03 | 5.1E-02 | CATA3_MAIZE | 0 |
| DN18692_c1_g9_i7 | -1.47 | 2.36 | 1.8E-03 | 5.1E-02 | HIP1_ORYSJ | 3.78E-30 |
| DN16837_c0_g2_i10 | 8.48 | 0.39 | 1.8E-03 | 5.1E-02 | RH15_ORYSJ | 4.34E-140 |
| DN20533_c0_g1_i8 | -0.67 | 7.78 | 1.8E-03 | 5.1E-02 | AB22G_ARATH | 0 |
| DN17282_c0_g4_i2 | 7.28 | -0.71 | 1.8E-03 | 5.2E-02 | PGLR_VITVI | 4.27E-120 |
| DN15449_c0_g7_i1 | -1.88 | 1.67 | 1.8E-03 | 5.2E-02 | #N/A | #N/A |
| DN18979_c0_g3_i4 | 7.99 | 2.94 | 1.8E-03 | 5.2E-02 | RMA1_CAPAN | 1.95E-32 |
| DN20985_c1_g1_i11 | -1.19 | 2.71 | 1.8E-03 | 5.2E-02 | #N/A | #N/A |
| DN14429_c0_g1_i2 | 5.45 | -0.14 | 1.8E-03 | 5.2E-02 | #N/A | #N/A |
| DN14415_c0_g1_i1 | -0.81 | 6.14 | 1.8E-03 | 5.2E-02 | CLPS1_ARATH | 5.34E-50 |
| DN15294_c0_g1_i2 | -0.81 | 7.57 | 1.8E-03 | 5.2E-02 | #N/A | #N/A |
| DN22027_c0_g1_i9 | 1.16 | 3.20 | 1.8E-03 | 5.2E-02 | Y5158_ARATH | 8.02E-100 |
| DN21861_c1_g2_i29 | -1.73 | 1.97 | 1.8E-03 | 5.2E-02 | #N/A | #N/A |
| DN17348_c0_g3_i3 | -1.01 | 8.02 | 1.8E-03 | 5.2E-02 | PHT1A_ORYSJ | 0 |
| DN15006_c1_g9_i1 | 1.20 | 4.43 | 1.8E-03 | 5.2E-02 | AZG1_ARATH | 0 |
| DN16705_c0_g1_i5 | -0.80 | 8.23 | 1.8E-03 | 5.3E-02 | CB5_ARATH | 1.49E-138 |
| DN18884_c4_g2_i26 | 4.08 | 1.60 | 1.8E-03 | 5.3E-02 | #N/A | #N/A |
| DN16542_c1_g1_i5 | -1.99 | 0.52 | 1.9E-03 | 5.3E-02 | RR16_SORBI | 6.47E-45 |
| DN16558_c0_g1_i4 | 7.80 | -0.24 | 1.9E-03 | 5.3E-02 | #N/A | #N/A |
| DN21345_c0_g1_i5 | 7.43 | -0.57 | 1.9E-03 | 5.3E-02 | #N/A | #N/A |
| DN17033_c2_g2_i8 | 2.96 | 0.14 | 1.9E-03 | 5.3E-02 | #N/A | #N/A |
| DN22360_c1_g1_i1 | 1.19 | 3.19 | 1.9E-03 | 5.3E-02 | #N/A | #N/A |
| DN22733_c2_g1_i10 | -0.89 | 4.32 | 1.9E-03 | 5.4E-02 | PPA1_ARATH | 0 |
| DN11517_c0_g1_i2 | 1.37 | 3.96 | 1.9E-03 | 5.4E-02 | #N/A | #N/A |
| DN19820_c0_g5_i1 | 7.38 | -0.62 | 1.9E-03 | 5.4E-02 | RFS2_ARATH | 2.13E-37 |
| DN22811_c2_g4_i2 | 1.05 | 3.82 | 1.9E-03 | 5.4E-02 | ODPB2_ORYSJ | 3.07E-93 |
| DN17657_c1_g1_i14 | -1.51 | 2.13 | 1.9E-03 | 5.4E-02 | COPIA_DROME | 3.32E-130 |
| DN22341_c1_g1_i31 | 2.70 | 2.21 | 1.9E-03 | 5.4E-02 | #N/A | #N/A |
| DN20660_c0_g2_i4 | 8.39 | 0.31 | 1.9E-03 | 5.4E-02 | #N/A | #N/A |
| DN22359_c0_g1_i1 | 6.03 | 1.05 | 1.9E-03 | 5.4E-02 | LHTL1_ARATH | 4.35E-47 |
| DN14687_c4_g4_i4 | 1.40 | 3.07 | 1.9E-03 | 5.4E-02 | #N/A | #N/A |
| DN16368_c0_g4_i3 | -1.35 | 3.62 | 1.9E-03 | 5.4E-02 | #N/A | #N/A |
| DN14692_c0_g1_i2 | -1.64 | 1.01 | 1.9E-03 | 5.4E-02 | YSL14_ORYSJ | 0 |
| DN19087_c0_g3_i12 | 1.70 | 1.93 | 1.9E-03 | 5.4E-02 | #N/A | #N/A |
| DN18656_c1_g3_i8 | -0.79 | 5.01 | 1.9E-03 | 5.4E-02 | SUT33_ARATH | 5.2E-138 |
| DN18377_c0_g6_i1 | 1.28 | 3.39 | 1.9E-03 | 5.4E-02 | #N/A | #N/A |
| DN18657_c0_g1_i4 | 8.32 | 0.24 | 1.9E-03 | 5.4E-02 | #N/A | #N/A |
| DN20978_c0_g1_i5 | 8.97 | 0.85 | 1.9E-03 | 5.4E-02 | FRL4A_ARATH | 1.98E-93 |
| DN16140_c1_g2_i4 | 4.02 | 0.32 | 1.9E-03 | 5.4E-02 | PLDZ1_ARATH | 0 |
| DN22664_c0_g2_i4 | -2.13 | 1.07 | 1.9E-03 | 5.4E-02 | #N/A | #N/A |
| DN18143_c0_g2_i11 | 7.82 | -0.22 | 1.9E-03 | 5.5E-02 | #N/A | #N/A |
| DN21890_c0_g1_i1 | 1.62 | 3.35 | 1.9E-03 | 5.5E-02 | RHL1_ARATH | 1.01E-59 |
| DN15136_c0_g4_i1 | 1.51 | 2.05 | 1.9E-03 | 5.5E-02 | DIR21_ARATH | 8.43E-41 |
